# Large language models in materials science: assessing RAG evaluation frameworks through graphene synthesis

**DOI:** 10.1039/d5ra09726f

**Published:** 2026-02-27

**Authors:** Zen Han Cho, Matthew Osvaldo, Sayan Doloi, Maloy Das, Jun Ci Goh, Bo Sheng Tan, Jiali Wang, Yujia Li, Xingchi Xiao, Amrita Joshi, Leonard Wei Tat Ng

**Affiliations:** a School of Materials Science and Engineering, Nanyang Technological University 50 Nanyang Avenue 639798 Singapore leonard.ngwt@ntu.edu.sg

## Abstract

Retrieval-Augmented Generation (RAG) systems increasingly support scientific research, yet evaluating their performance in specialized domains remains challenging due to the technical complexity and precision requirements of scientific knowledge. This study presents the first systematic analysis of automated evaluation frameworks for scientific RAG systems, using graphene synthesis in materials science as a representative case study. We develop a comprehensive evaluation protocol comparing four assessment approaches: RAGAS (an automated RAG evaluation framework), BERTScore, LLM-as-a-Judge, and expert human evaluation across 20 domain-specific questions. Our analysis of automated evaluators reveals that BERTScore lacks the interpretability and score sensitivity required to distinguish meaningful performance difference, while LLM-as-a-Judge failed to capture retrieval augmentation benefits. In contrast, RAGAS successfully captured relative performance improvements from retrieval augmentation, identifying performance gains in RAG-augmented systems (0.52-point improvement for Gemini, 1.03-point for Qwen on a 10-point scale), and demonstrating particular sensitivity to retrieval benefits in smaller, open-source models. However, it still exhibits fundamental limitations in absolute score interpretation for scientific content. These findings establish methodological guidelines for scientific RAG evaluation and highlight critical considerations for researchers deploying AI systems in specialized domains.

## Abstract 2

Automated evaluation of RAG systems is increasingly used in scientific applications, yet its reliability remains unclear. Using graphene synthesis as a case study, this work systematically benchmarks three automated evaluators.

## Graphical abstract

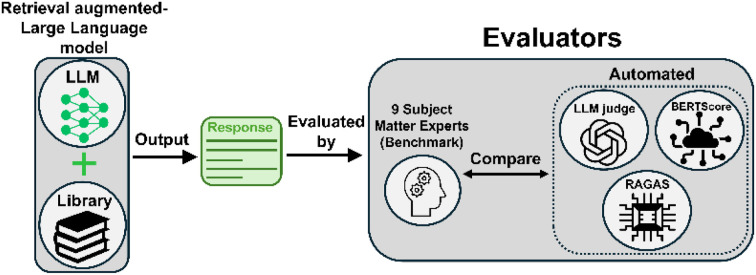

## Introduction

The integration of Large Language Models (LLMs) into scientific research workflows has accelerated rapidly, yet their evaluation in specialized domains remains methodologically underdeveloped. While general-purpose LLMs like GPT-3.5 demonstrate broad knowledge synthesis capabilities,^1,2^ their performance in technical fields like materials science presents unique evaluation challenges that existing frameworks inadequately address.

Retrieval-Augmented Generation (RAG)^3^ systems offer a promising solution to enhance LLM performance in scientific domains by incorporating domain-specific literature in real-time. Applications in fields such as education^4,5^ and medicine^6^ have demonstrated their potential for domain-specific tasks, while specialized models such as LLaMP,^7^ PaperQA^8^ and PaperQA2 (ref. 9) highlight their promise in scientific applications. Despite this progress, evaluation remains a major challenge, requiring frameworks capable of assessing not only factual accuracy but also the quality of scientific reasoning, context utilization, and domain-specific knowledge integration. Current evaluation approaches vary widely,^10–15^ with no established methodology for scientific applications where accuracy and precision are critical.

This gap is especially critical in materials science, where accurate knowledge synthesis can directly influence experimental design and research outcomes.^16^ Benchmarks such as GPQA^17^ and MaScQA^18^ have been developed to evaluate LLMs in the scientific domain, but they primarily rely on structured question formats. For example, the MaScQA dataset includes multiple-choice, matching, predefined numerical, and open numerical questions. While this structure ensures coverage across subfields, it falls short in evaluating open-ended questions. The significance of this shortcoming has been demonstrated in prior work,^19^ where an LLM chose the correct multiple-choice answer but produced flawed reasoning when asked to justify it, highlighting the risk of hallucinated reasoning in open-ended use.

Recent evaluation frameworks, such as RAGAS,^20^ propose comprehensive assessment of RAG systems through multiple metrics including factual correctness, context recall, and faithfulness. In contrast to MaScQA's structured formats, these approaches typically evaluate open-ended responses using an evaluator LLM.^21^ While promising,^22,23^ these frameworks have not been systematically validated in scientific contexts, where the nature of knowledge and the consequences of errors differ significantly from general applications.

**Fig. 1:**
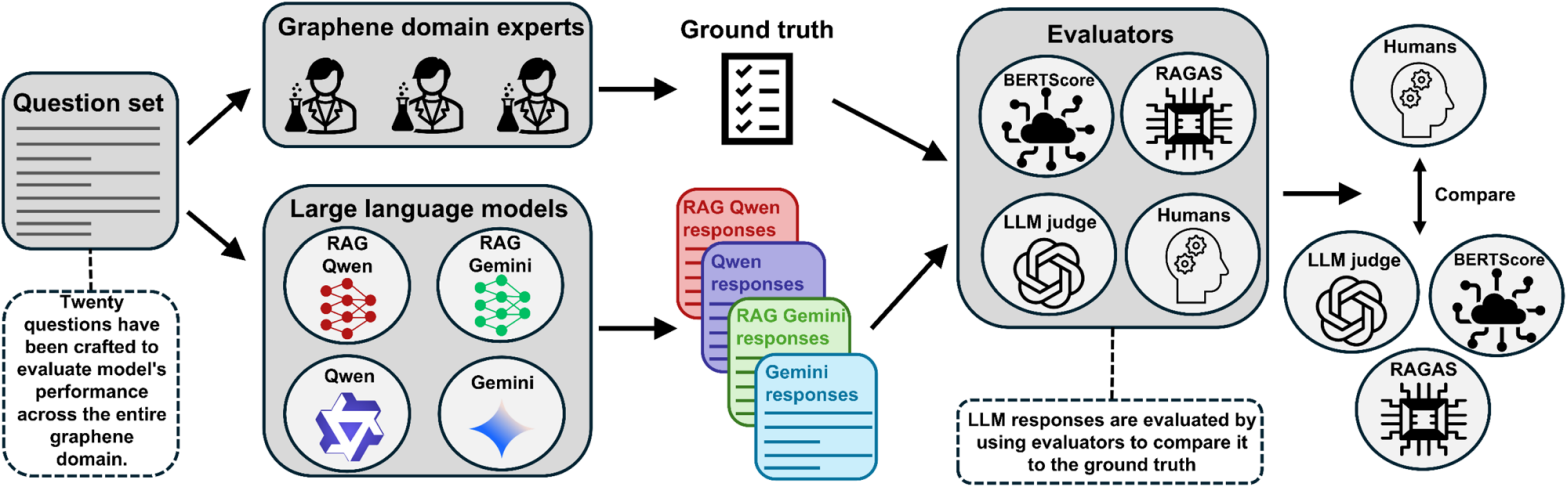
Overview of the evaluation workflow for assessing the performance of RAG-augmented and baseline LLMs. The figure shows the generation of answers from four LLM configurations (RAG-Qwen, RAG-Gemini, standard Qwen, and standard Gemini), and their evaluation using four methods: RAGAS, BERTScore, LLM judge, and a panel of subject matter experts. Human annotations serve as the benchmark for comparing the reliability and alignment of the automated evaluation metrics, with a focus on assessing the efficacy of RAGAS as a robust evaluation tool for RAG-LLMs.

This study addresses this gap by providing the first systematic evaluation of automated RAG assessment frameworks in a scientific domain. Using graphene synthesis as a representative case study, we investigate how well automated evaluation methods capture the performance characteristics essential for scientific applications. We evaluate four response modes, consisting of two RAG-LLM systems (Qwen and Gemini) and their baseline LLMs, using four evaluators: RAGAS, BERTScore, an LLM judge, and a panel of subject matter experts (Fig. 1). Our analysis reveals both the capabilities and fundamental limitations of current evaluation approaches, providing methodological guidance for researchers deploying RAG systems in specialized domains such as self-driving labs.^24,25^ The implications might extend beyond materials science to any technical field requiring precise knowledge synthesis, offering insights into the broader challenge of evaluating AI systems in specialized domains where accuracy and reliability are paramount.

## Experimental procedures

### Database preparation and RAG pipeline construction

We developed a domain-specific RAG pipeline by curating 300 peer-reviewed papers on graphene synthesis from leading scientific publishers including ScienceDirect, American Chemical Society Publications, and Springer Nature. Papers were selected based on detailed methodological descriptions of graphene production processes. For each paper, we extracted complete synthesis methodology sections, expanding technical abbreviations (GO: graphene oxide, RGO: reduced graphene oxide, CVD: chemical vapor deposition, PMMA: poly(methyl methacrylate)) to ensure contextual clarity and including complete bibliographic metadata.

Vector embeddings were generated using OpenAI's state-of-the-art^26^ text-embedding-3-large model *via* the LangChain framework, producing 3072-dimensional vectors optimized for semantic similarity retrieval. The embeddings were integrated into a Pinecone vector database configured for cosine similarity-based retrieval, with systematic batch processing to ensure efficient resource utilization.

### Question and ground truth establishment

To enable systematic evaluation across different question types, we developed a structured question taxonomy based on scientific query characteristics commonly encountered in materials science research. A materials science expert with extensive graphene research experience created 20 questions spanning four categories (Table S1, SI): major fabrication methods^27^ (8 questions), synthesis of graphene derivatives (6 questions), application-specific synthesis strategies (2 questions), and mechanistic understanding of materials and processes (4 questions).

Ground truth answers were established through expert consensus involving three materials science researchers with specialized knowledge in graphene synthesis and characterization.^28–30^ A lead expert first drafted the initial ground truth answers based solely on professional knowledge, without access to the RAG corpus. These answers were then independently reviewed by two additional experts under a double-blind protocol. Revisions were iteratively discussed until all three experts reached full consensus, at which point the ground truth answers were finalized. This procedure minimizes corpus-induced bias and ensures that the ground truth reflects genuine domain knowledge rather than database-specific information.

We note, however, that the relatively limited size and diversity of the dataset may not capture the full range of scientific reasoning challenges, potentially underrepresenting differences between models of varying scales.

### Obtaining responses from modes of questioning

To obtain a diverse range of responses for the Q-GT dataset, we employed four response modes: RAG-augmented and standard versions of two LLMs, Gemini-2.5-Flash and Qwen2.5-7B-Instruct. Gemini, released by Google on June 17, 2025, and freely accessible *via* a public API, was run with a default temperature of 1.0 and top p of 0.95 to balance determinism and variability. Qwen, an open-source model developed by Alibaba Cloud and accessed through the Hugging Face Transformers library, was prompted in a chat format with a system message followed by the user query. Tokenization was handled by “AutoTokenizer”, and responses were generated with a maximum of 1028 tokens.

**Fig. 2:**
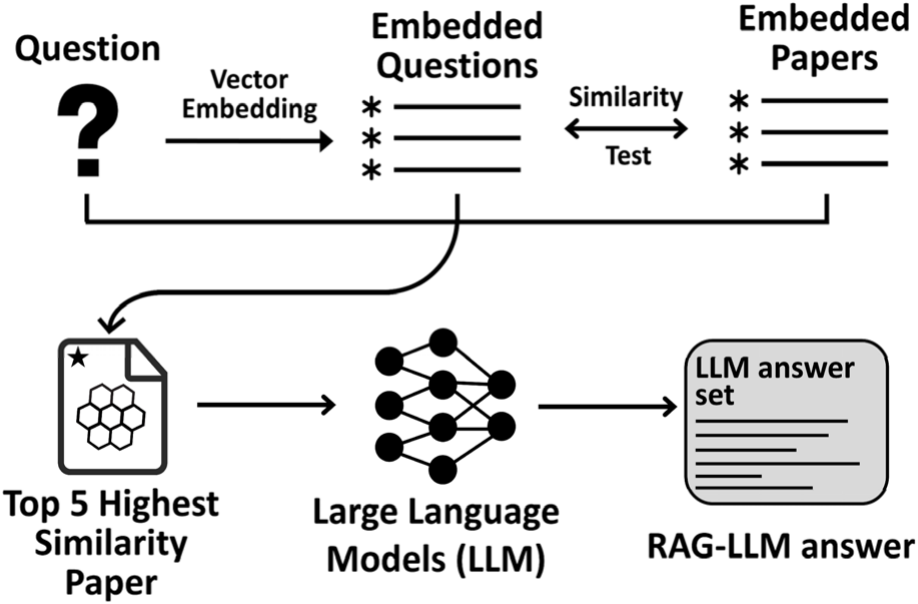
RAG-LLM workflow for response generation. User queries are transformed into vector embeddings and compared against the embedded research papers in the Pinecone database. The top five most similar papers are retrieved, and their contexts are combined with the original query in a structured prompt, which is subsequently passed to the LLM to generate the final RAG-LLM answer.

To generate responses with the RAG-LLM, queries are embedded using the same process described in database preparation, and the five most similar contexts are retrieved from the Pinecone database *via* cosine similarity. These contexts are then combined with the query in a prompt template (Section S2, SI) to produce contextually informed responses (Fig. 2). The baseline modes used the same LLMs without retrieval, providing a clear reference point for assessing the impact of augmentation on response quality.

### Comprehensive evaluation framework

**Fig. 3:**
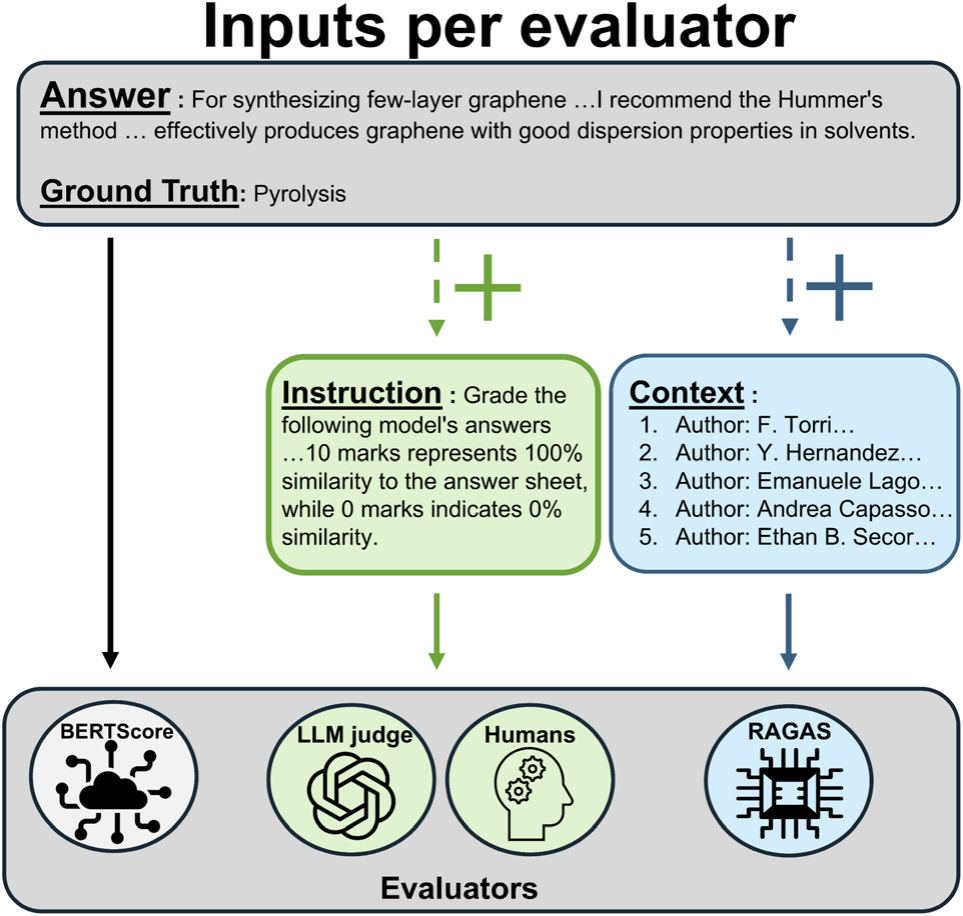
Workflow of the four evaluation approaches. All evaluators require both the model generated answer and the ground truth as inputs. BERTScore uses only these to compute semantic similarity. RAGAS, in addition to the model answer and ground truth, also incorporates the retrieved context to assess context recall and faithfulness. LLM judge and human evaluations depend on clearly specified instructions or rubrics, as they do not follow a standardized scoring framework like BERTScore or RAGAS.

We implemented four distinct evaluation approaches to assess both absolute performance and relative sensitivity to retrieval augmentation. An overview of the inputs and evaluation setup across all four methods, BERTScore, LLM judge, expert human evaluation, and RAGAS, is illustrated in Fig. 3.

(1) RAGAS metrics: we evaluate our RAG pipeline using RAGAS, an open-source framework that implements multiple quantitative metrics for comprehensive RAG system assessment we employed three core RAGAS metrics using GPT-4o as the evaluator with temperature set to 0 for consistency: Factual Correctness (FC), Context Recall (CR), and Faithfulness (FF).

FC assesses overlap between generated responses and ground truth using claim-level decomposition. RAGAS offers three evaluation modes for this metric: precision, recall, and *F*_1_ score. In this study, we adopt the recall mode, as it offers the most intuitive interpretation and is more easily transferable when replicating the evaluation process with a panel of human experts.
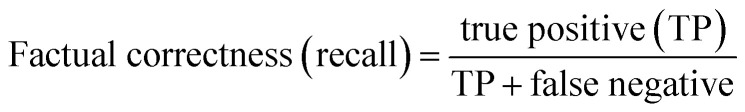


CR measures alignment between retrieved context and ground truth. This metric is calculated as:
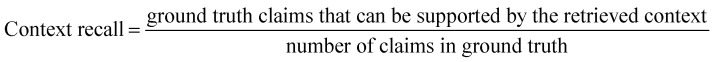


CR effectively captures the retriever's ability to surface relevant documents.

FF quantifies how well generated answers remain grounded in provided context, measuring the proportion of answer claims inferable from retrieved context, scoring from 0 to 1 (higher values indicate better alignment):
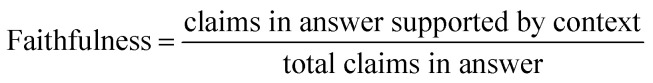


(2) BERTScore metric: to compare against the FC metric, we also employed BERTScore^31^ as an alternative method for evaluating semantic alignment between generated answers and ground truths. We used the facebook/bart-large-mnli model *via* Hugging Face transformers, employing recall mode for consistency with RAGAS factual correctness evaluation. All inputs were verified to remain within the 1022 token limit.

(3) LLM-as-a-judge (LLM Judge) metric: we implemented a custom evaluation approach using GPT-4o (temperature 0) with carefully engineered prompts (Section S3, SI) instructing the model to assess response quality on a 0–10 scale, similar to academic grading rubrics. The evaluation criteria emphasized content similarity and factual alignment with ground truth. Similar evaluation schemes have also been proposed in other domains, such as PaperBench,^32^ which evaluates the ability of LLM agents to reproduce machine learning papers from scratch using hierarchical rubrics and LLM judges.

(4) Expert human evaluator metric: nine subject matter experts independently evaluated responses using the same instructions and 0–10 scoring criteria as the LLM judge approach. Each response was evaluated by exactly three experts, with averaged scores used for analysis. Experts were blinded to response sources, and response positions were randomized across evaluation sheets to minimize bias.

## Results

### Performance patterns across evaluation methods

The evaluation revealed distinct patterns in how different assessment approaches capture RAG system performance. Human evaluators assigned the highest average scores (mean = 6.41, *σ* = 1.89), followed by LLM judge (6.00, *σ* = 2.24), BERTScore (5.89, *σ* = 0.70), and RAGAS (3.76, *σ* = 2.38). The substantial variation in both central tendency and dispersion across evaluators highlights fundamental differences in how each approach interprets and scores scientific content.

### Retrieval augmentation impact analysis

Human evaluation revealed clear performance hierarchies demonstrating the value of retrieval augmentation for scientific applications. RAG-Gemini achieved the highest average score (6.92), followed by RAG-Qwen (6.68), standard Gemini (6.37), and standard Qwen (5.68). The performance improvements from retrieval augmentation were substantial: 0.55 points for Gemini and 1.00 points for Qwen, representing 9% and 17% relative improvements respectively.

Notably, within the evaluated dataset, the impact of retrieval was more pronounced for smaller open-source models: RAG-Qwen not only exceeded the performance of standard Gemini despite its smaller size (7B *vs.* Gemini's larger scale) but also showed nearly twice the relative gain in FC compared to Gemini-2.5-Flash. This demonstrates that retrieval enhances smaller open-source models to the point where they can compete effectively with larger proprietary alternatives in domain-specific applications.

### Automated evaluator alignment analysis

**Fig. 4:**
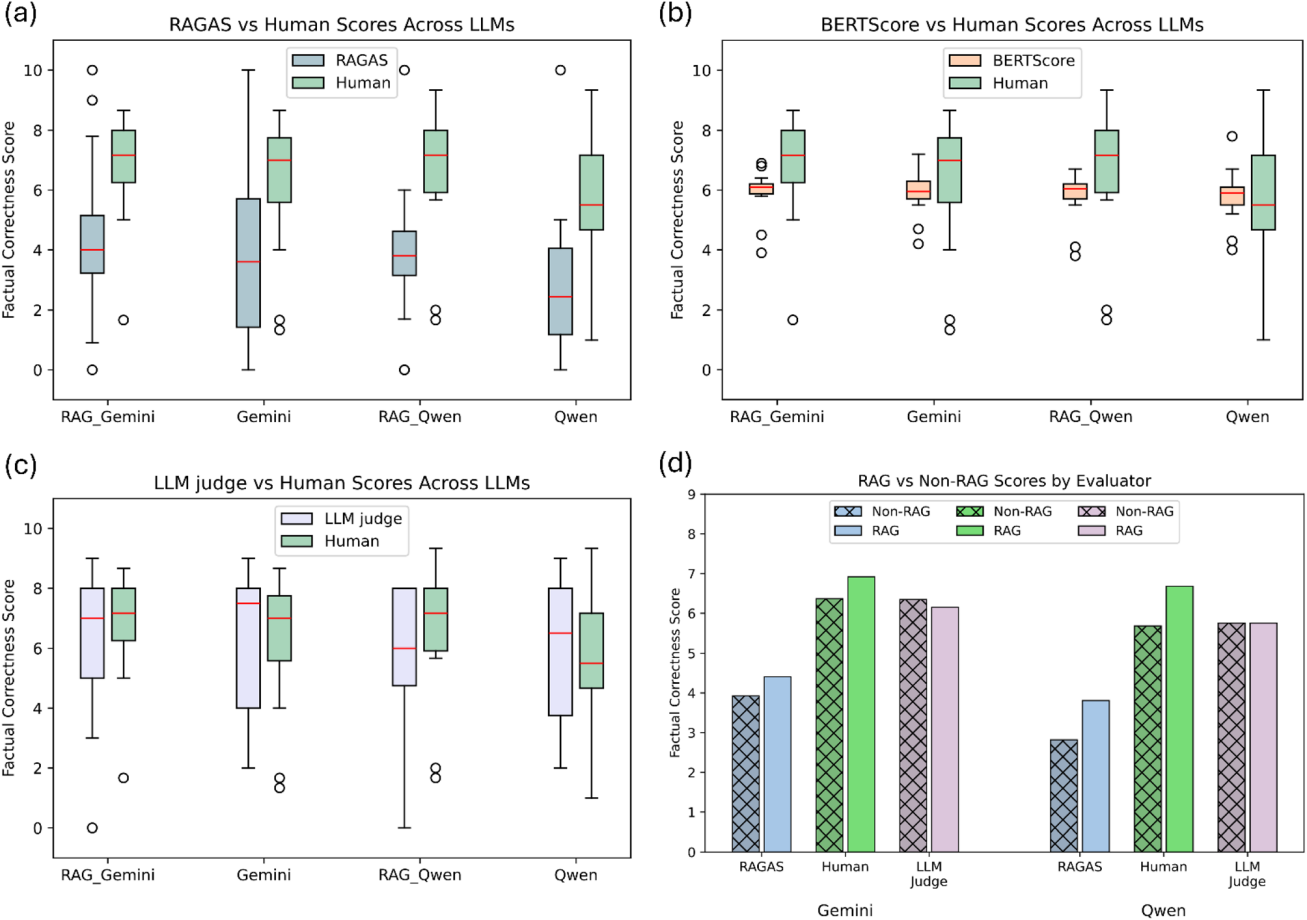
Comparison of factual correctness scores across LLMs and evaluators. (a) RAGAS *vs.* human scores for RAG and non-RAG variants of Gemini and Qwen. RAGAS underestimates factual correctness scores and shows poor alignment with human evaluation. (b) BERTScore *vs.* human scores across same LLMs. BERTScore show low variance in scores. (c) LLM Judge *vs.* human scores, showing the closest alignment in both distribution and median values. (d) Mean factual correctness scores across RAG and non-RAG LLMs by evaluator. Human and RAGAS reflect consistent factual gains from RAG augmentation, unlike LLM judge.

The relationship between automated evaluators and human judgment reveals critical insights about evaluation framework reliability in scientific contexts. RAGAS exhibited the largest absolute deviation from human scores (73.5% average difference) yet demonstrated the highest sensitivity to retrieval-augmented performance improvements (Fig. 4a). RAGAS successfully captured the relative performance gains observed by human evaluators: 0.52-point improvement for Gemini (*vs.* 0.55 human-observed) and 1.03-point improvement for Qwen (*vs.* 1.00 human-observed).

BERTScore showed minimal absolute deviation (8.78%) but suffered from restricted score distribution (*σ* = 0.70), clustering most outputs between 5.19–6.59 (Fig. 4b). When applied to human evaluation patterns, only 24% of human scores fell within BERTScore's expected range, indicating poor alignment with human scoring patterns despite superficial agreement in average scores. Consequently, BERTScore lacks the interpretability and responsiveness needed for evaluating factual correctness.

LLM judge demonstrated both low absolute deviation (7.53%) and appropriate score distribution (*σ* = 2.24), providing the closest overall alignment with human evaluation patterns (Fig. 4c). However, LLM judge failed to capture retrieval augmentation benefits consistently, incorrectly favouring standard Gemini over RAG-Gemini and showing minimal differentiation between Qwen variants (Fig. 4d).

To capture RAG system behaviour beyond factual correctness, RAGAS reports two additional component metrics. These metrics cannot be used for comparative assessment, as they are only defined for RAG-augmented models.

(1) Context recall (CR): inter-model CR scores were nearly identical (Table 1), as expected under fixed retrieval conditions, with minor variation reflecting the LLM-based nature of RAGAS. At the question level, however, CR scores varied substantially (mean = 3.92, *σ* = 3.26), with several questions receiving zeros. Two main factors accounted for this. First, the metric struggled to recognize domain-specific terminology; for example, Question 16 retrieved factually correct and relevant contexts yet still scored zero. Second, genuine retrieval failures occurred in cases requiring deeper expertise. Question 20, which probed mechanistic understanding, consistently showed poor retrieval, underscoring the limitations of similarity-based retrieval approaches for complex scientific reasoning (Section S4, Question 20, SI).

**Table 1:** Context recall and faithfulness scores (mean ± standard deviation) for LLMs^a^

Large language model	Context recall	Faithfulness
RAG-Gemini	3.92 ± 3.29	9.20 ± 1.54
RAG-Qwen	3.91 ± 3.23	7.32 ± 2.21

a RAGAS component metric analysis.

(2) Faithfulness: FF scores demonstrated model-specific patterns, with RAG-Gemini showing higher consistency (9.20 ± 1.54) compared to RAG-Qwen (7.32 ± 2.21). The higher variability in RAG-Qwen's scores indicates fluctuations in maintaining faithfulness to retrieved information, reflecting differences in contextual grounding during generation. For instance, in Question 19, both models retrieved identical relevant contexts, but RAG-Qwen produced unfaithful reasoning (FF = 4.0, FC = 1.7) as shown in Section S6 (SI), whereas RAG-Gemini remained faithful (FF = 7.1) and achieved a much higher factual correctness score (FC = 8.0).

## Discussion

### Methodological implications for scientific RAG evaluation

Our findings reveal that automated evaluation frameworks face fundamental challenges when applied to scientific content, reflecting the specialized nature of scientific knowledge and reasoning. For instance, BERTScore and LLM judge exhibit evaluator-specific limitations, namely restricted score distributions and failure to consistently capture retrieval augmentation benefits, respectively. In the case of RAGAS, Question 16 highlighted a structural weakness of the CR metric: despite retrieval of factually correct and relevant context, the score was zero, revealing its inability to map domain-specific terminology and limiting its utility in scientific applications.

By contrast, the FC metric was sensitive to retrieval augmentation benefits, but its poor absolute alignment with human judgment restricts its value for standalone assessment. The FF metric added complementary insights by capturing nuanced differences in how LLMs maintained contextual grounding, with these patterns further validated by corresponding FC scores.

Taken together, these observations indicate that RAGAS is most effective for comparative studies of RAG-based question and answering systems, where relative performance differences are the primary focus, rather than for applications requiring precise absolute performance levels. In such contexts, particularly those involving complex reasoning, expert human evaluation remains essential for determining deployment readiness.

### Framework selection guidelines for scientific applications

Based on our systematic comparison, we propose the following guidelines for evaluation framework selection in scientific RAG applications: For comparative studies evaluating factual correctness of multiple RAG systems under identical conditions, RAGAS provides quick and reliable relative performance assessment despite absolute score limitations. Among its component metrics, FF remains useful for capturing additional dimensions of evaluation, whereas CR requires further refinement before it can be reliably applied in scientific contexts.

For standalone system evaluation where absolute performance interpretation is critical, expert human evaluation remains the gold standard. However, resource constraints may call for hybrid strategies in which LLM judges provide preliminary screening, with human evaluation applied only once outputs surpass a quality threshold warranting closer assessment. For rapid development cycles where frequent evaluation is needed, LLM judge approaches offer the best balance of human alignment and practical accessibility, though their limitations in capturing retrieval benefits must be considered.

BERTScore, despite its superficial alignment with human averages, provides insufficient discrimination and should be avoided for scientific evaluation where subtle performance differences are important.

### Limitations and future directions

Our study focuses specifically on graphene synthesis, limiting direct generalizability to other scientific domains. However, the methodological insights about evaluation framework behavior likely extend to other technical fields with similar requirements for precision and domain expertise.

Future research should explore domain-adapted evaluation frameworks that incorporate scientific reasoning patterns and field-specific accuracy requirements. Developing evaluation approaches that can reliably assess conceptual understanding and reasoning quality, rather than primarily factual recall, represents a critical need for advancing AI applications in scientific research.

## Conclusion

This study provides a systematic analysis of automated evaluation frameworks for scientific RAG systems, revealing both capabilities and fundamental limitations of current approaches. While RAGAS's factual correctness metric can effectively capture relative performance improvements from retrieval augmentation, it exhibits significant challenges in absolute score interpretation for scientific content.

Our findings suggest that RAG systems can provide meaningful performance improvements for scientific applications, particularly for smaller open-source models, narrowing the performance gap with larger proprietary alternatives in our evaluation setting. However, the evaluation challenges identified highlight the need for more sophisticated assessment approaches tailored to scientific domains.^33–36^

The methodological guidelines developed through this analysis provide practical guidance for researchers deploying RAG systems in scientific applications. By understanding when different evaluation approaches succeed or fail, researchers can make informed decisions about evaluation strategies appropriate for their specific needs and constraints.

Beyond the immediate findings, this work establishes a foundation for developing evaluation methodologies appropriate for AI systems in specialized domains where accuracy and reliability are paramount. The insights gained might extend beyond materials science to any technical field requiring precise knowledge synthesis and reasoning capabilities.

## Author contributions

Z. H. C. was responsible for the conceptualization, data curation, investigation, design of methodology, implementation and validation of the computer code, and writing of the manuscript. M. O. was responsible for the data collection, design of methodology and reviewing of the manuscript. S. D., M. D. and X. X. were responsible for data collection and reviewing of the manuscript. J. C. G. was responsible for data curation and data collection. B. S. T., J. W., Y. L. and A. J. were responsible for data collection. L. W. T. N. was responsible for conceptualization, data collection, design of methodology, provision of computing resources, review and editing of the manuscript.

## Conflicts of interest

There are no conflicts to declare.

## Supplementary Material

RA-016-D5RA09726F-s001

## Data Availability

Code used to carry out the project and data for RAG training and RAGAS evaluation can be found at https://doi.org/10.17632/ry7phxn4js.3. Supplementary information (SI): (i) a 20-question graphene-synthesis question–ground truth dataset spanning multiple synthesis and processing routes, (ii) the exact prompt templates used for RAG and standard models to enable answer generation, (iii) the LLM-as-a-judge evaluation prompt , and (iv) the complete answer sets for all questions from each evaluated configuration (RAG-Gemini, Gemini, RAG-Qwen, and Qwen), to provide clear documentation of the dataset, prompts, and model outputs used in the analysis. See DOI: https://doi.org/10.1039/d5ra09726f.
