# Supplementary material for: Large language models in materials science: assessing RAG evaluation frameworks through graphene synthesis
Source: RSC Adv. 2026 Feb 27;16(13):11306–13. doi: 10.1039/d5ra09726f (PMC12947896; doi:10.1039/d5ra09726f)
Supplement: RA-016-D5RA09726F-s001 [file RA-016-D5RA09726F-s001.pdf]

Supplementary Information (SI) for RSC Advances.
This journal is © The Royal Society of Chemistry 2026

Supplementary information for:

Large Language Models in Materials Science: Assessing RAG Evaluation Frameworks through graphene synthesis

Zen Han Cho, Matthew Osvaldo, Sayan Doloi, Maloy Das, Jun Ci Goh, Bo Sheng Tan, Jiali Wang, Yujia Li, Xingchi Xiao, Amrita Joshi and Leonard Wei Tat Ng*

School of Materials Science and Engineering, Nanyang Technological University, 50 Nanyang Avenue, Singapore 639798

Table of Contents

S1. Question - Ground truth dataset 4
S2. Prompts for answer generation 8
  Prompt for RAG models 8
  Prompt for standard models 8
S3. Prompt for answer evaluation 9
  Prompt for LLM-as-a-judge (LLM Judge) metric 9
S4. Answer set for RAG-Gemini 10
  Question 1 10
  Question 2 14
  Question 3 18
  Question 4 23
  Question 5 27
  Question 6 31
  Question 7 34
  Question 8 38
  Question 9 42
  Question 10 46
  Question 11 50
  Question 12 55
  Question 13 60
  Question 14 64
  Question 15 68
  Question 16 72
  Question 17 76
  Question 18 80
  Question 19 83
  Question 20 87

S5. Answer set for Gemini 91
Question 1 91
Question 2 92
Question 3 93
Question 4 94
Question 5 95
Question 6 96
Question 7 97
Question 8 98
Question 9 99
Question 10 100
Question 11 101
Question 12 102
Question 13 103
Question 14 104
Question 15 105
Question 16 106
Question 17 107
Question 18 108
Question 19 109
Question 20 110
S6 Answer set for RAG-Qwen 111
Question 1 111
Question 2 115
Question 3 119
Question 4 124
Question 5 128
Question 6 132
Question 7 135
Question 8 139
Question 9 143
Question 10 147
Question 11 151
Question 12 155
Question 13 160
Question 14 163
Question 15 166

Question 16......170
Question 17......174
Question 18......178
Question 19......181
Question 20......185
S7. Answer set for Qwen......188
Question 1......188
Question 2......189
Question 3......190
Question 4......191
Question 5......192
Question 6......193
Question 7......194
Question 8......195
Question 9......196
Question 10......197
Question 11......198
Question 12......199
Question 13......200
Question 14......201
Question 15......202
Question 16......203
Question 17......204
Question 18......205
Question 19......206
Question 20......207

207 pages in total

S1. Question - Ground truth dataset

No	Question	ground truth
1	What are the steps involved to synthesize graphene via chemical vapour deposition?	First, a substrate, such copper, is cleaned, prepared and placed into a CVD chamber that is usually a tube made out of an inert material such as quartz. After which, the CVD chamber is evacuated into a vacuum or flooded with inert gases.The CVD chamber is then heated to some kind of critical temperature (about 1000 degrees celsius), before feedstock or precursor gases with high hydrocarbon contents are pumped into the CVD chamber. The high temperature in the chamber decomposes these feedstock gases into carbon atoms which then deposit onto the substrate's surface where they reassemble to into the characteristic graphene lattice. The feedstock gases also produce hydrogen which do not contribute to the reaction. The choice of substrate matters as it plays a role in catalysing the reaction. The temperature and duration of this deposition is controlled to either form mono-layer or few-layer graphene.
2	What are the steps involved to synthesize graphene via mechanical exfoliation?	Mechanical exfoliation of graphene, also known as the sticky tape method, involves first taking a piece of graphite, and then using a piece of sticky tape release a small layer of graphite onto it. This is done simply by placing the sticky tape repeatedly on the piece of graphite. To produce single atomic sheets of graphene, this tape is folded in half and repeatedly pulled apart, thereby shearing layers of graphene from the already deposited material until only a single-atomic layer is present.
3	What are the steps involved to synthesize graphene via plasma enhanced chemical vapour deposition?	The plasma-enhanced process is a modification of traditional chemical vapour deposition. As with traditional CVD, a prepared substrate (cleaned and heated to about 1000 degrees celsius) is inserted into a a CVD chamber made out of an inert material and gaseous precursors such as methane are introduced into the reaction chamber together with other inert gases such as H2 or Ar. Often plasma-enhanced CVD can use lower temperatures than traditional CVD. A plasma, typically generated by radio frequency energy at 13.56Mhz is used to excite and disassociate the precursor gases. This process creates highly reactive carbon radicals which deposit and self-assemble ont he heated substreate forming graphene layers. The growth can be tailored by adjusting parameters such as gas flow rates, plasma power and time.
4	What are the steps involved to synthesize graphene via electrochemical exfoliation?	First, an electrochemical set up is made involving a graphite working electrode, a counter electrode, a referene electrode and electrolyte and a power supply. All electrodes are inserted into the liquid electrolyte. Using the power supply, either a positive or negative potential is applied to the graphite electrode which either attracts anions (positive potential) or cations (negative potential) from the graphite electrode which cases expansion. The graphene can be subsequently exfoliated by mixing in the precursor..

5	What are the steps involved to synthesize graphene via arc discharge?	Graphene synthesis using arc discharge usises an electric arc to vaporise graphite,followed by the subsequest depostion of the graphene atoms and the formation of graphene flakes. You first need an arc reactor, with two water-cooled electrodes. The anode is usually filled with graphite which the cathode is a graphite rod. An electric arc is then established between the electrodes, using a high current (200A and under a specific pressure (e.g 500 Torr). This high temperature of the arc vaporises the graphite at the anode. The vaporised carbon atoms travel through the arc plasma and deposit on the chamber walls as graphene flakes.
6	What are the steps involved to synthesize graphene via pyrolysis?	Pyrolysis is done by first transforming organic materials like biomass into a carbonaceous material followed by further processing into graphene structures. First, biomass sources, such as rice husks or wheat straw are selected and pretreated by grinding, drying or in some cases, treated with chemicals like KOH to enhance carbonization. Pyrolysis is then carried out in a controlled, inert environment in a furnace or reactor. The temperature is gradually increased in the chamber in the reactor (typically between 600 to 1,200 degrees C), which causes the precursor material to decompose, thereby releasing volatile components and leaving behind a carbonaceous residue. The carbonaceous material is then post processed such as acid washing and annealing to improve the material's structural order to become graphene.
7	What are the steps involved to synthesize graphene via liquid phase exfoliation?	Graphite is first placed into some kind of liquid suspension medium, often ethanol, alcohol or some other kind of solvent, and then sonicated either through bath sonication or tip sonication. In bath sonication, the graphite-liquid suspension is usually placed into some kind of container before being placed in the bath sonicator. In tip sonication, the tip is inserted directly into the liquid. The suspension is then sonicated for a period of time. During the sonication, cavitation within th eliquids will cause the different graphene layers to exfoliate from one another. After a certain period of time, the suspensions are removed from the sonicator and then centrifuged. The supernatent of the liquid is then taken and characterised to confirm the presence of mono-layer or few-layer graphene. Surfactants can be utilised to maintain or improve the suspension but might cause issues with final device properties.

8	What are the steps involved to synthesize graphene via laser ablation?	Porous graphene can be made using the laser ablation of a nickel-graphite target under the flow of Argon gas. Although not technically a traditional graphene layer, porous graphene is a similar carbon allotrope with an inhomogenous structure and curvature on the graphene layers. First graphite and nickel particles are ball milled and then pressed into a graphite-nickel pellet under pressure to form a target. The target is then placed into a quartz tube where argon is allowed to flow at about 2.4l/min. The tube is then heated to 1050 degrees celsius and presurised at 300mbar. A Nd-YAG laser (1064nm in wavelength), with a 10 ns pulse duration and repetition rate of 10Hz is then focused on the carbon pellet. The illuminater area is about 12 mm2. The carbon atoms generated by this ablation are carried by the argon gas and then gathered on a collector on a water-cooled surface at the end of the tube and scraped off as porous graphene.
9	What are the steps involved to produce graphene oxide?	Graphene oxide (GO) can be synthesized by the oxidation of graphite into graphite oxide followed by the exfoliation of graphite oxide into GO. The leading method right now is the modified Hummer's method. A common procedure is as follopws: First, mix graphite and potassium permanganate in a 1: 6 ratio by weight. Then, in another beaker, mix 96% sulphuric acid and then add 75% phosphotic acid. The acids should be in a 6:1 ratio. Ensure the acids are added to the graphite and potassium permanganate slowly and bit by bit while constantly stirring. This allows for the intercalation of the graphite and oxidation of the graphite into graphite oxide. Stir the entire solution at 50 degrees centigrade for 12 hours and then allow to cool to room temperature. Next, pour the entire acid mixture into another beaker with ice made from de-ionised water. Add about 3mL of 30% hydrogen peroxide to the mixture and stire to allow the GO to exfoliate.
10	What are the steps involved to produce reduced graphene oxide?	After GO is produced from the modified Hummer's method the GO can be reduced chemically using a reducing agent such as hydrazine, ascorbic acid or even sugars to reduce it to reduced graphene oxide (rGO). Alternatively, GO can be thermally reduced by heating it up at high temperature in an inert atmosphere like argon or in the presence of hydrogen. This will break down the oxygen-containing groups on the GO causing them to volatilize and restore the carbon framework. it should be nmoted that this process is often incomplete and bcan produce rGOP with a lot of defects that affect conductivity.
11	What are the steps involved to produce graphene quantum dot?	Graphene Quantum Dots (GQDs) can be produced using hydrothermail reactions, laser ablation, microwave radiation and electrochemical oxidation. THe most straightforward is that of electrochemical oxidation. The electrochemical oxidation process involves the electrochemical cleavage of carbon precursores such as graphite into GQDs, typically under high voltage. First a potential is applied to a graphite electrode immersed in an electrolyte made of citric acid and sodium hydroxide. A second graphite rod is used as an cathode in the same solution. A DC power supply can provide the working bias voltage. The GQDs eventually exfoliate and dispersed into the electrolyte. The resulting GQDs can be purified by centrifugation and further techniques such as rotary evaporation.

12	What are the steps involved to produce graphene nanoribbon?	Graphene nanoribbons (GNRs) are narrow strips of single-layer graphene, essentially 'slices' of graphene that are one-dimensional. They have very high surface areas. They can be synthesized either by top-down approaches such as micromechanical cleavage or bottom-up approaches such as CVD. Top down approaches usually use multi-walled Carbon nanotubes (MWCNTs) to form these graphene nanoribbons. For instance usuing the hummers method on MWCNTs to form the ribbons instead of bulk graphite. Bottom up-approaches using CVD have highly-controlled conditions for growth to ensure orientation and length.
13	What is a method of exfoliating graphene from highly oriented pyrolytic graphite (HOPG) crystal?	HOPG is essentially highly oriented graphite which is ideal for producing graphene using mechanical cleavage with adhesive tape. HOPG can be purchased commercially and then exfoliated through existing processes such as micromechanical exfoliation tape to reult in high-quality graphene with minimal defects.
14	How to synthesize graphene using PET bottles?	Waste PET can be cleaned and then used as a precursor for pyrolysis into graphene. It usually takes a 2 step process. First, pyrolysis at 900 degrees centigrade into amorphous carbon. Next, the amorphous carbon is mixed with Boron powder and heat treated at 2400 degrees celsius for 1 hour under helium gas to form graphite. This high-temperature treatment with boron enables the carbon atoms to rearrange into the layered structure of graphite.. Yield rates are about 86%. This graphite can then be liquid-phase exfoliated to form graphene.
15	What is the purpose of PMMA in the synthesis of graphene?	PMMA can be pyrolised to decompose into carbon-rich material which can then form graphene sheets through cvd or some other pyrolytic process. PMMA can also be used as a transfer agent to protect and transfer graphene to other surfaces after growth on copper.
16	What is the purpose of plasma cleaning in preparing substrates for high-quality graphene growth?	Plasma cleaning removes contaminants, organic reside, enhances surface wettability and improves the adhesion of graphene to the substrate.
17	Could you elaborate on the role of copper foil in the synthesis of graphene films using the chemical vapor deposition method?	The copper foil usually acts as a substrate and catalyst for growing graphene layers in CVD. It provides a surface on which carbon atoms can nucleate and arrange themselves into a graphene structure.
18	What role does H2O2 play in the synthesis of graphene oxide?	It reduces excess oxidisers such as potassium permanganate in variants of the Hummer's method, thus halting oxidation and preventing uncontrolled reactions. It also can promote the formation of grpahene oxide by facilitating the unravelling of carbon chains.
19	Which method would u recommend for synthesizing monolayer graphene for producing transistors?	Micromechanical Cleavage
20	Which method would u recommend for synthesizing few layer graphene for dispersion into inks?	Pyrolysis

S2. Prompts for answer generation

Prompt for RAG models

INSTRUCTION:

You are a scientific assistant to a materials science researcher. Your task is to generate factually accurate and complete answers to user questions. Select the most relevant CONTEXT to supplement your answer but there is no need to explicitly state which context was selected. If none of the CONTEXTS are accurate or applicable, clearly state that and base your response solely on your internal knowledge. Do not go beyond what is necessary to directly answer the question.

CONTEXT:

{context}

QUESTION:

{question}

Prompt for standard models

INSTRUCTION:

Do not go beyond what is necessary to directly answer the question.

QUESTION:

{question}

S3. Prompt for answer evaluation

Prompt for LLM-as-a-judge (LLM Judge) metric

INSTRUCTION:

You will be given a model's answer and the ground truth. Your task is to provide a score by comparing the answer against the provided ground truth. The maximum score attainable is 10, and the minimum is 0. Grade each response as you would a student's work. Use your judgment to assess the overall similarity in meaning and content. A more structured way to interpret the scale is: 10 marks represents 100% similarity to the ground truth, while 0 marks indicates 0% similarity. Provide me with a score as such "score:"

Now here are the answer and ground truth

Answer: {answer}

Ground truth: {ground_truth}

S4. Answer set for RAG-Gemini

Question 1

What are the steps involved to synthesize graphene via chemical vapour deposition?

Context 1

Author: Sukang Bae

Doi:10.1038/nnano.2010.132

Method:

An 8-inch-wide tubular quartz reactor is used in the CVD system, allowing a monolayer graphene film to be synthesized on a roll of copper foil with dimensions as large as 30 inches in the diagonal direction. A temperature gradient usually exists that depends on the radial position inside the tubular reactor. In our preliminary work, this sometimes resulted in inhomogeneous growth of the graphene on the copper foils. To solve this problem, a ~7.5-inch quartz tube wrapped with a copper foil was inserted and suspended inside the 8-inch quartz tube. In this way, the radial inhomogeneity in the reaction temperature could be minimized. In the first step of synthesis, the roll of copper foil is inserted into a tubular quartz tube and then heated to 1,000 °C with flowing 8 s.c.c.m. H_2 at 90 mtorr. After reaching 1,000 °C, the sample is annealed for 30 min without changing the flow rate or pressure. The copper foils are heat-treated to increase the grain size from a few micrometres to ~100 μm, as we have found that the copper foils with larger grain size yield higher-quality graphene films. The gas mixture of CH_4 and H_2 is then flowed at 460 mtorr with rates of 24 and 8 s.c.c.m. for 30 min, respectively. Finally, the sample is rapidly cooled to room temperature (~10 °C s^{-1}) with flowing H_2 under a pressure of 90 mtorr.

Context 2

Author: Carlos Diaz-Pinto

Doi: 10.1021/nn300326x

Method:

The chemical vapour deposition (CVD) growth was carried out under ambient pressure in a 1" quartz tube furnace, using 25μm-thick copper foil (Alpha Aesar, item no. 13382). At room temperature, argon gas was used to flush the quartz tube at a rate of 2L/min for at least 15 minutes. The Ar flow was then stopped, a hydrogen flow of 200mL/min was started, and the temperature was raised to 1000°C in approximately 18 minutes. After holding at 1000°C for 20 minutes, the H_2 flow was stopped and replaced by an Ar flow of 150mL/min, maintained for another 20 minutes. Then, a methane flow of 13mL/min was introduced along with the continued Ar flow of 150mL/min for a typical graphene growth time of 10 minutes. After the growth phase, the CH_4 flow was turned off and the system was cooled to room temperature over approximately 1.5 hours, while maintaining the Ar flow at 150mL/min.

Context 3

Author: Tianru Wu

Doi: 10.1002/adfm.201201577

Method:

The graphene synthesis was done by ambient pressure chemical vapour deposition (APCVD) at a typical temperature ranging from 950–1050°C for 30–80 min. A mixture of Ar and H_2 was used as reactant gases. The H_2 flow rate was adjustable between 0–10 sccm while keeping the total flow rate at 300 sccm. Carbon supply was controlled by heating the precursor with a halogen lamp under a typical polystyrene temperature (Tp) range of 80–280°C. After the growth process, the halogen tungsten lamp was turned off, and the furnace was cooled down to room temperature naturally.

Context 4

Author: Jian Hao

Doi: 10.1038/s44286-025-00203-z

Method:

Synthesis of CVD graphene: Commercial copper foils were pretreated with nitric acid (4 wt%) for 10 min to remove the surface contamination. Cu foils were washed with deionized water four times and stored in isopropanol before use. A customized CVD furnace (Carbolite, TS1/3-1200, heating zone 1.2 m) hosting a quartz tube and an alumina tube (Zibo Highlion New Material) was built for graphene synthesis. The synthesis protocol of CVD graphene followed the previous reports. First, the Cu foil was treated at 1,020 °C for 2 h under 1 bar CO_2 flow to remove surface carbonaceous contaminations. After exchanging the gas to Ar and H_2 with a flow rate of 500 and 50 sccm, respectively, the furnace was slowly heated to 1,065 °C and the temperature was maintained for 3 h. The graphene synthesis was conducted at 1,020 °C using 9 sccm of CH_4 and 3 sccm of H_2 under a pressure of 180 mTorr for 30 min.

Context 5

Author: A. Kumar

Doi:10.1016/j.carbon.2011.11.033

Method:

Graphene film synthesis was performed in a SEKI AX5200S MPCVD (microwave plasma chemical vapor deposition) system with H_2 (50 sccm) and CH_4 (5 sccm) as the feed gases. A 25 µm thick copper foil (Alfa Aesar, 99.8% purity) was used as the substrate. The foil was cut in 2 x 2 cm^2 pieces and placed into the MPCVD chamber, which was evacuated to a base pressure of 2 Torr and then filled with high-purity hydrogen at a pressure of 10 Torr. The Cu foil was supported by a ceramic stand that elevated it from the Mo puck by about 15 mm. The elevation of the Cu foil above Mo puck was found to be critical for the growth of the films

because it ensures a strong coupling between the plasma and the Cu foil, thus enabling rapid self-heating of the foil by the microwave plasma. No additional heater was used. The thermocouple attached to the substrate table indicated a maximum reading of approximately 65°C during the process. A dual-wavelength infrared pyrometer indicated a maximum temperature of 700 ± 25 °C. However, as the copper foil is coated with graphene, the surface emissivity changes, making reliable IR measurements difficult. The entire growth is accomplished in less than 5 min and generally involves two stages. First, in a plasma cleaning and annealing step, the foil is kept in 400 W hydrogen plasma for 3 min. A H2 flow rate of 50 sccm was used with downstream pressure control to maintain 10 Torr chamber pressure throughout the process. This process leads to rapid self-heating and removal of copper oxide from the foil surface. The second stage involves introduction of CH4 (while maintaining the hydrogen plasma) at a flow of 5 sccm for a short duration (between 30 s and 2 min), providing approximately 10% CH4 concentration in hydrogen. Depending on the growth duration and whether or not annealing is used, different forms of carbon can be deposited.

RAG-Gemini

The general steps involved in synthesizing graphene via chemical vapor deposition (CVD) typically include:

1. **Substrate Preparation:** Copper foils are often pretreated, which may involve cleaning steps (e.g., with nitric acid) and/or annealing at high temperatures to increase grain size or remove surface contaminations.

2. **Chamber Setup and Purging:** The prepared copper foil is loaded into a reaction chamber (e.g., a quartz tube within a furnace). The chamber is then purged with an inert gas (like argon) or evacuated to remove ambient air and impurities.

3. **Heating and Annealing:** The substrate is heated to a high temperature, typically ranging from 950°C to 1065°C. During this heating phase, and often for a period after reaching the target temperature, the substrate is annealed under a flow of hydrogen (H_2) or a mixture of hydrogen and an inert gas. This annealing step helps to clean the copper surface (e.g., by reducing native oxides) and can promote the formation of larger copper grains, which are beneficial for high-quality graphene growth.

4. **Graphene Growth:** A carbon precursor gas (most commonly methane, CH_4) is introduced into the hot chamber, typically along with hydrogen and sometimes an inert gas. The carbon atoms from the precursor gas decompose at the high temperature and dissolve into the copper substrate, precipitating out as graphene on the surface as the copper becomes saturated or during cooling. This growth phase occurs for a specific duration at controlled flow rates and pressure.

5. **Cooling:** After the growth phase, the carbon precursor gas flow is stopped. The system is then cooled down to room temperature, often while maintaining a flow of hydrogen or an inert gas. The cooling rate can vary depending on the desired graphene properties.

Ground truth

First, a substrate, such copper, is cleaned, prepared and placed into a CVD chamber that is usually a tube made out of an inert material such as quartz. After which, the CVD chamber is evacuated into a vacuum or flooded with inert gases.The CVD chamber is then heated to some kind of critical temperature (about 1000 degrees celsius), before feedstock or precursor gases with high hydrocarbon contents are pumped into the CVD chamber. The high temperature in the chamber decomposes these feedstock gases into carbon atoms which then deposit onto the substrate's surface where they reassemble to into the characteristic graphene lattice. The feedstock gases also produce hydrogen which do not contribute to the reaction. The choice of substrate matters as it plays a role in catalysing the reaction. The temperature and duration of this deposition is controlled to either form mono-layer or few-layer graphene.

Question 2

What are the steps involved to synthesize graphene via mechanical exfoliation?

Context 1

Author: Florian R. Ong

Doi: 10.1088/0957-4484/26/40/405201

Method:

Graphene layers are then obtained by mechanical exfoliation of kish graphite onto a separate Si/SiO2 substrate, which we call the exfoliation substrate. Using Si/SiO2 as a substrate enables the discrimination of one to few layer flakes by inspection with an optical microscope. The number of layers is then confirmed by Rama spectrometry, and a monolayer is selected for the transfer step to follow. A solution of cellulose acetate butyrate (CAB) in ethyl acetate 60 mg/mL, which is a hydrophobic polymer, is dripped onto the substrate, covering the graphene monolayer of interest. The latter is still distinguishable under the optical microscope after the CAB polymer dries off. The needles of the micromanipulator are used to punch markers around the monolayer. These markers will later allow for the alignment of the graphene relative to the gate on the destination substrate, onto which graphene is optically indiscernible.

Context 2

Author: Yuan Huang

Doi: 10.1021/acsnano.5b04258

Method:

We used SiO2/Si as the substrate and ordinary adhesive tape as the transfer medium. Contact with the tape transfers thick graphite flakes from a highly oriented pyrolytic graphite (HOPG) crystal. Prior to exfoliating thin graphene from these flakes, the SiO2/Si substrate is ultrasonically cleaned in acetone, 2-propanol, and deionized (DI) water, and then subjected to oxygen plasma to remove ambient adsorbates from its surface. Following the plasma cleaning step, the graphite-loaded tape is brought in contact with the substrate. Instead of immediately removing it to complete the exfoliation, the substrate with the attached tape is annealed for 25 min at~100°C, in air on a conventional laboratory hot plate. After the sample is cooled to room temperature, the adhesive tape is removed, which completes the exfoliation.

Context 3

Author: K. S. Novoselov

Doi: 10.1126/science.1102896

Method:

The starting material was 1-mm-thick platelets of highly -oriented pyrolytic graphite (HOPG). We used commercially available HOPG of grades ZYH (www.ntmdt.ru) and HOPG-1 (www.2spi.com) with μ >100,000 cm2 /Vs at 4K. Using dry etching in oxygen plasma, we first prepared 5 μm-deep mesas on top of the platelets (mesas were squares of various sizes from 20 μm to 2 mm). The structured surface was then pressed against a 1-μm-thick layer of a fresh wet photoresist spun over a glass substrate. After baking, the mesas became attached to the photoresist layer, which allowed us to cleave them off the rest of the HOPG sample. Then, using scotch tape we started repeatedly peeling flakes of graphite off the mesas. Thin flakes left in the photoresist were released in acetone. When a Si wafer was dipped in the solution and then washed in plenty of water and propanol, some flakes became captured on the wafer's surface (as a substrate, we used n+ -doped Si with a SiO2 layer on top; in order to avoid accidental damage - especially during plasma etching - we chose to use relatively thick SiO2 with t =300nm). After this, we used ultrasound cleaning in propanol, which removed mostly thick flakes. Thin flakes (d < 10 nm) were found to attach strongly to SiO2, presumably due to van der Waals and/or capillary forces. To select from the resulting films only those that are just a few graphene layers thick, we used a combination of optical, electron-beam and atomic -force microscopy. Graphitic films thinner than 50 nm are transparent to visible light but nevertheless can easily be seen on the SiO2 surface because of the added optical path that shifts the interference colors. The color for a 300 nm wafer is violet-blue and the extra thickness due to graphitic films shifts it to blue. At thicknesses d less than ~1.5nm, as measured by AFM, graphene films are no longer visible even via the interference shift as it becomes too small. This provides a natural marker that we use to distinguish between two groups of films that we refer to as few- and multi- layer graphene. The graphene films whose properties are reported in the main paper were selected as those that were completely invisible in an optical microscope (OM). Although invisible in optics, few-layer graphene (FLG) can still be seen clearly in a high-resolution SEM (we used FEI Serion). To identify FLG films, we have compared optical and scanning-electron micrographs of large areas on the wafer, trying to find the films visible in SEM but not in OM.

Context 4

Author: Dan Li

Doi: 10.1038/nnano.2007.451

Method:

Graphite oxide was synthesized from natural graphite (SP-1, Bay Carbon) by a modified Hummers method as originally presented by Kovtyukhova and colleagues. As-synthesized graphite oxide was suspended in water to give a brown dispersion, which was subjected to dialysis to completely remove residual salts and acids. Ultrapure Milli-Q® water was used in all experiments. As-purified graphite oxide suspensions were then dispersed in water to create a 0.05 wt% dispersion. Exfoliation of graphite oxide to graphene oxide (GO) was achieved by ultrasonication of the dispersion using a Brandson Digital Sonifier (S450D, 500 W, 30% amplitude) for 30 min. The obtained brown dispersion was then subjected to 30 min of centrifugation at 3,000 r.p.m. to remove any unexfoliated graphite oxide (usually present in a very small amount) using an Eppendorf 5702 centrifuge with a rotor radius of 14 cm. In a

typical procedure for chemical conversion of graphite oxide to graphene, the resulting homogeneous dispersion (5.0 ml) was mixed with 5.0 ml of water, 5.0 μl of hydrazine solution (35 wt% in water, Aldrich) and 35.0 μl of ammonia solution (28 wt% in water, Crown Scientific) in a 20-ml glass vial. The weight ratio of hydrazine to GO was about 7:10. After being vigorously shaken or stirred for a few minutes, the vial was put in a water bath (~95 °C) for 1 h. Unless specifically stated, graphene dispersions prepared according to the above procedure were used for further characterization and film fabrication in this work. The graphene paper was made by filtration of a dispersion through an Anodisc membrane filter (47 mm in diameter, 0.2 μm pore size; Whatman), similar to the method reported for making GO paper. The graphene paper was cut by a razor blade into rectangular strips of approximately 4 mm × 15 mm for mechanical testing.

Context 5

Author: Tim J. Booth

Doi: 10.1021/nl801412y

Method:

Graphene crystals are first prepared by standard micromechanical cleavage techniques . Sufficiently large flakes produced in this way are widely distributed over a substrate (occurring with a typical number density of < 1 per cm2) and in a great minority as compared to thicker flakes. This prevents their identification via atomic-resolution techniques such as scanning probe or electron microscopies either due to prohibitively small search areas or a lack of response specific to single-layer graphene. Fortunately, one-atom thick crystals can still be identified on surfaces covered with thin dielectric films due to a color shift induced by graphene, which allows crystals to be found rapidly with a trained eye and a quality optical microscope. In the current work, we have used Si wafers that, in contrast to the standard approach, are not oxidized but instead covered with a 90 nm thick film of polymethyl methacrylate (PMMA). The optical properties of PMMA are close to those of SiO2, and the visible contrast of graphene is optimal at this particular thickness. The PMMA film also serves later as a sacrificial layer during the final liftoff. Once a suitable graphene crystal is identified in an optical microscope, we employ photolithography to produce a chosen pattern (in our case, a Transmission Electron Microscopy (TEM) grid) on top of graphene (we usually used a double-layer resist consisting of 200 nm polymethyl glutarimide (PMGI) and 200 nm S1805. A 100 nm Au film with a 5 nm Cr adhesion layer is thermally evaporated after developing the resist. Liftoff of the metal film is not performed in acetone, which would destroy the base layer, but in a 2.45 wt % Tetramethylammonium hydroxide (TMAH) solution (MF-319 developer; MicroChem) at 70°C, resulting in a minimal etch rate for PMMA (< 5° Åmin-1). The next step involves another round of photolithography, in which the graphene crystal is remasked with the same photoresist. The mask serves here to protect graphene during electrodeposition, when a thick copper film is electrochemically grown on top of the Au film, repeating the designed pattern. We have chosen a CuSO4/H2SO4 electrolyte because of its low toxicity, resist and substrate compatibility and ease of deposition. Finally, acetone is used to strip the remaining resist, releasing the copper TEM grid with the attached graphene membrane. The sample is dried in a critical point dryer to prevent the membrane rupturing due to surface tension.

RAG-Gemini

To synthesize graphene via mechanical exfoliation, the general steps involved are:

1. **Preparation of Graphite Source**: Begin with a bulk graphite material, such as highly oriented pyrolytic graphite (HOPG) or kish graphite. In some methods, the graphite surface may be pre-patterned, for example, by creating mesas using dry etching in oxygen plasma.

2. **Exfoliation**: An adhesive material, commonly ordinary adhesive tape or scotch tape, is pressed against the graphite surface. Alternatively, a photoresist layer can be used. Upon removal, the adhesive peels off layers of graphite. This peeling process may be repeated multiple times to obtain thinner flakes.

3. **Substrate Preparation (Optional but Common)**: The destination substrate, typically a silicon wafer with a thin silicon dioxide (Si/SiO2) layer, is often pre-cleaned. This can involve ultrasonic cleaning in solvents like acetone and isopropanol, followed by deionized water, and sometimes oxygen plasma treatment to remove ambient adsorbates from its surface.

4. **Transfer to Substrate**: The exfoliated graphite flakes, often still attached to the adhesive tape or released into a solvent (e.g., acetone) from an intermediate layer (like photoresist or a polymer film), are then transferred onto the prepared substrate. This may involve direct contact of the tape with the substrate, or dipping the substrate into a solution containing the flakes. In some variations, a polymer solution (e.g., cellulose acetate butyrate or CAB) is applied over the graphene on an exfoliation substrate to facilitate its transfer to a new substrate.

5. **Post-Transfer Processing (Optional)**: After the adhesive tape or polymer is in contact with the substrate, some methods involve annealing the substrate with the attached material at an elevated temperature (e.g., ~100°C) before cooling and final removal of the adhesive.

6. **Identification and Selection**: Once transferred, the flakes on the Si/SiO2 substrate are inspected to identify graphene of desired thickness (e.g., monolayer or few-layer). This is commonly done using an optical microscope, as the interference effect of the Si/SiO2 layer causes visible contrast variations depending on flake thickness. The number of layers can be further confirmed using techniques such as Raman spectrometry, atomic force microscopy (AFM), or scanning electron microscopy (SEM).

Ground truth

Mechanical exfoliation of graphene, also known as the sticky tape method, involves first taking a piece of graphite, and then using a piece of sticky tape release a small layer of graphite onto it. This is done simply by placing the sticky tape repeatedly on the piece of graphite. To produce single atomic sheets of graphene, this tape is folded in half and repeatedly pulled apart, thereby shearing layers of graphene from the already deposited material until only a single-atomic layer is present.

Question 3

What are the steps involved to synthesize graphene via plasma enhanced chemical vapour deposition (PECVD)?

Context 1

Author: Yong Seung Kim

Doi: 10.1039/C2NR33034B

Method:

The graphene films studied in this work were grown on 25 μm-thick Cu foils by plasma-enhanced chemical vapor deposition (PECVD). The chamber is equipped with an inductively coupled plasma reactor. The radiofrequency (RF) coil, having a diameter of ~25 cm, is located 35 cm above the top surface of the graphite substrate holder. In this geometry, the heating effect of the substrate by plasma was negligible. A residual gas analyzer (RGA 100, Stanford Research System) is attached at the side wall of the chamber to investigate the density of discharged species. A differential pumping technique is employed to meet the required working pressure of the RGA. The system is pumped with a turbomolecular pump (Osaka Vacuum LTD, TG1003), keeping the base pressure as low as ~10^-7 Torr. Polycrystalline Cu foil was cut into 7 x 7 cm2 pieces and mounted in the chamber without any pre-cleaning treatment. Five different stages were employed to synthesize graphene films on Cu foil using methane (CH4) as the carbon source. The Cu substrate was heated to the growth temperature (700–830 °C) at a heating rate of 3 °C/s. When it reached the target temperature, H2 gas was introduced into the chamber at a flow rate of 40 standard cubic centimeters per minute (sccm). Hydrogen gas was discharged by an RF power of 50 W for 2 minutes to eliminate surface oxides on the copper foil. Then, the chamber was purged with Ar at a flow rate of 100 sccm for 2 minutes to remove residual hydrogen gas. During the graphene growth stage, radiofrequency (RF) plasma was generated for a specified growth time under a continuous flow of argon (or hydrogen, 40 sccm) and methane (1 sccm), while the pressure was kept at 10 mTorr. The plasma power was varied from 10 to 200 W and the growth time from 0.2 to 4 minutes. Subsequently, the sample was cooled down rapidly to room temperature at a cooling rate of 3 °C/s by turning off the heating power, and then it was taken out for characterization.

Context 2

Author: A. Kumar

Doi: 10.1016/j.carbon.2011.11.033

Method:

Graphene film synthesis was performed in a SEKI AX5200S MPCVD (microwave plasma chemical vapor deposition) system with H2 (50 sccm) and CH4 (5 sccm) as the feed gases. A 25 μm thick copper foil (Alfa Aesar, 99.8% purity) was used as the substrate. The foil was cut in 2 x 2 cm2 pieces and placed into the MPCVD chamber, which was evacuated to a base pressure of 2 Torr and then filled with high-purity hydrogen at a pressure of 10 Torr. The Cu

foil was supported by a ceramic stand that elevated it from the Mo puck by about 15 mm. The elevation of the Cu foil above Mo puck was found to be critical for the growth of the films because it ensures a strong coupling between the plasma and the Cu foil, thus enabling rapid self-heating of the foil by the microwave plasma. No additional heater was used. The thermocouple attached to the substrate table indicated a maximum reading of approximately 65°C during the process. A dual-wavelength infrared pyrometer indicated a maximum temperature of 700 ± 25 °C. However, as the copper foil is coated with graphene, the surface emissivity changes, making reliable IR measurements difficult. The entire growth is accomplished in less than 5 min and generally involves two stages. First, in a plasma cleaning and annealing step, the foil is kept in 400 W hydrogen plasma for 3 min. A H2 flow rate of 50 sccm was used with downstream pressure control to maintain 10 Torr chamber pressure throughout the process. This process leads to rapid self-heating and removal of copper oxide from the foil surface. The second stage involves introduction of CH4 (while maintaining the hydrogen plasma) at a flow of 5 sccm for a short duration (between 30 s and 2 min), providing approximately 10% CH4 concentration in hydrogen. Depending on the growth duration and whether or not annealing is used, different forms of carbon can be deposited.

Context 3

Author: Lam Van Nang

Doi: 10.1149/2.082204jes

Method:

To investigate the rf plasma effect, graphene growth was carried out with a diluted CH4 gas, in which graphene was not formed without applying plasma. The CH4 gas of 1 SCCM (denoting cubic centimeter per minute at STP) and the mixed gases of 10% H2 and 90% Ar (forming gas) of 100 SCCM were introduced in a hot-wall tubular reaction chamber. The Cu foil (Alfa Aesar) was pre-treated with an acetic acid solution at 60°C for 10 min, followed by washing with de-ionized water. The foil was heated to a graphene synthesis temperature (950°C) and annealed for 10 min with forming gas flow (100 SCCM) to remove native oxides from the Cu foil and to enlarge its grains. The synthesis pressure was fixed at 1 Torr. The synthesis time and rf plasma power is 5min and 200 W, respectively. The synthesized graphene on Cu was transferred on SiO2/Si or glass substrate for various characterizations. First, a 500 nm layer of poly-methyl methacrylate (PMMA) was spin coated on the surface of the graphene film to serve as support. The graphene layer at the back side of the Cu foil was then etched off using oxygen plasma. After the etching of the Cu foil with FeCl3 aqueous solution at 40°C, the PMMA/graphene was transferred to a target substrate. The graphene on the SiO2/Si or glass substrate was achieved by dissolving the PMMA film using acetone

Context 4

Author: Alexander Malesevic

Doi: 10.1088/0957-4484/19/30/305604

Method:

Few-layer graphene (FLG) was synthesized in a Microwave plasma-enhanced chemical vapour deposition (MW PECVD) set-up composed of an Iplas Cyrannus MW plasma source mounted on top of a stainless-steel vessel. The plasma source consists of a 6 kW, 2.45 GHz cylindrical MW resonator, which couples the MW power through five annular slots in a quartz tube and enables operation either in the TM012 or in the surface wave plasma mode. For all the experiments, the plasma was operated in the TM012 mode. A flexible substrate holder allows free positioning of substrates along the axis of the quartz tube. A broad range of materials was used as substrates, including quartz, silicon, nickel, platinum, germanium, titanium, tungsten, stainless steel, tantalum and molybdenum. Prior to growth, the substrates are heated for 20 min in a 2 kW MW plasma at 40 Torr, using a H_2 flow of 200 sccm. This plasma is sufficiently powerful to heat the samples from room temperature up to 700°C, as measured by a thermocouple embedded in the substrate holder. Immediately after the heating step, CH_4 mixed with H_2 is introduced into the growth chamber with a CH_4/H_2 ratio of 1/8 at a total flow rate of 200 sccm. Keeping the plasma power and pressure at 2 kW and 40 Torr, respectively, FLG was grown with this gas mixture for various growth times ranging from 1 to 3000 s.

Context 5

Author: D.A. Boyd

Doi: 10.1038/ncomms7620

Method:

The copper substrates were placed on a quartz flat inside of quartz tube. A typical substrate size was (8×13) mm2. The tube was evacuated to 25 to 30 mTorr. A 2-5 sccm flow of room temperature hydrogen gas with 0.4% methane and a comparable amount of nitrogen gas was added and the pressure was controlled at 500 mTorr. The addition of methane to the gas flow was controlled by a precision leak valve, and a typical concentration, as measured by gas chromatography, was 0.4 %. The initially low vacuum conditions (~ 25 mTorr) provided sufficient partial pressures of nitrogen (atmospheric) for the plasma enhanced chemical vapour deposition (PECVD) process. (We note that a leak valve for including purified nitrogen was added to the setup to study the effects of increased nitrogen partial pressures.) Typical partial pressures measured by residual gas analysis (RGA) are as follows: N_2 (Mass 28): 6.9 × 10^-8 Torr, CH_4 (Mass 16): 1.5 × 10^-7 Torr , O_2 (Mass 32): 5.4 × 10^-9 Torr , H_2O (Mass 18): 1.2 × 10^-6 Torr, CO_2 (Mass 44): 2.3 × 10^-8 Torr. From these values we estimate that the concentration of nitrogen in the gas flow is typically on the same order as that of methane. The PECVD process was found to be highly sensitive to the relative amounts of methane and nitrogen. Excessive methane mixtures resulted in no etching, while excessive nitrogen mixtures would result in excessive etching of the copper. Slight adjustments to the methane concentration could be made via either optical emission spectroscopy (OES) or RGA. A low-power (ranging from 10 to 40 W) cold hydrogen plasma was formed over the copper substrate using an Evenson cavity. Exposure of copper substrates to the plasma enabled continuing etching and cleansing of the copper surface during the graphene growth process. The gas temperature inside the plasma was measured using a thermocouple sheathed in boron nitride. The thermocouple was placed in the plasma above the sample and could be translated along the tube. The peak gas temperature measured in plasma treatment was 160°C at 10 W and 425°C at 40 W, and the gas temperature profile decreased rapidly (by 120°C at 10 W and 425°C at 40 W within 1 cm) from the peak value. The maximum

temperature of the copper substrate (Ts) was measured using the melting point of known solids, lead and zinc, and found to be within the range of 327.5°C < Ts < 419.5°C at 40 W. Typically after 5 to 20 minutes of direct exposure to the plasma, a large-area monolayer graphene formed on the backside of copper substrates while the front side of the substrates was coated with disordered graphite. Upon the completion of graphene growth, the plasma was extinguished, and the gas flows were stopped. The process tube was then evacuated and back filled with Ar, and the substrate with graphene coating was subsequently removed. Copper deposition was visible on the inside of the tube and on the sample holder as the result of plasma etching of the copper substrates.

RAG-Gemini

The synthesis of graphene via plasma-enhanced chemical vapor deposition (PECVD) typically involves the following steps:

1. **Substrate Preparation and Loading**: A copper foil substrate (often cut to specific dimensions, e.g., 2x2 cm^2 or 7x7 cm^2) is placed into the PECVD reaction chamber. Pre-cleaning of the copper foil (e.g., with acetic acid) may be performed prior to loading, though some methods omit this step.

2. **Chamber Evacuation**: The reaction chamber is evacuated to a low base pressure, typically ranging from approximately 10^{-7} Torr to a few Torr, using a vacuum pump system.

3. **Substrate Heating and Pre-treatment**:

* The substrate is heated to the target growth temperature, which can range from approximately 327°C to 950°C. Heating can be achieved by an external heater or by self-heating due to interaction with the plasma.

* A plasma cleaning step is usually performed. This commonly involves introducing hydrogen gas (e.g., 40-200 sccm) and generating a hydrogen plasma (e.g., 50-400 W, for 2-20 minutes). This process helps to remove native oxides from the copper surface and/or enlarge copper grains.

* In some methods, an inert gas like argon may be introduced to purge residual gases after the plasma cleaning.

4. **Graphene Growth**:

* A carbon source gas, most commonly methane (CH_4, e.g., 1-5 sccm), is introduced into the chamber. This is often mixed with other gases such as hydrogen, argon, or nitrogen, depending on the specific method.

* A radiofrequency (RF) or microwave (MW) plasma is generated, typically with power ranging from 10 W up to several kilowatts, at a controlled chamber pressure (e.g., 10 mTorr to 40 Torr).

* The plasma facilitates the decomposition of the precursor gases and the deposition of graphene onto the substrate. This growth stage is maintained for a specified duration, typically ranging from 0.2 minutes to 20 minutes.

5. **Cool-down**: After the growth period, the plasma is extinguished, and the heating power is turned off. The sample is then cooled down to room temperature, sometimes rapidly.

6. **Sample Retrieval**: Once cooled, the synthesized graphene film on the copper substrate is removed from the chamber for further characterization or processing.

Ground truth

The plasma-enhanced process is a modification of traditional chemical vapour deposition. As with traditional CVD, a prepared substrate (cleaned and heated to about 1000 degrees celsius) is inserted into a a CVD chamber made out of an inert material and gaseous precursors such as methane are introduced into the reaction chamber together with other inert gases such as H2 or Ar. Often plasma-enhanced CVD can use lower temperatures than traditional CVD. A plasma, typically generated by radio frequency energy at 13.56Mhz is used to excite and disassociate the precursor gases. This process creates highly reactive carbon radicals which deposit and self-assemble ont he heated substreate forming graphene layers. The growth can be tailored by adjusting parameters such as gas flow rates, plasma power and time.

Question 4

What are the steps involved to synthesize graphene via electrochemical exfoliation?

Context 1

Author: Khaled Parvez

Doi: 10.1021/ja5017156

Method:

Electrochemical exfoliation of graphite: Natural graphite flakes were used as a carbon electrode (i.e., anode) for electrochemical exfoliation of graphite. The graphite flakes adhered to a conductive carbon tape, forming a pellet. A Pt wire was used as a cathode. The electrolyte for the exfoliation was prepared by dissolving 1.06 g (NH4)2SO4 in 80 mL H2O (i.e., 0.1 M). The distance between the graphite and the Pt electrode was ~2 cm and was kept constant throughout the electrochemical process. Electrochemical exfoliation was carried out by applying positive voltage (10 V) to the graphite electrode. After the graphite exfoliation was completed, the product was collected through a PTFE membrane filter with 0.2 m pore size and washed several times with deionised water by vacuum filtration. The resultant exfoliated graphene (EG) was then dispersed in DMF by sonication at low power for 10 min. The dispersion was maintained for 48 h to precipitate unexfoliated graphite flakes or particles. The top part of the dispersion was used for characterisation and device fabrication. For largescale production of EG, graphite foil (Alfa Aesar) was used instead of flakes.

Context 2

Author: Khaled Parvez

Doi: 10.1021/nn400576v

Method:

Electrochemical exfoliation process: Natural graphite flakes (Sigma Aldrich) were used as carbon source (working electrode) for electrochemical exfoliation. The graphite flakes were adhered on a conductive carbon tape to form a pellet and then immersed into the H2SO4 solution (Sigma Aldrich; 95-97%). A Pt wire was used as a counter electrode. The electrochemical exfoliation of graphite was carried out by applying positive voltage (+10 V) on the working electrode. The exfoliated graphene (EG) was then collected with a polytetrafluoroethylene (PTFE) membrane filter (pore size 0.2 μm) and washed repeatedly with DI water by vacuum filtration. The resultant EG was dispersed in N,N'-dimethylformamide (DMF) by sonication at low power for 10 min. The dispersion was kept for 24 h for the precipitation of unexfoliated graphite flakes and/or particles. The top part of the dispersion was used for further characterization and device fabrication.

Context 3

Author: Guoxiu Wang

Doi: 10.1016/j.carbon.2009.07.040

Method:

High purity graphite rods (6 mm, 99.999%, Aldrich) were used as electrodes. Poly(sodium-4-styrenesulfonate) (PSS, Mw = 70,000, Aldrich) was dissolved in de-ionized (DI) water to form the electrolyte (0.001 M). In a typical synthesis, two graphite rods were placed in an electrolysis cell filled with the electrolyte. A constant potential of 5 V (DC voltage) was applied to the two electrodes (CHI 660 C Electrochemical Workstation). After 20 min electrolysis, black product gradually appeared at the positive electrode (anode). The exfoliation continued for 4 h. Then the product (a dispersion) was taken from the electrolysis cell. The dispersion was centrifuged at low speed (1000 rpm) to remove large agglomerates. The top of the dispersion was then decanted. This graphene–PSS suspension is very stable in nature. After 6 months storage, there is no any precipitation. To obtain dry graphene powders, the dispersion was washed with DI water and ethanol, and then dried in a vacuum oven at 80°C. The yield of graphene was estimated by weighting the dried graphene powders and the dried sediment. Our electrolytic exfoliation method results in producing graphene at a yield at about 15 wt.%.

Context 4

Author: Sheng Yang

Doi: 10.1021/jacs.5b09000

Method:

Graphite exfoliation was conducted in a home-built setup that used rolled graphite foils as working electrodes and platinum foils as counter electrodes. An aqueous solution containing ammonium sulphate with (2,2,6,6-tetramethylpiperidin-1-yl)oxyl (TEMPO) was tested as an electrolyte. Before exfoliation, the graphite foil was pretreated by alternately soaking it in liquid nitrogen and absolute ethanol. During this process, violent nitrogen evolution and ethanol solidification promoted the expansion of the graphite foils. The dilation was extremely rapid (within 10 s) and yielded a drastic volume increase at the graphite boundary (~10 times), which facilitated the expansion of the graphite layers, as did the following anion intercalation steps. When the graphite anodes were immersed in the electrolyte, by applying a static bias of 10 V, they detached into small pieces in less than 10 s, spreading on top of the electrolyte and/or on the bottom of the electrochemical cell. After filtration and copious washing, these exfoliated graphene sheets were collected and then dispersed in DMF via mild sonication. A stable dispersion with a concentration of ~0.3 mg/mL was obtained that was stable for 2 weeks without apparent aggregation.

Context 5

Author: Qingbin Zheng

Doi: 10.1021/nn2018683

Method:

Five grams of natural graphite flakes (Asbury Graphite Mills) and 150 mL of sulfuric acid (H2SO4, General Chemical) were mixed and stirred in a round-bottom flask at a speed of 200 rpm. Fifty milliliters of fuming nitric acid was added into the mixture. The mixture was kept at room temperature and stirred for 24 h. Two-hundred milliliters of deionized water was then poured slowly into the mixture. The resultant mixture was washed using DI water, followed by centrifugation and drying at 60 °C for 24 h to obtain graphite intercalation compound. The dry graphite intercalation compound powder was thermally expanded at 1050 °C for 15 s to obtain expanded graphite that was used for the production of graphene oxide (GO). One gram of expanded graphite and 200 mL of sulfuric acid were mixed and stirred in a three-neck flask. Ten grams of KMnO4 was added to the mixture while stirring. The solution was transferred into an ice bath, and 200 mL of deionized water and 50 mL of H2O2 were poured slowly into the mixture, realizing color change of the suspension to light brown. The GO particles were washed and centrifuged with a HCl solution three times, then centrifuged again and washed with deionized water until the pH of the solution became about 5 to 6. The GO particles obtained thereby were diluted using DI water (~1 mg/mL) and dispersed by gentle shaking.

RAG-Gemini

The synthesis of graphene via electrochemical exfoliation generally involves the following steps:

1. **Electrode and Electrolyte Setup**: Graphite (flakes, rods, or foils) is used as the working electrode (anode), and a counter electrode (e.g., platinum wire, platinum foil, or another graphite rod) is set up. These electrodes are immersed in an electrolyte solution. Common electrolytes include ammonium sulfate ((NH4)2SO4) in water, sulfuric acid (H2SO4), or poly(sodium-4-styrenesulfonate) (PSS) in de-ionized water.

2. **Electrochemical Exfoliation**: A positive voltage (typically 5-10 V) is applied to the graphite working electrode. This causes the graphite layers to exfoliate, leading to the detachment of graphene sheets into the electrolyte, often forming a dispersion.

3. **Collection and Purification**: The exfoliated product is collected, usually by filtration (e.g., using a PTFE membrane filter). The collected material is then washed multiple times with deionized water to remove residual electrolyte and impurities, often via vacuum filtration.

4. **Separation of Unexfoliated Material (Optional)**: To obtain a purer graphene dispersion, the product may be subjected to centrifugation at a low speed to remove larger unexfoliated graphite flakes or agglomerates.

5. **Dispersion**: The resultant exfoliated graphene is often dispersed in a solvent, such as N,N'-dimethylformamide (DMF), typically assisted by mild sonication for a short period (e.g., 10 minutes). The dispersion may then be allowed to stand for a period (e.g., 24-48 hours) to allow any remaining unexfoliated graphite to settle, with the top part of the dispersion being used for further applications.

6. **Drying (Optional)**: To obtain dry graphene powders, the dispersion can be washed and then dried in a vacuum oven.

Ground truth

First, an electrochemical set up is made involving a graphite working electrode, a counter electrode, a referene electrode and electrolyte and a power supply. All electrodes are inserted into the liquid electrolyte. Using the power supply, either a positive or negative potential is applied to the graphite electrode which either attracts anions (positive potential) or cations (negative potential) from the graphite electrode which cases expansion. The graphene can be subsequently exfoliated by mixing in the precursor.

Question 5

What are the steps involved to synthesize graphene via arc discharge?

Context 1

Author: Zhiyong Wang

Doi: 10.1088/0957-4484/21/17/175602

Method:

The electric arc oven for synthesis of graphene mainly comprises two electrodes and a steel chamber cooled by water. The cathode and anode were graphite rods (purity: 99.99%) with diameters of 8 and 6 mm, respectively. The current in the discharge process was maintained at 100 A. Initial air pressures of 1000 Torr was employed . The pressure in the oven was increased to a certain level during the discharge process because of the heating effect. A 10 cm long anode graphite rod was consumed in a period of ~10 min. After the consumption of the anode, flue products deposited on the inner wall of the chamber were collected. Several tens of grams of products were obtained per day. Heat treatment in air was performed to purify the products. The sample was heated in air from room temperature to 600°C for 2h and then was cooled down to room temperature.

Context 2

Author: Tianru Wu

Doi: 10.1002/adfm.201201577

Method:

The graphene synthesis was done by ambient pressure chemical vapour deposition (APCVD) at a typical temperature ranging from 950–1050°C for 30–80 min. A mixture of Ar and H_2 was used as reactant gases. The H_2 flow rate was adjustable between 0–10 sccm while keeping the total flow rate at 300 sccm. Carbon supply was controlled by heating the precursor with a halogen lamp under a typical polystyrene temperature (Tp) range of 80–280°C. After the growth process, the halogen tungsten lamp was turned off, and the furnace was cooled down to room temperature naturally.

Context 3

Author: Maxim G. Rybin

Doi: 10.1016/j.cartre.2024.100349

Method:

All experiments were done using the original equipment designed in RUSGRAPHENE company specially for chemical vapour deposition (CVD) synthesis of graphene films. The chamber walls are cooled by water. The main differences of the equipment from the commercial analogs (such as a CVD furnace quartz tube or cold-wall CVD reactors) are the following: 1) 25 μm thickness copper foil with 30 × 110 mm size is heated resistively with the electrical current increasing up to 85 A and voltage around 3 V for heating up to 1050 °C; 2) the temperature during the synthesis is measured through the optical window in the chamber by the double wavelength infrared pyrometer (Mikron M770) directly from the copper substrate. The setup provides an opportunity to control all of the synthesis parameters such as the heating and cooling rates, the temperature of substrate during the experiment, the gas pressure in the chamber and the gases concentrations. As the experimental setup differs from the commercial analogs, the description of synthesis process is needed. The process of graphene synthesis goes in two steps: the preparation of copper foil and the formation of graphene film. At the first stage the preparation of copper substrate means its annealing. The foil was treated by 3 M nitric acid solution before synthesis to etch contamination from the copper surface for 20 s and then the foil with the size of 30 × 110 mm and the thickness of 25 μm is held between the electrodes in the chamber. In our experiment, we do not use gas flows and do not use mass flow controllers. To fill the chamber with the necessary gases, we use manual needle valves and, by controlling the partial pressure in the chamber, set the desired concentration of gases. First, we pump the chamber to vacuum with an oil rotary pump and shut off the pump. Next, we fill the chamber with argon up to 80 millibars and add hydrogen plus 20 millibars. Thus, for the synthesis of graphene, a mixture of gases argon and hydrogen in a ratio of 4:1 under the pressure values of 100 millibars is used. Then the substrate heating by an electrical current passing is started. A linear current growth is monitored by the software. When the substrate temperature reaches the required value (1050 °C), the current increase is stopped and the temperature is held during 1 min. After annealing the copper foil, methane is injected into the chamber in a small amount, also using a manual needle valve. The injection of methane for providing a desired concentration (from 0.36 % to 4.5 %) is started after annealing. For example, at the pressure of 100 millibars, to inject 0.36 % methane, it is necessary to increase the pressure by 0.36 millibars (up to 100.36 millibars). This is achieved by opening the needle valve for 1 s. For a 4.5 % injection (or 4.5 millibars), the needle valve opens slightly longer (for 10 s). The time of graphene formation is adjusted from 30 s to 10 min. Finally, the substrate is cooled down to the room temperature during 10 s. Graphene samples synthesized on copper foils could be easily transferred onto an arbitrary substrate using PMMA (Poly- (methyl methacrylate)) as a supporting polymer substrate and ammonium persulfate as an etchant. The graphene layer deposited on copper is covered by polymer. Then the metal substrate is etched in the solution (1 g of ammonia persulfate in 5 ml of H2O). Finally, the polymer with graphene is stacked to the desired substrate and PMMA is dissolved in acetone.

Context 4

Author: A. Kumar

Doi: 10.1016/j.carbon.2011.11.033

Method:

Graphene film synthesis was performed in a SEKI AX5200S MPCVD (microwave plasma chemical vapor deposition) system with H_2 (50 sccm) and CH_4 (5 sccm) as the feed gases. A 25 μm thick copper foil (Alfa Aesar, 99.8% purity) was used as the substrate. The foil was cut in 2 x 2 cm^2 pieces and placed into the MPCVD chamber, which was evacuated to a base pressure of 2 Torr and then filled with high-purity hydrogen at a pressure of 10 Torr. The Cu foil was supported by a ceramic stand that elevated it from the Mo puck by about 15 mm. The elevation of the Cu foil above Mo puck was found to be critical for the growth of the films because it ensures a strong coupling between the plasma and the Cu foil, thus enabling rapid self-heating of the foil by the microwave plasma. No additional heater was used. The thermocouple attached to the substrate table indicated a maximum reading of approximately 65°C during the process. A dual-wavelength infrared pyrometer indicated a maximum temperature of 700 ± 25 °C. However, as the copper foil is coated with graphene, the surface emissivity changes, making reliable IR measurements difficult. The entire growth is accomplished in less than 5 min and generally involves two stages. First, in a plasma cleaning and annealing step, the foil is kept in 400 W hydrogen plasma for 3 min. A H_2 flow rate of 50 sccm was used with downstream pressure control to maintain 10 Torr chamber pressure throughout the process. This process leads to rapid self-heating and removal of copper oxide from the foil surface. The second stage involves introduction of CH_4 (while maintaining the hydrogen plasma) at a flow of 5 sccm for a short duration (between 30 s and 2 min), providing approximately 10% CH_4 concentration in hydrogen. Depending on the growth duration and whether or not annealing is used, different forms of carbon can be deposited.

Context 5

Author: Sukang Bae

Doi: 10.1038/nnano.2010.132

Method:

An 8-inch-wide tubular quartz reactor is used in the CVD system, allowing a monolayer graphene film to be synthesized on a roll of copper foil with dimensions as large as 30 inches in the diagonal direction. A temperature gradient usually exists that depends on the radial position inside the tubular reactor. In our preliminary work, this sometimes resulted in inhomogeneous growth of the graphene on the copper foils. To solve this problem, a ~7.5-inch quartz tube wrapped with a copper foil was inserted and suspended inside the 8-inch quartz tube. In this way, the radial inhomogeneity in the reaction temperature could be minimized. In the first step of synthesis, the roll of copper foil is inserted into a tubular quartz tube and then heated to 1,000 °C with flowing 8 s.c.c.m. H_2 at 90 mtorr. After reaching 1,000 °C, the sample is annealed for 30 min without changing the flow rate or pressure. The copper foils are heat-treated to increase the grain size from a few micrometres to ~100 μm, as we have found that the copper foils with larger grain size yield higher-quality graphene films. The gas mixture of CH_4 and H_2 is then flowed at 460 mtorr with rates of 24 and 8 s.c.c.m. for 30 min, respectively. Finally, the sample is rapidly cooled to room temperature (~10 °C s^{-1}) with flowing H_2 under a pressure of 90 mtorr.

RAG-Gemini

To synthesize graphene via arc discharge, the following steps are involved:

1. **Setup**: An electric arc oven is used, consisting of a steel chamber cooled by water, equipped with graphite rod electrodes (an 8 mm diameter cathode and a 6 mm diameter anode, both 99.99% purity graphite).

2. **Discharge**: The current in the discharge process is maintained at 100 A, with an initial air pressure of 1000 Torr. During the discharge, the anode graphite rod (10 cm long) is consumed over approximately 10 minutes, and the pressure inside the oven increases due to heating.

3. **Collection**: After the anode is consumed, the flue products deposited on the inner wall of the chamber are collected.

4. **Purification**: The collected products undergo heat treatment in air, where they are heated from room temperature to 600°C for 2 hours, and then cooled back to room temperature.

Ground truth

Graphene synthesis using arc discharge usises an electric arc to vaporise graphite,followed by the subsequest depostion of the graphene atoms and the formation of graphene flakes. You first need an arc reactor, with two water-cooled electrodes. The anode is usually filled with graphite which the cathode is a graphite rod. An electric arc is then established between the electrodes, using a high current (200A and under a specific pressure (e.g 500 Torr). This high temperature of the arc vaporises the graphite at the anode. The vaporised carbon atoms travel through the arc plasma and deposit on the chamber walls as graphene flakes.

Question 6

What are the steps involved to synthesize graphene via pyrolysis?

Context 1

Author: Emanuele Pompei

Doi: 10.1039/D4NR01986E

Method:

The porousified SiC wafers are cut into pieces (2×8 mm2) by a wafer saw. Pieces are individually transferred to an ultra-high vacuum (UHV) chamber where they are degassed overnight at 1000 K. After the degassing, samples are cooled to room temperature (RT) and heated again to 1650 K to allow the graphene formation on the surface of the pores. After 150 s of annealing, the sample is let to cool down to room temperature. It was observed that the temperature reached by the sample during the annealing depends on the quality of the mechanical contact between the sample and the sample holder. Therefore, by clamping one side of the sample more firmly than the other, we obtained the non-homogeneous graphenized sample discussed in the Functionalization with gold nanoparticles section.

Context 2

Author: Zhen-Yu Juang

Doi: 10.1016/j.carbon.2010.05.001

Method:

Single Layer Graphene and Few Layer Graphene were synthesized using Ni foil (30 μm in thickness, The Nilaco Corp.) as the substrate. After the Ni substrates were loaded into a quartz tubular furnace, the samples were heated to the process temperature of 900°C and maintained for 10 min under a H_2/Ar (10 and 400 sccm, respectively) atmosphere. A CH_4/H_2/Ar gas mixture (10, 10 and 400 sccm, respectively) was then introduced into the quartz tube for 10 min, and the furnace was cooled to room temperature with fast (3 °C/s) and slow (0.3°C/s) cooling rates. The gas mixture during cooling is the same as heating stage. The pressure was maintained at 750 Torr during whole process using advanced pressure control system (APC, MKS Instruments)

Context 3

Author: Tianru Wu

Doi: 10.1002/adfm.201201577

Method:

The graphene synthesis was done by ambient pressure chemical vapour deposition (APCVD) at a typical temperature ranging from 950–1050°C for 30–80 min. A mixture of Ar and H_2 was used as reactant gases. The H_2 flow rate was adjustable between 0–10 sccm while keeping the total flow rate at 300 sccm. Carbon supply was controlled by heating the precursor with a halogen lamp under a typical polystyrene temperature (Tp) range of 80–280°C. After the growth process, the halogen tungsten lamp was turned off, and the furnace was cooled down to room temperature naturally.

Context 4

Author: Hong-Kyu Seo

Doi: 10.1038/srep16710

Method:

Synthesis of Graphene from coal tar pitch: The coal tar pitch (softening point = 60.8 °C, RIST) was diluted with quinoline solvent (JUNSEI Chemical) to a concentration of 8 wt%. Before spin-coating, SiO_2 (500 nm)/Si substrates (2 cm × 2 cm) (NAMKANG HI-TECH) were treated with UV/ozone for 30 min to establish good wetting between coal tar pitch (CTP) solution and substrate and then a 20-nm thickness of CTP film was deposited on SiO_2/Si substrates uniformly by spin coating (6000 rpm, 60 s). After baking at 240 °C for 30 min, the Ni layer (200 nm) was deposited (Magnetron Sputtering System, SNTEK; working pressure 7 mTorr; power 50 W; Ar flow rate 50 sccm) on a coal tar pitch film at room temperature. Then the sample was annealed using thermal chemical vapour deposition (CVD) (1100 °C, 4 min) with flowing 50 sccm Ar and 10 sccm H_2 at a total pressure of ~0.3 Torr, then cooled to room temperature by removing the tube from the furnace while maintaining the same Ar/H_2 flow. The Ni layer was etched away by dipping the sample into $FeCl_3$ solution (Iron(III) chloride solution 45°Be’, JUNSEI Chemical) for 1 min and the sample was rinsed with DI water.

Context 5

Author: Aamir Ahmed

Doi: 10.1016/j.mseb.2024.117828

Method:

Synthesis of carbon: The bubble wraps were washed 5-times with DI water. After drying, these sheets were cut into tiny pieces and put into a petri dish. The dish was then heated at 100 °C to melt the pieces followed by solidifying. These solidified sheets were chopped into small pieces and immersed in a beaker with concentrated H_2SO_4. The beaker containing the sample was transferred to a muffle furnace and subjected to thermal treatment at 150 °C for a duration of 3 h. The excess sulphuric acid was drained out and the resultant was a black hard mass. The acquired solid was subjected to repeated washing with deionized water until a neutral pH was attained. This sulphonation step was followed by carbonization. The centrifuged sample was heated in a muffle furnace at 500 °C for 1 h. The sample then obtained was washed with DI water, dried at room temperature, and reduced to powder using mortal and pestle. Conversion of carbon into reduced graphene oxide (rGO): In this process, 1 g synthesized carbon and 1 g $NaNO_3$ were added to 45 ml of H_2SO_4 (kept in an ice bath) and

stirred for 30 min until a homogenous black solution is produced. To this solution, 5 g KMnO4 was slowly added and stirred at 50 °C for 2 h forming a green solution. 100 ml DI water and 6 ml H2O2 (35 %) were added drop-by-drop to stop the reaction. The precipitates were washed with 300 ml HCl (10 %) and 200 ml HCl (37 %) in a centrifuge. The final sample was heated at 250 °C in a muffle furnace, again washed with DI water, and reduced to rGO powder.

RAG-Gemini

None of the provided contexts describe the synthesis of graphene directly via pyrolysis. The methods described include:

* Thermal decomposition of SiC wafers in an ultra-high vacuum (UHV) chamber.

* Chemical Vapor Deposition (CVD) using Ni foil as a substrate with CH4/H2/Ar gas mixtures.

* Ambient Pressure Chemical Vapor Deposition (APCVD) using Ar, H2, and a heated precursor (polystyrene).

* Thermal CVD of a coal tar pitch film on a Ni layer.

* Carbonization (a form of pyrolysis) of bubble wraps to produce carbon, which is subsequently converted to reduced graphene oxide (rGO) via chemical treatment.

In general, the synthesis of graphene via pyrolysis typically involves the following steps:

1. **Precursor Preparation**: A carbon-rich precursor material (e.g., polymers, biomass, organic compounds) is selected and prepared, sometimes into a specific form like a film or powder.

2. **Thermal Treatment**: The precursor is subjected to high temperatures, usually in an inert atmosphere (e.g., argon, nitrogen) or vacuum, to prevent oxidation. This heating causes the precursor to decompose, and its carbon atoms rearrange into graphitic structures.

3. **Graphene Formation**: Depending on the specific precursor, temperature, and heating rate, the carbon atoms can reassemble into graphene layers. This process might be referred to as carbonization or graphitization.

4. **Cooling and Collection**: The resulting material is then cooled, often under controlled conditions, and collected for further processing or characterization.

Ground truth

Pyrolysis is done by first transforming organic materials like biomass into a carbonaceous material followed by further processing into graphene structures. First, biomass sources, such as rice husks or wheat straw are selected and pretreated by grinding, drying or in some cases, treated with chemicals like KOH to enhance carbonization. Pyrolysis is then carried out in a controlled, inert environment in a furnace or reactor. The temperature is gradually increased in the chamber in the reactor (typically between 600 to 1,200 degrees C), which causes the precursor material to decompose, thereby releasing volatile components and leaving behind a carbonaceous residue. The carbonaceous material is then post processed such as acid washing and annealing to improve the material's structural order to become graphene.

Question 7

What are the steps involved to synthesize graphene via liquid phase exfoliation?

Context 1

Author: Emanuele Lago

Doi: 10.1039/C6RA21962D

Method:

Graphene dispersion is produced by liquid phase exfoliation (LPE) of natural graphite. 500 mg of graphite flakes are dispersed in 50 mL of NMP and exfoliated in a sonic bath (VWR Ultrasonic Cleaner USC-THD) for 6 hours. Then, the dispersion is ultra-centrifuged at 10000 rpm (~17000 g), using sediment based separation (SBS) to remove un-exfoliated and thicker graphite flakes, for 30 min at 15 °C with an ultra-centrifuge (Beckman Coulter Optima™ XE-90, equipped with a SW32Ti rotor). Finally, the supernatant is collected by pipetting. A solvent exchange process is carried out for the re-dispersion of the exfoliated flakes in 1,3-dioxolane, a nontoxic and low boiling point (78 °C) solvent, using a Heidolph Hei-Vap rotary evaporator. After the evaporation process of N-methyl-2-pyrrolidone (NMP), the graphitic material is collected and washed three times with acetone to remove the NMP residual using a compact centrifuge (Sigma-Aldrich). The washing step is repeated and the flakes are eventually dispersed in 50 ml of 1,3-dioxolane, adjusting its concentration at 10 mg mL-1.

Context 2

Author: Kewen Pan

Doi: 10.1038/s41467-018-07632-w

Method:

Liquid exfoliation of graphene with high concentration: Expandable graphite with +50 mesh flake size was purchased from Sigma-Aldrich. Cyrene (dihydrolevoglucosenone, >99%) was provided by Circa Group Pty Ltd. N-methyl-2-pyrrolidinone (NMP, >99%) was from Alfa Asia. CAB (butyryl content 35–39%) was from Arcos Organics. Graphene nanoflakes were obtained via the liquid phase exfoliation method. Expandable graphite were placed in a ceramic crucible and then heated in an 800 W commercial microwave oven for 30 s to obtain expanded graphite with fewer layers. The expanded graphite flakes were stirred and washed in deionized water to remove residual acid until the pH is close to 7. Then, the mixture was dried in an oven for 5 h at 100 °C. Dried expanded graphite was deposited into NMP solvent (10 mg mL−1) and Cyrene (10 mg mL−1), respectively where these organic solvents provide appropriate surface energy for sonication processes. After that, the mixture was sonicated in an ultrasonic bath (SHESTO, UT8061-EUK). Samples were extracted at 0, 2, 4, 6, 8, 10, 12, 14, 16, 20, 24, 32, 40, 48, and 56 sonication hours for sheet resistance measurement.

Context 3

Author: Ethan B. Secor

Doi: 10.1002/adma.201502866

Method:

Graphene was exfoliated from graphite using a high shear mixer (Silverson L5M-A) with a square hole high shear screen. Ethyl cellulose (EC) (Sigma–Aldrich, 4 cP grade as measured at 5% in 80:20 toluene: ethanol, 48% ethoxy) was dissolved in ethanol (Koptec, 200 proof) at a concentration of 0.2–2% w/v, and flake graphite (Asbury Graphite Mills, Grade 3061) was added at 10% w/v. This mixture was shear mixed for 2 h at 10,230 rpm in an ice bath, and then centrifuged at 4000 rpm (≈3000 g) for 2 h to sediment out large graphite flakes (Beckman Coulter Avanti J-26 XPI centrifuge). The supernatant containing graphene, EC, and ethanol was harvested by pipette. For the flocculation step, this supernatant was mixed in a 16:9 wt. ratio with an aqueous NaCl solution (0.04 g/mL NaCl, Sigma–Aldrich, >99.5%) and centrifuged for 6 min at 7500 rpm (≈10 000 g) to sediment the graphene/ EC composite. This sediment was washed with deionized water, collected by vacuum filtration (Millipore Nitrocellulose HAWP 0.45 μm filter paper), and then dried to yield the graphene/EC powder, with a graphene content of 25–65 wt% depending on the starting EC loading.

Context 4

Author: Qingbin Zheng

Doi: 10.1021/nn2018683

Method:

Five grams of natural graphite flakes (Asbury Graphite Mills) and 150 mL of sulfuric acid (H2SO4, General Chemical) were mixed and stirred in a round-bottom flask at a speed of 200 rpm. Fifty milliliters of fuming nitric acid was added into the mixture. The mixture was kept at room temperature and stirred for 24 h. Two-hundred milliliters of deionized water was then poured slowly into the mixture. The resultant mixture was washed using DI water, followed by centrifugation and drying at 60 °C for 24 h to obtain graphite intercalation compound. The dry graphite intercalation compound powder was thermally expanded at 1050 °C for 15 s to obtain expanded graphite that was used for the production of graphene oxide (GO). One gram of expanded graphite and 200 mL of sulfuric acid were mixed and stirred in a three-neck flask. Ten grams of KMnO4 was added to the mixture while stirring. The solution was transferred into an ice bath, and 200 mL of deionized water and 50 mL of H2O2 were poured slowly into the mixture, realizing color change of the suspension to light brown. The GO particles were washed and centrifuged with a HCl solution three times, then centrifuged again and washed with deionized water until the pH of the solution became about 5 to 6. The GO particles obtained thereby were diluted using DI water (~1 mg/mL) and dispersed by gentle shaking.

Context 5

Author: Nai Gui Shang

Doi: 10.1039/C2CC17185F

Method:

Graphite powders of 50 mg (99.0% purity, from Sigma-Aldrich) with a grain size less than 20 μm were mixed and ground with 0.1—0.5 mL ionic liquid (IL, 1-butyl-3-methylimidazolium hexafluorophosphate, BMIMPF6, from Sigma-Aldrich) for 0.5–4 hrs. Then the mixtures were added into a solution of 15 mL N,N-dimethylformamide (DMF) and 15 mL acetone and centrifuged at a speed of 3000 rpm for 30 min in order to remove the ionic liquid. This washing cycle was repeated three times, and the final sediment was dispersed in 1 L DMF. After one-day sedimentation, the large/thick graphitic flakes completely precipitated on the bottom of the bottle. The supernatant was dried, and the yield was calculated as 20 wt%, which is much larger than that of other methods reported. The supernatant was collected and centrifuged to get a dense suspension, with which the graphene samples were prepared for the study of their microstructure and electrical properties. The suspension was diluted and dropped on lacy carbon-coated Cu grid for TEM observations, on clean Si wafers or mica sheets for the XPS, Raman, and AFM studies, and on thermally oxidised Si wafer substrates (300 nm SiO2) for the study of electrical properties. To obtain uniform large-area graphene thin films, the Si/SiO2 substrate was functionalised with 5% 3-triethoxysilylpropylamine (APTES) aqueous solution for 30 min. All samples were completely dried under an infrared light or on a hot plate before measurements. We should note the role of ILs in this study. ILs are low-temperature molten salts, and as their name denotes, are liquids composed entirely of ions. They have been proposed as a new class of “green” organic solvents because not only are they able to solvate a large variety of organic and inorganic compounds (polar or non-polar), but they also possess high thermal and chemical stability, high ionic conductivity, wide electrochemical window, and negligible vapour pressure. The mixing of a small quantity of IL and graphite flakes forms a gel-like composite. When graphite flakes are ground with ionic liquids, the shear force detaches the graphene layers from the graphite flakes. The ionic liquids can effectively surround each layer, preventing the detached graphenes from restacking. Based on simulation and experimental studies on the processing of carbon nanotubes with imidazolium-based ILs, the electronic structure of graphene layers in the bucky gel remains unchanged, and there is no charge transfer between graphene and imidazolium cations

RAG-Gemini

The synthesis of graphene via liquid phase exfoliation (LPE) typically involves the following steps:

1. **Preparation and Dispersion:** Graphite, often in the form of natural or expandable flakes, is dispersed in a suitable solvent or solvent mixture. Examples of solvents include N-methyl-2-pyrrolidone (NMP), Cyrene (dihydrolevoglucosenone), or ethanol (sometimes with additives like ethyl cellulose). In some cases, graphite may be pre-treated, such as heating expandable graphite to obtain expanded graphite, or ground with an ionic liquid.

2. **Exfoliation:** The dispersed graphite undergoes an exfoliation process to delaminate individual graphene layers. This step applies energy to overcome the van der Waals forces holding the graphite layers together. Common methods include:

* **Ultrasonication:** Exposing the dispersion to ultrasonic waves using a sonic bath for several hours.

* **High Shear Mixing:** Stirring the mixture vigorously at high RPMs using a high shear mixer.

* **Mechanical Grinding:** Grinding graphite flakes with an ionic liquid, where the shear force facilitates the detachment of layers.

3. **Separation:** After exfoliation, a separation step is crucial to remove unexfoliated, larger, or thicker graphite flakes from the exfoliated graphene. This is commonly achieved by:

* **Centrifugation:** The dispersion is centrifuged at a specific speed (e.g., 4000 rpm to 10000 rpm) for a set duration, causing larger particles to sediment while the finer graphene sheets remain suspended in the supernatant. The supernatant containing the graphene is then collected.

* **Sedimentation:** Allowing the large/thick graphitic flakes to settle over time, and collecting the supernatant.

4. **Post-Processing (Optional but common):**

* **Washing:** The collected graphene dispersion may be washed multiple times (e.g., with acetone or deionized water) to remove residual solvents, additives (like NMP), or impurities.

* **Solvent Exchange/Redispersion:** The graphene can be re-dispersed in a different, often more desirable solvent, sometimes after an evaporation process to remove the initial solvent.

* **Drying:** The graphene suspension can be dried (e.g., via vacuum filtration or on a hot plate) to obtain graphene powder or for further processing into films.

Ground truth

Graphite is first placed into some kind of liquid suspension medium, often ethanol, alcohol or some other kind of solvent, and then sonicated either through bath sonication or tip sonication. In bath sonication, the graphite-liquid suspension is usually placed into some kind of container before being placed in the bath sonicator. In tip sonication, the tip is inserted directly into the liquid. The suspension is then sonicated for a period of time. During the sonication, cavitation within th eliquids will cause the different graphene layers to exfoliate from one another. After a certain period of time, the suspensions are removed from the sonicator and then centrifuged. The supernatent of the liquid is then taken and characterised to confirm the presence of mono-layer or few-layer graphene. Surfactants can be utilised to maintain or improve the suspension but might cause issues with final device properties.

Question 8

What are the steps involved to synthesize graphene via laser ablation?

Context 1

Author: Rafal Hameed

Doi: 10.1016/j.matpr.2019.09.185

Method:

Graphene sheet has synthesis through pulsed laser ablation in liquid process of a pure (99.9%) graphite pellet. This pellet cut from a rod of graphite (5 mm diameter). The graphene sheet synthesis in two process steps, at the beginning carbon nanotubes CNTs synthesis, that accomplished using pulsed laser ablation of graphite pellet that put in the bottom of quartz vessel and immersed in (2 ml) water. The volume and the height (5 mm) of water were constant to make sure that a constant power reached to the target, the second step involved re-irradiation of the obtained colloidal that ready before and contained carbon nanotubes, after removing the target with the same laser energy. Pulsed Nd: YAG laser used of 1064 nm wavelength with output pulse duration is 7 ns and (80– 160 mJ) laser energies, for fixed pulses (100 pulses).

Context 2

Author: Min Yi

Doi: 10.1021/nn101796e

Method:

Sample Preparation (Laser Synthesis of Epitaxial Graphene on SiC): N-type (nitrogen, 3 × 1018 cm−3) 4H-SiC(0001) wafer, on-axis, with chemical mechanical polishing (CMP, Ra ≤ 1 nm) on the Si face was purchased from SiCrystal AG, which was used for RHEED, X-ray diffraction, and TEM characterizations. High-purity semi-insulating (ρ ≥ 1 × 105 Ω·cm) 4H-SiC(0001) wafer, on-axis, was also purchased from Cree: the wafer was epiready polished by NovaSiC up to an atomically flat surface (Rq ≤ 1 Å), which was used for STM and Raman measurements. The substrates (5 mm × 5 mm) were cut from the wafers and treated by sequential ultrasonic bath in acetone, methanol, and isopropyl alcohol (for 5 min to remove grease), Piranha cleaning (mixture of H2SO4 + H2O2 (1:1) for 3 min to wipe off metal powder and organic contaminants) and HF etching (10% HF solution for 3 min to remove the surface oxide). The sample was placed in a high-vacuum (HV) chamber (base pressure ≈ 10−7 Torr, pumpdown time ≈ 2 h) designed for pulsed laser deposition (PLD). The SiC surface was irradiated with a pulsed KrF excimer laser (Lambda Physik LPX 210i, λ = 248 nm, pulse length ≈ 25 ns) with a nonhomogenized beam. The beam intensity profile was measured with 1 mm interval at the removable beam blocker with hole (20 mm × 10 mm) near the laser exit port, and the regions with higher fluence than average were blocked by shadow mask (stainless

steel). The SiC substrate with specially designed holder was loaded, inside the chamber, perpendicular in the direction of the laser beam. The experiments were performed at ~1.1, ~1.2, and ~1.4 J/cm2 with 500 shots (20 Hz, 25 s) to synthesize monolayer, bilayer, trilayer graphene, respectively. The vacuum condition of ~10−6 Torr was used for all experiments.

Context 3

Author: Dapeng Wei

Doi: 10.1016/j.carbon.2012.11.026

Method:

Two 1 x 2 cm quartz wafers were used as substrate to grow graphene. The quartz wafers were first cleaned by ultrasonication in methanol, acetone, and DI water, and were dried by high purity N2 gas. Photoresist S-1805 (from Shipley Comp.) was diluted with a volume ratio of 1:6 in Thinner-P (from MicroChem Corp.), and was then spin-coated on the quartz wafer at 10,000 rpm. The thickness of the coated film is about 30 nm. The photoresist-coated quartz wafers were then baked for 5 min at 120°C . One coated quartz wafer was covered by another piece of quartz wafer, and then mounted on a sample stage in a vacuum chamber. Before growing graphene, the chamber was pumped and purged by high-purity N2 gas, and maintained at a pressure below 0.1 Torr. A continuous wave (CW) Nd:YAG laser with a wavelength of 532 nm was focused on the S-1805 film through the transparent quartz substrate using a lens of 150 mm focal length. The carbon atoms were decomposed from the laser heated photoresist, then dissolved in the molten quartz, and extracted to form graphene when the temperature of quartz was decreased. With our optical setup, the graphene was produced with a laser power of 2.8 W, irradiated for 3–5 min.

Context 4

Author: Emanuele Pompei

Doi: 10.1039/D4NR01986E

Method:

The porousified SiC wafers are cut into pieces (2×8 mm2) by a wafer saw. Pieces are individually transferred to an ultra-high vacuum (UHV) chamber where they are degassed overnight at 1000 K. After the degassing, samples are cooled to room temperature (RT) and heated again to 1650 K to allow the graphene formation on the surface of the pores. After 150 s of annealing, the sample is let to cool down to room temperature. It was observed that the temperature reached by the sample during the annealing depends on the quality of the mechanical contact between the sample and the sample holder. Therefore, by clamping one side of the sample more firmly than the other, we obtained the non-homogeneous graphenized sample discussed in the Functionalization with gold nanoparticles section.

Context 5

Author: Fatemeh Kazemizadeh

Doi: 10.1016/j.physb.2017.11.052

Method:

Powdering graphite combined with nickel particles at a concentration of 2 at% was obtained using ball milling process. The powder was pressed under 1300 kg cm-2 for 15 min, and a graphite pellet was formed. The targetwas embedded in a quartz tube, 5 cm in diameter and 80 cm in length and argon gas was flowed through it at the rate of 2.4 l/min. The tube was put in a furnace, 1050°C and pressurized at 300 mbar. Fundamental mode of a Q-switched pulsed Nd-YAG laser (1064 nm in wavelength), with 10ns pulse duration, and repetition rate of 10 Hz, was focused on the carbon pellet so that the illuminated area reduced to 12 mm2. Carbon atoms generated by ablation were gathered on a collector on a water-cooled metal surface in the end of the tube. The synthesized product was then scraped off the collector and analyzed. The experiment was carried out with two different laser fluences of 3.5 and 5.5 J/cm2. The last laser energy was irradiated on a graphite pellet without metal particles for further investigation.

RAG-Gemini

The synthesis of graphene via laser ablation involves ablating a carbon source with a laser in a controlled environment, followed by collection or in-situ formation of graphene. The specific steps can vary depending on the environment:

1. Pulsed Laser Ablation in Liquid (PLAL):

* A graphite pellet is prepared and immersed in a liquid, such as water, within a vessel.

* A pulsed laser is focused on the graphite target, causing ablation and initial synthesis of carbon nanotubes (CNTs) in a colloidal solution.

* The graphite target is then removed.

* The obtained colloidal solution, containing CNTs, is re-irradiated with the same laser energy to facilitate the conversion of CNTs into graphene sheets.

2. Pulsed Laser Ablation in Gas/Furnace:

* A graphite pellet, which may optionally be combined with metal particles, is prepared (e.g., by powdering and pressing).

* The graphite target is placed inside a tube (e.g., a quartz tube).

* An inert gas, such as argon, is flowed through the tube, and the tube is typically heated in a furnace and pressurized.

* A pulsed laser is focused onto the carbon pellet, leading to the generation of carbon atoms through ablation.

* These ablated carbon atoms are then gathered on a collector, such as a water-cooled metal surface, where graphene is formed.

* The synthesized graphene product is subsequently scraped off the collector for further analysis.

Ground truth

Porous graphene can be made using the laser ablation of a nickel-graphite target under the flow of Argon gas. Although not technically a traditional graphene layer, porous graphene is a similar carbon allotrope with an inhomogenous structure and curvature on the graphene layers. First graphite and nickel particles are ball milled and then pressed into a graphite-nickel pellet under pressure to form a target. The target is then placed into a quartz tube where argon is allowed to flow at about 2.4l/min. The tube is then heated to 1050 degrees celsius and presurised at 300mbar. A Nd-YAG laser (1064nm in wavelength), with a 10 ns pulse duration and repetition rate of 10Hz is then focused on the carbon pellet. The illuminater area is about 12 mm2. The carbon atoms generated by this ablation are carried by the argon gas and then gathered on a collector on a water-cooled surface at the end of the tube and scraped off as porous graphene.

Question 9

What are the steps involved to produce graphene oxide?

Context 1

Author: Maria Refalo Magro

Doi: 10.1016/j.cartre.2025.100509

Method:

The process of separating and oxidizing graphite into graphite oxide and eventually graphene oxide involves the following steps. Initially, 1 g of graphite flakes was combined with 150 mL of concentrated H_2SO_4 and mechanically stirred at 400 rpm for approximately 10 minutes at room temperature. 4 g of $KMnO_4$ were then added incrementally, in 1 g portions, every hour. This incremental addition was done to manage the generation of Mn_2O_7 and mitigate the risk of explosive decomposition.Subsequently, the mixture was left stirring at 900 rpm for 24 h at room temperature. A total of 150 mL of deionized (DI) water was then added in small volumes making sure the temperature of the mixture did not exceed 90 °C with each addition.After cooling down, a few drops of 30 % hydrogen peroxide (H_2O_2) were added until no further effervescence occurred. The peroxide addition serves to reduce and eliminate residual permanganate. The resulting mixture was then subjected to centrifugation (Hermle Z366) at 8820 rpm (rcf = 10,000), to isolate the graphite oxide from the supernatant which was discarded. The graphite oxide was then redispersed in deionized water and the procedure (centrifugation followed by re-dispersion) was repeated several times until the supernatant's final pH reached 6 – 6.5. The final dispersion stage (from graphite oxide to graphene oxide) involved sonication for 1 h at 100 % power and a frequency of 80 kHz at room temperature using an Elmasonic P30H sonicator bath. This critical step allowed for the separation of the layers comprising graphite oxide, yielding free graphene oxide sheets. The sonicated suspension was then dried in an oven for 48 h at 40 - 50 °C resulting in the production of a thin dark-brown graphene oxide 'paper'.

Context 2

Author: Dheeraj

Doi: 10.1016/j.poly.2024.117175

Method:

Graphene oxide was produced employing a modified Hummer's method. Initially, a mixture of H_2SO_4 and H_3PO_4 in a 9:1 vol ratio was prepared and placed in a 500 mL round bottom flask, where it was vigorously stirred. Subsequently, 0.55 g of graphite powder was introduced into the acid mixture while maintaining continuous stirring. After achieving complete homogenization, 2.74 g $KMnO_4$ was gradually poured into the solution with vigorous stirring. The solution exhibited a transition to a dark green color during this process, and a small

quantity of H2O2 was added dropwise, causing an exothermic reaction. To control the reaction's temperature, the entire process was conducted within an ice bath. Following this, a solution composed of 10 mL of hydrochloric acid and 50 mL of deionized water was introduced into the mixture over a span of 10 min. The resulting residue was carefully collected and subjected to multiple wash cycles with HCl and DI water. Lastly, the precipitated material was desiccated in an oven at 70 °C for a duration of 12 h.

Context 3

Author: Qingbin Zheng

Doi:10.1021/nn2018683

Method:

Five grams of natural graphite flakes (Asbury Graphite Mills) and 150 mL of sulfuric acid (H2SO4, General Chemical) were mixed and stirred in a round-bottom flask at a speed of 200 rpm. Fifty milliliters of fuming nitric acid was added into the mixture. The mixture was kept at room temperature and stirred for 24 h. Two-hundred milliliters of deionized water was then poured slowly into the mixture. The resultant mixture was washed using DI water, followed by centrifugation and drying at 60 °C for 24 h to obtain graphite intercalation compound. The dry graphite intercalation compound powder was thermally expanded at 1050 °C for 15 s to obtain expanded graphite that was used for the production of graphene oxide (GO). One gram of expanded graphite and 200 mL of sulfuric acid were mixed and stirred in a three-neck flask. Ten grams of KMnO4 was added to the mixture while stirring. The solution was transferred into an ice bath, and 200 mL of deionized water and 50 mL of H2O2 were poured slowly into the mixture, realizing color change of the suspension to light brown. The GO particles were washed and centrifuged with a HCl solution three times, then centrifuged again and washed with deionized water until the pH of the solution became about 5 to 6. The GO particles obtained thereby were diluted using DI water (∼1 mg/mL) and dispersed by gentle shaking.

Context 4

Author: Abhijit Ganguly

Doi: 10.1021/jp203741y

Method:

Highly oxidised Graphene Oxide (GO) was produced using a modified Hummers' process. The starting material Graphite powder with particle size ≤20 μm was purchased from Fluka and is denoted here as "pristine" graphite. All other chemical and reagents were purchased from Aldrich. A mixture of 2.5 g of Graphite and 1.9 g of NaNO3 was placed in a flask cooled in an ice bath. 85 mL of H2SO4 was added to the mixture and stirred until homogenized. Solution of 11.25 g of KMnO4 in distilled water was gradually added to the solution while stirring. After 2 hours, the solution was removed from the ice bath, and further stirred for 5 days. Finally, brown-coloured viscous slurry was obtained. The slurry was added to 500 mL aqueous solution of 5 wt% H2SO4 over 1 hour while being continuously stirred. The mixture was stirred for a further 2 hours. Subsequently, 10 ml of H2O2 (30 wt% aqueous solution) was

then added to the mixture and stirred for further 2 hours. This mixture was then left to settle overnight. The mixture was filtered and further purified by dispersing in 500 mL aqueous solution of 3 wt% H2SO4 and 0.5 wt% H2O2. After two days of precipitation, the supernatant solution was removed. This process was repeated five times. The solid product obtained after the rigorous cleaning process was rinsed using copious amounts of distilled water and dried in oven, as reported in literature. The resulting solid was dispersed in water by ultrasonication for 2 h to produce a GO aqueous dispersion. After one-day sedimentation, the thick flakes were removed and the supernatant was collected for further measurements.

Context 5

Author: Jinping Zhao

Doi: 10.1021/nn1015506

Method:

The detailed experimental procedure for the preparation of large-area graphene oxide (GO) sheets is described as follows. A 2 g portion of natural flake graphite with an average size of 500−600 μm, 2 g of NaNO3, and 96 mL of concentrated H2SO4 were mixed at 0 °C. During the following stages the mixture was continuously stirred using a magnet stirrer. 12 g of KMnO4 was gradually added to the above mixture while keeping the temperature at 0 °C. The mixture obtained was first stirred at 0 °C for 90 min and then at 35 °C for 2 h. Distilled water (80 mL) was slowly dropped into the resulting solution, over a period of around 30 min, to dilute the mixture. Then 200 mL of distilled water was added followed by 10 mL of H2O2 (30%), and the stirring continued for 10 min to obtain a graphite oxide suspension. During this final step, H2O2 (30%) reduced the residual permanganate and manganese dioxide to colorless soluble manganese sulfate. The graphite oxide deposit was collected from the graphite oxide suspension by high-speed centrifugation at 16000 rpm for 10 min, and repeatedly washed with distilled water until the pH = 7. Then a mild sonication (80 W, 5 min) was used to exfoliate the graphite oxide to obtain a GO suspension. To obtain uniform large-area GOs, a low-speed centrifugation at 3000 rpm was first used to remove thick multilayer flakes until all the visible particles were removed (3−5 min). Then the supernatant was further centrifuged at 5000 rpm for 5 min to separate large flakes (precipitate) and small flakes (supernatant). Finally, the obtained precipitates containing large flakes were redispersed in water to get a large-area GO sheet suspension. To determine the yield of this process, we measured the weight of the above precipitate containing large flakes after drying in a vacuum oven at 90 °C for 24 h to completely remove the water. The weight of large flakes obtained from the original 2 g of sample was about 0.25 g.

RAG-Gemini

The production of graphene oxide typically involves the following steps:

1. **Preparation of Graphite-Acid Mixture**: Graphite (flakes or powder) is combined with strong acids, most commonly sulfuric acid (H2SO4), and sometimes nitric acid (HNO3) or phosphoric acid (H3PO4), often along with other reagents like NaNO3. This mixture is continuously stirred.

2. **Oxidation**: An oxidizing agent, usually potassium permanganate (KMnO4), is gradually added to the mixture. This step is often performed under controlled temperature conditions (e.g., in an ice bath or at specific temperatures like 0 °C to 35 °C) to manage the exothermic reaction. The mixture is stirred for an extended period (hours to days) to ensure thorough oxidation.

3. **Dilution and Quenching**: The reaction mixture is diluted by slowly adding deionized water. Hydrogen peroxide (H2O2) is then introduced to reduce any residual permanganate and manganese dioxide, typically causing effervescence or a color change.

4. **Washing and Purification**: The resulting graphite oxide material is separated from the supernatant, often through centrifugation, and then subjected to multiple washing cycles. Washes typically involve hydrochloric acid (HCl) and deionized water until the pH of the supernatant becomes neutral or near-neutral (e.g., 5-7).

5. **Exfoliation**: The purified graphite oxide is dispersed in deionized water and exfoliated into individual graphene oxide sheets, commonly achieved through sonication (ultrasonication).

6. **Drying**: The graphene oxide suspension is then dried, usually in an oven, to obtain the solid graphene oxide product.

Ground truth

Graphene oxide (GO) can be synthesized by the oxidation of graphite into graphite oxide followed by the exfoliation of graphite oxide into GO. The leading method right now is the modified Hummer's method. A common procedure is as follopws: First, mix graphite and potassium permanganate in a 1: 6 ratio by weight. Then, in another beaker, mix 96% sulphuric acid and then add 75% phosphotic acid. The acids should be in a 6:1 ratio. Ensure the acids are added to the graphite and potassium permanganate slowly and bit by bit while constantly stirring. This allows for the intercalation of the graphite and oxidation of the graphite into graphite oxide. Stir the entire solution at 50 degrees centigrade for 12 hours and then allow to cool to room temperature. Next, pour the entire acid mixture into another beaker with ice made from de-ionised water. Add about 3mL of 30% hydrogen peroxide to the mixture and stire to allow the GO to exfoliate.

Question 10

What are the steps involved to produce reduced graphene oxide?

Context 1

Author: Benjamin Diby Ossonon

Doi:10.1039/c6ra28311j

Method:

Graphene oxide was synthesized from natural graphite through the Hummers method which has been improved. The graphite is first pre-oxidized by mixing 5g graphite powder with concentrated sulfuric acid (12.5 mL), potassium persulfate (2.5 g) and phosphorus pentoxide (2.5g). The mixture was heated at 80°C for 6 hours. After dilution with 500 mL of H2O, the mixture was stirred at room temperature overnight. After that, the product is recovered by centrifugation and washed thoroughly with Nanopure water until the filtrate has a pH close to 7. The product obtained is then dried at room temperature for one day. Then, the pre-oxidized graphite is dispersed in H2SO4 (0°C, 115 mL). The temperature of the mixture is carefully controlled to not exceed 10°C. Subsequently, potassium permanganate (15 g) is gradually added with constant stirring for 1 hour. The dispersion is then incubated at 35°C for 2 h and this is followed by the addition of Nanopure water (225 mL) in small portions (15 mL) to control the temperature of the mixture, which must remain below 50°C. To completely dissolve the KMnO4 , hydrogen peroxide (30%, 12.5 mL) was immediately added at the end of a second dilution (H2O, 700mL), and the mixture is stirred for 48 hours. Finally, the suspension is filtered, washed first with HCl (10%) to remove residual metal ions, and repeatedly with Nanopure water until the pH of filtrate becomes neutral. The filtrate is quickly tested by adding a few drops of 1 M NaOH to verify the presence of metal ions in graphene oxide (GO). The product obtained (graphite oxide) is then dried in air. The resultant graphite oxide was dispersed in Nanopure water kept in the ultrasonic bath for 24 hours to maximize exfoliation. The reduced graphene oxide (RGO) is obtained by thermal reduction of GO in Ar/5% H2 at 800°C for 2 h. The resulting RGO can be dispersed in water and the dispersion stayed stable for few hours.

Context 2

Author: Tran Van Khai

Doi: 10.1039/C2JM33194B

Method:

Graphene oxide (GO) was synthesized from graphite powder via a modification of Hummers and Offeman's method. In a typical reaction, 5 g of graphite, 60 mL of H3PO4, and 180 mL of H2SO4 were stirred together with a Teflon-coated magnetic stirring bar in an ice bath. Next, 60 g of KMnO4 was slowly added while the temperature was maintained at 0 °C. Once mixed,

the solution is transferred to a 35 ± 5 °C water bath and stirred for 3 h, forming a thick paste. Next, distilled water (450 mL) was slowly dropped into the resulting paste to dilute the mixture, and then the solution was stirred for 1 h while the temperature was raised to 90 ± 5 °C. Finally, 800 mL of distilled water was added, followed by the slow addition of 60 mL H2O2 (30%), turning the color of the solution from dark brown to yellow. During this final step, H2O2 (30%) reduced the residual permanganate and manganese dioxide to colorless soluble manganese sulfate. The GO deposit was collected from the GO suspension by high-speed centrifugation at 15 000 rpm for 30 min. The obtained GO was then washed with 1000 mL of HCl (5%) and repeatedly washed with distilled water until pH = 7. To obtain uniform GO, a low-speed centrifugation at 3000 rpm was first used to remove thick multilayer sheets until all the visible particles were removed (3–5 min). Then the supernatant was further centrifuged at 10 000 rpm for 30 min to remove small GO pieces and water-soluble byproducts. The final sediment was redispersed in 500 mL of DMF with mild sonication, resulting in a solution of exfoliated GO. In general, for chemically reduced GO (RGO), 500 mL of exfoliated GO was stirred for 30 min and 10 mL of hydrazine monohydrate was added. The mixtures were heated at 150 ± 5 °C in an oil bath for 24 h; a black solid precipitated from the reaction mixtures. Products were collected by centrifugation at 12 000 rpm for 45 min and washed with DI water and methanol until pH = 7. Next, the obtained RGO was dried and stored in a vacuum oven at 60 °C until use.

Context 3

Author: Nahid Rehman

Doi: 10.1016/j.sbsr.2025.100789

Method:

The process of producing graphene oxide (GO), the precursor of reduced GO (rGO), is derived from a modified Hummer's approach, which is non-explosive and non-toxic. Using ice baths with magnetic stirring allows control over the highly exothermic processes that produce GO. The modified Hummers approach synthesized the reduced graphene oxide. In a 500 ml beaker, add 27 ml of H2SO4:H3PO4 (9:1) and stir for a minute. To this solution, add 225 mg of graphite powder under continuous stirring. Slowly add 1.32 g of KMnO4; this step follows the exothermic reaction; therefore, necessary precautions should be taken. Leave the solution stirring for 6 h unless the dark green color of the solution is observed. Allow the contents of the beaker to cool down at room temperature. Add dropwise 0.675 mL of 30 % H2O2 on the ice bed and stir it for 10 min at room temperature to remove excess KMnO4. When the mixture is cooled down (exothermic nature of reaction), add 10 mL of 30 % HCl and 30 mL of distilled water (DW) followed by centrifugation at 5000 rpm for 7 min. Decant the supernatant obtained and wash thrice with the HCl and DW mix. Oven-dry the leftovers for 24 h at 50 °C to procure the graphene oxide powder. The color change from brown to black is indicative of GO reduction. This work employed the thermal reduction approach to acquire the reduced form of graphene oxide. The process of chemically reducing GO to rGO requires the use of hazardous and non-environmentally friendly chemical reagents. After the produced GO was heated to 60 °C for three days in a hot air oven, the mass yield of the material decreased as a result of the removal of oxygenated functional groups and any remaining water molecules between the graphene sheets, which allowed rGO to develop through thermal reduction

Context 4

Author: Miftahul Husnah

Doi: 10.1088/2053-1591/aa707f

Method:

Synthesis of graphite oxide: The reduced graphene oxide (rGO) synthesis process was started by preparing 9:1 mixture of H_2SO_4/H_3PO_4 (23 ml) and adding 1 g graphite flakes. Afterwards, 3 g $KMnO_4$was slowly added into the solution and maintained below 10 °C during the addition process. The mixture was stirred for 40 min at 50 °C and then diluted with deionized water. 30% hydrogen peroxide (H_2O_2) was added to stop the oxidation process. The addition of H_2O_2 into the solution changed its color into yellowish green with bubbles that indicated a high degree of oxidation process. The solution was filtered and washed until the PH close to 7. Then, the product was heated at 60 °C for 12 h. Synthesis of graphene oxide (GO) and rGO: Colloids of GO were prepared by dispersing into 133 ml ethylene glycol (EG). While stirring, the rGO was prepared by dripped hydrate hexahydrate (N_2H_4). The reduction process was completed by introducing the sample into a conventional microwave for 20 min. Afterwards, the sample was filtered and washed using deionized water and alcohol, and dried for 12 h.

Context 5

Author: Ruichao Zhao

Doi: 10.1016/j.matchemphys.2025.130850

Method:

Typically, flake graphite (10 g), $KMnO_4$ (6 g) and K_2FeO_4 (4 g) as the oxidants, and boric acid (0.01 g) as a stabilizer were first dispersed in 100 mL of concentrated sulfuric acid in a vessel and stirred for 1.5 h at less than 5 °C. After the addition of another $KMnO_4$ (5 g), the vessel was transferred into a water bath at about 35 °C and stirred for another 3 h to complete the deep oxidation. Next, as 250 mL of deionized water was slowly added, the temperature was adjusted to 95 °C and held for 15 minutes, when the diluted suspension turned brown, indicating the hydrolysis and absolute exfoliation of intercalated graphite oxide. Finally, this brown suspension was further treated with 12 mL H_2O_2 (30%) to reduce the residual oxidants and intermediates to soluble sulfate, then centrifuged at 10000 rpm for 20 min to remove the residual graphite, and washed with 1 mol/L HCl and deionized water repeatedly, producing the terminal GO. Thereafter, reduced graphene oxide (rGO1, rGO2) was prepared via a facile hydrothermal procedure as follows. GO colloidal (2 mg·mL-1, 100 mL) was ultrasonicated for 30 min to reach complete dispersion. Subsequently, $C_6H_8O_6$ was added into the above colloidal and subject to another 30 min ultrasonication to form a uniform dispersion. The as-formed dispersion was then transferred into an autoclave for hydrothermal treatment at 180 ℃ for 12 h. Finally, the solid product was cleansed and dried under 60 ℃ to obtain reduced graphene oxide. Quantitatively, usage of 0.8000 g, 1.2000 g of $C_6H_8O_6$ yields reduced graphene oxide sample rGO1, rGO2, respectively.

RAG-Gemini

Reduced graphene oxide (RGO) is typically produced by reducing graphene oxide (GO). The methods for obtaining RGO from GO vary:

*   **Thermal Reduction:** Graphene oxide can be thermally reduced by heating. For example, GO can be heated in an Ar/5% H2 atmosphere at 800°C for 2 hours, or in a hot air oven at 60°C for three days.

*   **Chemical Reduction:**

    *   **Using Hydrazine Monohydrate:** Exfoliated GO is stirred with hydrazine monohydrate, then the mixture is heated at 150 ± 5 °C in an oil bath for 24 hours. The resulting solid is collected by centrifugation and washed with deionized water and methanol, then dried.

    *   **Using N2H4 with Microwave Treatment:** GO is dispersed in ethylene glycol, and N2H4 is added while stirring. The reduction process is completed by introducing the sample to a conventional microwave for 20 minutes. The product is then filtered, washed with deionized water and alcohol, and dried.

    *   **Using Ascorbic Acid (C6H8O6) via Hydrothermal Method:** A GO colloidal dispersion is ultrasonicated, C6H8O6 is added, and the mixture is ultrasonicated again to form a uniform dispersion. This dispersion is then transferred into an autoclave for hydrothermal treatment at 180°C for 12 hours. The final solid product is cleansed and dried.

Ground truth

After GO is produced from the modified Hummer's method the GO can be reduced chemically using a reducing agent such as hydrazine, ascorbic acid or even sugars to reduce it to reduced graphene oxide (rGO). Alternatively, GO can be thermally reduced by heating it up at high temperature in an inert atmosphere like argon or in the presence of hydrogen. This will break down the oxygen-containing groups on the GO causing them to volatilize and restore the carbon framework. it should be nmoted that this process is often incomplete and bcan produce rGOP with a lot of defects that affect conductivity.

Question 11

"What are the steps involved to produce graphene quantum dot?

Context 1

Author: Satyaprakash Ahirwar

Doi: 10.1021/acsomega.7b01539

Method:

Graphite rods are purchased from Alfa Aesar. The graphite rods with a diameter of 3.05 mm and an approximate length of 60 mm are heated in a furnace (at 1050°C for 5 min) in the presence of air. After 5 min, the graphite rods are allowed to cool at room temperature. Thereafter, the graphite rods are washed with Milli-Q water to remove large particles from the surface of the graphite rods. The graphite rods are then used as anode and cathode, i.e., electrodes to be dipped in the electrolyte. Four types of graphene quantum dots (GQDs) are prepared i.e., GQD1−GQD4. For GQD1−GQD4, the electrolyte used is a mixture of citric acid monohydrate (0.1 M) and NaOH (0.15M (GQD1), 0.2M (GQD2), 0.3M (GQD3) and 0.4M (GQD4)) in Milli-Q water (50 mL). Thereafter, the electrochemical experiments are done with CHI660D Electrochemical Workstation. The separation between the graphite rods is ca. 25 mm. Cyclic voltammetry (CV) is performed prior to performing chronoamperometry. CV is performed with a voltage range of −1 to +1 V, to wet the graphite electrodes. Thereafter, chronoamperometry is performed with a voltage of 10 V and a sensitivity (I/V) of 0.1 for 30 min. As a result, the color of the electrolyte solution changes from colorless to yellow, which confirms the exfoliation of graphite rod. After completion of this reaction, calcium chloride (0.15 M) is added to the prepared solution. This solution is slightly heated to precipitate calcium citrate. Centrifugation is performed for 15 min at 10 000 rpm twice to separate the calcium citrate precipitate. The supernatant is thereafter filtered through membrane filtration (Himedia, Dialysis Membrane-150, LA401, pore size ca. 2.4nm) for 7 days to remove salt from the solution.

Context 2

Author: Ashwin Kumar Narasimhan

Doi: 10.1039/c7ra10702a

Method:

Synthesis of Graphene Quantum Dots (GQDs): Here we used pure highly oriented pyrolytic graphite (HOPG) plate as a source immersed in 5 mL of polyethylene glycol and 5 mL Milli-Q watersolution (1 : 2). A focused nanosecond pulsed laserwas optically directed to the graphite plate. The laser source was operated at 1064 nm with specific parameters (energy = 40 mJ, pulse duration = 6 ns,frequency = 10 Hz). After 30 minutes of ablation on graphite plate (3 mm thickness), single wall graphene layers and reduced GQDs were produced in the PEG–water

solution. For characterization studies, the solution was centrifuged at 15000 rpm for 30 minutes to remove large particles and graphene layers. We denote this preparation as control GQDs. Furthermore, the control-GQDs solution was refluxed at 200°C for 20 minutes and 1 hour. After heating for 20 min and 1 h the water evaporates completely, and the final product contains pegylated GQDs. We denote the resulting preparations as 20 min and 1 h GQDs corresponding to refluxing times of 20 minutes and 1 hour respectively.

Context 3

Author: Ling-Jun Wang

Doi: 10.1063/1.3533021

Method:

The graphene flakes were produced by mechanical cleaving of graphite crystallites by Scotch tape and then were transferred to a highly doped Si substrate with a 100 nm SiO2 top layer. Thin flakes were found by optical microscopy, and single layer graphene flakes were selected by the Raman spectroscopy measurement. We used the standard electron beam lithography and lift off technique to make the Ohmic contact (Ti/Au) on the present graphene devices. Next, a new layer of poly(methyl methacrylate) is exposed by electron beam to form a designed pattern. Then, the unprotected areas are removed by oxygen reactive ion etching. The quantum dot is an isolated central island of diameter 90 nm, connected by 30 nm wide tunneling barriers to source and drain contacts. Here, the Si wafer was used as the back gate and there is also a graphene side gate near the small dot. The SET has a similar pattern while the conducting island has a much larger diameter (180 nm).

Context 4

Author: Ruquan Ye

Doi: 10.1038/ncomms3943

Method:

Synthesis of graphene quantum dots (GQDs) from coal: In a typical procedure, 300 mg of coal was suspended in concentrated sulphuric acid (60 ml) and nitric acid (20 ml), and followed by cup sonication (Cole Parmer, model 08849−00) for 2 h. The reaction was then stirred and heated in an oil bath at 100 or 120 °C for 24 h. The solution was cooled to room temperature and poured into a beaker containing 100 ml ice, followed by adding NaOH (3 M) until the pH was 7. The neutral mixture was then filtered through a 0.45-μm polytetrafluoroethylene membrane and the filtrate was dialyzed in 1,000 Da dialysis bag for 5 days. For the larger GQDs synthesized from anthracite (a-GQDs), the time can be shortened to 1 to 2 h using cross-flow ultrafiltration (Spectrum Labs, KrosFlo Research IIi TFF system with 3 kD cutoff membrane). After purification, the solution was concentrated using rotary evaporation to obtain solid GQDs.

Context 5

Author: Sha Li

Doi: 10.1038/s41598-025-96207-z

Method:

High-quality graphene was grown in a BM Pro 2×8” Chemical Vapour Deposition (CVD) furnace from Aixtron Ltd., using methane (CH4) as precursor and copper (Cu, 200 mm × 200 mm foil) as the catalyst. Semi-dry transfer was carried out using sacrificial polymeric support placed on the graphene surface and the Cu foil was etched away using ammonium persulfate (APS) etchant. After rinsing the bottom surface of the graphene with deionised (DI) water, it was dried and stamped on the target substrate using uniaxial pressure. Finally, the polymer was removed using solvents. Local back-gated graphene field-effect transistor (GFETs) were fabricated on 150 mm and 200 mm Si wafers with 90 nm silicon dioxide (SiO2) using standard CMOS-compatible photolithography technology. Firstly, the titanium/palladium (Ti/Pd) local back gate with a thickness of 5/40 nm was evaporated onto the substrate, followed by a standard metal lift-off process. A 75 nm thick Al2O3 layer was subsequently deposited via the atomic layer deposition (ALD) method to form the dielectric layer Afterward, the bottom contact of 30 nm Pd was evaporated. After transfer, graphene was patterned using reactive ion etching (RIE) by oxygen plasma and then a final layer of 40 nm Pd was evaporated onto the substrate for top contacting. The QDs were obtained from Quantum Solutions and had diameters on the order of 6–7 nm according to the data sheet. QD deposition was performed by spin-coating a total of 12 layers of PbS QDs via layer-by-layer ligand replacement in an inert atmosphere glovebox to minimize exposure to water and oxygen, followed by encapsulation with an aluminum oxide layer deposited via an ALD tool. The oleic acid ligands were replaced with ethanedithiol (EDT) or benzene dithiol (BDT) by in-situ ligand replacement. There are two different sizes of QDs with two different ligands, resulting in a 4-4-4 absorber-type arrangement (4 coatings of QDs with a 0.64 eV bandgap, another 4 coatings of QDs with a 0.64 eV bandgap, and finally, 4 coatings of QDs with a 1.25 eV bandgap), with an overall thickness on the order of 300 nm. The absorber layer was then removed everywhere except on the graphene channels of the GFETs, using lithographic patterning and a solution-based etching process (concentrated HCl and HI acid solutions). The devices were finally encapsulated with Al2O3 using an ALD process.

RAG-Gemini

Graphene quantum dots (GQDs) can be produced using various methods, including electrochemical exfoliation, laser ablation, and chemical oxidation from coal.

Here are the steps involved in each method:

1. Electrochemical Exfoliation from Graphite Rods:

* **Graphite Rod Preparation:** Graphite rods (3.05 mm diameter, ~60 mm length) are heated in a furnace at 1050°C for 5 minutes in air, then cooled to room temperature and washed with Milli-Q water to remove surface particles.

* **Electrolyte Preparation:** An electrolyte is prepared by mixing 0.1 M citric acid monohydrate with varying concentrations of NaOH (0.15M, 0.2M, 0.3M, or 0.4M) in 50 mL Milli-Q water.

*   **Electrochemical Setup:** The treated graphite rods are used as anode and cathode (electrodes) and dipped into the electrolyte with a separation of approximately 25 mm. An electrochemical workstation (CHI660D) is used for the process.

*   **Electrode Wetting:** Cyclic voltammetry (CV) is performed with a voltage range of -1 to +1 V to ensure the graphite electrodes are adequately wetted.

*   **GQD Exfoliation:** Chronoamperometry is then performed at a voltage of 10 V and a sensitivity (I/V) of 0.1 for 30 minutes. This process exfoliates the graphite, turning the colorless electrolyte solution yellow, which indicates GQD formation.

*   **Purification:**

    *   Calcium chloride (0.15 M) is added to the solution, which is then slightly heated to precipitate calcium citrate.

    *   The solution is centrifuged twice at 10,000 rpm for 15 minutes to separate the calcium citrate precipitate.

    *   The supernatant is subsequently filtered through a membrane (pore size ca. 2.4 nm) for 7 days to remove any remaining salts.

2. Laser Ablation from Highly Oriented Pyrolytic Graphite (HOPG):

*   **Graphite Immersion:** A pure highly oriented pyrolytic graphite (HOPG) plate (3 mm thickness) is immersed in a 1:2 solution of 5 mL polyethylene glycol and 5 mL Milli-Q water.

*   **Laser Ablation:** A focused nanosecond pulsed laser, operating at 1064 nm with specific parameters (energy = 40 mJ, pulse duration = 6 ns, frequency = 10 Hz), is optically directed onto the graphite plate for 30 minutes. This process ablates the graphite, producing single-wall graphene layers and reduced GQDs in the solution.

*   **Purification (Control GQDs):** The solution is centrifuged at 15,000 rpm for 30 minutes to remove larger particles and graphene layers, yielding "control GQDs."

*   **Optional Further Treatment (Pegylated GQDs):** For pegylated GQDs, the control-GQDs solution can be refluxed at 200°C for 20 minutes or 1 hour until the water completely evaporates.

3. Chemical Oxidation from Coal:

*   **Coal Suspension:** 300 mg of coal is suspended in a mixture of 60 mL concentrated sulphuric acid and 20 mL nitric acid.

*   **Sonication:** The suspension is sonicated using a cup sonicator for 2 hours.

*   **Heating:** The reaction mixture is then stirred and heated in an oil bath at 100 or 120°C for 24 hours.

*   **Neutralization:** After heating, the solution is cooled to room temperature, poured into 100 mL of ice, and then 3 M NaOH is added until the pH reaches 7.

*   **Purification:**

    *   The neutral mixture is filtered through a 0.45-μm polytetrafluoroethylene membrane.

* The filtrate is then dialyzed in a 1,000 Da dialysis bag for 5 days.

* (Alternative for larger GQDs): For larger GQDs synthesized from anthracite, the purification time can be shortened to 1-2 hours using cross-flow ultrafiltration with a 3 kD cutoff membrane.

* **Concentration:** The purified GQD solution is concentrated using rotary evaporation to obtain solid GQDs.

Ground truth

Graphene Quantum Dots (GQDs) can be produced using hydrothermail reactions, laser ablation, microwave radiation and electrochemical oxidation. THe most straightforward is that of electrochemical oxidation. The electrochemical oxidation process involves the electrochemical cleavage of carbon precursores such as graphite into GQDs, typically under high voltage. First a potential is applied to a graphite electrode immersed in an electrolyte made of citric acid and sodium hydroxide. A second graphite rod is used as an cathode in the same solution. A DC power supply can provide the working bias voltage. The GQDs eventually exfoliate and dispersed into the electrolyte. The resulting GQDs can be purified by centrifugation and further techniques such as rotary evaporation.

Question 12

What are the steps involved to produce graphene nanoribbon?

Context 1

Author: César Moreno

Doi: 10.1126/science.aar2009

Method:

Our method relies on the hierarchical control of three thermally activated reaction steps, labeled T1 to T3. Nanoribbons and pores with nanometer size, atomic-scale uniformity, and long-range order are formed in separate steps. Graphene nanoribbons (GNRs) are first synthesized by following a previously used route, consisting of the surface-assisted Ullmann coupling of aromatic dihalide monomers into polymer chains (T1) and the cyclodehydrogenative aromatization of the intermediate polymeric chains into GNRs (T2). The final step (T3) interconnects GNRs laterally in a reproducible manner by means of a highly selective dehydrogenative cross-coupling. This step requires a careful design of the monomer precursor, which defines the edge topology of the resulting GNR that is necessary for a high yield and selectivity of the cross-coupling reaction. The monomer precursor synthesized in this work, labeled DP-DBBA (diphenyl–10,10′-dibromo-9,9′-bianthracene), is a derivative of theDBBA used in the synthesis of seven–carbon atom–wide armchair GNRs (7-AGNR), with phenyl substituents added at (2,2′) sites. The latter is the key element for the promotion of the inter-GNR connections that lead to the NPG structure. The choice of catalytic surface is also relevant for the selection of the reaction paths that define the intermediates and for the separation of thermal windows that lead to their hierarchical control. Here we use the Au(111) surface, where each reaction step has a different thermal activation onset. The NPG can then be transferred to suitable substrates in which its functionalities can be exploited.

Context 2

Author: Junan Fang

Doi: 10.1016/j.nanoms.2025.02.010

Method:

Preparation of cove-type graphene nanoribbon (cGNRs-Dipyrene:) In a dried 500 mL flask add 500 mg of Poly-Dipyrene and 300 mL of anhydrous dichloromethane. Under nitrogen protection, degas the mixture using a dry diaphragm pump three times for approximately 10 min. Dissolve 9.94 g of anhydrous $FeCl3$ in 15 mL of anhydrous $CH3NO2$ (acetonitrile), degas it, and drip it into the Poly-Dipyrene solution under a nitrogen atmosphere while stirring. Maintain stirring and pass nitrogen gas continuously through the solution at ambient temperature for 3 d. Once the reaction is done, most of the dichloromethane is removed at amlower temperature. Add 400 mL of methanol, stir for 10 min, and allow it to rest for 6 h. Decant the supernatant and collect the cloudy lower phase for spinning in a centrifuge. Obtain the black precipitate and perform extraction using the Soxhlet method with methanol. The

collected black solid is the final product compound, cGNRs-Dipyrene, with a total weight of 480 mg and a yield of 94 %.

Context 3

Author: Young Duck Kim

Doi: 10.1038/nnano.2015.118

Method:

(1) Mechanically exfoliated graphene was prepared on a SiO_2/Si substrate. (2) A PMMA (Polymethyl methacrylate, 950K C4) was spin coated on graphene at 4500 rpm followed by baking process at 180°C for 5 minutes. (3-4) To make a patterned graphene array, PMMA on unwanted areas of graphene was exposed by e-beam lithography, and the remaining PMMA after development acted as an etch mask during O_2 plasma etching. (5) Patterned graphene array was prepared after removing PMMA with acetone. (6) PMMA was spin coated again on the patterned graphene ribbons using the same recipe as in step (2). (7) PMMA membrane with graphene ribbons was separated from SiO_2/Si substrate in 10% (wt) potassium hydroxide water solution (KOH). (8) The separated PMMA membrane with attached graphene was rinsed with DI-water to remove the KOH residue from the graphene surface and dried at room temperature in Nitrogen atmosphere. (9) The position of the PMMA membrane with patterned graphene arrays was manipulated on prepared trench substrate (depth: 300 ~ 1100 nm) using home-made micro-position aligner. (10) Using micro contact transfer method, each side of the graphene ribbons were attached to the Au electrodes of the prepared trench. (11) The PMMA layer was removed by an acetone wash followed by an IPA (Isopropanol) rinse. The suspended ME graphene devices are completed after a critical point drying process.

Context 4

Author: Xiaoyin Yang

Doi: 10.1021/ja710234t

Method:

Compound 1: Magnesium turnings (3.6 g, 150 mmol) were placed in an Ar-flushed flask and dry THF (10 mL) was added. 1,2-Dibromoethane (0.2 mL) was added and the resulting mixture was stirred at room temperature (rt) for a few minutes. After the reaction ceasing, the solution was removed via cannulation and dry THF (100 mL) was added. Then, a solution of 1-bromo-4-(3,7-dimethyloctyl)benzene (22 g, 100 mmol) was slowly added at room temperature. The reaction starts within a few minutes. After addition, the reaction mixture was stirred for 12 h at room temperature. The grey solution of 4-(3,7- dimethyloctyl)phenyl magnesium bromide was then cannulated into a suspension of 1,4-dichloro-2,3,5,6- tetrabromobenzene (4.6 g, 10 mmol) in dry THF (20 mL) under argon and the resulting mixture was stirred an rt for 12 h. I_2 (25 g, 100 mmol) was added directly to the reaction mixture at 0°C and the reaction was stirred at rt under argon for 2 h. The reaction was quenched with water and the resulting mixture was extracted with $CHCl_3$ (100 mL × 3). The combined organic layers were washed with 2M aqueous $NaHSO_3$ solution (200 mL × 2) and brine (50 mL), dried with $MgSO_4$ and concentrated in vacuum. The residue was purified with chromatography (Silica gel, n-

Hex./CH2Cl2 = 20/1) to give 7.7 g of compound 1 as a thick oil. The yield is 65%. Compound 2: A suspension of compound 1 (1.2 g, 1.0 mmol), 4-bromophenylboronic acid (800 mg, 4.0 mmol), K2CO3 (2.76 g, 20 mmol) and Aliquat ® 336 (8 mg, 0.02 mmol) in toluene (30 mL) was degassed three times via 'freeze-pump-thaw' cycles. Pd(PPh3)4 (58 mg, 0.05 mmol) was added quickly and the reaction mixture was degassed three times again via 'freeze-pump-thaw' cycles. Then the reaction was allowed to be stirred at 80°C under argon for 18 h. The reaction was quenched with water (20 mL) and the resulting mixture was extracted with dichloromethane (30 mL × 3). The combined organic layers were washed with brine (30 mL), dried with MgSO4 and concentrated in vacuum. The residue was purified by column (Silica gel, nHex./CH2Cl2 = 20/1) to give compound 2 as a white solid (1.17 g, Yield = 93). Compound 3: To a solution of compound 2 (600 mg, 0.48 mmol) in dry THF (8 mL) under argon at -78 °C, n-BuLi(1.6 M/n-Hexane, 0.78 mL, 1.24 mmol) was added and the resulting solution was stirred at – 78 o C for 1 h. Then 2-isopropoxy-4,4,5,5-tetramethyl-[1,3,2]dioxaborolane (267 mg, 1.44 mmol) was added at -78 °C and the reaction was allowed to be stirred at rt for 3 h. The reaction was quenched with water (5 mL) and the resulting mixture was extracted with dichloromethane (15 mL × 3). The combined organic layers were washed with brine (10 mL), dried with MgSO4 and concentrated in vacuum. The residue was purified by column (Silica gel, n-Hex./CH2Cl2 = 4/1) to give compound 3 as a thick oil (530 mg, Yield = 82%). Polymer 4: A mixture of compound 1 (89 mg, 0.074 mmol), compound 3 (100 mg, 0.074 mmol), K2CO3 (2 M/H2O, 2 mL, 4 mmol) and Aliquat® 336 (0.6 mg, 0.0015 mmol) in toluene (5 mL) was degassed three times via 'freeze-pump-thaw' cycles. Pd(PPh3)4 (4.3 mg, 0.004 mmol) was added quickly and the reaction mixture was degassed three times again via 'freeze-pump-thaw' cycles. The reaction was refluxed under argon for 3 days. Then, a degassed solution of bromobenzene (15 mg, 0.1 mmol) in toluene (0.5 mL) was added into the reaction mixture via a syringe. After refluxing for 12 h, a degassed solution of phenylboronic acid (12 mg, 0.1 mmol) in toluene (0.5 mL) was added into the reaction mixture via a syringe and the reaction was refluxed for another 12 h. The reaction mixture was poured into a mixture of methanol (200 mL) and con. aqueous HCl solution (30 mL) and stirred overnight. The resulting black solid was filtered off and subjected to Soxhlet extraction for 2 days in acetone. The residue was redissolved in hot THF and precipitated again in methanol. The solid was filtered, washed with methanol and dried in vacuum to give polymer 4 as a grey solid (112 mg, Yield = 75%). Graphene Nanoribbon 5: A suspension of polymer 4 (10 mg) in freshly distilled CH2Cl2 (200 mL) was purged with argon for 15 mins. Then, a solution of FeCl3 (114 mg, 0.7 mmol) in CH3NO2 (0.3 mL) was added dropwise and the resulting black solution was stirred at rt for 12 h with bubbling of argon. Stopped the argon bubbling and the reaction mixture was stirred at rt for 36 h. The solvent was removed under argon and THF (1 mL) was added to redissolved the reaction mixture. The resulting solution was dropped into a mixture of methanol (100 mL) and con. aqueous HCl solution (30 mL) and stirred overnight. The resulting black solid was filtered off and subjected to Soxhlet extraction for 2 days in acetone. The residue was redissolved in hot THF and precipitated again in methanol. The solid was filtered, washed with methanol and dried in vacuum to give polymer 5 as a black solid (6.4 mg, Yield = 65%).

Context 5

Author: Néstor Merino-Díez

Doi: 10.1021/acs.jpclett.8b00796

Method:

This is demonstrated using 4,4”-dibromoterphenyl (DBTP) as a molecular precursor and two different gold surfaces as substrate, namely Au(111) and Au(322). DBTP molecules adsorbed on Au surfaces are known to undergo a series of chemical reactions upon annealing. In our experiments, the Au(111) and Au(322) surfaces were cleaned similarly, by standard Ar^+ sputtering and annealing cycles. The molecules were then deposited on the surfaces by means of a home-built Knudsen cell heated to ∼115 °C during sublimation. During the deposition, the surfaces were held at room temperature and annealed thereafter to subsequently trigger the different reaction steps. We then anneal the samples to 180°C to trigger the polymerization of DBTP into poly paraphenylene (PPP) through Ullmann coupling, whereby single Br atoms are left on the surface as byproducts, typically forming rows sandwiched between the PPP chains. Further increasing the temperature, the Br atoms desorb from the substrate, allowing the PPP chains to approach each other and fuse as the cyclodehydrogenation is activated at temperatures around 380 °C. As a result, wider armchair-oriented graphene nanoribbons (aGNRs) form, whose width is determined by the number of participant PPP wires. Quantified by the number of dimer lines across the aGNR , the resultant widths thus correspond to multiples of three (3naGNR, n being the number of fused PPP). Since aGNRs are classified into three families depending on their number of dimer lines ($3p - 1$, $3p$, and $3p + 1$, p being an integer), all nanoribbons synthesized in these experiments thus correspond to the same 3p family.

RAG-Gemini

The production of graphene nanoribbons (GNRs) typically involves a multi-step process, often categorized as bottom-up chemical synthesis from molecular precursors. While specific precursors and conditions vary, common steps include:

1. **Precursor Design and Synthesis:** The initial step involves the careful design and synthesis of specific aromatic molecular monomers. These monomers are engineered to contain the necessary atoms and functional groups that will dictate the width, edge topology, and overall structure of the resulting GNR. For instance, diaryl dihalide monomers or more complex polycyclic aromatic precursors are commonly used.

2. **Polymerization:**

  * **Surface-Assisted Polymerization:** In this method, the designed monomers are deposited onto a catalytic surface, such as Au(111). Upon thermal annealing (e.g., at 180°C), the monomers undergo a coupling reaction, such as Ullmann coupling, to form extended linear polymer chains.

  * **Solution-Based Polymerization:** Alternatively, in solution-based approaches, the monomers are reacted in a solvent, often via coupling reactions like Suzuki coupling, to form the desired linear polymer backbone.

3. **Cyclodehydrogenation (Aromatization):** This is the crucial step where the linear polymer chains are converted into the final GNR structure through intramolecular cyclization and hydrogen elimination.

*   **Surface-Assisted Cyclodehydrogenation:** For surface-assisted synthesis, further thermal annealing at higher temperatures (e.g., around 380°C) activates the cyclodehydrogenation. This causes the polymer chains to fuse laterally and aromatize, forming the extended sp2-hybridized graphene lattice of the nanoribbon.

*   **Solution-Based Cyclodehydrogenation:** In solution, a chemical oxidant (e.g., anhydrous FeCl3) is introduced to the polymer solution. This initiates the oxidative cyclodehydrogenation process, leading to the formation of GNRs.

4. **Purification and Isolation:** After the synthesis, the GNRs are typically subjected to purification steps to remove unreacted precursors, byproducts, and catalyst residues. This can involve techniques such as solvent removal, precipitation, centrifugation, and Soxhlet extraction.

5. **Transfer (for Surface-Assisted Synthesis):** If GNRs are synthesized on a surface, they may need to be subsequently transferred to a different substrate for further characterization or device integration.

Ground truth

Graphene nanoribbons (GNRs) are narrow strips of single-layer graphene, essentially 'slices' of graphene that are one-dimensional. They have very high surface areas. They can be synthesized either by top-down approaches such as micromechanical cleavage or bottom-up approaches such as CVD. Top down approaches usually use multi-walled Carbon nanotubes (MWCNTs) to form these graphene nanoribbons. For instance usuing the hummers method on MWCNTs to form the ribbons instead of bulk graphite. Bottom up-approaches using CVD have highly-controlled conditions for growth to ensure orientation and length.

Question 13

What is a method of exfoliating graphene from highly oriented pyrolytic graphite (HOPG) crystal?

Context 1

Author: Yuan Huang

Doi: 10.1021/acsnano.5b04258

Method:

We used SiO2/Si as the substrate and ordinary adhesive tape as the transfer medium. Contact with the tape transfers thick graphite flakes from a highly oriented pyrolytic graphite (HOPG) crystal. Prior to exfoliating thin graphene from these flakes, the SiO2/Si substrate is ultrasonically cleaned in acetone, 2-propanol, and deionized (DI) water, and then subjected to oxygen plasma to remove ambient adsorbates from its surface. Following the plasma cleaning step, the graphite-loaded tape is brought in contact with the substrate. Instead of immediately removing it to complete the exfoliation, the substrate with the attached tape is annealed for 25 min at~100°C, in air on a conventional laboratory hot plate. After the sample is cooled to room temperature, the adhesive tape is removed, which completes the exfoliation.

Context 2

Author: Dan Li

Doi: 10.1038/nnano.2007.451

Method:

Graphite oxide was synthesized from natural graphite (SP-1, Bay Carbon) by a modified Hummers method as originally presented by Kovtyukhova and colleagues. As-synthesized graphite oxide was suspended in water to give a brown dispersion, which was subjected to dialysis to completely remove residual salts and acids. Ultrapure Milli-Q® water was used in all experiments. As-purified graphite oxide suspensions were then dispersed in water to create a 0.05 wt% dispersion. Exfoliation of graphite oxide to graphene oxide (GO) was achieved by ultrasonication of the dispersion using a Brandson Digital Sonifier (S450D, 500 W, 30% amplitude) for 30 min. The obtained brown dispersion was then subjected to 30 min of centrifugation at 3,000 r.p.m. to remove any unexfoliated graphite oxide (usually present in a very small amount) using an Eppendorf 5702 centrifuge with a rotor radius of 14 cm. In a typical procedure for chemical conversion of graphite oxide to graphene, the resulting homogeneous dispersion (5.0 ml) was mixed with 5.0 ml of water, 5.0 µl of hydrazine solution (35 wt% in water, Aldrich) and 35.0 µl of ammonia solution (28 wt% in water, Crown Scientific) in a 20-ml glass vial. The weight ratio of hydrazine to GO was about 7:10. After being vigorously shaken or stirred for a few minutes, the vial was put in a water bath (~95 °C) for 1 h. Unless specifically stated, graphene dispersions prepared according to the above

procedure were used for further characterization and film fabrication in this work. The graphene paper was made by filtration of a dispersion through an Anodisc membrane filter (47 mm in diameter, 0.2 µm pore size; Whatman), similar to the method reported for making GO paper. The graphene paper was cut by a razor blade into rectangular strips of approximately 4 mm × 15 mm for mechanical testing.

Context 3

Author: Vincent Huc

Doi: 10.1088/0957-4484/20/17/179801

Method:

The proposed method is somewhat reminiscent of the process developed for the industrial production of thin silicon-on-insulator wafers . A thin “layered” crystalline film is bonded and firmly mechanically supported during the thinning. Our process mainly consists of two steps: Firstly, graphite bonding is realized using a thin layer of epoxy resist which is subsequently cured under pressure. Secondly the bonded graphite is exfoliated using the scotch tape technique. A freshly cleaved highly oriented pyrolytic graphite crystal is glued using very thin epoxy-based glue. After curing, the glue leaves creates a transparent, void and bubble-free interlayer between the substrate and the highly oriented pyrolytic graphite sample. This fabrication method can be refined by using a pre-cut (<100µm thick) flexible highly oriented pyrolytic graphite sample as the top layer. This method may appear as a refinement of the exfoliation process known as the “scotch-tape” technique. However significant differences do exist with respect to that well-known method. Firsly one should note that the useful graphene sheets is the one left on the adhesive side (i.e. the epoxy underlayer) after the curing step (hence the name “reverse exfoliation”). This leads to larger and flatter flakes with very few pleats. The second one is that a uniaxial compression is applied to the sample during the adhesive curing step, in order to increase the flatness of the adhesive interlayer. The third one is that the adhesive used in this study was found to be compatible with conventional micro/nanofabrication techniques including lithographic and etching processes. This allows transport measurements to be performed in-situ directly on the bonded monolayer.

Context 4

Author: G. Rajendra Kumar

Doi: 10.1039/C5RA24810H

Method:

In a typical procedure, 50 g of graphite flakes and 80 g of oxalic acid (OA) were placed into a stainless steel jar (2500 ml volume) containing 10 mm diameter stainless steel balls, which were used as the grinding medium. Low diameter stainless steel balls were utilized to increase the impact and shear energy between the balls and powder, and to have a low dimensional interaction with the graphite layers. Initially, the dual-drive ball milling was executed at three different critical speeds (40%, 70%, and 98%), but the critical speed of 40% offers efficient exfoliation with less defects than 70% and 98%. Accordingly, the optimized critical speed of 40% was adopted for graphite exfoliation using shear force ball milling. The milling was carried

out for up to 20 h and the rest time was one hour per cycle. Then, the milled graphite was treated with 1 M hydrochloric acid (HCl) to acidify the carboxylates and to remove the iron impurities from the milling jar. Then, the mixture was washed with distilled water several times until the pH was neutral. The mixture was filtered and subjected to heat treatment at 600 °C under an argon atmosphere for an appropriate time interval. Finally, as-prepared graphene was stored for future use.

Context 5

Author: Arjunan Ariharan

Doi: 10.4236/graphene.2016.52005

Method:

Graphene Oxide (GO) was synthesized from graphite by a modified Hummers method. Briefly, graphite powder (2.0 g) was taken in a beaker, added 50 m of concentrated sulfuric acid (H2SO4) under vigorous agitation in an ice bath. Afterwards, sodium nitrate (2.0 g) and potassium permanganate (6.0 g) were slowly added in a sequence. Then, the mixture was transferred into a water bath and kept at 35˚C for 2 hour. After that, 100 mL of distilled water was slowly added, causing a temperature rise to 98˚C. Later, 140 mL of 4% H2O2 was dropped into the reaction system. Finally, the product was washed with distilled water three times. The acquired solid was dried in vacuum at 50˚C for 48 hours.

RAG-Gemini

Graphene can be exfoliated from highly oriented pyrolytic graphite (HOPG) crystals using mechanical exfoliation methods.

One common approach involves:

1. Preparing a SiO2/Si substrate by ultrasonic cleaning in acetone, 2-propanol, and deionized water, followed by oxygen plasma treatment to remove ambient adsorbates.

2. Transferring thick graphite flakes from an HOPG crystal onto ordinary adhesive tape.

3. Bringing the graphite-loaded tape into contact with the cleaned SiO2/Si substrate.

4. Annealing the substrate with the attached tape for approximately 25 minutes at 100°C in air.

5. After cooling to room temperature, the adhesive tape is removed, which completes the exfoliation of thin graphene onto the substrate.

Another refined method, sometimes referred to as "reverse exfoliation," uses an epoxy-based glue:

1. A freshly cleaved HOPG crystal is glued using a thin layer of epoxy-based glue.

2. The glue is cured under pressure, creating a transparent, void- and bubble-free interlayer between the HOPG and a substrate.

3. The bonded graphite is then exfoliated using a technique similar to the scotch tape method. In this variation, the useful graphene sheets are typically left on the adhesive side (the epoxy underlayer) after the curing step, leading to larger and flatter flakes. Uniaxial compression can be applied during the adhesive curing to enhance the flatness of the adhesive interlayer.

Ground truth

HOPG is essentially highly oriented graphite which is ideal for producing graphene using mechanical cleavage with adhesive tape. HOPG can be purchased commercially and then exfoliated through existing processes such as micromechanical exfoliation tape to reult in high-quality graphene with minimal defects.

Question 14

How to synthesize graphene using PET bottles?

Context 1

Author: Aamir Ahmed

Doi: 10.1016/j.mseb.2024.117828

Method:

Synthesis of carbon: The bubble wraps were washed 5-times with DI water. After drying, these sheets were cut into tiny pieces and put into a petri dish. The dish was then heated at 100 °C to melt the pieces followed by solidifying. These solidified sheets were chopped into small pieces and immersed in a beaker with concentrated H2SO4. The beaker containing the sample was transferred to a muffle furnace and subjected to thermal treatment at 150 °C for a duration of 3 h. The excess sulphuric acid was drained out and the resultant was a black hard mass. The acquired solid was subjected to repeated washing with deionized water until a neutral pH was attained. This sulphonation step was followed by carbonization. The centrifuged sample was heated in a muffle furnace at 500 °C for 1 h. The sample then obtained was washed with DI water, dried at room temperature, and reduced to powder using mortal and pestle. Conversion of carbon into reduced graphene oxide (rGO): In this process, 1 g synthesized carbon and 1 g NaNO3 were added to 45 ml of H2SO4 (kept in an ice bath) and stirred for 30 min until a homogenous black solution is produced. To this solution, 5 g KMnO4 was slowly added and stirred at 50 °C for 2 h forming a green solution. 100 ml DI water and 6 ml H2O2 (35 %) were added drop-by-drop to stop the reaction. The precipitates were washed with 300 ml HCl (10 %) and 200 ml HCl (37 %) in a centrifuge. The final sample was heated at 250 °C in a muffle furnace, again washed with DI water, and reduced to rGO powder.

Context 2

Author: Jian-Hao Chen

Doi: 10.1002/adma.200701059

Method:

First, photolithography is used to prepare 30 nm thick Au source and drain electrodes on a silicon wafer with an oxidized surface (SiO2/Si). The electrodes are then transferred onto the Polyethylene Terephthalate (PET) substrate as described elsewhere. Then, single-and few-layer graphene is obtained from Kish graphite by mechanical exfoliation on 300 nm thick thermally grown silicon dioxide on silicon substrates, and its thickness and morphology characterized by atomic force microscopy. Mechanical exfoliation yields atomically clean graphene sheets and our AFM images also indicate that the graphene sheet is free of nanometer-scale contaminants. In addition, chemical contamination caused by exposure to photoresist and lift-off chemicals is avoided in this process. The desired graphene sheet is printed at 170°C at 500 psi from the SiO2/Si substrate to the source-drain electrode assembly on PET.

Context 3

Author: Subash Sharma

Doi: 10.1016/j.carbon.2014.01.051

Method:

A horizontal ambient pressure (AP) chemical vapour deposition (CVD) system with a quartz tube of length 90 cm and diameter of 50 mm was taken as the reactor placed in two split furnaces. Cu foil (Nilaco Corp.) of thickness 20 μm and purity 99.99% was used as substrate for graphene synthesis. Solid waste plastic used for material packaging is used as carbon source in these studies. Cu foil was cleaned by sonicating in acetone for 30 min and placed in the growth furnace without further treatment. Cu substrate was annealed for 30 min in 100 standard cubic centimeters per minute (sccm) of H_2 at 1020 °C before the feedstock was introduced. Annealing is an important step to enlarge Cu grain size, reduce lattice mismatch and dislocations, which help to limit number of nucleation sites. 3 mg of waste plastic as carbon source was placed in a ceramic boat inside the lower temperature furnace. Graphene growth was carried out for 90 minutes using a gas mixture of Ar and H_2 with the flow rate 98 and 2.5 sccm, respectively. During the growth process, the rate of increase in pyrolysis temperature for the waste plastic feedstock was optimized as 1.5 °C/min and the pyrolysis temperature was increased gradually to around 480 °C until all the precursor was evaporated. Supply of precursor was continuously increased during the growth process to provide sufficient carbon atoms for uninterrupted growth of graphene crystal. The injection rate of polymeric components contained in the waste plastic was changed by the rate of evaporation. Thereafter, it was allowed to cool at a cooling rate of (~16°C/min). Individual graphene crystal was transferred to SiO_2/Si substrate by coating a poly(methyl methacrylate) (PMMA) supporting layer. Etching of underneath Cu foil was achieved by using a 50 mg/ml concentrated $Fe(NO_3)_3$ solution. Subsequently, PMMA/graphene stack layer was transferred to SiO_2/Si substrate and PMMA coating was dissolved in acetone. Finally, transferred graphene was treated with a diluted nitric acid solution to remove the residual $Fe(NO_3)_3$ and dried in air.

Context 4

Author: Mohammad Choucair

Doi: 10.1038/nnano.2008.365

Method:

All solvothermal reactions were performed in a Teflon-lined Parr Instrument Company 4749 reactor having a maximum volume of 23 ml. A typical synthesis consists of heating a 1:1 molar ratio of sodium (2 g) and ethanol (5 ml) in a sealed reactor vessel at 220 °C for 72 h to yield the solid solvothermal product—the graphene precursor. This material is then rapidly pyrolysed, and the remaining product washed with deionized water (100 ml). The suspended solid is then vacuum filtered and dried in a vacuum oven at 100 °C for 24 h. The final yield of graphene is approximately 0.1 g per 1 ml of ethanol—typically yielding ∼0.5 g per solvothermal reaction.

Context 5

Author: Noha A. El Essawy

Doi: 10.1016/j.ecoenv.2017.07.014

Method:

First, waste PET bottles were crushed and sieved to obtain the desired size fractions (1–3 mm) using a conventional sieve-shaker. Then 2 g of raw PET waste was introduced into an enclosed 50 mL stainless steel autoclave reactor (SS316). The closed stainless steel reactor was placed inside the center of an electric furnace and heated to 800 °C with a rate of 8 °C/min and maintained at this temperature for 1 h. After that the system was left to cool overnight. The resulted dark products were collected and crushed

RAG-Gemini

Graphene can be synthesized from PET bottles using a couple of methods:

1. **Pyrolysis:**

   *   Waste PET bottles are crushed and sieved to obtain desired size fractions (e.g., 1–3 mm).

   *   The PET waste is then introduced into an enclosed stainless steel autoclave reactor.

   *   The reactor is heated to 800 °C at a rate of 8 °C/min and maintained at this temperature for 1 hour.

   *   After the heating period, the system is left to cool down overnight.

   *   The resulting dark products are collected and crushed.

2. **Chemical Vapor Deposition (CVD):**

   *   Waste plastic (such as that used for packaging, which can include PET) serves as the carbon source in a horizontal ambient pressure CVD system.

   *   A copper foil (e.g., 20 μm thick, 99.99% purity) is used as the substrate, first cleaned by sonicating in acetone.

   *   The copper substrate is annealed for 30 minutes in hydrogen (100 sccm) at 1020 °C to enlarge grain size and reduce nucleation sites.

   *   The waste plastic (e.g., 3 mg) is placed in a ceramic boat within a lower temperature furnace.

   *   Graphene growth is carried out for 90 minutes using a gas mixture of argon (98 sccm) and hydrogen (2.5 sccm).

* During growth, the pyrolysis temperature for the waste plastic feedstock is gradually increased at an optimized rate (e.g., 1.5 °C/min) to around 480 °C until all the precursor evaporates, ensuring a continuous supply of carbon atoms.

* After growth, the system is cooled at a rate of approximately 16°C/min.

* The synthesized graphene is then transferred to a target substrate (e.g., SiO2/Si) by coating with a poly(methyl methacrylate) (PMMA) supporting layer, etching the copper foil using a ferric nitrate solution, transferring the PMMA/graphene stack, dissolving the PMMA in acetone, and finally treating with a diluted nitric acid solution to remove residuals.

Ground truth

Waste PET can be cleaned and then used as a precursor for pyrolysis into graphene. It usually takes a 2 step process. First, pyrolysis at 900 degrees centigrade into amorphous carbon. Next, the amorphous carbon is mixed with Boron powder and heat treated at 2400 degrees celsius for 1 hour under helium gas to form graphite. This high-temperature treatment with boron enables the carbon atoms to rearrange into the layered structure of graphite.. Yield rates are about 86%. This graphite can then be liquid-phase exfoliated to form graphene.

Question 15

What is the purpose of PMMA in the synthesis of graphene?

Context 1

Author: Cristina Vallés

Doi: 10.1016/j.carbon.2019.10.075

Method:

Synthesis of NH2 terminated graphene nanoplatelets (NH2-GNPs): NH2-GNPs were synthesized by a nucleophilic substitution reaction through a spontaneous diazonium coupling reaction. In a typical reaction, 150 mg of graphene nanoplatelets (GNP-M25 from XG Sciences, with lateral dimension and thickness of ~25 μm and ~6 nm, respectively, quoted by the manufacturer) was initially suspended in 125 ml of acetonitrile (CH3CN) together with 1.5 g of p-phenylene diamine and heated up to 60 °C under mechanical stirring. Once this temperature was reached, 2 mL of isoamylnitrite were added to the mixture and left to react under mechanical stirring for 24h. The resultant solid was then separated by vacuum filtration, rinsed first three times with CH3CN and then three times with EtOH and left to dry at room temperature. This material was labelled as NH2-GNPs. Synthesis of PMMA grafted graphene nanoplatelets (PMMA-NH-GNPs): NH2-GNPs and PMMA powders (50/85 mg) were dispersed together in chloroform (CHCl3) and the mixture was stirred at 70 °C for 24 h. After that time, the reaction mixture was cooled down to room temperature, filtered to collect the resultant powder, washed with 50 mL of CHCl3 and dried. This material was labelled as PMMA-NH-GNPs. (PMMA-NH-GNPs/PMMA) composite preparation: The appropriate amount of filler (PMMA-NH-GNPs or GNPs) and 1 g of PMMA were dissolved in 15 mL of CHCl3 using mechanical stirring at room temperature for 30 minutes to prepare composite materials (PMMA-NH-GNPs/PMMA or GNPs/PMMA) at various loadings from 0.5 to 5 wt.%. Composite films were prepared by depositing these dispersions on a Teflon dish using the solvent-casting method, followed by complete removal of the solvent and peeling-off of the composite films. Specimens with the desired shapes and sizes were cut out of these composite films for characterization.

Context 2

Author: Wei Wu

Doi: 10.1016/j.snb.2010.06.070

Method:

Briefly, the procedure is described as follows. Graphene was grown by thermal chemical vapour deposition (CVD) on a Cu foil, at a temperature of 1000°C and under 1 atm pressure with methane as the precursor gas. A quartz tube with 2 diameter was used as the reaction chamber for our CVD system. Cu foils were rolled up in a roll, but without the rolled-up layers touching to each other. Following the growth, poly(methyl methacrylate) (PMMA) was spun on graphene/Cu substrate to form PMMA/graphene/copper sandwich structure. Later, Cu foil was

etched away using an iron nitrate aqua solution. After the Cu foil was completely etched away, graphene with PMMA/graphene film was transferred onto a Si wafer with 300 nm thermally grown SiO2. The PMMA was then removed by repeatedly rinsing the film in acetone.

Context 3

Author: F.S. Al-Hazmi

Doi: 10.1016/j.synthmet.2014.12.028

Method:

5 g of graphite flakes (thickness ~0.1 μm) was first immersed into 30 ml of ice cooled glutaric acid over night. The pretreated graphite flakes were filtered and left to dry at ambient conditions. The pretreated graphite subjected to a thermal shock at 700 °C for 1 min to expand the graphite. 500 mg of the expanded graphite flakes were dispersed in 50 ml methanol and sonicated for 10 min to ensure the complete dispersion of pretreated graphite in the methanol solution. The graphite solution was introduced into a Teflon autoclave with capacity 80 cm3 and put into microwave oven at power of 800 W and temperature 200 °C for 30 min. After this time, the reaction was terminated by put the Teflon autoclave into ice cooled bath. The supernatant was collected by centrifuging and washed several times with deionized water and HCl (10%). The graphene sheets were dried under vacuum over night at 70 °C.

Context 4

Author: Xin Zhao

Doi: 10.1039/C9NR04720D

Method:

The raw graphite single flakes used to prepare the mechanically-cleaved monolayer graphene were purchased from Germany. The thermal release tape used to exfoliate graphite into graphene was supplied by Japan. Poly(methyl methacrylate) (PMMA) plates were purchased from UK and were subsequently cut into small beams of 70 mm in length and 20 mm in width, followed by polishing at the cut edges to minimize scratches and defects. This procedure allows the PMMA beams to be deformed to high strains (~2%) without fracture. Preparation of exfoliated monolayer graphene on PMMA: For the deformation and fracture studies of monolayer graphene, the monolayer graphene flakes were prepared by mechanical cleavage of single flake graphite. The graphite flake was initially placed in the middle of the Nitto tape and repeatedly peeled with another layer of tape. At the end of this procedure, the material that remained on the tape was a mixture of multilayer graphene flakes of different thicknesses and lateral dimensions. By repeated peeling, the multilayer graphene was finally cleaved into thin graphene sheets. The tape covered with different layers of graphene was subsequently pressed onto the PMMA beam. As a result, graphene flakes with different thickness and lateral dimensions were obtained on the PMMA beam.

Context 5

Author: Qi Chen

Doi: 10.1038/srep02315

Method:

The average diameter of poly(methly methacrylate) (PMMA) spheres used here is 300 nm. The cationic polyelectrolyte (PEI) is purchased from Alfa Aesar. Graphene Oxide (GO) nanosheets are prepared using a modified Hummer's method from graphite powders. The obtained GO nanosheets are dispersed in deionized water by ultrasonic treatment prior to being used in the experiment. The reference sample of graphene nanosheets is purchased from a commercial source (ACS Material) with purity of ~99.8% and a single-layer ratio of ~ 80%. To prepare reduced Graphene Oxide (rGO) nanospheres, we follow a previous procedure. We modify the surface of PMMA spherical templates with disperser of PEI in aqueous solution. PMMA spheres (5 mg/mL) are dispersed in deionized water containing PEI (2.5 mg/mL) at pH 9.0 under stirring to introduce positive charges at the surface of PMMA spheres. The pH value in the solution is adjusted by dilute HCl or ammonia. After 10 minutes ultrasonic treatment, the suspension is stirred for another 15 minutes to ensure saturated absorption of PEI. To remove the excess PEI, the suspension is further treated by two cycles of centrifugation (8000 rpm at 17% for 5 min) and washing. The PEI-coated PMMA spheres are then dispersed in deionized water and ultrasonically treated for another 10 min. We add a portion of the colloidal suspension of negatively charged GO nanosheets to the turbid PMMA suspension and keep stirring until the supernatant is almost transparent. The sediments are the products owing to the electrostatic interaction of the oppositely charged nanosheets and PMMA surface. The resulting materials are recovered by a separation and washing process, and then dispersed in water by sonication and dried by lyophilization. We place 0.1 g of dried sample in a carbon-powder-surrounding crucible inside a conventional microwave oven in an argon (Ar) atmosphere. The microwave oven (Galanz G80F20CN1L-DG (SO)) functions at full power (800 W, 2.45 GHz) in 200 s cycles (on for 150 s, off for 50 s) for a total reaction time of 600 s. The microwave irradiation reduces GO to graphene, removes the PEI moiety, and decomposes PMMA particles. After reaction, hollow rGO nanospheres are obtained, and trifle PMMA residue is removed with tetrahydrofuran.

RAG-Gemini

Poly(methyl methacrylate) (PMMA) serves various purposes in the synthesis and processing of graphene:

*   **As a temporary support layer for graphene transfer:** In some methods, PMMA is spin-coated onto graphene grown on a metal substrate (e.g., copper). This PMMA/graphene film acts as a temporary support during the etching of the metal, allowing the graphene to be subsequently transferred onto another substrate. The PMMA is then removed.

*   **As a substrate:** Graphene flakes, prepared by methods like mechanical cleavage, can be transferred and deposited onto PMMA beams or plates for further study or application.

*   **As a sacrificial template:** PMMA spheres can be used as templates for creating hollow graphene structures. Graphene oxide (GO) nanosheets are adsorbed onto PMMA spheres, and subsequent reduction and microwave irradiation decompose the PMMA, leaving hollow reduced graphene oxide (rGO) nanospheres.

*   **For composite preparation or functionalization:** PMMA can be grafted onto graphene (e.g., NH2-terminated graphene nanoplatelets) or used as a polymer matrix to create composite materials with graphene.

Ground truth

PMMA can be pyrolised to decompose into carbon-rich material which can then form graphene sheets through cvd or some other pyrolytic process. PMMA can also be used as a transfer agent to protect and transfer graphene to other surfaces after growth on copper.

Question 16

What is the purpose of plasma cleaning in preparing substrates for high-quality graphene growth?

Context 1

Author: Lam Van Nang

Doi: 10.1149/2.082204jes

Method:

To investigate the rf plasma effect, graphene growth was carried out with a diluted CH4 gas, in which graphene was not formed without applying plasma. The CH4 gas of 1 SCCM (denoting cubic centimeter per minute at STP) and the mixed gases of 10% H2 and 90% Ar (forming gas) of 100 SCCM were introduced in a hot-wall tubular reaction chamber. The Cu foil (Alfa Aesar) was pre-treated with an acetic acid solution at 60°C for 10 min, followed by washing with de-ionized water. The foil was heated to a graphene synthesis temperature (950°C) and annealed for 10 min with forming gas flow (100 SCCM) to remove native oxides from the Cu foil and to enlarge its grains. The synthesis pressure was fixed at 1 Torr. The synthesis time and rf plasma power is 5min and 200 W, respectively. The synthesized graphene on Cu was transferred on SiO2/Si or glass substrate for various characterizations. First, a 500 nm layer of poly-methyl methacrylate (PMMA) was spin coated on the surface of the graphene film to serve as support. The graphene layer at the back side of the Cu foil was then etched off using oxygen plasma. After the etching of the Cu foil with FeCl3 aqueous solution at 40°C, the PMMA/graphene was transferred to a target substrate. The graphene on the SiO2/Si or glass substrate was achieved by dissolving the PMMA film using acetone

Context 2

Author: Yong Seung Kim

Doi: 10.1039/C2NR33034B

Method:

The graphene films studied in this work were grown on 25 µm-thick Cu foils by plasma-enhanced chemical vapor deposition (PECVD). The chamber is equipped with an inductively coupled plasma reactor. The radiofrequency (RF) coil, having a diameter of ~25 cm, is located 35 cm above the top surface of the graphite substrate holder. In this geometry, the heating effect of the substrate by plasma was negligible. A residual gas analyzer (RGA 100, Stanford Research System) is attached at the side wall of the chamber to investigate the density of discharged species. A differential pumping technique is employed to meet the required working pressure of the RGA. The system is pumped with a turbomolecular pump (Osaka Vacuum LTD, TG1003), keeping the base pressure as low as ~10^-7 Torr. Polycrystalline Cu foil was cut into 7 x 7 cm2 pieces and mounted in the chamber without any pre-cleaning treatment. Five different stages were employed to synthesize graphene films on Cu foil using methane (CH4) as the carbon source. The Cu substrate was heated to the growth temperature

(700–830 °C) at a heating rate of 3 °C/s. When it reached the target temperature, H_2 gas was introduced into the chamber at a flow rate of 40 standard cubic centimeters per minute (sccm). Hydrogen gas was discharged by an RF power of 50 W for 2 minutes to eliminate surface oxides on the copper foil. Then, the chamber was purged with Ar at a flow rate of 100 sccm for 2 minutes to remove residual hydrogen gas. During the graphene growth stage, radiofrequency (RF) plasma was generated for a specified growth time under a continuous flow of argon (or hydrogen, 40 sccm) and methane (1 sccm), while the pressure was kept at 10 mTorr. The plasma power was varied from 10 to 200 W and the growth time from 0.2 to 4 minutes. Subsequently, the sample was cooled down rapidly to room temperature at a cooling rate of 3 °C/s by turning off the heating power, and then it was taken out for characterization.

Context 3

Author: D.A. Boyd

Doi: 10.1038/ncomms7620

Method:

The copper substrates were placed on a quartz flat inside of quartz tube. A typical substrate size was (8×13) mm2. The tube was evacuated to 25 to 30 mTorr. A 2-5 sccm flow of room temperature hydrogen gas with 0.4% methane and a comparable amount of nitrogen gas was added and the pressure was controlled at 500 mTorr. The addition of methane to the gas flow was controlled by a precision leak valve, and a typical concentration, as measured by gas chromatography, was 0.4 %. The initially low vacuum conditions (~ 25 mTorr) provided sufficient partial pressures of nitrogen (atmospheric) for the plasma enhanced chemical vapour deposition (PECVD) process. (We note that a leak valve for including purified nitrogen was added to the setup to study the effects of increased nitrogen partial pressures.) Typical partial pressures measured by residual gas analysis (RGA) are as follows: N_2 (Mass 28): 6.9 × 10^-8 Torr, CH_4 (Mass 16): 1.5 × 10^-7 Torr , O_2 (Mass 32): 5.4 × 10^-9 Torr , H_2O (Mass 18): 1.2 × 10^-6 Torr, CO_2 (Mass 44): 2.3 × 10^-8 Torr. From these values we estimate that the concentration of nitrogen in the gas flow is typically on the same order as that of methane. The PECVD process was found to be highly sensitive to the relative amounts of methane and nitrogen. Excessive methane mixtures resulted in no etching, while excessive nitrogen mixtures would result in excessive etching of the copper. Slight adjustments to the methane concentration could be made via either optical emission spectroscopy (OES) or RGA. A low-power (ranging from 10 to 40 W) cold hydrogen plasma was formed over the copper substrate using an Evenson cavity. Exposure of copper substrates to the plasma enabled continuing etching and cleansing of the copper surface during the graphene growth process. The gas temperature inside the plasma was measured using a thermocouple sheathed in boron nitride. The thermocouple was placed in the plasma above the sample and could be translated along the tube. The peak gas temperature measured in plasma treatment was 160°C at 10 W and 425°C at 40 W, and the gas temperature profile decreased rapidly (by 120°C at 10 W and 425°C at 40 W within 1 cm) from the peak value. The maximum temperature of the copper substrate (Ts) was measured using the melting point of known solids, lead and zinc, and found to be within the range of 327.5°C < Ts < 419.5°C at 40 W. Typically after 5 to 20 minutes of direct exposure to the plasma, a large-area monolayer graphene formed on the backside of copper substrates while the front side of the substrates was coated with disordered graphite. Upon the completion of graphene growth, the plasma was extinguished, and the gas flows were stopped. The process tube was then evacuated and

back filled with Ar, and the substrate with graphene coating was subsequently removed. Copper deposition was visible on the inside of the tube and on the sample holder as the result of plasma etching of the copper substrates.

Context 4

Author: Soo Min Kim

Doi: 10.1088/0957-4484/24/36/365602

Method:

Pre-cleaning step: To remove impurities, the foil was briefly dipped in Ni etchant solution for 30–90 s depending on the number of impurities observed (Note: we have observed that Cu foil having more impurities requires a longer surface cleaning time. However, if the cleaning time is more than 2 min, the copper foil becomes severely damaged and is not useful for growth of graphene.) The main component of the Ni etchant is nitric acid, which reacts with Cu according to Cu(s) + 4HNO3 (aq) -> Cu(NO3)2(aq) + 2NO2(g) + 2H2O. (1) During this reaction, the Cu on the surface is being oxidized and dissolved in the solution; at the same time NO2 gas is violently generated, helping to push the impurity particles away. In this way, the original surface of the Cu foil, together with the impurities, is scraped off and a new, clean but very rough surface is obtained. After cleaning, the copper foil is dipped in DI water for further washing. This washing process is carried out three times, each time with fresh DI water. It should be noted that this step is very important. To remove the DI water from the copper completely, the copper foil is briefly washed with acetone and isopropanol and dried by blowing with N2. Graphene growth procedure: Graphene was synthesized by low pressure chemical vapor deposition using a copper foil (25 μm, 99.8%, Alfa Aesar) as a catalytic metal substrate. After the pre-cleaning process, the copper foil was annealed at 1000°C for 30 min under a 10 sccm hydrogen atmosphere (330 mTorr) to increase the grain size and to obtain a smooth surface, followed by graphene synthesis under 5 and 10 sccm of methane and hydrogen atmospheres for 40 min (450 mTorr) while maintaining the same temperature. During the cooling of the chamber, 10 sccm of hydrogen was flowed until the furnace was cooled to room temperature.

Context 5

Author: Ki Chang Kwon

Doi: 10.1038/srep04830

Method:

Graphene growth: The as-received and electroplated Cu foils were loaded into a thermal chemical vapour deposition (CVD) chamber without precleaning. Graphene samples were grown using methane (CH4) and hydrogen (H2) gas. Under vacuum conditions of 90 mTorr (12 Pa), the furnace was heated without gas flow for 30 min. Before the growth of graphene, the Cu foil was preheated at 950°C for 30 min. In order to increase the grain size of Cu, H2 gas was supplied to the furnace under 150 mTorr (20 Pa) of pressure at a rate of 33 cm3/min (sccm). After the preheating step, a gas mixture of CH4:H2 = 200:33 sccm was supplied under vacuum conditions for 60 min to synthesize the graphene on the Cu foils electroplated by

reusing Cu etchant (R-Cu). The growth time of graphene on the R-Cu foil was reduced to 30 min from 60 min for the as-received Cu foil. After growth, the furnace was cooled to room temperature at a rate of 10–15°C/min under 33 sccm of H2 flow. Poly(methyl methacrylate) (PMMA) or fluoropolymer (CYTOP) was then spin-coated onto the graphene-coated Cu foil as a supporting material and the PMMA (or CYTOP)-coated foil was heated on a hot plate to 180°C for 1 min, after which O2 plasma was used to etch the graphene on the opposite side of the Cu foil. The sample was then immersed in an ammonium persulfate [1M (NH4)2S2O8] bath at room temperature for 3 h to etch away the Cu foil and the etching solution was collected. Then, the remaining PMMA (or CYTOP)-coated graphene was carefully dipped into a de-ionized (DI) water bath 7–9 times to remove any residual etchant. The PMMA (or CYTOP)-coated graphene was then transferred onto a glass substrate. The PMMA (or CYTOP) was removed by an acetone bath at 50°C for 30 min after the PMMA (or CYTOP)/graphene layer had completely adhered to the target substrate.

RAG-Gemini

Plasma cleaning is employed in preparing substrates for high-quality graphene growth primarily to eliminate surface oxides and to ensure continuous etching and cleansing of the copper surface. This process helps create a clean and active surface suitable for graphene synthesis.

Ground truth

Plasma cleaning removes contaminants, organic reside, enhances surface wettability and improves the adhesion of graphene to the substrate.

Question 17

Could you elaborate on the role of copper foil in the synthesis of graphene films using the chemical vapor deposition method?

Context 1

Author: A. Kumar

Doi: 10.1016/j.carbon.2011.11.033

Method:

Graphene film synthesis was performed in a SEKI AX5200S MPCVD (microwave plasma chemical vapor deposition) system with H2 (50 sccm) and CH4 (5 sccm) as the feed gases. A 25 µm thick copper foil (Alfa Aesar, 99.8% purity) was used as the substrate. The foil was cut in 2 x 2 cm2 pieces and placed into the MPCVD chamber, which was evacuated to a base pressure of 2 Torr and then filled with high-purity hydrogen at a pressure of 10 Torr. The Cu foil was supported by a ceramic stand that elevated it from the Mo puck by about 15 mm. The elevation of the Cu foil above Mo puck was found to be critical for the growth of the films because it ensures a strong coupling between the plasma and the Cu foil, thus enabling rapid self-heating of the foil by the microwave plasma. No additional heater was used. The thermocouple attached to the substrate table indicated a maximum reading of approximately 65°C during the process. A dual-wavelength infrared pyrometer indicated a maximum temperature of 700 ± 25 °C. However, as the copper foil is coated with graphene, the surface emissivity changes, making reliable IR measurements difficult. The entire growth is accomplished in less than 5 min and generally involves two stages. First, in a plasma cleaning and annealing step, the foil is kept in 400 W hydrogen plasma for 3 min. A H2 flow rate of 50 sccm was used with downstream pressure control to maintain 10 Torr chamber pressure throughout the process. This process leads to rapid self-heating and removal of copper oxide from the foil surface. The second stage involves introduction of CH4 (while maintaining the hydrogen plasma) at a flow of 5 sccm for a short duration (between 30 s and 2 min), providing approximately 10% CH4 concentration in hydrogen. Depending on the growth duration and whether or not annealing is used, different forms of carbon can be deposited.

Context 2

Author: Richard Piner

Doi: 10.1021/nn4031564

Method:

Graphene films were grown on a 125 µm thick oxygen-free high conductivity (OFHC) copper foil (C10200, Eagle Brass). This foil was cut into 1.5 cm × 3 cm strips that were cleaned using an ultrasonic system (VWR, B2500A-MT) with an acetone bath for 15 min, and then rinsed by acetone and isopropyl alcohol before being blow dried with nitrogen gas. The foil was then placed inside a 0.8 in. ID fused silica tube that was then put inside a 1 in. fused silica tube. (This makes it easy to load/unload the copper foils and clean any evaporated copper from the

small diameter silica tube.) A typical process is as follows: (i) load the Cu foil into the silica tube, evacuate, fill with 5% hydrogen balanced with argon to ambient pressure, heat to 1035 °C for 10 min; (ii) evacuate and fill with pure argon to ambient pressure and heat to 1035 °C for 10 min; (iii) evacuate, fill with pure argon to 100 Torr, heat to 1035 °C, fill the chamber with 5% methane balanced with argon to 150 Torr, keep the temperature at 1035 °C for 10 min and turn off the heating power. The copper foil can reach 1035 °C from room temperature within 2 min, and the cooling rate is typically about 30 °C/s.

Context 3

Author: Soo Min Kim

Doi: 10.1088/0957-4484/24/36/365602

Method:

Pre-cleaning step: To remove impurities, the foil was briefly dipped in Ni etchant solution for 30–90 s depending on the number of impurities observed (Note: we have observed that Cu foil having more impurities requires a longer surface cleaning time. However, if the cleaning time is more than 2 min, the copper foil becomes severely damaged and is not useful for growth of graphene.) The main component of the Ni etchant is nitric acid, which reacts with Cu according to Cu(s) + 4HNO3 (aq) -> Cu(NO3)2(aq) + 2NO2(g) + 2H2O. (1) During this reaction, the Cu on the surface is being oxidized and dissolved in the solution; at the same time NO2 gas is violently generated, helping to push the impurity particles away. In this way, the original surface of the Cu foil, together with the impurities, is scraped off and a new, clean but very rough surface is obtained. After cleaning, the copper foil is dipped in DI water for further washing. This washing process is carried out three times, each time with fresh DI water. It should be noted that this step is very important. To remove the DI water from the copper completely, the copper foil is briefly washed with acetone and isopropanol and dried by blowing with N2. Graphene growth procedure: Graphene was synthesized by low pressure chemical vapor deposition using a copper foil (25 μm, 99.8%, Alfa Aesar) as a catalytic metal substrate. After the pre-cleaning process, the copper foil was annealed at 1000°C for 30 min under a 10 sccm hydrogen atmosphere (330 mTorr) to increase the grain size and to obtain a smooth surface, followed by graphene synthesis under 5 and 10 sccm of methane and hydrogen atmospheres for 40 min (450 mTorr) while maintaining the same temperature. During the cooling of the chamber, 10 sccm of hydrogen was flowed until the furnace was cooled to room temperature.

Context 4

Author: Thomas H. Bointon

Doi: 10.1002/adma.201501600

Method:

25 μm thick copper foils (Alfa Aesar 99.999%) were annealed for 10 minutes at 1035°C in a H2 atmosphere to increase the Cu grain size. A typical processing for the growth of continuous graphene films involves the following steps: (1) heating up the chemical vapour deposition (CVD) system from room temperature to the growth temperature, (2) Cu foil annealing, (3)

graphene nucleation and growth, (4) cooling down the system to room temperature. During the heating up stage, the CVD system was heated to 1035°C in 3 minutes with H2 gas flown at a rate of 0.4sccm and a chamber pressure of 0.01 Torr. The annealing step was performed for 10 minutes at 1035°C in a H2 atmosphere, keeping the H2 gas flow rate at 0.4sccm and the chamber pressure of 0.01 Torr. The temperature was then lowered at 1000°C for the growth of continuous graphene films. A constant flow rate of 0.4sccm of H2 was kept throughout the nucleation and growth. For the nucleation stage, 1.4sccm of CH4 was introduced for 40 seconds. This was followed by the growth stage where the CH4 flow rate was increased to 7sccm for 300 seconds. Finally, the system was cooled down at room temperature keeping the H2 gas flow rate at 0.4sccm.

Context 5

Author: V Miseikis

Doi: 10.1088/2053-1583/2/1/014006

Method:

The 25µm thick Cu foil used in this work, was supplied by Alfa Aesar (purity 99.8%, lot no. 13382). The foil was electropolished in an electrochemical cell made using a commercially available Coplin staining jar as the vessel and an electrolyte solution. The grooves of the staining jar ensured that the foil was kept flat and parallel to the counter electrode (a thicker Cu plate), which helped achieve homogeneous polishing of the surface. Graphene films were synthesized at a pressure of 25 mbar inside a 4 inch cold-wall chemical vapour deposition (CVD) system (Aixtron BM). To achieve more reproducible atmospheric and thermal conditions and to reduce the effective gas flow, the sample was contained in a custom-made enclosure, comprising a quartz disk suspended 6 mm above the sample using graphite spacers. A typical CVD growth process contains four distinct parts: temperature ramp-up (I), annealing (II), growth (III) and cool-down (IV). The annealing as well as the growth was performed at approximately 1060 °C, which was calibrated according to the melting point of Cu. The annealing time was kept at 10 min in all cases. The gas flow during the temperature ramp up and the annealing stages was 1000 sccm. The samples which had the largest grains were annealed in argon atmosphere; other samples were annealed in hydrogen. The gas flow rates during growth were typically set to 1 sccm of methane, 20 sccm of hydrogen and 900 sccm of argon. The growth time was one hour. After the growth, the chamber was cooled in argon/hydrogen atmosphere to a temperature of 120 °C before introducing the samples to air.

RAG-Gemini

In the synthesis of graphene films using chemical vapor deposition (CVD), copper foil plays a crucial role primarily as a **catalytic metal substrate**.

Key aspects of its role include:

* **Catalytic Activity**: Copper actively facilitates the decomposition of carbon-containing precursor gases (like methane) into carbon atoms, which then precipitate and form graphene on its surface.

*   **Substrate for Growth**: It provides the physical surface upon which graphene nucleation and growth occur.

*   **Influence on Graphene Quality**: The surface properties of the copper foil, such as its cleanliness, smoothness, and grain size, significantly influence the quality, domain size, and continuity of the synthesized graphene films. Pre-cleaning steps (e.g., ultrasonic cleaning, chemical etching, electropolishing) are often employed to remove impurities and oxides, while annealing at high temperatures (e.g., 1000-1060 °C) is used to increase the copper grain size and obtain a smoother surface, which are beneficial for graphene growth.

*   **High-Temperature Stability**: Copper's ability to withstand the high temperatures typically required for CVD (often over 1000 °C) makes it a suitable substrate for the process.

Ground truth

The copper foil usually acts as a substrate and catalyst for growing graphene layers in CVD. It provides a surface on which carbon atoms can nucleate and arrange themselves into a graphene structure.

Question 18

What role does H2O2 play in the synthesis of graphene oxide?

Context 1

Author: Dheeraj

Doi: 10.1016/j.poly.2024.117175

Method:

Graphene oxide was produced employing a modified Hummer's method. Initially, a mixture of H2SO4 and H3PO4 in a 9:1 vol ratio was prepared and placed in a 500 mL round bottom flask, where it was vigorously stirred. Subsequently, 0.55 g of graphite powder was introduced into the acid mixture while maintaining continuous stirring. After achieving complete homogenization, 2.74 g KMnO4 was gradually poured into the solution with vigorous stirring. The solution exhibited a transition to a dark green color during this process, and a small quantity of H2O2 was added dropwise, causing an exothermic reaction. To control the reaction's temperature, the entire process was conducted within an ice bath. Following this, a solution composed of 10 mL of hydrochloric acid and 50 mL of deionized water was introduced into the mixture over a span of 10 min. The resulting residue was carefully collected and subjected to multiple wash cycles with HCl and DI water. Lastly, the precipitated material was desiccated in an oven at 70 °C for a duration of 12 h.

Context 2

Author: Shruti Rialach

Doi: 10.1016/j.electacta.2025.145976

Method:

Synthesis of graphene oxide: Graphene oxide was synthesized via streamlined version of Hummers' Process. A fresh piranha solution was prepared by mixing concentrated sulfuric acid (H2SO4) and concentrated phosphoric acid (H3PO4) in a volumetric ratio of 9:1 in a beaker. The typical concentrations used were 0.81 M of H2SO4 and 0.09 M of H3PO4 to harness a 0.75 M acidic mixture which serves as the medium for oxidation. The solution was ultrasonicated for more than 30 min, followed by a stirring at 500 rpm for another 30 min. Thereafter, 0.675 mM of graphite powder was incorporated into the acid mixture while stirring continuously, ensuring the mixture is uniform and well-dispersed. Slowly, 3.96 mM of potassium permanganate (KMnO4) was introduced into the mixture. The slow addition helps in controlling the exothermic reaction and prevents overheating. Once all the KMnO4 was added, the mixture was maintained under agitation for 22 h to ensure complete oxidation. After the oxidation period, hydrogen peroxide (H2O2) was added to the mixture to reduce the residual KMnO4 and other manganese compounds until the color of the solution turned yellowish-green (2.03 mM of H2O2 was used in the reduction of this mixture). The yellow-green solution was ultrasonicated for 30 min to ensure an intimate contact between the solute and the solvent, and to break down the particles into smaller ones, again followed by

continuous stirring. Next, 100 mL diluted HCl solution (HCl:DI water = 1:3 v/v) was gradually added dropwise to the former solution using a standard rapid transfer port (RTP) three-part syringe inside a well-ventilated fume hood, initiating an exothermic reaction which caused the solution to turn dark brown, indicating the complete reduction process. The final graphene oxide solution was allowed to cool naturally for about 2–3 h in the fume hood.

Context 3

Author: Arjunan Ariharan

Doi: 10.4236/graphene.2016.52005

Method:

Graphene Oxide (GO) was synthesized from graphite by a modified Hummers method. Briefly, graphite powder (2.0 g) was taken in a beaker, added 50 m of concentrated sulfuric acid (H2SO4) under vigorous agitation in an ice bath. Afterwards, sodium nitrate (2.0 g) and potassium permanganate (6.0 g) were slowly added in a sequence. Then, the mixture was transferred into a water bath and kept at 35˚C for 2 hour. After that, 100 mL of distilled water was slowly added, causing a temperature rise to 98˚C. Later, 140 mL of 4% H2O2 was dropped into the reaction system. Finally, the product was washed with distilled water three times. The acquired solid was dried in vacuum at 50˚C for 48 hours.

Context 4

Author: Abhijit Ganguly

Doi: 10.1021/jp203741y

Method:

Highly oxidised Graphene Oxide (GO) was produced using a modified Hummers' process. The starting material Graphite powder with particle size ≤20 μm was purchased from Fluka and is denoted here as "pristine" graphite. All other chemical and reagents were purchased from Aldrich. A mixture of 2.5 g of Graphite and 1.9 g of NaNO3 was placed in a flask cooled in an ice bath. 85 mL of H2SO4 was added to the mixture and stirred until homogenized. Solution of 11.25 g of KMnO4 in distilled water was gradually added to the solution while stirring. After 2 hours, the solution was removed from the ice bath, and further stirred for 5 days. Finally, brown-coloured viscous slurry was obtained. The slurry was added to 500 mL aqueous solution of 5 wt% H2SO4 over 1 hour while being continuously stirred. The mixture was stirred for a further 2 hours. Subsequently, 10 ml of H2O2 (30 wt% aqueous solution) was then added to the mixture and stirred for further 2 hours. This mixture was then left to settle overnight. The mixture was filtered and further purified by dispersing in 500 mL aqueous solution of 3 wt% H2SO4 and 0.5 wt% H2O2. After two days of precipitation, the supernatant solution was removed. This process was repeated five times. The solid product obtained after the rigorous cleaning process was rinsed using copious amounts of distilled water and dried in oven, as reported in literature. The resulting solid was dispersed in water by ultrasonication for 2 h to produce a GO aqueous dispersion. After one-day sedimentation, the thick flakes were removed and the supernatant was collected for further measurements.

Context 5

Author: Benjamin Diby Ossonon

Doi: 10.1039/c6ra28311j

Method:

Graphene oxide was synthesized from natural graphite through the Hummers method which has been improved. The graphite is first pre-oxidized by mixing 5g graphite powder with concentrated sulfuric acid (12.5 mL), potassium persulfate (2.5 g) and phosphorus pentoxide (2.5g). The mixture was heated at 80°C for 6 hours. After dilution with 500 mL of H2O, the mixture was stirred at room temperature overnight. After that, the product is recovered by centrifugation and washed thoroughly with Nanopure water until the filtrate has a pH close to 7. The product obtained is then dried at room temperature for one day. Then, the pre-oxidized graphite is dispersed in H2SO4 (0°C, 115 mL). The temperature of the mixture is carefully controlled to not exceed 10°C. Subsequently, potassium permanganate (15 g) is gradually added with constant stirring for 1 hour. The dispersion is then incubated at 35°C for 2 h and this is followed by the addition of Nanopure water (225 mL) in small portions (15 mL) to control the temperature of the mixture, which must remain below 50°C. To completely dissolve the KMnO4 , hydrogen peroxide (30%, 12.5 mL) was immediately added at the end of a second dilution (H2O, 700mL), and the mixture is stirred for 48 hours. Finally, the suspension is filtered, washed first with HCl (10%) to remove residual metal ions, and repeatedly with Nanopure water until the pH of filtrate becomes neutral. The filtrate is quickly tested by adding a few drops of 1 M NaOH to verify the presence of metal ions in graphene oxide (GO). The product obtained (graphite oxide) is then dried in air. The resultant graphite oxide was dispersed in Nanopure water kept in the ultrasonic bath for 24 hours to maximize exfoliation. The reduced graphene oxide (RGO) is obtained by thermal reduction of GO in Ar/5% H2 at 800°C for 2 h. The resulting RGO can be dispersed in water and the dispersion stayed stable for few hours.

RAG-Gemini

In the synthesis of graphene oxide, hydrogen peroxide (H2O2) is typically added to reduce residual potassium permanganate (KMnO4) and other manganese compounds. This process helps to quench the oxidation reaction and aids in the removal of manganese byproducts, often indicated by a color change of the solution (e.g., to yellowish-green or brown).

Ground truth

It reduces excess oxidisers such as potassium permanganate in variants of the Hummer's method, thus halting oxidation and preventing uncontrolled reactions. It also can promote the formation of grpahene oxide by facilitating the unravelling of carbon chains.

Question 19

Which method would u recommend for synthesizing monolayer graphene for producing transistors?

Context 1

Author: Thomas Moldt

Doi: 10.1021/nn202293f

Method:

Materials: Single-crystal graphite flakes (National de Graphite) 1.7 mm in size have been used to produce graphene. Few depositions have been performed with very large single-crystal graphite flakes, with a size of 5 mm. The graphite flake is cleaved once using sticky tape in order to achieve a clean and fresh surface. The flake is then placed on a microscope coverslip, with a thickness of 120 µm (Menzel-Gläser). The coverslip is cleaned before deposition by sonication in acetone and then 2-propanol. Anodic Bonding Setup: This is composed of a grounded metal block used as back electrode and can be heated to 300 °C using a temperature feedback controlled heating plate. The glass coverslip is placed on the grounded electrode. The top electrode, a cylindrical metal rod with a diameter of 2 mm, mounted vertically above the back gate, is pressed on the graphite flake, while applying a dc voltage for 20–30 min. The setup allows dc voltages of up to 10 kV. After the deposition, thick graphite material is removed from the coverslip by using sticky tape. Anodic Bonding procedure: In the anodic bonding a single crystal flake of graphite is pressed on glass, and a high voltage of 0.5–2 kV is applied between the graphite and a metal back contact, while heating the glass at about 200 °C for 10–20 min. In case of the positive electrode applied to the top contact, a negative charge concentration occurs in the glass at the side facing the positive electrode. A few layers of graphite, including single layers, stick on the glass by electrostatic interaction. The anodic bonding is a simple technique because there are only two deposition parameters: temperature and voltage. Thus, in order to determine the optimum conditions to have high-yield and high-quality single-layer graphene, we made several samples at different temperatures (between 160 and 260 °C) and voltage (between 0.4 and 3 kV). Transfer and Transport: The graphene flakes produced by anodic bonding have been transferred to other substrates by using the wedging technique. We transferred graphene flakes from the coverslip to a silicon substrate covered with 90 nm silicon oxide (IDB Technology) for transport measurements. Electron beam lithography and e-beam evaporation were used to prepare a set of contacts (5 nm Ti/50 nm Au). A Hall bar mesa structure has been prepared by reactive plasma etching.

Context 2

Author: Uk Sim

Doi: 10.1021/acsami.6b11750

Method:

Monolayer graphene was synthesized using thermal chemical vapour deposition (CVD). First, a copper (Cu) foil (10 × 10 cm2) was placed in a quartz tube and heated to 1000 °C for 60 min with flowing H2 at 10 sccm. Then, a gas mixture of 45 sccm CH4 and 10 sccm H2 flowed for 30 min under 10 Torr. After 30 min, the heater was turned off and removed from the Cu foil. Next, to handle the monolayer graphene on the front side of the Cu foil, a protective layer of poly(methyl methacrylate) (PMMA) was spin-coated on the graphene on the front side of the Cu foil, and the graphene on the back was removed using oxygen plasma (100 W RF power, 12 s). Then, the PMMA film on the graphene was removed using acetone, and the remaining Cu was etched away using a 0.1 M ammonium persulfate solution. Finally, the monolayer graphene was rinsed in deionized water and transferred onto a p-type silicon substrate without a silicon oxide layer. For the various stacked graphene layers, graphene with PMMA was floated on deionized water and transferred onto another graphene layer on Cu and etched/rinsed again. The transfer and etching/rinsing procedure was repeated for up to 4 layers of graphene. To synthesize graphene with defects, the monolayer graphene on the back side of the copper was removed using oxygen plasma, and the graphene on the front side of the Cu was treated with argon plasma (10 W RF power, 4 s). The PMMA layer was coated onto this graphene, and the Cu foil was removed. Then, the treated graphene was transferred onto silicon or used in the stacking process.

Context 3

Author: Florian R. Ong

Doi: 10.1088/0957-4484/26/40/405201

Method:

Graphene layers are then obtained by mechanical exfoliation of kish graphite onto a separate Si/SiO2 substrate, which we call the exfoliation substrate. Using Si/SiO2 as a substrate enables the discrimination of one to few layer flakes by inspection with an optical microscope. The number of layers is then confirmed by Rama spectrometry, and a monolayer is selected for the transfer step to follow. A solution of cellulose acetate butyrate (CAB) in ethyl acetate 60 mg/mL, which is a hydrophobic polymer, is dripped onto the substrate, covering the graphene monolayer of interest. The latter is still distinguishable under the optical microscope after the CAB polymer dries off. The needles of the micromanipulator are used to punch markers around the monolayer. These markers will later allow for the alignment of the graphene relative to the gate on the destination substrate, onto which graphene is optically indiscernible.

Context 4

Author: Jian-Hao Chen

Doi: 10.1002/adma.200701059

Method:

First, photolithography is used to prepare 30 nm thick Au source and drain electrodes on a silicon wafer with an oxidized surface (SiO2/Si). The electrodes are then transferred onto the Polyethylene Terephthalate (PET) substrate as described elsewhere. Then, single-and few-

layer graphene is obtained from Kish graphite by mechanical exfoliation on 300 nm thick thermally grown silicon dioxide on silicon substrates, and its thickness and morphology characterized by atomic force microscopy. Mechanical exfoliation yields atomically clean graphene sheets and our AFM images also indicate that the graphene sheet is free of nanometer-scale contaminants. In addition, chemical contamination caused by exposure to photoresist and lift-off chemicals is avoided in this process. The desired graphene sheet is printed at 170°C at 500 psi from the $SiO2/Si$ substrate to the source-drain electrode assembly on PET.

Context 5

Author: Peiyue Shen

Doi: 10.1088/2752-5724/acbecd

Method:

The graphene samples used in this experiment were mechanically exfoliated from bulk graphite onto the silicon substrate with an oxide layer of about 285 nm. The graphene layer numbers and domains were first selected roughly by optical microscopy and SNOM system with a $CO2$ laser (~ 10.6μm), and then confirmed by a homemade Raman spectroscopy system. The selected samples were then made to be the standard field effect transistors by Electron Beam Lithography System and Electron-beam Evaporation System, and the top metal gate is made of 3nm Cr and 30nm Au. Then the samples were annealed at 180℃ with 30 sccm $H2$ plasma of 35W power for one hour to remove the organic residues on the surface. Finally, we used Atomic Force Microscopy (AFM) tips at lift mode to remove the remained contaminations on the graphene surface. The force of the AFM tip was set at a tiny value to avoid changing the domain shape.

RAG-Gemini

For synthesizing monolayer graphene for producing transistors, the method described by Peiyue Shen et al. is recommended.

This method involves:

1. **Mechanical Exfoliation:** Monolayer graphene is obtained by mechanically exfoliating bulk graphite onto a silicon substrate with an oxide layer (approx. 285 nm thick). The number of layers is initially selected using optical microscopy and a SNOM system, then confirmed by Raman spectroscopy.

2. **Device Fabrication:** The selected graphene samples are then directly processed into standard field effect transistors using Electron Beam Lithography and Electron-beam Evaporation. The top metal gate for these transistors is composed of 3 nm Cr and 30 nm Au.

3. **Contamination Removal:** To ensure high device quality, the samples are annealed at 180 °C with $H2$ plasma (30 sccm, 35W power) for one hour to remove organic residues. Additionally, Atomic Force Microscopy (AFM) tips in lift mode are used with a tiny force to remove any remaining contaminations on the graphene surface without altering its domain shape.

This approach directly yields high-quality graphene and details its integration into a functional transistor, including crucial cleaning steps for optimal performance.

Ground truth

Micromechanical Cleavage.

Question 20

Which method would u recommend for synthesizing few layer graphene for dispersion into inks?

Context 1

Author: F. Torrisi

Doi: 10.1021/nn2044609

Method:

We prepare the graphene-based printable ink as follows. Graphite flakes are sonicated (Decon bath, 100W) in N-Methylpyrrolidone (NMP) for 9 hours. The unexfoliated flakes are left to settle for 10 mins after sonication. The decanted dispersions are then ultracentrifuged using a TH-641 swinging bucket rotor in a Sorvall WX100 Ultra-centrifuge at 10,000 rpm (~15,000g) for an hour and filtered to remove flakes>1μm, that might clog the nozzle. The resulting ink is characterized by Optical Absorption Spectroscopy (OAS), High Resolution Transmission Electron Microscopy (HRTEM), Electron diffraction and Raman spectroscopy

Context 2

Author: Y. Hernandez

Doi: 10.1038/nnano.2008.215

Method:

We prepared a dispersion of sieved graphite powder in N-methylpyrrolidone (NMP) by bath sonication. After sonication we obtained a grey liquid consisting of a homogenous phase and large numbers of macroscopic aggregates. As with nanotube dispersions, these aggregates could be removed by a mild centrifugation (CF), giving a homogenous dark dispersion. In order to find the concentration after CF, we filtered the graphite dispersion through polyvinylidene fluoride (PVDF) filters. Careful measurements of the filtered mass, accounting for residual solvent, gave the concentration of dispersed phase after centrifugation. This procedure was repeated for three other solvents known to successfully disperse nanotubes: N,N-Dimethylacetamide (DMA), γ Butyrolactone (GBL) and 1,3-Dimethyl-2-Imidazolidinone (DMEU). These dispersions were then characterised by UV-vis-IR absorption spectroscopy with the absorption co-efficient plotted versus wavelength. The spectra are featureless in the visible-IR region as is expected from theory. Each of these four dispersions were diluted a number of times and the absorption spectra recorded. Finally, we have deposited graphene monolayers on SiO2 surfaces via spray coating, demonstrating that this processing method can potentially be used to prepare samples for microelectronic applications.

Context 3

Author: Emanuele Lago

Doi: 10.1039/C6RA21962D

Method:

Graphene dispersion is produced by liquid phase exfoliation (LPE) of natural graphite. 500 mg of graphite flakes are dispersed in 50 mL of NMP and exfoliated in a sonic bath (VWR Ultrasonic Cleaner USC-THD) for 6 hours. Then, the dispersion is ultra-centrifuged at 10000 rpm (~17000 g), using sediment based separation (SBS) to remove un-exfoliated and thicker graphite flakes, for 30 min at 15 °C with an ultra-centrifuge (Beckman Coulter Optima™ XE-90, equipped with a SW32Ti rotor). Finally, the supernatant is collected by pipetting. A solvent exchange process is carried out for the re-dispersion of the exfoliated flakes in 1,3-dioxolane, a nontoxic and low boiling point (78 °C) solvent, using a Heidolph Hei-Vap rotary evaporator. After the evaporation process of N-methyl-2-pyrrolidone (NMP), the graphitic material is collected and washed three times with acetone to remove the NMP residual using a compact centrifuge (Sigma-Aldrich). The washing step is repeated and the flakes are eventually dispersed in 50 ml of 1,3-dioxolane, adjusting its concentration at 10 mg mL-1.

Context 4

Author: Andrea Capasso

Doi: 10.1016/j.ssc.2015.08.011

Method:

We exploited liquid phase exfoliation of graphite to produce the graphene inks in NMP and in the mixture EtOH/H2O. For the NMP-based ink, 1 g of graphite flakes (Sigma Aldrich) was dispersed in 100 mL of NMP and ultrasonicated (Branson ® 5800) for 6 hours. The obtained dispersion was then ultracentrifuged at ~16000 g (in a Beckman Coulter Optima™ XE-90 with a SW41Ti rotor) for 30 mins at 15 °C, exploiting sedimentation-based separation (SBS) to remove thick flakes and un-exfoliated graphite. After the ultracentrifugation process, we collected the supernatant by pipetting. The optimization of ink-jet printing ideally requires highly concentrated inks. To achieve such a target, the supernatant extracted after the first ultracentrifugation process was further ultracentrifuged at ~200,000g for 60 mins at 15 °C. The high g force value promotes the sedimentation of the graphene flakes at the bottom of the ultracentrifuge tubes taking advantage of the higher density of the graphene flakes (~2.1g/cm3) in comparison with the solvent (ρNMP = 1.03 g/cm3). The pellet (sedimented graphene flakes) is collected and the supernatant is discarded. The pellet was re-suspended in 3 mL of pure NMP using an ultrasonic bath for 10 min. This time was sufficient to re-disperse the graphene flakes, thus obtaining a stable (for more than 2 months) ink. The EtOH/H2O ink was then prepared as follows. 1 g of graphite was dispersed in an EtOH/H2O mixture [1:1 in volume] by ultrasonication for 6 h (Branson® 5800). The mixture was centrifuged at 670g for 10 min (in a Beckman Coulter Optima™ XE-90 with a SW41Ti rotor), longer centrifugation time or higher speed endorses the precipitation of the flakes in dispersion together with non-exfoliated graphitic material. After the centrifugation process, the supernatant was collected by pipetting. In order to get a concentrated graphene ink, the supernatant was ultra-centrifuged at ~16,000g for 15 min at 15 °C. The supernatant was discarded and the pellets were re-suspended in 3 mL of pure EtOH/H2O [1:1] mixture, using an ultrasonic bath for 10 min, to re-disperse the graphene flakes, thus obtaining the final ink. After 1 week, there is the formation

of sediments that are however easily re-dispersed in the same solvent by manual shaking of the bottle containing the ink.

Context 5

Author: Ethan B. Secor

Doi: 10.1002/adma.201502866

Method:

Graphene was exfoliated from graphite using a high shear mixer (Silverson L5M-A) with a square hole high shear screen. Ethyl cellulose (EC) (Sigma–Aldrich, 4 cP grade as measured at 5% in 80:20 toluene: ethanol, 48% ethoxy) was dissolved in ethanol (Koptec, 200 proof) at a concentration of 0.2–2% w/v, and flake graphite (Asbury Graphite Mills, Grade 3061) was added at 10% w/v. This mixture was shear mixed for 2 h at 10,230 rpm in an ice bath, and then centrifuged at 4000 rpm (≈3000 g) for 2 h to sediment out large graphite flakes (Beckman Coulter Avanti J-26 XPI centrifuge). The supernatant containing graphene, EC, and ethanol was harvested by pipette. For the flocculation step, this supernatant was mixed in a 16:9 wt. ratio with an aqueous NaCl solution (0.04 g/mL NaCl, Sigma–Aldrich, >99.5%) and centrifuged for 6 min at 7500 rpm (≈10 000 g) to sediment the graphene/ EC composite. This sediment was washed with deionized water, collected by vacuum filtration (Millipore Nitrocellulose HAWP 0.45 μm filter paper), and then dried to yield the graphene/EC powder, with a graphene content of 25–65 wt% depending on the starting EC loading.

RAG-Gemini

A recommended method for synthesizing few-layer graphene for dispersion into inks is liquid phase exfoliation (LPE) of graphite followed by a multi-step centrifugation process.

1. **Exfoliation:** Disperse graphite flakes in a suitable solvent, such as N-methylpyrrolidone (NMP), and exfoliate them using sonication (e.g., bath sonication for 6-9 hours).

2. **Initial Separation:** After sonication, remove unexfoliated and thicker graphite flakes through mild ultracentrifugation (e.g., 10,000 rpm, or approximately 15,000-17,000g, for 30 minutes to 1 hour). Collect the supernatant, which contains the exfoliated graphene. This step, often referred to as sedimentation-based separation (SBS), is crucial for selecting few-layer flakes.

3. **Concentration (Optional but Recommended for Ink-Jet Printing):** To achieve highly concentrated inks, further ultracentrifuge the collected supernatant at a much higher force (e.g., ~200,000g for 60 minutes). This high g-force promotes the sedimentation of the exfoliated graphene flakes, forming a pellet at the bottom.

4. **Re-dispersion:** Discard the supernatant and re-disperse the collected graphene pellet in a minimal volume of the desired solvent (e.g., NMP or an EtOH/H2O mixture) using an ultrasonic bath for about 10 minutes to obtain a stable, concentrated graphene ink.

This approach ensures the removal of larger flakes and allows for the preparation of highly concentrated, stable graphene inks suitable for various applications. The few-layer nature of the exfoliated graphene can be confirmed by characterization techniques such as Optical Absorption Spectroscopy, High Resolution Transmission Electron Microscopy, Electron diffraction, and Raman spectroscopy.

Ground truth

Pyrolysis

S5. Answer set for Gemini

Question 1

What are the steps involved to synthesize graphene via chemical vapour deposition?

Gemini

The steps involved in synthesizing graphene via chemical vapor deposition (CVD) are:

1. **Substrate Preparation:** A metal catalyst substrate (commonly copper or nickel foil) is cleaned and placed in a reaction chamber.

2. **Heating:** The chamber is heated to a high temperature (typically 800-1100 °C) under a vacuum or inert gas atmosphere (e.g., argon).

3. **Gas Introduction:** A carbon-containing precursor gas (e.g., methane, acetylene) and often hydrogen are introduced into the chamber. Hydrogen can act as a catalyst or etchant to control graphene growth.

4. **Graphene Growth:** The carbon precursor gas decomposes on the hot metal surface, and carbon atoms dissolve into or adsorb onto the metal. As the system cools or the carbon supply is maintained, these carbon atoms precipitate and arrange into a hexagonal lattice structure, forming graphene on the substrate surface.

5. **Cooling:** The chamber is cooled down, often under an inert atmosphere, to stabilize the grown graphene.

Ground truth

First, a substrate, such copper, is cleaned, prepared and placed into a CVD chamber that is usually a tube made out of an inert material such as quartz. After which, the CVD chamber is evacuated into a vacuum or flooded with inert gases.The CVD chamber is then heated to some kind of critical temperature (about 1000 degrees celsius), before feedstock or precursor gases with high hydrocarbon contents are pumped into the CVD chamber. The high temperature in the chamber decomposes these feedstock gases into carbon atoms which then deposit onto the substrate's surface where they reassemble to into the characteristic graphene lattice. The feedstock gases also produce hydrogen which do not contribute to the reaction. The choice of substrate matters as it plays a role in catalysing the reaction. The temperature and duration of this deposition is controlled to either form mono-layer or few-layer graphene.

Question 2

What are the steps involved to synthesize graphene via mechanical exfoliation?

Gemini

The steps involved to synthesize graphene via mechanical exfoliation are:

1. **Obtain a bulk graphite crystal:** Start with a high-quality graphite sample.

2. **Apply adhesive tape:** Press a piece of adhesive tape onto the graphite surface.

3. **Peel the tape:** Carefully peel the tape away, which removes layers of graphite.

4. **Repeat peeling (optional, for higher yield):** Fold the tape onto itself or use fresh tape to repeatedly exfoliate thinner layers from the transferred graphite.

5. **Transfer to substrate:** Press the tape containing the exfoliated graphite layers onto a desired substrate (e.g., silicon dioxide/silicon wafer).

6. **Remove the tape:** Carefully peel away the tape, leaving behind thin flakes of graphite, including single and few-layer graphene, on the substrate.

7. **Identify graphene:** Use an optical microscope to locate the transparent, single-layer graphene flakes on the substrate.

Ground truth

Mechanical exfoliation of graphene, also known as the sticky tape method, involves first taking a piece of graphite, and then using a piece of sticky tape release a small layer of graphite onto it. This is done simply by placing the sticky tape repeatedly on the piece of graphite. To produce single atomic sheets of graphene, this tape is folded in half and repeatedly pulled apart, thereby shearing layers of graphene from the already deposited material until only a single-atomic layer is present.

Question 3

What are the steps involved to synthesize graphene via plasma enhanced chemical vapour deposition (PECVD)?

Gemini

The steps involved in synthesizing graphene via Plasma Enhanced Chemical Vapor Deposition (PECVD) typically include:

1. **Substrate Preparation:** A metallic substrate (commonly copper or nickel foil) is cleaned and placed inside the PECVD chamber.

2. **Precursor Introduction:** Carbon-containing precursor gases (e.g., methane, acetylene, ethanol vapor) along with carrier/dilution gases (e.g., hydrogen, argon) are introduced into the reaction chamber.

3. **Plasma Generation:** Radio frequency (RF) or direct current (DC) power is applied to ionize the precursor gases, generating a plasma. This plasma creates highly reactive species (radicals, ions, electrons).

4. **Substrate Heating:** The substrate is heated to a specific temperature, which aids in the catalytic decomposition of precursors and promotes graphene growth.

5. **Graphene Deposition/Growth:** Reactive carbon species from the plasma deposit onto the heated substrate surface, forming a graphene film. The plasma energy can help lower the growth temperature compared to thermal CVD.

6. **Cooling:** The substrate is cooled down, allowing the synthesized graphene film to stabilize.

Ground truth

The plasma-enhanced process is a modification of traditional chemical vapour deposition. As with traditional CVD, a prepared substrate (cleaned and heated to about 1000 degrees celsius) is inserted into a a CVD chamber made out of an inert material and gaseous precursors such as methane are introduced into the reaction chamber together with other inert gases such as H2 or Ar. Often plasma-enhanced CVD can use lower temperatures than traditional CVD. A plasma, typically generated by radio frequency energy at 13.56Mhz is used to excite and disassociate the precursor gases. This process creates highly reactive carbon radicals which deposit and self-assemble ont he heated substreate forming graphene layers. The growth can be tailored by adjusting parameters such as gas flow rates, plasma power and time.

Question 4

What are the steps involved to synthesize graphene via electrochemical exfoliation?

Gemini

The steps involved in synthesizing graphene via electrochemical exfoliation are:

1. **Prepare the electrochemical cell**: Set up a two- or three-electrode system with a graphite source (e.g., graphite foil, rods, flakes) as the working electrode, an inert material (e.g., platinum, carbon) as the counter electrode, and optionally a reference electrode (e.g., Ag/AgCl, SCE).

2. **Prepare the electrolyte**: Immerse the electrodes in a suitable electrolyte solution (e.g., aqueous salts, ionic liquids, organic solvents with salts).

3. **Apply an electrical potential or current**: A voltage is applied between the working and counter electrodes, or a constant current is passed through the system. This drives the intercalation of electrolyte ions into the graphite layers.

4. **Exfoliate the graphite**: The intercalation of ions, often coupled with gas evolution (e.g., H2 or O2 from water splitting) between the graphite layers, leads to the mechanical separation and exfoliation of individual or few-layer graphene sheets from the bulk graphite.

5. **Collect and purify**: The exfoliated graphene flakes disperse in the electrolyte and are then collected (e.g., by filtration, centrifugation) and washed to remove residual electrolyte and impurities.

Ground truth

First, an electrochemical set up is made involving a graphite working electrode, a counter electrode, a referene electrode and electrolyte and a power supply. All electrodes are inserted into the liquid electrolyte. Using the power supply, either a positive or negative potential is applied to the graphite electrode which either attracts anions (positive potential) or cations (negative potential) from the graphite electrode which cases expansion. The graphene can be subsequently exfoliated by mixing in the precursor.

Question 5

What are the steps involved to synthesize graphene via arc discharge?

Gemini

The steps involved in synthesizing graphene via arc discharge are:

1. **Set up Reaction Chamber:** An enclosed chamber is prepared and filled with an inert gas (e.g., argon, helium) at a controlled pressure. Hydrogen or other reactive gases may also be introduced.

2. **Position Graphite Electrodes:** Two high-purity graphite rods are placed inside the chamber, serving as the anode and cathode, with a small gap between them.

3. **Initiate Arc Discharge:** A high current (DC or AC) is applied across the electrodes, generating an intense electric arc between them.

4. **Vaporization and Plasma Formation:** The extreme heat of the arc vaporizes the graphite electrodes, creating a high-temperature carbon plasma.

5. **Nucleation and Growth:** As the carbon atoms and clusters from the plasma cool rapidly, they nucleate and grow into graphene flakes.

6. **Collection:** The synthesized graphene, often mixed with other carbon nanomaterials, deposits as a soot-like material on the chamber walls or a collection substrate.

Ground truth

Graphene synthesis using arc discharge usises an electric arc to vaporise graphite,followed by the subsequest depostion of the graphene atoms and the formation of graphene flakes. You first need an arc reactor, with two water-cooled electrodes. The anode is usually filled with graphite which the cathode is a graphite rod. An electric arc is then established between the electrodes, using a high current (200A and under a specific pressure (e.g 500 Torr). This high temperature of the arc vaporises the graphite at the anode. The vaporised carbon atoms travel through the arc plasma and deposit on the chamber walls as graphene flakes.

Question 6

What are the steps involved to synthesize graphene via pyrolysis?

Gemini

The steps involved to synthesize graphene via pyrolysis generally include:

1. **Precursor Preparation:** Selecting and preparing a carbon-containing precursor (e.g., polymer, biomass, or organic compound). This may involve cleaning, grinding, or impregnating the material.

2. **Pyrolysis:** Heating the precursor in an inert atmosphere (e.g., nitrogen, argon, or vacuum) to high temperatures (typically 600-3000 °C). This decomposes the precursor, driving off volatile components and leaving behind a carbonaceous char.

3. **Graphitization (Optional/Integrated):** For higher quality graphene, the pyrolysis might be followed by or conducted at temperatures high enough to induce graphitization, where the amorphous carbon atoms arrange into ordered graphitic layers.

4. **Cooling:** Allowing the synthesized material to cool down in an inert atmosphere to prevent oxidation.

5. **Post-processing/Purification:** Further steps may be required to purify the graphene, such as acid treatment, washing, or mechanical exfoliation, to remove impurities or separate the graphene from other carbon forms (e.g., amorphous carbon, multi-layered graphite).

.

Ground truth

Pyrolysis is done by first transforming organic materials like biomass into a carbonaceous material followed by further processing into graphene structures. First, biomass sources, such as rice husks or wheat straw are selected and pretreated by grinding, drying or in some cases, treated with chemicals like KOH to enhance carbonization. Pyrolysis is then carried out in a controlled, inert environment in a furnace or reactor. The temperature is gradually increased in the chamber in the reactor (typically between 600 to 1,200 degrees C), which causes the precursor material to decompose, thereby releasing volatile components and leaving behind a carbonaceous residue. The carbonaceous material is then post processed such as acid washing and annealing to improve the material's structural order to become graphene.

Question 7

What are the steps involved to synthesize graphene via liquid phase exfoliation?

Gemini

The steps involved in synthesizing graphene via liquid phase exfoliation are:

1. **Selection of Solvent:** Choose a suitable solvent or solvent mixture (e.g., NMP, water with surfactants).

2. **Dispersion of Graphite:** Add bulk graphite powder to the selected solvent.

3. **Exfoliation (Sonication/Shear Mixing):** Apply high-energy sonication or high-shear mixing to delaminate the graphite into individual graphene flakes.

4. **Separation/Purification:** Subject the dispersion to centrifugation or allow it to settle to separate unexfoliated graphite and thicker flakes from the desired few-layer graphene.

5. **Collection:** Decant or extract the supernatant containing the exfoliated graphene dispersion.

Ground truth

Graphite is first placed into some kind of liquid suspension medium, often ethanol, alcohol or some other kind of solvent, and then sonicated either through bath sonication or tip sonication. In bath sonication, the graphite-liquid suspension is usually placed into some kind of container before being placed in the bath sonicator. In tip sonication, the tip is inserted directly into the liquid. The suspension is then sonicated for a period of time. During the sonication, cavitation within th eliquids will cause the different graphene layers to exfoliate from one another. After a certain period of time, the suspensions are removed from the sonicator and then centrifuged. The supernatent of the liquid is then taken and characterised to confirm the presence of mono-layer or few-layer graphene. Surfactants can be utilised to maintain or improve the suspension but might cause issues with final device properties.

Question 8

What are the steps involved to synthesize graphene via laser ablation?

Gemini

The steps involved in synthesizing graphene via laser ablation are:

1. **Prepare the Carbon Target:** A solid carbon-based material (e.g., graphite, carbon nanotubes, polymers) is chosen as the target for ablation.

2. **Set Up Laser System:** A high-power pulsed laser (e.g., excimer, Nd:YAG, femtosecond laser) is focused onto the surface of the carbon target.

3. **Ablation in Controlled Environment:** The laser beam strikes the target in a controlled environment, typically an inert gas atmosphere (e.g., argon, helium) or a liquid medium (e.g., water, organic solvent). The laser energy instantly vaporizes and ablates material from the target surface, creating a plasma plume.

4. **Nucleation and Growth:** Within the plasma plume, carbon atoms and clusters cool rapidly, nucleating and growing into graphene flakes or quantum dots.

5. **Collection:** The synthesized graphene is then collected from the surrounding medium (e.g., by filtration if in liquid, or deposition on a substrate if in gas).

Ground truth

Porous graphene can be made using the laser ablation of a nickel-graphite target under the flow of Argon gas. Although not technically a traditional graphene layer, porous graphene is a similar carbon allotrope with an inhomogenous structure and curvature on the graphene layers. First graphite and nickel particles are ball milled and then pressed into a graphite-nickel pellet under pressure to form a target. The target is then placed into a quartz tube where argon is allowed to flow at about 2.4l/min. The tube is then heated to 1050 degrees celsius and presurised at 300mbar. A Nd-YAG laser (1064nm in wavelength), with a 10 ns pulse duration and repetition rate of 10Hz is then focused on the carbon pellet. The illuminater area is about 12 mm2. The carbon atoms generated by this ablation are carried by the argon gas and then gathered on a collector on a water-cooled surface at the end of the tube and scraped off as porous graphene.

Question 9

What are the steps involved to produce graphene oxide?

Gemini

The production of graphene oxide typically involves the following steps:

1. **Oxidation of Graphite:** Graphite powder is treated with strong oxidizing agents (e.g., sulfuric acid, nitric acid, potassium permanganate, hydrogen peroxide) to intercalate and oxidize the graphitic layers, introducing oxygen-containing functional groups (hydroxyl, epoxy, carboxyl, carbonyl) onto the basal planes and edges. This process often uses a modified Hummers method.

2. **Exfoliation:** The oxidized graphite (graphene oxide paper) is then dispersed in water, often with sonication or vigorous stirring, to overcome the weak van der Waals forces between the highly functionalized layers. This causes the individual graphene oxide sheets to separate and disperse into a colloidal suspension.

3. **Washing and Purification:** The resulting suspension is subjected to extensive washing (e.g., centrifugation, decantation, dialysis) to remove unreacted chemicals, residual acids, and salts. This process continues until the pH of the supernatant becomes neutral.

4. **Drying:** The purified graphene oxide dispersion can be dried to obtain solid graphene oxide. This can be done through various methods like freeze-drying, spray-drying, or vacuum drying, depending on the desired morphology and application.

Ground truth

Graphene oxide (GO) can be synthesized by the oxidation of graphite into graphite oxide followed by the exfoliation of graphite oxide into GO. The leading method right now is the modified Hummer's method. A common procedure is as followws: First, mix graphite and potassium permanganate in a 1: 6 ratio by weight. Then, in another beaker, mix 96% sulphuric acid and then add 75% phosphotic acid. The acids should be in a 6:1 ratio. Ensure the acids are added to the graphite and potassium permanganate slowly and bit by bit while constantly stirring. This allows for the intercalation of the graphite and oxidation of the graphite into graphite oxide. Stir the entire solution at 50 degrees centigrade for 12 hours and then allow to cool to room temperature. Next, pour the entire acid mixture into another beaker with ice made from de-ionised water. Add about 3mL of 30% hydrogen peroxide to the mixture and stire to allow the GO to exfoliate.

Question 10

What are the steps involved to produce reduced graphene oxide?

Gemini

The steps involved to produce reduced graphene oxide (rGO) are:

1. **Oxidation of graphite:** Graphite is oxidized using strong acids and oxidizing agents (e.g., modified Hummers' method) to create graphene oxide (GO). This process introduces various oxygen-containing functional groups on the graphite layers.

2. **Exfoliation of graphene oxide:** The as-synthesized graphene oxide is exfoliated, typically through sonication in a solvent like water, to yield individual GO sheets.

3. **Reduction of graphene oxide:** The exfoliated graphene oxide is then subjected to a reduction process (chemical, thermal, or electrochemical) to remove most of the oxygen-containing functional groups and restore the graphene-like structure, resulting in reduced graphene oxide.

Ground truth

After GO is produced from the modified Hummer's method the GO can be reduced chemically using a reducing agent such as hydrazine, ascorbic acid or even sugars to reduce it to reduced graphene oxide (rGO). Alternatively, GO can be thermally reduced by heating it up at high temperature in an inert atmosphere like argon or in the presence of hydrogen. This will break down the oxygen-containing groups on the GO causing them to volatilize and restore the carbon framework. it should be nmoted that this process is often incomplete and bcan produce rGOP with a lot of defects that affect conductivity.

Question 11

"What are the steps involved to produce graphene quantum dot?

Gemini

The production of graphene quantum dots (GQDs) can be achieved through various methods. A common and relatively straightforward method involves the hydrothermal synthesis from a carbon-rich precursor:

1. **Precursor Solution Preparation:** Dissolve a carbon-containing precursor (e.g., citric acid, glucose, or biomass) in a suitable solvent, typically deionized water.

2. **Hydrothermal Treatment:** Transfer the precursor solution into a sealed reactor (autoclave) and heat it to an elevated temperature (e.g., 160-200 °C) for a specific duration (e.g., 4-12 hours). This high-temperature, high-pressure environment carbonizes and fragments the precursor into quantum-sized carbon structures.

3. **Cooling and Purification:** After the reaction, allow the reactor to cool naturally. The resulting solution is then typically purified by centrifugation to remove larger aggregates or unreacted precursor particles. Further purification, such as dialysis, may be employed to remove small molecular byproducts and achieve better monodispersity, yielding the final graphene quantum dot solution.

Ground truth

Graphene Quantum Dots (GQDs) can be produced using hydrothermail reactions, laser ablation, microwave radiation and electrochemical oxidation. THe most straightforward is that of electrochemical oxidation. The electrochemical oxidation process involves the electrochemical cleavage of carbon precursores such as graphite into GQDs, typically under high voltage. First a potential is applied to a graphite electrode immersed in an electrolyte made of citric acid and sodium hydroxide. A second graphite rod is used as an cathode in the same solution. A DC power supply can provide the working bias voltage. The GQDs eventually exfoliate and dispersed into the electrolyte. The resulting GQDs can be purified by centrifugation and further techniques such as rotary evaporation.

Question 12

What are the steps involved to produce graphene nanoribbon?

Gemini

The production of graphene nanoribbons typically involves the following steps, categorized by approach:

1. **Top-down approaches (from larger graphene or carbon structures):**

* **Unzipping Carbon Nanotubes:** This involves longitudinally cutting open the sidewalls of multi-walled or single-walled carbon nanotubes, often through chemical (e.g., potassium permanganate in acid) or plasma etching methods.

* **Lithographic Patterning and Etching of Graphene:** A larger graphene sheet (e.g., CVD-grown or exfoliated) is covered with a resist, patterned using electron beam lithography or photolithography, and then etched (e.g., oxygen plasma, reactive ion etching) to define the ribbon shapes.

2. **Bottom-up approaches (from molecular precursors):**

* **On-Surface Synthesis:** Specifically designed molecular precursors are deposited onto a catalytic surface (e.g., Au(111)). Through a series of thermally induced reactions (e.g., Ullmann coupling, cyclodehydrogenation), these molecules polymerize and cyclize to form precisely defined graphene nanoribbons with atomic precision.

Ground truth

Graphene nanoribbons (GNRs) are narrow strips of single-layer graphene, essentially 'slices' of graphene that are one-dimensional. They have very high surface areas. They can be synthesized either by top-down approaches such as micromechanical cleavage or bottom-up approaches such as CVD. Top down approaches usually use multi-walled Carbon nanotubes (MWCNTs) to form these graphene nanoribbons. For instance usuing the hummers method on MWCNTs to form the ribbons instead of bulk graphite. Bottom up-approaches using CVD have highly-controlled conditions for growth to ensure orientation and length.

Question 13

What is a method of exfoliating graphene from highly oriented pyrolytic graphite (HOPG) crystal?

Gemini

Mechanical exfoliation (e.g., using adhesive tape) is a method of exfoliating graphene from highly oriented pyrolytic graphite (HOPG) crystal.

Ground truth

HOPG is essentially highly oriented graphite which is ideal for producing graphene using mechanical cleavage with adhesive tape. HOPG can be purchased commercially and then exfoliated through existing processes such as micromechanical exfoliation tape to reult in high-quality graphene with minimal defects.

Question 14

How to synthesize graphene using PET bottles?

Gemini

Graphene can be synthesized from PET bottles primarily through a process called **flash Joule heating**.

This method involves:

1. **Preparation:** Shredding or grinding the PET plastic into a fine powder.

2. **Heating:** Applying a very rapid, high-intensity electrical current through the PET powder (often mixed with a conductive carbon source).

3. **Transformation:** The intense heat (thousands of Kelvin) generated by the electrical discharge causes the PET to instantly decompose and its carbon atoms to reassemble into turbostratic graphene flakes.

Ground truth

Waste PET can be cleaned and then used as a precursor for pyrolysis into graphene. It usually takes a 2 step process. First, pyrolysis at 900 degrees centigrade into amorphous carbon. Next, the amorphous carbon is mixed with Boron powder and heat treated at 2400 degrees celsius for 1 hour under helium gas to form graphite. This high-temperature treatment with boron enables the carbon atoms to rearrange into the layered structure of graphite.. Yield rates are about 86%. This graphite can then be liquid-phase exfoliated to form graphene.

Question 15

What is the purpose of PMMA in the synthesis of graphene?

Gemini

PMMA (polymethyl methacrylate) serves as a **sacrificial support layer** during the transfer of graphene from its growth substrate (e.g., copper foil) to a target substrate. It provides **mechanical stability** to the delicate graphene film, preventing tearing or damage during the transfer process, and is subsequently dissolved away after transfer.

Ground truth

PMMA can be pyrolised to decompose into carbon-rich material which can then form graphene sheets through cvd or some other pyrolytic process. PMMA can also be used as a transfer agent to protect and transfer graphene to other surfaces after growth on copper.

Question 16

What is the purpose of plasma cleaning in preparing substrates for high-quality graphene growth?

Gemini

The purpose of plasma cleaning is to remove organic and inorganic contaminants from the substrate surface. This process creates a clean, active, and uniform substrate, which is essential for promoting consistent nucleation, strong adhesion, and the formation of defect-free, high-quality graphene.

Ground truth

Plasma cleaning removes contaminants, organic reside, enhances surface wettability and improves the adhesion of graphene to the substrate.

Question 17

Could you elaborate on the role of copper foil in the synthesis of graphene films using the chemical vapor deposition method?

Gemini

In the chemical vapor deposition (CVD) synthesis of graphene films, copper foil serves a crucial dual role:

1. **Catalyst:** Copper facilitates the decomposition of carbon-containing precursor gases (e.g., methane) at elevated temperatures. It lowers the activation energy required for the precursor molecules to break down into reactive carbon species.

2. **Substrate:** It provides a surface for these reactive carbon species to diffuse, nucleate, and grow into a graphene layer. Copper's low carbon solubility at typical CVD growth temperatures (around 1000°C) is critical; this ensures that carbon atoms primarily precipitate and form a layer *on its surface* rather than dissolving deeply into the bulk of the metal. This surface-limited growth mechanism is essential for forming high-quality, typically monolayer or few-layer graphene films.

After growth, the copper foil can be selectively etched away, allowing the synthesized graphene film to be transferred to another desired substrate.

Ground truth

The copper foil usually acts as a substrate and catalyst for growing graphene layers in CVD. It provides a surface on which carbon atoms can nucleate and arrange themselves into a graphene structure.

Question 18

What role does H_2O_2 play in the synthesis of graphene oxide?

Gemini

H_2O_2 acts as an oxidizing agent and is involved in the exfoliation of graphite layers during the synthesis of graphene oxide.

Ground truth

It reduces excess oxidisers such as potassium permanganate in variants of the Hummer's method, thus halting oxidation and preventing uncontrolled reactions. It also can promote the formation of grpahene oxide by facilitating the unravelling of carbon chains.

Question 19

Which method would u recommend for synthesizing monolayer graphene for producing transistors?

Gemini

For synthesizing monolayer graphene for producing transistors, **Chemical Vapor Deposition (CVD)** is highly recommended.

Reasons:

* **High Quality:** Produces large-area, high-quality monolayer graphene with low defect density.

* **Monolayer Control:** Offers good control over the number of graphene layers, which is crucial for consistent transistor performance.

* **Scalability:** Capable of synthesizing graphene over large areas, making it suitable for industrial production.

* **Versatility:** The synthesized graphene can be transferred to various substrates commonly used in transistor fabrication (e.g., SiO2/Si).

Ground truth

Micromechanical Cleavage.

Question 20

Which method would u recommend for synthesizing few layer graphene for dispersion into inks?

Gemini

Liquid Phase Exfoliation (LPE) or Electrochemical Exfoliation would be recommended for synthesizing few-layer graphene for dispersion into inks.

Ground truth

Pyrolysis

S6 Answer set for RAG-Qwen

Question 1

What are the steps involved to synthesize graphene via chemical vapour deposition?

Context 1

Author: Sukang Bae

Doi:10.1038/nnano.2010.132

Method:

An 8-inch-wide tubular quartz reactor is used in the CVD system, allowing a monolayer graphene film to be synthesized on a roll of copper foil with dimensions as large as 30 inches in the diagonal direction. A temperature gradient usually exists that depends on the radial position inside the tubular reactor. In our preliminary work, this sometimes resulted in inhomogeneous growth of the graphene on the copper foils. To solve this problem, a ~7.5-inch quartz tube wrapped with a copper foil was inserted and suspended inside the 8-inch quartz tube. In this way, the radial inhomogeneity in the reaction temperature could be minimized. In the first step of synthesis, the roll of copper foil is inserted into a tubular quartz tube and then heated to 1,000 °C with flowing 8 s.c.c.m. H_2 at 90 mtorr. After reaching 1,000 °C, the sample is annealed for 30 min without changing the flow rate or pressure. The copper foils are heat-treated to increase the grain size from a few micrometres to ~100 μm, as we have found that the copper foils with larger grain size yield higher-quality graphene films. The gas mixture of CH_4 and H_2 is then flowed at 460 mtorr with rates of 24 and 8 s.c.c.m. for 30 min, respectively. Finally, the sample is rapidly cooled to room temperature (~10 °C s^{-1}) with flowing H_2 under a pressure of 90 mtorr.

Context 2

Author: Carlos Diaz-Pinto

Doi: 10.1021/nn300326x

Method:

The chemical vapour deposition (CVD) growth was carried out under ambient pressure in a 1" quartz tube furnace, using 25μm-thick copper foil (Alpha Aesar, item no. 13382). At room temperature, argon gas was used to flush the quartz tube at a rate of 2L/min for at least 15 minutes. The Ar flow was then stopped, a hydrogen flow of 200mL/min was started, and the temperature was raised to 1000°C in approximately 18 minutes. After holding at 1000°C for 20 minutes, the H_2 flow was stopped and replaced by an Ar flow of 150mL/min, maintained for another 20 minutes. Then, a methane flow of 13mL/min was introduced along with the continued Ar flow of 150mL/min for a typical graphene growth time of 10 minutes. After the growth phase, the CH_4 flow was turned off and the system was cooled to room temperature over approximately 1.5 hours, while maintaining the Ar flow at 150mL/min.

Context 3

Author: Tianru Wu

Doi: 10.1002/adfm.201201577

Method:

The graphene synthesis was done by ambient pressure chemical vapour deposition (APCVD) at a typical temperature ranging from 950–1050°C for 30–80 min. A mixture of Ar and H_2 was used as reactant gases. The H_2 flow rate was adjustable between 0–10 sccm while keeping the total flow rate at 300 sccm. Carbon supply was controlled by heating the precursor with a halogen lamp under a typical polystyrene temperature (Tp) range of 80–280°C. After the growth process, the halogen tungsten lamp was turned off, and the furnace was cooled down to room temperature naturally.

Context 4

Author: Jian Hao

Doi: 10.1038/s44286-025-00203-z

Method:

Synthesis of CVD graphene: Commercial copper foils were pretreated with nitric acid (4 wt%) for 10 min to remove the surface contamination. Cu foils were washed with deionized water four times and stored in isopropanol before use. A customized CVD furnace (Carbolite, TS1/3-1200, heating zone 1.2 m) hosting a quartz tube and an alumina tube (Zibo Highlion New Material) was built for graphene synthesis. The synthesis protocol of CVD graphene followed the previous reports. First, the Cu foil was treated at 1,020 °C for 2 h under 1 bar CO_2 flow to remove surface carbonaceous contaminations. After exchanging the gas to Ar and H_2 with a flow rate of 500 and 50 sccm, respectively, the furnace was slowly heated to 1,065 °C and the temperature was maintained for 3 h. The graphene synthesis was conducted at 1,020 °C using 9 sccm of CH_4 and 3 sccm of H_2 under a pressure of 180 mTorr for 30 min.

Context 5

Author: A. Kumar

Doi:10.1016/j.carbon.2011.11.033

Method:

Graphene film synthesis was performed in a SEKI AX5200S MPCVD (microwave plasma chemical vapor deposition) system with H_2 (50 sccm) and CH_4 (5 sccm) as the feed gases. A 25 μm thick copper foil (Alfa Aesar, 99.8% purity) was used as the substrate. The foil was cut in 2 x 2 cm^2 pieces and placed into the MPCVD chamber, which was evacuated to a base pressure of 2 Torr and then filled with high-purity hydrogen at a pressure of 10 Torr. The Cu foil was supported by a ceramic stand that elevated it from the Mo puck by about 15 mm. The elevation of the Cu foil above Mo puck was found to be critical for the growth of the films

because it ensures a strong coupling between the plasma and the Cu foil, thus enabling rapid self-heating of the foil by the microwave plasma. No additional heater was used. The thermocouple attached to the substrate table indicated a maximum reading of approximately 65°C during the process. A dual-wavelength infrared pyrometer indicated a maximum temperature of 700 ± 25 °C. However, as the copper foil is coated with graphene, the surface emissivity changes, making reliable IR measurements difficult. The entire growth is accomplished in less than 5 min and generally involves two stages. First, in a plasma cleaning and annealing step, the foil is kept in 400 W hydrogen plasma for 3 min. A H2 flow rate of 50 sccm was used with downstream pressure control to maintain 10 Torr chamber pressure throughout the process. This process leads to rapid self-heating and removal of copper oxide from the foil surface. The second stage involves introduction of CH4 (while maintaining the hydrogen plasma) at a flow of 5 sccm for a short duration (between 30 s and 2 min), providing approximately 10% CH4 concentration in hydrogen. Depending on the growth duration and whether or not annealing is used, different forms of carbon can be deposited.

RAG-Qwen

To synthesize graphene via chemical vapour deposition (CVD), several key steps are typically involved across different methods:

1. **Preparation of Copper Foil**: The copper foil is often cleaned and pretreated to remove contaminants. This may include treating the foil with nitric acid or other chemicals, washing it with deionized water, and storing it in isopropanol.

2. **Furnace Setup**: The copper foil is inserted into a CVD furnace, which is equipped with a quartz or alumina tube. The furnace is evacuated to a low pressure and then filled with a carrier gas, usually hydrogen (H2).

3. **Temperature Control**: The furnace is heated to a specific temperature, which can vary depending on the method, typically ranging from 950°C to 1065°C. The temperature is maintained for a certain period, often around 30 minutes to several hours.

4. **Gas Flow Introduction**: A mixture of gases is introduced into the furnace. Commonly used gases include hydrogen (H2) and methane (CH4). The flow rates of these gases are carefully controlled. For example, H2 might flow at 8 to 500 sccm, while CH4 might flow at 24 to 9 sccm.

5. **Growth Period**: The graphene synthesis occurs under the controlled conditions of temperature and gas flow. The growth period can last from a few minutes to over an hour, depending on the specific method.

6. **Cooling**: After the growth period, the sample is rapidly cooled to room temperature, often with the carrier gas still flowing.

7. **Post-Growth Processing**: The resulting graphene film on the copper foil may undergo additional processing, such as transfer to another substrate, to prepare it for further characterization or application.

These steps illustrate the general process of synthesizing graphene via CVD, though the exact details can vary between different research groups and methodologies.

Ground truth

First, a substrate, such copper, is cleaned, prepared and placed into a CVD chamber that is usually a tube made out of an inert material such as quartz. After which, the CVD chamber is evacuated into a vacuum or flooded with inert gases.The CVD chamber is then heated to some kind of critical temperature (about 1000 degrees celsius), before feedstock or precursor gases with high hydrocarbon contents are pumped into the CVD chamber. The high temperature in the chamber decomposes these feedstock gases into carbon atoms which then deposit onto the substrate's surface where they reassemble to into the characteristic graphene lattice. The feedstock gases also produce hydrogen which do not contribute to the reaction. The choice of substrate matters as it plays a role in catalysing the reaction. The temperature and duration of this deposition is controlled to either form mono-layer or few-layer graphene.

Question 2

What are the steps involved to synthesize graphene via mechanical exfoliation?

Context 1

Author: Florian R. Ong

Doi: 10.1088/0957-4484/26/40/405201

Method:

Graphene layers are then obtained by mechanical exfoliation of kish graphite onto a separate Si/SiO2 substrate, which we call the exfoliation substrate. Using Si/SiO2 as a substrate enables the discrimination of one to few layer flakes by inspection with an optical microscope. The number of layers is then confirmed by Rama spectrometry, and a monolayer is selected for the transfer step to follow. A solution of cellulose acetate butyrate (CAB) in ethyl acetate 60 mg/mL, which is a hydrophobic polymer, is dripped onto the substrate, covering the graphene monolayer of interest. The latter is still distinguishable under the optical microscope after the CAB polymer dries off. The needles of the micromanipulator are used to punch markers around the monolayer. These markers will later allow for the alignment of the graphene relative to the gate on the destination substrate, onto which graphene is optically indiscernible.

Context 2

Author: Yuan Huang

Doi: 10.1021/acsnano.5b04258

Method:

We used SiO2/Si as the substrate and ordinary adhesive tape as the transfer medium. Contact with the tape transfers thick graphite flakes from a highly oriented pyrolytic graphite (HOPG) crystal. Prior to exfoliating thin graphene from these flakes, the SiO2/Si substrate is ultrasonically cleaned in acetone, 2-propanol, and deionized (DI) water, and then subjected to oxygen plasma to remove ambient adsorbates from its surface. Following the plasma cleaning step, the graphite-loaded tape is brought in contact with the substrate. Instead of immediately removing it to complete the exfoliation, the substrate with the attached tape is annealed for 25 min at~100°C, in air on a conventional laboratory hot plate. After the sample is cooled to room temperature, the adhesive tape is removed, which completes the exfoliation.

Context 3

Author: K. S. Novoselov

Doi: 10.1126/science.1102896

Method:

The starting material was 1-mm-thick platelets of highly -oriented pyrolytic graphite (HOPG). We used commercially available HOPG of grades ZYH (www.ntmdt.ru) and HOPG-1 (www.2spi.com) with μ >100,000 cm2 /Vs at 4K. Using dry etching in oxygen plasma, we first prepared 5 μm-deep mesas on top of the platelets (mesas were squares of various sizes from 20 μm to 2 mm). The structured surface was then pressed against a 1-μm-thick layer of a fresh wet photoresist spun over a glass substrate. After baking, the mesas became attached to the photoresist layer, which allowed us to cleave them off the rest of the HOPG sample. Then, using scotch tape we started repeatedly peeling flakes of graphite off the mesas. Thin flakes left in the photoresist were released in acetone. When a Si wafer was dipped in the solution and then washed in plenty of water and propanol, some flakes became captured on the wafer's surface (as a substrate, we used n+ -doped Si with a SiO2 layer on top; in order to avoid accidental damage - especially during plasma etching - we chose to use relatively thick SiO2 with t =300nm). After this, we used ultrasound cleaning in propanol, which removed mostly thick flakes. Thin flakes (d < 10 nm) were found to attach strongly to SiO2, presumably due to van der Waals and/or capillary forces. To select from the resulting films only those that are just a few graphene layers thick, we used a combination of optical, electron-beam and atomic -force microscopy. Graphitic films thinner than 50 nm are transparent to visible light but nevertheless can easily be seen on the SiO2 surface because of the added optical path that shifts the interference colors. The color for a 300 nm wafer is violet-blue and the extra thickness due to graphitic films shifts it to blue. At thicknesses d less than ~1.5nm, as measured by AFM, graphene films are no longer visible even via the interference shift as it becomes too small. This provides a natural marker that we use to distinguish between two groups of films that we refer to as few- and multi- layer graphene. The graphene films whose properties are reported in the main paper were selected as those that were completely invisible in an optical microscope (OM). Although invisible in optics, few-layer graphene (FLG) can still be seen clearly in a high-resolution SEM (we used FEI Serion). To identify FLG films, we have compared optical and scanning-electron micrographs of large areas on the wafer, trying to find the films visible in SEM but not in OM.

Context 4

Author: Dan Li

Doi: 10.1038/nnano.2007.451

Method:

Graphite oxide was synthesized from natural graphite (SP-1, Bay Carbon) by a modified Hummers method as originally presented by Kovtyukhova and colleagues. As-synthesized graphite oxide was suspended in water to give a brown dispersion, which was subjected to dialysis to completely remove residual salts and acids. Ultrapure Milli-Q® water was used in all experiments. As-purified graphite oxide suspensions were then dispersed in water to create a 0.05 wt% dispersion. Exfoliation of graphite oxide to graphene oxide (GO) was achieved by ultrasonication of the dispersion using a Brandson Digital Sonifier (S450D, 500 W, 30% amplitude) for 30 min. The obtained brown dispersion was then subjected to 30 min of centrifugation at 3,000 r.p.m. to remove any unexfoliated graphite oxide (usually present in a very small amount) using an Eppendorf 5702 centrifuge with a rotor radius of 14 cm. In a

typical procedure for chemical conversion of graphite oxide to graphene, the resulting homogeneous dispersion (5.0 ml) was mixed with 5.0 ml of water, 5.0 µl of hydrazine solution (35 wt% in water, Aldrich) and 35.0 µl of ammonia solution (28 wt% in water, Crown Scientific) in a 20-ml glass vial. The weight ratio of hydrazine to GO was about 7:10. After being vigorously shaken or stirred for a few minutes, the vial was put in a water bath (~95 °C) for 1 h. Unless specifically stated, graphene dispersions prepared according to the above procedure were used for further characterization and film fabrication in this work. The graphene paper was made by filtration of a dispersion through an Anodisc membrane filter (47 mm in diameter, 0.2 µm pore size; Whatman), similar to the method reported for making GO paper. The graphene paper was cut by a razor blade into rectangular strips of approximately 4 mm × 15 mm for mechanical testing.

Context 5

Author: Tim J. Booth

Doi: 10.1021/nl801412y

Method:

Graphene crystals are first prepared by standard micromechanical cleavage techniques . Sufficiently large flakes produced in this way are widely distributed over a substrate (occurring with a typical number density of < 1 per cm2) and in a great minority as compared to thicker flakes. This prevents their identification via atomic-resolution techniques such as scanning probe or electron microscopies either due to prohibitively small search areas or a lack of response specific to single-layer graphene. Fortunately, one-atom thick crystals can still be identified on surfaces covered with thin dielectric films due to a color shift induced by graphene, which allows crystals to be found rapidly with a trained eye and a quality optical microscope. In the current work, we have used Si wafers that, in contrast to the standard approach, are not oxidized but instead covered with a 90 nm thick film of polymethyl methacrylate (PMMA). The optical properties of PMMA are close to those of SiO2, and the visible contrast of graphene is optimal at this particular thickness. The PMMA film also serves later as a sacrificial layer during the final liftoff. Once a suitable graphene crystal is identified in an optical microscope, we employ photolithography to produce a chosen pattern (in our case, a Transmission Electron Microscopy (TEM) grid) on top of graphene (we usually used a double-layer resist consisting of 200 nm polymethyl glutarimide (PMGI) and 200 nm S1805. A 100 nm Au film with a 5 nm Cr adhesion layer is thermally evaporated after developing the resist. Liftoff of the metal film is not performed in acetone, which would destroy the base layer, but in a 2.45 wt % Tetramethylammonium hydroxide (TMAH) solution (MF-319 developer; MicroChem) at 70°C, resulting in a minimal etch rate for PMMA (< 5° Åmin-1). The next step involves another round of photolithography, in which the graphene crystal is remasked with the same photoresist. The mask serves here to protect graphene during electrodeposition, when a thick copper film is electrochemically grown on top of the Au film, repeating the designed pattern. We have chosen a CuSO4/H2SO4 electrolyte because of its low toxicity, resist and substrate compatibility and ease of deposition. Finally, acetone is used to strip the remaining resist, releasing the copper TEM grid with the attached graphene membrane. The sample is dried in a critical point dryer to prevent the membrane rupturing due to surface tension.

RAG-Qwen

The steps involved in synthesizing graphene via mechanical exfoliation typically include the following:

1. **Substrate Preparation**:

- Use a suitable substrate such as Si/SiO_2, SiO_2/Si, or PMMA-coated Si. The substrate helps in identifying and transferring individual graphene layers.

2. **Graphite Source**:

- Obtain graphite source materials like highly oriented pyrolytic graphite (HOPG) or bulk graphite.

3. **Exfoliation**:

- For Si/SiO_2 or SiO_2/Si substrates, use adhesive tape or a micromanipulator with needles to peel off thin flakes from the graphite source.

- For PMMA-coated Si substrates, prepare a pattern on the substrate using photolithography and etch the PMMA layer to expose the graphene.

4. **Transfer**:

- Transfer the graphene flake to the desired substrate using the same adhesive tape or a polymer solution like cellulose acetate butyrate (CAB).

5. **Alignment and Cleaning**:

- Align the transferred graphene using markers or patterns created earlier.

- Clean the substrate to remove any contaminants or residues.

6. **Characterization**:

- Use optical microscopy, electron microscopy, and other techniques to confirm the number of graphene layers and the quality of the transferred graphene.

These steps ensure that the graphene layer is isolated, transferred, and characterized accurately for further use in devices or research applications.

Ground truth

Mechanical exfoliation of graphene, also known as the sticky tape method, involves first taking a piece of graphite, and then using a piece of sticky tape release a small layer of graphite onto it. This is done simply by placing the sticky tape repeatedly on the piece of graphite. To produce single atomic sheets of graphene, this tape is folded in half and repeatedly pulled apart, thereby shearing layers of graphene from the already deposited material until only a single-atomic layer is present.

Question 3

What are the steps involved to synthesize graphene via plasma enhanced chemical vapour deposition (PECVD)?

Context 1

Author: Yong Seung Kim

Doi: 10.1039/C2NR33034B

Method:

The graphene films studied in this work were grown on 25 µm-thick Cu foils by plasma-enhanced chemical vapor deposition (PECVD). The chamber is equipped with an inductively coupled plasma reactor. The radiofrequency (RF) coil, having a diameter of ~25 cm, is located 35 cm above the top surface of the graphite substrate holder. In this geometry, the heating effect of the substrate by plasma was negligible. A residual gas analyzer (RGA 100, Stanford Research System) is attached at the side wall of the chamber to investigate the density of discharged species. A differential pumping technique is employed to meet the required working pressure of the RGA. The system is pumped with a turbomolecular pump (Osaka Vacuum LTD, TG1003), keeping the base pressure as low as ~10^-7 Torr. Polycrystalline Cu foil was cut into 7 x 7 cm2 pieces and mounted in the chamber without any pre-cleaning treatment. Five different stages were employed to synthesize graphene films on Cu foil using methane (CH_4) as the carbon source. The Cu substrate was heated to the growth temperature (700–830 °C) at a heating rate of 3 °C/s. When it reached the target temperature, H_2 gas was introduced into the chamber at a flow rate of 40 standard cubic centimeters per minute (sccm). Hydrogen gas was discharged by an RF power of 50 W for 2 minutes to eliminate surface oxides on the copper foil. Then, the chamber was purged with Ar at a flow rate of 100 sccm for 2 minutes to remove residual hydrogen gas. During the graphene growth stage, radiofrequency (RF) plasma was generated for a specified growth time under a continuous flow of argon (or hydrogen, 40 sccm) and methane (1 sccm), while the pressure was kept at 10 mTorr. The plasma power was varied from 10 to 200 W and the growth time from 0.2 to 4 minutes. Subsequently, the sample was cooled down rapidly to room temperature at a cooling rate of 3 °C/s by turning off the heating power, and then it was taken out for characterization.

Context 2

Author: A. Kumar

Doi: 10.1016/j.carbon.2011.11.033

Method:

Graphene film synthesis was performed in a SEKI AX5200S MPCVD (microwave plasma chemical vapor deposition) system with H_2 (50 sccm) and CH_4 (5 sccm) as the feed gases. A 25 µm thick copper foil (Alfa Aesar, 99.8% purity) was used as the substrate. The foil was cut in 2 x 2 cm2 pieces and placed into the MPCVD chamber, which was evacuated to a base pressure of 2 Torr and then filled with high-purity hydrogen at a pressure of 10 Torr. The Cu

foil was supported by a ceramic stand that elevated it from the Mo puck by about 15 mm. The elevation of the Cu foil above Mo puck was found to be critical for the growth of the films because it ensures a strong coupling between the plasma and the Cu foil, thus enabling rapid self-heating of the foil by the microwave plasma. No additional heater was used. The thermocouple attached to the substrate table indicated a maximum reading of approximately 65°C during the process. A dual-wavelength infrared pyrometer indicated a maximum temperature of 700 ± 25 °C. However, as the copper foil is coated with graphene, the surface emissivity changes, making reliable IR measurements difficult. The entire growth is accomplished in less than 5 min and generally involves two stages. First, in a plasma cleaning and annealing step, the foil is kept in 400 W hydrogen plasma for 3 min. A H2 flow rate of 50 sccm was used with downstream pressure control to maintain 10 Torr chamber pressure throughout the process. This process leads to rapid self-heating and removal of copper oxide from the foil surface. The second stage involves introduction of CH4 (while maintaining the hydrogen plasma) at a flow of 5 sccm for a short duration (between 30 s and 2 min), providing approximately 10% CH4 concentration in hydrogen. Depending on the growth duration and whether or not annealing is used, different forms of carbon can be deposited.

Context 3

Author: Lam Van Nang

Doi: 10.1149/2.082204jes

Method:

To investigate the rf plasma effect, graphene growth was carried out with a diluted CH4 gas, in which graphene was not formed without applying plasma. The CH4 gas of 1 SCCM (denoting cubic centimeter per minute at STP) and the mixed gases of 10% H2 and 90% Ar (forming gas) of 100 SCCM were introduced in a hot-wall tubular reaction chamber. The Cu foil (Alfa Aesar) was pre-treated with an acetic acid solution at 60°C for 10 min, followed by washing with de-ionized water. The foil was heated to a graphene synthesis temperature (950°C) and annealed for 10 min with forming gas flow (100 SCCM) to remove native oxides from the Cu foil and to enlarge its grains. The synthesis pressure was fixed at 1 Torr. The synthesis time and rf plasma power is 5min and 200 W, respectively. The synthesized graphene on Cu was transferred on SiO2/Si or glass substrate for various characterizations. First, a 500 nm layer of poly-methyl methacrylate (PMMA) was spin coated on the surface of the graphene film to serve as support. The graphene layer at the back side of the Cu foil was then etched off using oxygen plasma. After the etching of the Cu foil with FeCl3 aqueous solution at 40°C, the PMMA/graphene was transferred to a target substrate. The graphene on the SiO2/Si or glass substrate was achieved by dissolving the PMMA film using acetone

Context 4

Author: Alexander Malesevic

Doi: 10.1088/0957-4484/19/30/305604

Method:

Few-layer graphene (FLG) was synthesized in a Microwave plasma-enhanced chemical vapour deposition (MW PECVD) set-up composed of an Iplas Cyrannus MW plasma source mounted on top of a stainless-steel vessel. The plasma source consists of a 6 kW, 2.45 GHz cylindrical MW resonator, which couples the MW power through five annular slots in a quartz tube and enables operation either in the TM012 or in the surface wave plasma mode. For all the experiments, the plasma was operated in the TM012 mode. A flexible substrate holder allows free positioning of substrates along the axis of the quartz tube. A broad range of materials was used as substrates, including quartz, silicon, nickel, platinum, germanium, titanium, tungsten, stainless steel, tantalum and molybdenum. Prior to growth, the substrates are heated for 20 min in a 2 kW MW plasma at 40 Torr, using a H_2 flow of 200 sccm. This plasma is sufficiently powerful to heat the samples from room temperature up to 700°C, as measured by a thermocouple embedded in the substrate holder. Immediately after the heating step, CH_4 mixed with H_2 is introduced into the growth chamber with a CH_4/H_2 ratio of 1/8 at a total flow rate of 200 sccm. Keeping the plasma power and pressure at 2 kW and 40 Torr, respectively, FLG was grown with this gas mixture for various growth times ranging from 1 to 3000 s.

Context 5

Author: D.A. Boyd

Doi: 10.1038/ncomms7620

Method:

The copper substrates were placed on a quartz flat inside of quartz tube. A typical substrate size was (8×13) mm^2. The tube was evacuated to 25 to 30 mTorr. A 2-5 sccm flow of room temperature hydrogen gas with 0.4% methane and a comparable amount of nitrogen gas was added and the pressure was controlled at 500 mTorr. The addition of methane to the gas flow was controlled by a precision leak valve, and a typical concentration, as measured by gas chromatography, was 0.4 %. The initially low vacuum conditions (~ 25 mTorr) provided sufficient partial pressures of nitrogen (atmospheric) for the plasma enhanced chemical vapour deposition (PECVD) process. (We note that a leak valve for including purified nitrogen was added to the setup to study the effects of increased nitrogen partial pressures.) Typical partial pressures measured by residual gas analysis (RGA) are as follows: N_2 (Mass 28): 6.9×10^{-8} Torr, CH_4 (Mass 16): 1.5×10^{-7} Torr , O_2 (Mass 32): 5.4×10^{-9} Torr , H_2O (Mass 18): 1.2×10^{-6} Torr, CO_2 (Mass 44): 2.3×10^{-8} Torr. From these values we estimate that the concentration of nitrogen in the gas flow is typically on the same order as that of methane. The PECVD process was found to be highly sensitive to the relative amounts of methane and nitrogen. Excessive methane mixtures resulted in no etching, while excessive nitrogen mixtures would result in excessive etching of the copper. Slight adjustments to the methane concentration could be made via either optical emission spectroscopy (OES) or RGA. A low-power (ranging from 10 to 40 W) cold hydrogen plasma was formed over the copper substrate using an Evenson cavity. Exposure of copper substrates to the plasma enabled continuing etching and cleansing of the copper surface during the graphene growth process. The gas temperature inside the plasma was measured using a thermocouple sheathed in boron nitride. The thermocouple was placed in the plasma above the sample and could be translated along the tube. The peak gas temperature measured in plasma treatment was 160°C at 10 W and 425°C at 40 W, and the gas temperature profile decreased rapidly (by 120°C at 10 W and 425°C at 40 W within 1 cm) from the peak value. The maximum

temperature of the copper substrate (Ts) was measured using the melting point of known solids, lead and zinc, and found to be within the range of 327.5°C < Ts < 419.5°C at 40 W. Typically after 5 to 20 minutes of direct exposure to the plasma, a large-area monolayer graphene formed on the backside of copper substrates while the front side of the substrates was coated with disordered graphite. Upon the completion of graphene growth, the plasma was extinguished, and the gas flows were stopped. The process tube was then evacuated and back filled with Ar, and the substrate with graphene coating was subsequently removed. Copper deposition was visible on the inside of the tube and on the sample holder as the result of plasma etching of the copper substrates.

RAG-Qwen

Synthesizing graphene via plasma-enhanced chemical vapor deposition (PECVD) involves several key steps, which can vary slightly depending on the specific experimental setup and conditions. Here is a general outline based on the methods described in the provided contexts:

1. **Preparation of the Copper Substrate**:

- Clean the copper foil using an appropriate method such as acetic acid treatment or simply washing with deionized water. This step helps remove any contaminants that might interfere with the graphene growth process.

2. **Substrate Heating and Plasma Cleaning**:

- Heat the copper foil to a specific temperature (often around 700-950°C) in a hydrogen plasma. This step is crucial for removing surface oxides and enhancing the growth of graphene.

- The plasma cleaning step typically uses hydrogen gas at a flow rate of 50-400 sccm, and the process can last from a few minutes to tens of minutes.

3. **Introduction of Carbon Source Gas**:

- Introduce methane (CH_4) gas into the reaction chamber at a controlled flow rate (usually 1-10 sccm).

- Maintain a suitable pressure in the chamber, often around 10-100 mTorr.

4. **Plasma Generation and Growth**:

- Generate plasma using a radiofrequency (RF) or microwave power supply. The power level and duration can vary widely, from 10 W to 200 W, and the growth time can range from 0.2 to 4 minutes.

- The presence of hydrogen in the plasma helps in the decomposition of methane and the formation of graphene.

5. **Cooling and Sample Removal**:

- Rapidly cool the substrate to room temperature after the growth process is completed.

- Remove the substrate from the reaction chamber and prepare it for further characterization.

6. **Post-Growth Processing** (if necessary):

- Transfer the graphene-coated substrate to another substrate for further studies.

- Etch off the graphene layer from the backside of the copper substrate if needed.

These steps are broadly consistent across the different methods described in the provided contexts, though the specific details of the equipment used and the exact parameters can differ.

Ground truth

The plasma-enhanced process is a modification of traditional chemical vapour deposition. As with traditional CVD, a prepared substrate (cleaned and heated to about 1000 degrees celsius) is inserted into a a CVD chamber made out of an inert material and gaseous precursors such as methane are introduced into the reaction chamber together with other inert gases such as H2 or Ar. Often plasma-enhanced CVD can use lower temperatures than traditional CVD. A plasma, typically generated by radio frequency energy at 13.56Mhz is used to excite and disassociate the precursor gases. This process creates highly reactive carbon radicals which deposit and self-assemble ont he heated substreate forming graphene layers. The growth can be tailored by adjusting parameters such as gas flow rates, plasma power and time.

Question 4

What are the steps involved to synthesize graphene via electrochemical exfoliation?

Context 1

Author: Khaled Parvez

Doi: 10.1021/ja5017156

Method:

Electrochemical exfoliation of graphite: Natural graphite flakes were used as a carbon electrode (i.e., anode) for electrochemical exfoliation of graphite. The graphite flakes adhered to a conductive carbon tape, forming a pellet. A Pt wire was used as a cathode. The electrolyte for the exfoliation was prepared by dissolving 1.06 g (NH4)2SO4 in 80 mL H2O (i.e., 0.1 M). The distance between the graphite and the Pt electrode was ~2 cm and was kept constant throughout the electrochemical process. Electrochemical exfoliation was carried out by applying positive voltage (10 V) to the graphite electrode. After the graphite exfoliation was completed, the product was collected through a PTFE membrane filter with 0.2 m pore size and washed several times with deionised water by vacuum filtration. The resultant exfoliated graphene (EG) was then dispersed in DMF by sonication at low power for 10 min. The dispersion was maintained for 48 h to precipitate unexfoliated graphite flakes or particles. The top part of the dispersion was used for characterisation and device fabrication. For largescale production of EG, graphite foil (Alfa Aesar) was used instead of flakes.

Context 2

Author: Khaled Parvez

Doi: 10.1021/nn400576v

Method:

Electrochemical exfoliation process: Natural graphite flakes (Sigma Aldrich) were used as carbon source (working electrode) for electrochemical exfoliation. The graphite flakes were adhered on a conductive carbon tape to form a pellet and then immersed into the H2SO4 solution (Sigma Aldrich; 95-97%). A Pt wire was used as a counter electrode. The electrochemical exfoliation of graphite was carried out by applying positive voltage (+10 V) on the working electrode. The exfoliated graphene (EG) was then collected with a polytetrafluoroethylene (PTFE) membrane filter (pore size 0.2 μm) and washed repeatedly with DI water by vacuum filtration. The resultant EG was dispersed in N,N'-dimethylformamide (DMF) by sonication at low power for 10 min. The dispersion was kept for 24 h for the precipitation of unexfoliated graphite flakes and/or particles. The top part of the dispersion was used for further characterization and device fabrication.

Context 3

Author: Guoxiu Wang

Doi: 10.1016/j.carbon.2009.07.040

Method:

High purity graphite rods (6 mm, 99.999%, Aldrich) were used as electrodes. Poly(sodium-4-styrenesulfonate) (PSS, Mw = 70,000, Aldrich) was dissolved in de-ionized (DI) water to form the electrolyte (0.001 M). In a typical synthesis, two graphite rods were placed in an electrolysis cell filled with the electrolyte. A constant potential of 5 V (DC voltage) was applied to the two electrodes (CHI 660 C Electrochemical Workstation). After 20 min electrolysis, black product gradually appeared at the positive electrode (anode). The exfoliation continued for 4 h. Then the product (a dispersion) was taken from the electrolysis cell. The dispersion was centrifuged at low speed (1000 rpm) to remove large agglomerates. The top of the dispersion was then decanted. This graphene–PSS suspension is very stable in nature. After 6 months storage, there is no any precipitation. To obtain dry graphene powders, the dispersion was washed with DI water and ethanol, and then dried in a vacuum oven at 80°C. The yield of graphene was estimated by weighting the dried graphene powders and the dried sediment. Our electrolytic exfoliation method results in producing graphene at a yield at about 15 wt.%.

Context 4

Author: Sheng Yang

Doi: 10.1021/jacs.5b09000

Method:

Graphite exfoliation was conducted in a home-built setup that used rolled graphite foils as working electrodes and platinum foils as counter electrodes. An aqueous solution containing ammonium sulphate with (2,2,6,6-tetramethylpiperidin-1-yl)oxyl (TEMPO) was tested as an electrolyte. Before exfoliation, the graphite foil was pretreated by alternately soaking it in liquid nitrogen and absolute ethanol. During this process, violent nitrogen evolution and ethanol solidification promoted the expansion of the graphite foils. The dilation was extremely rapid (within 10 s) and yielded a drastic volume increase at the graphite boundary (∼10 times), which facilitated the expansion of the graphite layers, as did the following anion intercalation steps. When the graphite anodes were immersed in the electrolyte, by applying a static bias of 10 V, they detached into small pieces in less than 10 s, spreading on top of the electrolyte and/or on the bottom of the electrochemical cell. After filtration and copious washing, these exfoliated graphene sheets were collected and then dispersed in DMF via mild sonication. A stable dispersion with a concentration of ∼0.3 mg/mL was obtained that was stable for 2 weeks without apparent aggregation.

Context 5

Author: Qingbin Zheng

Doi: 10.1021/nn2018683

Method:

Five grams of natural graphite flakes (Asbury Graphite Mills) and 150 mL of sulfuric acid (H2SO4, General Chemical) were mixed and stirred in a round-bottom flask at a speed of 200 rpm. Fifty milliliters of fuming nitric acid was added into the mixture. The mixture was kept at room temperature and stirred for 24 h. Two-hundred milliliters of deionized water was then poured slowly into the mixture. The resultant mixture was washed using DI water, followed by centrifugation and drying at 60 °C for 24 h to obtain graphite intercalation compound. The dry graphite intercalation compound powder was thermally expanded at 1050 °C for 15 s to obtain expanded graphite that was used for the production of graphene oxide (GO). One gram of expanded graphite and 200 mL of sulfuric acid were mixed and stirred in a three-neck flask. Ten grams of KMnO4 was added to the mixture while stirring. The solution was transferred into an ice bath, and 200 mL of deionized water and 50 mL of H2O2 were poured slowly into the mixture, realizing color change of the suspension to light brown. The GO particles were washed and centrifuged with a HCl solution three times, then centrifuged again and washed with deionized water until the pH of the solution became about 5 to 6. The GO particles obtained thereby were diluted using DI water (~1 mg/mL) and dispersed by gentle shaking.

RAG-Qwen

To synthesize graphene via electrochemical exfoliation, follow these general steps:

1. **Preparation of Electrodes**: Use natural graphite flakes as the working electrode and a platinum wire as the counter electrode. The graphite flakes are adhered to a conductive carbon tape to form a pellet.

2. **Electrolyte Preparation**: Prepare an electrolyte solution, such as a 0.1 M ammonium sulfate (NH4)2SO4 solution in water or a mixture of concentrated sulfuric acid (H2SO4) and fuming nitric acid (HNO3).

3. **Electrochemical Exfoliation**: Apply a positive voltage (typically 10 V) to the graphite electrode while immersing it in the electrolyte. Maintain a consistent distance (about 2 cm) between the graphite and the counter electrode during the process.

4. **Collection of Exfoliated Graphene**: After the exfoliation process is complete, collect the resulting graphene using a PTFE membrane filter with a pore size of 0.2 μm. Wash the collected graphene with deionized water using vacuum filtration.

5. **Dispersal**: Disperse the collected graphene in a suitable solvent like N,N'-dimethylformamide (DMF) by sonication at low power for 10 minutes. Allow the dispersion to settle for a period ranging from 24 hours to 48 hours to precipitate any unexfoliated graphite flakes or particles.

6. **Characterization and Device Fabrication**: Use the top part of the dispersion for further characterization and device fabrication.

These steps can vary slightly depending on the specific method and conditions used, but they generally follow this sequence for electrochemical exfoliation of graphene.

Ground truth

First, an electrochemical set up is made involving a graphite working electrode, a counter electrode, a referene electrode and electrolyte and a power supply. All electrodes are inserted into the liquid electrolyte. Using the power supply, either a positive or negative potential is applied to the graphite electrode which either attracts anions (positive potential) or cations (negative potential) from the graphite electrode which cases expansion. The graphene can be subsequently exfoliated by mixing in the precursor.

Question 5

What are the steps involved to synthesize graphene via arc discharge?

Context 1

Author: Zhiyong Wang

Doi: 10.1088/0957-4484/21/17/175602

Method:

The electric arc oven for synthesis of graphene mainly comprises two electrodes and a steel chamber cooled by water. The cathode and anode were graphite rods (purity: 99.99%) with diameters of 8 and 6 mm, respectively. The current in the discharge process was maintained at 100 A. Initial air pressures of 1000 Torr was employed . The pressure in the oven was increased to a certain level during the discharge process because of the heating effect. A 10 cm long anode graphite rod was consumed in a period of ~10 min. After the consumption of the anode, flue products deposited on the inner wall of the chamber were collected. Several tens of grams of products were obtained per day. Heat treatment in air was performed to purify the products. The sample was heated in air from room temperature to 600°C for 2h and then was cooled down to room temperature.

Context 2

Author: Tianru Wu

Doi: 10.1002/adfm.201201577

Method:

The graphene synthesis was done by ambient pressure chemical vapour deposition (APCVD) at a typical temperature ranging from 950–1050°C for 30–80 min. A mixture of Ar and H2 was used as reactant gases. The H2 flow rate was adjustable between 0–10 sccm while keeping the total flow rate at 300 sccm. Carbon supply was controlled by heating the precursor with a halogen lamp under a typical polystyrene temperature (Tp) range of 80–280°C. After the growth process, the halogen tungsten lamp was turned off, and the furnace was cooled down to room temperature naturally.

Context 3

Author: Maxim G. Rybin

Doi: 10.1016/j.cartre.2024.100349

Method:

All experiments were done using the original equipment designed in RUSGRAPHENE company specially for chemical vapour deposition (CVD) synthesis of graphene films. The chamber walls are cooled by water. The main differences of the equipment from the commercial analogs (such as a CVD furnace quartz tube or cold-wall CVD reactors) are the following: 1) 25 µm thickness copper foil with 30 × 110 mm size is heated resistively with the electrical current increasing up to 85 A and voltage around 3 V for heating up to 1050 °C; 2) the temperature during the synthesis is measured through the optical window in the chamber by the double wavelength infrared pyrometer (Mikron M770) directly from the copper substrate. The setup provides an opportunity to control all of the synthesis parameters such as the heating and cooling rates, the temperature of substrate during the experiment, the gas pressure in the chamber and the gases concentrations. As the experimental setup differs from the commercial analogs, the description of synthesis process is needed. The process of graphene synthesis goes in two steps: the preparation of copper foil and the formation of graphene film. At the first stage the preparation of copper substrate means its annealing. The foil was treated by 3 M nitric acid solution before synthesis to etch contamination from the copper surface for 20 s and then the foil with the size of 30 × 110 mm and the thickness of 25 µm is held between the electrodes in the chamber. In our experiment, we do not use gas flows and do not use mass flow controllers. To fill the chamber with the necessary gases, we use manual needle valves and, by controlling the partial pressure in the chamber, set the desired concentration of gases. First, we pump the chamber to vacuum with an oil rotary pump and shut off the pump. Next, we fill the chamber with argon up to 80 millibars and add hydrogen plus 20 millibars. Thus, for the synthesis of graphene, a mixture of gases argon and hydrogen in a ratio of 4:1 under the pressure values of 100 millibars is used. Then the substrate heating by an electrical current passing is started. A linear current growth is monitored by the software. When the substrate temperature reaches the required value (1050 °C), the current increase is stopped and the temperature is held during 1 min. After annealing the copper foil, methane is injected into the chamber in a small amount, also using a manual needle valve. The injection of methane for providing a desired concentration (from 0.36 % to 4.5 %) is started after annealing. For example, at the pressure of 100 millibars, to inject 0.36 % methane, it is necessary to increase the pressure by 0.36 millibars (up to 100.36 millibars). This is achieved by opening the needle valve for 1 s. For a 4.5 % injection (or 4.5 millibars), the needle valve opens slightly longer (for 10 s). The time of graphene formation is adjusted from 30 s to 10 min. Finally, the substrate is cooled down to the room temperature during 10 s. Graphene samples synthesized on copper foils could be easily transferred onto an arbitrary substrate using PMMA (Poly- (methyl methacrylate)) as a supporting polymer substrate and ammonium persulfate as an etchant. The graphene layer deposited on copper is covered by polymer. Then the metal substrate is etched in the solution (1 g of ammonia persulfate in 5 ml of H_2O). Finally, the polymer with graphene is stacked to the desired substrate and PMMA is dissolved in acetone.

Context 4

Author: A. Kumar

Doi: 10.1016/j.carbon.2011.11.033

Method:

Graphene film synthesis was performed in a SEKI AX5200S MPCVD (microwave plasma chemical vapor deposition) system with H_2 (50 sccm) and CH_4 (5 sccm) as the feed gases. A 25 µm thick copper foil (Alfa Aesar, 99.8% purity) was used as the substrate. The foil was cut in 2 x 2 cm^2 pieces and placed into the MPCVD chamber, which was evacuated to a base pressure of 2 Torr and then filled with high-purity hydrogen at a pressure of 10 Torr. The Cu foil was supported by a ceramic stand that elevated it from the Mo puck by about 15 mm. The elevation of the Cu foil above Mo puck was found to be critical for the growth of the films because it ensures a strong coupling between the plasma and the Cu foil, thus enabling rapid self-heating of the foil by the microwave plasma. No additional heater was used. The thermocouple attached to the substrate table indicated a maximum reading of approximately 65°C during the process. A dual-wavelength infrared pyrometer indicated a maximum temperature of 700 ± 25 °C. However, as the copper foil is coated with graphene, the surface emissivity changes, making reliable IR measurements difficult. The entire growth is accomplished in less than 5 min and generally involves two stages. First, in a plasma cleaning and annealing step, the foil is kept in 400 W hydrogen plasma for 3 min. A H_2 flow rate of 50 sccm was used with downstream pressure control to maintain 10 Torr chamber pressure throughout the process. This process leads to rapid self-heating and removal of copper oxide from the foil surface. The second stage involves introduction of CH_4 (while maintaining the hydrogen plasma) at a flow of 5 sccm for a short duration (between 30 s and 2 min), providing approximately 10% CH_4 concentration in hydrogen. Depending on the growth duration and whether or not annealing is used, different forms of carbon can be deposited.

Context 5

Author: Sukang Bae

Doi: 10.1038/nnano.2010.132

Method:

An 8-inch-wide tubular quartz reactor is used in the CVD system, allowing a monolayer graphene film to be synthesized on a roll of copper foil with dimensions as large as 30 inches in the diagonal direction. A temperature gradient usually exists that depends on the radial position inside the tubular reactor. In our preliminary work, this sometimes resulted in inhomogeneous growth of the graphene on the copper foils. To solve this problem, a ~7.5-inch quartz tube wrapped with a copper foil was inserted and suspended inside the 8-inch quartz tube. In this way, the radial inhomogeneity in the reaction temperature could be minimized. In the first step of synthesis, the roll of copper foil is inserted into a tubular quartz tube and then heated to 1,000 °C with flowing 8 s.c.c.m. H_2 at 90 mtorr. After reaching 1,000 °C, the sample is annealed for 30 min without changing the flow rate or pressure. The copper foils are heat-treated to increase the grain size from a few micrometres to ~100 µm, as we have found that the copper foils with larger grain size yield higher-quality graphene films. The gas mixture of CH_4 and H_2 is then flowed at 460 mtorr with rates of 24 and 8 s.c.c.m. for 30 min, respectively. Finally, the sample is rapidly cooled to room temperature (~10 °C s^{-1}) with flowing H_2 under a pressure of 90 mtorr.

RAG-Qwen

The steps involved to synthesize graphene via arc discharge, based on the provided context, are as follows:

1. **Setup Preparation**: Use an electric arc oven equipped with two graphite electrodes (cathode and anode) and a steel chamber cooled by water.

2. **Electrode Configuration**: Place graphite rods as the electrodes, where the cathode has a diameter of 8 mm and the anode has a diameter of 6 mm, both with a purity of 99.99%.

3. **Current and Pressure Settings**: Maintain a constant current of 100 A during the discharge process. Initially, set the air pressure to 1000 Torr, which increases due to the heating effect during the discharge.

4. **Graphite Consumption and Product Collection**: The anode graphite rod will consume approximately 10 cm over a period of about 10 minutes. After the anode is consumed, the flue products deposited on the inner wall of the chamber are collected. Typically, several tens of grams of products are obtained per day.

5. **Heat Treatment**: Perform heat treatment in air to purify the collected products. Heat the sample from room temperature to 600°C over 2 hours and then cool it back to room temperature.

These steps outline the process of synthesizing graphene via arc discharge as described in the given context.

Ground truth

Graphene synthesis using arc discharge usises an electric arc to vaporise graphite,followed by the subsequest depostion of the graphene atoms and the formation of graphene flakes. You first need an arc reactor, with two water-cooled electrodes. The anode is usually filled with graphite which the cathode is a graphite rod. An electric arc is then established between the electrodes, using a high current (200A and under a specific pressure (e.g 500 Torr). This high temperature of the arc vaporises the graphite at the anode. The vaporised carbon atoms travel through the arc plasma and deposit on the chamber walls as graphene flakes.

Question 6

What are the steps involved to synthesize graphene via pyrolysis?

Context 1

Author: Emanuele Pompei

Doi: 10.1039/D4NR01986E

Method:

The porousified SiC wafers are cut into pieces (2×8 mm2) by a wafer saw. Pieces are individually transferred to an ultra-high vacuum (UHV) chamber where they are degassed overnight at 1000 K. After the degassing, samples are cooled to room temperature (RT) and heated again to 1650 K to allow the graphene formation on the surface of the pores. After 150 s of annealing, the sample is let to cool down to room temperature. It was observed that the temperature reached by the sample during the annealing depends on the quality of the mechanical contact between the sample and the sample holder. Therefore, by clamping one side of the sample more firmly than the other, we obtained the non-homogeneous graphenized sample discussed in the Functionalization with gold nanoparticles section.

Context 2

Author: Zhen-Yu Juang

Doi: 10.1016/j.carbon.2010.05.001

Method:

Single Layer Graphene and Few Layer Graphene were synthesized using Ni foil (30 μm in thickness, The Nilaco Corp.) as the substrate. After the Ni substrates were loaded into a quartz tubular furnace, the samples were heated to the process temperature of 900°C and maintained for 10 min under a H2/Ar (10 and 400 sccm, respectively) atmosphere. A CH4/H2/Ar gas mixture (10, 10 and 400 sccm, respectively) was then introduced into the quartz tube for 10 min, and the furnace was cooled to room temperature with fast (3 °C/s) and slow (0.3°C/s) cooling rates. The gas mixture during cooling is the same as heating stage. The pressure was maintained at 750 Torr during whole process using advanced pressure control system (APC, MKS Instruments)

Context 3

Author: Tianru Wu

Doi: 10.1002/adfm.201201577

Method:

The graphene synthesis was done by ambient pressure chemical vapour deposition (APCVD) at a typical temperature ranging from 950–1050°C for 30–80 min. A mixture of Ar and H2 was used as reactant gases. The H2 flow rate was adjustable between 0–10 sccm while keeping the total flow rate at 300 sccm. Carbon supply was controlled by heating the precursor with a halogen lamp under a typical polystyrene temperature (Tp) range of 80–280°C. After the growth process, the halogen tungsten lamp was turned off, and the furnace was cooled down to room temperature naturally.

Context 4

Author: Hong-Kyu Seo

Doi: 10.1038/srep16710

Method:

Synthesis of Graphene from coal tar pitch: The coal tar pitch (softening point = 60.8 °C, RIST) was diluted with quinoline solvent (JUNSEI Chemical) to a concentration of 8 wt%. Before spin-coating, SiO2 (500 nm)/Si substrates (2 cm × 2 cm) (NAMKANG HI-TECH) were treated with UV/ozone for 30 min to establish good wetting between coal tar pitch (CTP) solution and substrate and then a 20-nm thickness of CTP film was deposited on SiO2/Si substrates uniformly by spin coating (6000 rpm, 60 s). After baking at 240 °C for 30 min, the Ni layer (200 nm) was deposited (Magnetron Sputtering System, SNTEK; working pressure 7 mTorr; power 50 W; Ar flow rate 50 sccm) on a coal tar pitch film at room temperature. Then the sample was annealed using thermal chemical vapour deposition (CVD) (1100 °C, 4 min) with flowing 50 sccm Ar and 10 sccm H2 at a total pressure of ~0.3 Torr, then cooled to room temperature by removing the tube from the furnace while maintaining the same Ar/H2 flow. The Ni layer was etched away by dipping the sample into FeCl3 solution (Iron(III) chloride solution 45°Be’, JUNSEI Chemical) for 1 min and the sample was rinsed with DI water.

Context 5

Author: Aamir Ahmed

Doi: 10.1016/j.mseb.2024.117828

Method:

Synthesis of carbon: The bubble wraps were washed 5-times with DI water. After drying, these sheets were cut into tiny pieces and put into a petri dish. The dish was then heated at 100 °C to melt the pieces followed by solidifying. These solidified sheets were chopped into small pieces and immersed in a beaker with concentrated H2SO4. The beaker containing the sample was transferred to a muffle furnace and subjected to thermal treatment at 150 °C for a duration of 3 h. The excess sulphuric acid was drained out and the resultant was a black hard mass. The acquired solid was subjected to repeated washing with deionized water until a neutral pH was attained. This sulphonation step was followed by carbonization. The centrifuged sample was heated in a muffle furnace at 500 °C for 1 h. The sample then obtained was washed with DI water, dried at room temperature, and reduced to powder using mortal and pestle. Conversion of carbon into reduced graphene oxide (rGO): In this process, 1 g synthesized carbon and 1 g NaNO3 were added to 45 ml of H2SO4 (kept in an ice bath) and

stirred for 30 min until a homogenous black solution is produced. To this solution, 5 g $KMnO_4$ was slowly added and stirred at 50 °C for 2 h forming a green solution. 100 ml DI water and 6 ml H_2O_2 (35 %) were added drop-by-drop to stop the reaction. The precipitates were washed with 300 ml HCl (10 %) and 200 ml HCl (37 %) in a centrifuge. The final sample was heated at 250 °C in a muffle furnace, again washed with DI water, and reduced to rGO powder.

RAG-Qwen

The synthesis of graphene via pyrolysis typically involves several key steps, as illustrated in the methods described by various authors:

1. **Preparation of the Carbon Source**: The carbon source is prepared, often through the pyrolysis of a hydrocarbon precursor or a polymer. For example, in the method by Aamir Ahmed, the carbon source is derived from the pyrolysis of bubble wrap, which is first melted and then subjected to sulfuric acid treatment.

2. **Deposition of the Carbon Source on a Substrate**: The prepared carbon source is deposited on a suitable substrate. In the case of Hong-Kyu Seo's method, this involves spin-coating a coal tar pitch solution onto a SiO_2/Si substrate and then depositing a nickel layer before annealing.

3. **Annealing**: The substrate with the carbon source is subjected to high-temperature annealing to induce the formation of graphene. The temperature and duration can vary. For instance, in Tianru Wu's method, the annealing is performed at temperatures ranging from 950°C to 1050°C for 30 to 80 minutes, while in Emanuele Pompei's method, the sample is heated to 1650 K for 150 seconds.

4. **Removal of Metal Catalyst**: If a metal catalyst is used, it is removed after the graphene formation. In Hong-Kyu Seo's method, the nickel layer is etched away using $FeCl_3$ solution.

5. **Cooling and Post-Treatment**: After annealing, the sample is allowed to cool to room temperature. Depending on the specific method, additional treatments such as washing and further reduction might be applied to obtain reduced graphene oxide (rGO), as seen in Aamir Ahmed's method.

These steps outline the general process of synthesizing graphene via pyrolysis, although the exact details can vary based on the specific starting material, substrate, and conditions used.

Ground truth

Pyrolysis is done by first transforming organic materials like biomass into a carbonaceous material followed by further processing into graphene structures. First, biomass sources, such as rice husks or wheat straw are selected and pretreated by grinding, drying or in some cases, treated with chemicals like KOH to enhance carbonization. Pyrolysis is then carried out in a controlled, inert environment in a furnace or reactor. The temperature is gradually increased in the chamber in the reactor (typically between 600 to 1,200 degrees C), which causes the precursor material to decompose, thereby releasing volatile components and leaving behind a carbonaceous residue. The carbonaceous material is then post processed such as acid washing and annealing to improve the material's structural order to become graphene.

Question 7

What are the steps involved to synthesize graphene via liquid phase exfoliation?

Context 1

Author: Emanuele Lago

Doi: 10.1039/C6RA21962D

Method:

Graphene dispersion is produced by liquid phase exfoliation (LPE) of natural graphite. 500 mg of graphite flakes are dispersed in 50 mL of NMP and exfoliated in a sonic bath (VWR Ultrasonic Cleaner USC-THD) for 6 hours. Then, the dispersion is ultra-centrifuged at 10000 rpm (~17000 g), using sediment based separation (SBS) to remove un-exfoliated and thicker graphite flakes, for 30 min at 15 °C with an ultra-centrifuge (Beckman Coulter Optima™ XE-90, equipped with a SW32Ti rotor). Finally, the supernatant is collected by pipetting. A solvent exchange process is carried out for the re-dispersion of the exfoliated flakes in 1,3-dioxolane, a nontoxic and low boiling point (78 °C) solvent, using a Heidolph Hei-Vap rotary evaporator. After the evaporation process of N-methyl-2-pyrrolidone (NMP), the graphitic material is collected and washed three times with acetone to remove the NMP residual using a compact centrifuge (Sigma-Aldrich). The washing step is repeated and the flakes are eventually dispersed in 50 ml of 1,3-dioxolane, adjusting its concentration at 10 mg mL-1.

Context 2

Author: Kewen Pan

Doi: 10.1038/s41467-018-07632-w

Method:

Liquid exfoliation of graphene with high concentration: Expandable graphite with +50 mesh flake size was purchased from Sigma-Aldrich. Cyrene (dihydrolevoglucosenone, >99%) was provided by Circa Group Pty Ltd. N-methyl-2-pyrrolidinone (NMP, >99%) was from Alfa Asia. CAB (butyryl content 35–39%) was from Arcos Organics. Graphene nanoflakes were obtained via the liquid phase exfoliation method. Expandable graphite were placed in a ceramic crucible and then heated in an 800 W commercial microwave oven for 30 s to obtain expanded graphite with fewer layers. The expanded graphite flakes were stirred and washed in deionized water to remove residual acid until the pH is close to 7. Then, the mixture was dried in an oven for 5 h at 100 °C. Dried expanded graphite was deposited into NMP solvent (10 mg mL−1) and Cyrene (10 mg mL−1), respectively where these organic solvents provide appropriate surface energy for sonication processes. After that, the mixture was sonicated in an ultrasonic bath (SHESTO, UT8061-EUK). Samples were extracted at 0, 2, 4, 6, 8, 10, 12, 14, 16, 20, 24, 32, 40, 48, and 56 sonication hours for sheet resistance measurement.

Context 3

Author: Ethan B. Secor

Doi: 10.1002/adma.201502866

Method:

Graphene was exfoliated from graphite using a high shear mixer (Silverson L5M-A) with a square hole high shear screen. Ethyl cellulose (EC) (Sigma–Aldrich, 4 cP grade as measured at 5% in 80:20 toluene: ethanol, 48% ethoxy) was dissolved in ethanol (Koptec, 200 proof) at a concentration of 0.2–2% w/v, and flake graphite (Asbury Graphite Mills, Grade 3061) was added at 10% w/v. This mixture was shear mixed for 2 h at 10,230 rpm in an ice bath, and then centrifuged at 4000 rpm (≈3000 g) for 2 h to sediment out large graphite flakes (Beckman Coulter Avanti J-26 XPI centrifuge). The supernatant containing graphene, EC, and ethanol was harvested by pipette. For the flocculation step, this supernatant was mixed in a 16:9 wt. ratio with an aqueous NaCl solution (0.04 g/mL NaCl, Sigma–Aldrich, >99.5%) and centrifuged for 6 min at 7500 rpm (≈10 000 g) to sediment the graphene/ EC composite. This sediment was washed with deionized water, collected by vacuum filtration (Millipore Nitrocellulose HAWP 0.45 μm filter paper), and then dried to yield the graphene/EC powder, with a graphene content of 25–65 wt% depending on the starting EC loading.

Context 4

Author: Qingbin Zheng

Doi: 10.1021/nn2018683

Method:

Five grams of natural graphite flakes (Asbury Graphite Mills) and 150 mL of sulfuric acid (H2SO4, General Chemical) were mixed and stirred in a round-bottom flask at a speed of 200 rpm. Fifty milliliters of fuming nitric acid was added into the mixture. The mixture was kept at room temperature and stirred for 24 h. Two-hundred milliliters of deionized water was then poured slowly into the mixture. The resultant mixture was washed using DI water, followed by centrifugation and drying at 60 °C for 24 h to obtain graphite intercalation compound. The dry graphite intercalation compound powder was thermally expanded at 1050 °C for 15 s to obtain expanded graphite that was used for the production of graphene oxide (GO). One gram of expanded graphite and 200 mL of sulfuric acid were mixed and stirred in a three-neck flask. Ten grams of KMnO4 was added to the mixture while stirring. The solution was transferred into an ice bath, and 200 mL of deionized water and 50 mL of H2O2 were poured slowly into the mixture, realizing color change of the suspension to light brown. The GO particles were washed and centrifuged with a HCl solution three times, then centrifuged again and washed with deionized water until the pH of the solution became about 5 to 6. The GO particles obtained thereby were diluted using DI water (~1 mg/mL) and dispersed by gentle shaking.

Context 5

Author: Nai Gui Shang

Doi: 10.1039/C2CC17185F

Method:

Graphite powders of 50 mg (99.0% purity, from Sigma-Aldrich) with a grain size less than 20 μm were mixed and ground with 0.1—0.5 mL ionic liquid (IL, 1-butyl-3-methylimidazolium hexafluorophosphate, BMIMPF6, from Sigma-Aldrich) for 0.5–4 hrs. Then the mixtures were added into a solution of 15 mL N,N-dimethylformamide (DMF) and 15 mL acetone and centrifuged at a speed of 3000 rpm for 30 min in order to remove the ionic liquid. This washing cycle was repeated three times, and the final sediment was dispersed in 1 L DMF. After one-day sedimentation, the large/thick graphitic flakes completely precipitated on the bottom of the bottle. The supernatant was dried, and the yield was calculated as 20 wt%, which is much larger than that of other methods reported. The supernatant was collected and centrifuged to get a dense suspension, with which the graphene samples were prepared for the study of their microstructure and electrical properties. The suspension was diluted and dropped on lacy carbon-coated Cu grid for TEM observations, on clean Si wafers or mica sheets for the XPS, Raman, and AFM studies, and on thermally oxidised Si wafer substrates (300 nm SiO2) for the study of electrical properties. To obtain uniform large-area graphene thin films, the Si/SiO2 substrate was functionalised with 5% 3-triethoxysilylpropylamine (APTES) aqueous solution for 30 min. All samples were completely dried under an infrared light or on a hot plate before measurements. We should note the role of ILs in this study. ILs are low-temperature molten salts, and as their name denotes, are liquids composed entirely of ions. They have been proposed as a new class of “green” organic solvents because not only are they able to solvate a large variety of organic and inorganic compounds (polar or non-polar), but they also possess high thermal and chemical stability, high ionic conductivity, wide electrochemical window, and negligible vapour pressure. The mixing of a small quantity of IL and graphite flakes forms a gel-like composite. When graphite flakes are ground with ionic liquids, the shear force detaches the graphene layers from the graphite flakes. The ionic liquids can effectively surround each layer, preventing the detached graphenes from restacking. Based on simulation and experimental studies on the processing of carbon nanotubes with imidazolium-based ILs, the electronic structure of graphene layers in the bucky gel remains unchanged, and there is no charge transfer between graphene and imidazolium cations

RAG-Qwen

To synthesize graphene via liquid phase exfoliation, several steps are typically involved:

1. **Graphite Preparation**: Start with graphite flakes, which can be natural graphite or expandable graphite that has been processed to reduce its thickness.

2. **Dispersion in Solvent**: Graphite flakes are dispersed in a suitable solvent. Common solvents include N-methyl-2-pyrrolidone (NMP), dimethylformamide (DMF), and 1,3-dioxolane. The graphite is often mixed with a small amount of a surfactant or polymer to enhance exfoliation and prevent re-aggregation.

3. **Exfoliation**: The dispersion is subjected to intense mechanical energy through techniques such as ultrasonication or high-shear mixing. This process breaks down the

graphite flakes into individual graphene sheets. The duration and intensity of the exfoliation depend on the specific method and equipment used.

4. **Centrifugation**: After exfoliation, the dispersion is centrifuged to separate unexfoliated graphite flakes and aggregates from the graphene sheets. This step is crucial for obtaining a high-quality graphene dispersion.

5. **Solvent Exchange**: The supernatant containing the graphene sheets is collected and exchanged to a different solvent if needed. This step might involve removing the initial solvent and replacing it with another solvent that is more compatible with subsequent processing or applications.

6. **Washing and Dispersal**: The graphene sheets are often washed with solvents like acetone or water to remove any residual chemicals from the exfoliation process. The graphene is then dispersed in the final solvent at an appropriate concentration for further use or processing.

The exact details of these steps can vary depending on the specific research or application requirements. Different researchers may use variations in solvent types, exfoliation methods, and post-processing techniques to optimize the quality and properties of the graphene produced.

Ground truth

Graphite is first placed into some kind of liquid suspension medium, often ethanol, alcohol or some other kind of solvent, and then sonicated either through bath sonication or tip sonication. In bath sonication, the graphite-liquid suspension is usually placed into some kind of container before being placed in the bath sonicator. In tip sonication, the tip is inserted directly into the liquid. The suspension is then sonicated for a period of time. During the sonication, cavitation within th eliquids will cause the different graphene layers to exfoliate from one another. After a certain period of time, the suspensions are removed from the sonicator and then centrifuged. The supernatent of the liquid is then taken and characterised to confirm the presence of mono-layer or few-layer graphene. Surfactants can be utilised to maintain or improve the suspension but might cause issues with final device properties.

Question 8

What are the steps involved to synthesize graphene via laser ablation?

Context 1

Author: Rafal Hameed

Doi: 10.1016/j.matpr.2019.09.185

Method:

Graphene sheet has synthesis through pulsed laser ablation in liquid process of a pure (99.9%) graphite pellet. This pellet cut from a rod of graphite (5 mm diameter). The graphene sheet synthesis in two process steps, at the beginning carbon nanotubes CNTs synthesis, that accomplished using pulsed laser ablation of graphite pellet that put in the bottom of quartz vessel and immersed in (2 ml) water. The volume and the height (5 mm) of water were constant to make sure that a constant power reached to the target, the second step involved re-irradiation of the obtained colloidal that ready before and contained carbon nanotubes, after removing the target with the same laser energy. Pulsed Nd: YAG laser used of 1064 nm wavelength with output pulse duration is 7 ns and (80– 160 mJ) laser energies, for fixed pulses (100 pulses).

Context 2

Author: Min Yi

Doi: 10.1021/nn101796e

Method:

Sample Preparation (Laser Synthesis of Epitaxial Graphene on SiC): N-type (nitrogen, 3 × 1018 cm−3) 4H-SiC(0001) wafer, on-axis, with chemical mechanical polishing (CMP, Ra ≤ 1 nm) on the Si face was purchased from SiCrystal AG, which was used for RHEED, X-ray diffraction, and TEM characterizations. High-purity semi-insulating (ρ ≥ 1 × 105 Ω·cm) 4H-SiC(0001) wafer, on-axis, was also purchased from Cree: the wafer was epiready polished by NovaSiC up to an atomically flat surface (Rq ≤ 1 Å), which was used for STM and Raman measurements. The substrates (5 mm × 5 mm) were cut from the wafers and treated by sequential ultrasonic bath in acetone, methanol, and isopropyl alcohol (for 5 min to remove grease), Piranha cleaning (mixture of H2SO4 + H2O2 (1:1) for 3 min to wipe off metal powder and organic contaminants) and HF etching (10% HF solution for 3 min to remove the surface oxide). The sample was placed in a high-vacuum (HV) chamber (base pressure ≈ 10−7 Torr, pumpdown time ≈ 2 h) designed for pulsed laser deposition (PLD). The SiC surface was irradiated with a pulsed KrF excimer laser (Lambda Physik LPX 210i, λ = 248 nm, pulse length ≈ 25 ns) with a nonhomogenized beam. The beam intensity profile was measured with 1 mm interval at the removable beam blocker with hole (20 mm × 10 mm) near the laser exit port, and the regions with higher fluence than average were blocked by shadow mask (stainless

steel). The SiC substrate with specially designed holder was loaded, inside the chamber, perpendicular in the direction of the laser beam. The experiments were performed at ∼1.1, ∼1.2, and ∼1.4 J/cm2 with 500 shots (20 Hz, 25 s) to synthesize monolayer, bilayer, trilayer graphene, respectively. The vacuum condition of ∼10−6 Torr was used for all experiments.

Context 3

Author: Dapeng Wei

Doi: 10.1016/j.carbon.2012.11.026

Method:

Two 1 x 2 cm quartz wafers were used as substrate to grow graphene. The quartz wafers were first cleaned by ultrasonication in methanol, acetone, and DI water, and were dried by high purity N2 gas. Photoresist S-1805 (from Shipley Comp.) was diluted with a volume ratio of 1:6 in Thinner-P (from MicroChem Corp.), and was then spin-coated on the quartz wafer at 10,000 rpm. The thickness of the coated film is about 30 nm. The photoresist-coated quartz wafers were then baked for 5 min at 120°C . One coated quartz wafer was covered by another piece of quartz wafer, and then mounted on a sample stage in a vacuum chamber. Before growing graphene, the chamber was pumped and purged by high-purity N2 gas, and maintained at a pressure below 0.1 Torr. A continuous wave (CW) Nd:YAG laser with a wavelength of 532 nm was focused on the S-1805 film through the transparent quartz substrate using a lens of 150 mm focal length. The carbon atoms were decomposed from the laser heated photoresist, then dissolved in the molten quartz, and extracted to form graphene when the temperature of quartz was decreased. With our optical setup, the graphene was produced with a laser power of 2.8 W, irradiated for 3–5 min.

Context 4

Author: Emanuele Pompei

Doi: 10.1039/D4NR01986E

Method:

The porousified SiC wafers are cut into pieces (2×8 mm2) by a wafer saw. Pieces are individually transferred to an ultra-high vacuum (UHV) chamber where they are degassed overnight at 1000 K. After the degassing, samples are cooled to room temperature (RT) and heated again to 1650 K to allow the graphene formation on the surface of the pores. After 150 s of annealing, the sample is let to cool down to room temperature. It was observed that the temperature reached by the sample during the annealing depends on the quality of the mechanical contact between the sample and the sample holder. Therefore, by clamping one side of the sample more firmly than the other, we obtained the non-homogeneous graphenized sample discussed in the Functionalization with gold nanoparticles section.

Context 5

Author: Fatemeh Kazemizadeh

Doi: 10.1016/j.physb.2017.11.052

Method:

Powdering graphite combined with nickel particles at a concentration of 2 at% was obtained using ball milling process. The powder was pressed under 1300 kg cm-2 for 15 min, and a graphite pellet was formed. The targetwas embedded in a quartz tube, 5 cm in diameter and 80 cm in length and argon gas was flowed through it at the rate of 2.4 l/min. The tube was put in a furnace, 1050°C and pressurized at 300 mbar. Fundamental mode of a Q-switched pulsed Nd-YAG laser (1064 nm in wavelength), with 10ns pulse duration, and repetition rate of 10 Hz, was focused on the carbon pellet so that the illuminated area reduced to 12 mm2. Carbon atoms generated by ablation were gathered on a collector on a water-cooled metal surface in the end of the tube. The synthesized product was then scraped off the collector and analyzed. The experiment was carried out with two different laser fluences of 3.5 and 5.5 J/cm2. The last laser energy was irradiated on a graphite pellet without metal particles for further investigation.

RAG-Qwen

Synthesizing graphene via laser ablation involves several key steps:

1. **Preparation of the Target Material**:

   - For some methods, a graphite pellet is prepared. This can be done through processes like ball milling combined with metal particles or by cutting graphite rods into specific sizes. The graphite pellet is then placed in a quartz vessel or tube.

2. **Setting Up the Experimental Environment**:

   - The experimental setup typically includes a vacuum chamber to ensure low-pressure conditions, which are crucial for laser ablation processes. The graphite target is positioned within this chamber, often with a precise alignment to the laser beam.

3. **Laser Irradiation**:

   - A pulsed laser is used to irradiate the graphite target. The laser parameters vary depending on the method, including the wavelength (e.g., 1064 nm for Nd:YAG lasers), pulse duration (e.g., 7 ns to 10 ns), and energy per pulse (e.g., 80-160 mJ). The number of pulses can also vary, such as 100 pulses in some cases.

4. **Collection and Analysis**:

   - After irradiation, the graphene sheets are collected from the target or a collector placed downstream. These sheets can then be analyzed using various techniques to confirm their structure and properties.

Here are examples from the provided contexts:

- **Rafal Hameed**:

  - Uses a pulsed Nd:YAG laser to ablate a graphite pellet immersed in water, resulting in the formation of carbon nanotubes followed by graphene sheets through re-irradiation of the colloidal solution.

- **Min Yi**:

- Utilizes a pulsed KrF excimer laser to irradiate a SiC substrate, leading to the epitaxial growth of graphene layers.

- **Dapeng Wei**:

  - Employes a CW Nd:YAG laser to decompose carbon from a photoresist layer on a quartz substrate, forming graphene through a process involving laser heating and cooling.

- **Emanuele Pompei**:

  - Involves heating porous SiC wafers to high temperatures under UHV conditions, allowing graphene to form within the pores through laser-induced decomposition of carbon.

- **Fatemeh Kazemizadeh**:

  - Uses a Q-switched pulsed Nd-YAG laser to ablate a graphite pellet containing nickel particles, collecting the resulting graphene on a water-cooled surface.

Each method highlights variations in the specific details of laser type, target material, and processing conditions, but the core principle of using laser ablation to generate graphene remains consistent across these examples.

Ground truth

Porous graphene can be made using the laser ablation of a nickel-graphite target under the flow of Argon gas. Although not technically a traditional graphene layer, porous graphene is a similar carbon allotrope with an inhomogenous structure and curvature on the graphene layers. First graphite and nickel particles are ball milled and then pressed into a graphite-nickel pellet under pressure to form a target. The target is then placed into a quartz tube where argon is allowed to flow at about 2.4l/min. The tube is then heated to 1050 degrees celsius and presurised at 300mbar. A Nd-YAG laser (1064nm in wavelength), with a 10 ns pulse duration and repetition rate of 10Hz is then focused on the carbon pellet. The illuminater area is about 12 mm2. The carbon atoms generated by this ablation are carried by the argon gas and then gathered on a collector on a water-cooled surface at the end of the tube and scraped off as porous graphene.

Question 9

What are the steps involved to produce graphene oxide?

Context 1

Author: Maria Refalo Magro

Doi: 10.1016/j.cartre.2025.100509

Method:

The process of separating and oxidizing graphite into graphite oxide and eventually graphene oxide involves the following steps. Initially, 1 g of graphite flakes was combined with 150 mL of concentrated H_2SO_4 and mechanically stirred at 400 rpm for approximately 10 minutes at room temperature. 4 g of $KMnO_4$ were then added incrementally, in 1 g portions, every hour. This incremental addition was done to manage the generation of Mn_2O_7 and mitigate the risk of explosive decomposition.Subsequently, the mixture was left stirring at 900 rpm for 24 h at room temperature. A total of 150 mL of deionized (DI) water was then added in small volumes making sure the temperature of the mixture did not exceed 90 °C with each addition.After cooling down, a few drops of 30 % hydrogen peroxide (H_2O_2) were added until no further effervescence occurred. The peroxide addition serves to reduce and eliminate residual permanganate. The resulting mixture was then subjected to centrifugation (Hermle Z366) at 8820 rpm (rcf = 10,000), to isolate the graphite oxide from the supernatant which was discarded. The graphite oxide was then redispersed in deionized water and the procedure (centrifugation followed by re-dispersion) was repeated several times until the supernatant's final pH reached 6 – 6.5. The final dispersion stage (from graphite oxide to graphene oxide) involved sonication for 1 h at 100 % power and a frequency of 80 kHz at room temperature using an Elmasonic P30H sonicator bath. This critical step allowed for the separation of the layers comprising graphite oxide, yielding free graphene oxide sheets. The sonicated suspension was then dried in an oven for 48 h at 40 - 50 °C resulting in the production of a thin dark-brown graphene oxide ‘paper’.

Context 2

Author: Dheeraj

Doi: 10.1016/j.poly.2024.117175

Method:

Graphene oxide was produced employing a modified Hummer's method. Initially, a mixture of H_2SO_4 and H_3PO_4 in a 9:1 vol ratio was prepared and placed in a 500 mL round bottom flask, where it was vigorously stirred. Subsequently, 0.55 g of graphite powder was introduced into the acid mixture while maintaining continuous stirring. After achieving complete homogenization, 2.74 g $KMnO_4$ was gradually poured into the solution with vigorous stirring.

The solution exhibited a transition to a dark green color during this process, and a small quantity of H_2O_2 was added dropwise, causing an exothermic reaction. To control the reaction's temperature, the entire process was conducted within an ice bath. Following this, a solution composed of 10 mL of hydrochloric acid and 50 mL of deionized water was introduced into the mixture over a span of 10 min. The resulting residue was carefully collected and subjected to multiple wash cycles with HCl and DI water. Lastly, the precipitated material was desiccated in an oven at 70 °C for a duration of 12 h.

Context 3

Author: Qingbin Zheng

Doi:10.1021/nn2018683

Method:

Five grams of natural graphite flakes (Asbury Graphite Mills) and 150 mL of sulfuric acid (H_2SO_4, General Chemical) were mixed and stirred in a round-bottom flask at a speed of 200 rpm. Fifty milliliters of fuming nitric acid was added into the mixture. The mixture was kept at room temperature and stirred for 24 h. Two-hundred milliliters of deionized water was then poured slowly into the mixture. The resultant mixture was washed using DI water, followed by centrifugation and drying at 60 °C for 24 h to obtain graphite intercalation compound. The dry graphite intercalation compound powder was thermally expanded at 1050 °C for 15 s to obtain expanded graphite that was used for the production of graphene oxide (GO). One gram of expanded graphite and 200 mL of sulfuric acid were mixed and stirred in a three-neck flask. Ten grams of $KMnO_4$ was added to the mixture while stirring. The solution was transferred into an ice bath, and 200 mL of deionized water and 50 mL of H_2O_2 were poured slowly into the mixture, realizing color change of the suspension to light brown. The GO particles were washed and centrifuged with a HCl solution three times, then centrifuged again and washed with deionized water until the pH of the solution became about 5 to 6. The GO particles obtained thereby were diluted using DI water (~1 mg/mL) and dispersed by gentle shaking.

Context 4

Author: Abhijit Ganguly

Doi: 10.1021/jp203741y

Method:

Highly oxidised Graphene Oxide (GO) was produced using a modified Hummers' process. The starting material Graphite powder with particle size ≤20 μm was purchased from Fluka and is denoted here as "pristine" graphite. All other chemical and reagents were purchased from Aldrich. A mixture of 2.5 g of Graphite and 1.9 g of $NaNO_3$ was placed in a flask cooled in an ice bath. 85 mL of H_2SO_4 was added to the mixture and stirred until homogenized. Solution of 11.25 g of $KMnO_4$ in distilled water was gradually added to the solution while stirring. After 2 hours, the solution was removed from the ice bath, and further stirred for 5

days. Finally, brown-coloured viscous slurry was obtained. The slurry was added to 500 mL aqueous solution of 5 wt% H_2SO_4 over 1 hour while being continuously stirred. The mixture was stirred for a further 2 hours. Subsequently, 10 ml of H_2O_2 (30 wt% aqueous solution) was then added to the mixture and stirred for further 2 hours. This mixture was then left to settle overnight. The mixture was filtered and further purified by dispersing in 500 mL aqueous solution of 3 wt% H_2SO_4 and 0.5 wt% H_2O_2. After two days of precipitation, the supernatant solution was removed. This process was repeated five times. The solid product obtained after the rigorous cleaning process was rinsed using copious amounts of distilled water and dried in oven, as reported in literature. The resulting solid was dispersed in water by ultrasonication for 2 h to produce a GO aqueous dispersion. After one-day sedimentation, the thick flakes were removed and the supernatant was collected for further measurements.

Context 5

Author: Jinping Zhao

Doi: 10.1021/nn1015506

Method:

The detailed experimental procedure for the preparation of large-area graphene oxide (GO) sheets is described as follows. A 2 g portion of natural flake graphite with an average size of 500−600 μm, 2 g of $NaNO_3$, and 96 mL of concentrated H_2SO_4 were mixed at 0 °C. During the following stages the mixture was continuously stirred using a magnet stirrer. 12 g of $KMnO_4$ was gradually added to the above mixture while keeping the temperature at 0 °C. The mixture obtained was first stirred at 0 °C for 90 min and then at 35 °C for 2 h. Distilled water (80 mL) was slowly dropped into the resulting solution, over a period of around 30 min, to dilute the mixture. Then 200 mL of distilled water was added followed by 10 mL of H_2O_2 (30%), and the stirring continued for 10 min to obtain a graphite oxide suspension. During this final step, H_2O_2 (30%) reduced the residual permanganate and manganese dioxide to colorless soluble manganese sulfate. The graphite oxide deposit was collected from the graphite oxide suspension by high-speed centrifugation at 16000 rpm for 10 min, and repeatedly washed with distilled water until the pH = 7. Then a mild sonication (80 W, 5 min) was used to exfoliate the graphite oxide to obtain a GO suspension. To obtain uniform large-area GOs, a low-speed centrifugation at 3000 rpm was first used to remove thick multilayer flakes until all the visible particles were removed (3−5 min). Then the supernatant was further centrifuged at 5000 rpm for 5 min to separate large flakes (precipitate) and small flakes (supernatant). Finally, the obtained precipitates containing large flakes were redispersed in water to get a large-area GO sheet suspension. To determine the yield of this process, we measured the weight of the above precipitate containing large flakes after drying in a vacuum oven at 90 °C for 24 h to completely remove the water. The weight of large flakes obtained from the original 2 g of sample was about 0.25 g.

RAG-Qwen

The steps involved in producing graphene oxide typically follow a modified Hummers' method, which includes the following general steps:

1. **Graphite Treatment**: Graphite flakes are mixed with concentrated sulfuric acid (H2SO4) and optionally phosphoric acid (H3PO4) or fuming nitric acid (HNO3). This initial treatment intercalates the graphite layers.

2. **Oxidant Addition**: Potassium permanganate (KMnO4) is added incrementally to the mixture to oxidize the graphite. This process is often carried out under controlled conditions such as stirring at specific speeds and temperatures to manage the oxidation process and prevent explosive decomposition.

3. **Neutralization and Washing**: The mixture is then neutralized and washed with deionized water to remove excess acids and reduce the permanganate ions. This step may involve adding hydrogen peroxide (H2O2) to further reduce any residual permanganate and mangenese dioxide to soluble manganese sulfate.

4. **Centrifugation and Dispersal**: The resulting graphite oxide is isolated through centrifugation and then redispersed in deionized water. This step may be repeated to ensure purity and to adjust the pH to a range suitable for further processing.

5. **Exfoliation**: Sonochemical methods are often employed to exfoliate the graphite oxide into individual graphene oxide sheets. This is typically done by sonication in a sonicator bath.

6. **Drying**: The graphene oxide is then dried to remove any remaining liquid, often at a temperature below 100°C to preserve the structure.

These steps can vary slightly depending on the specific protocol used, but they generally follow this sequence to produce graphene oxide from graphite.

Ground truth

Graphene oxide (GO) can be synthesized by the oxidation of graphite into graphite oxide followed by the exfoliation of graphite oxide into GO. The leading method right now is the modified Hummer's method. A common procedure is as follopws: First, mix graphite and potassium permanganate in a 1: 6 ratio by weight. Then, in another beaker, mix 96% sulphuric acid and then add 75% phosphotic acid. The acids should be in a 6:1 ratio. Ensure the acids are added to the graphite and potassium permanganate slowly and bit by bit while constantly stirring. This allows for the intercalation of the graphite and oxidation of the graphite into graphite oxide. Stir the entire solution at 50 degrees centigrade for 12 hours and then allow to cool to room temperature. Next, pour the entire acid mixture into another beaker with ice made from de-ionised water. Add about 3mL of 30% hydrogen peroxide to the mixture and stire to allow the GO to exfoliate.

Question 10

What are the steps involved to produce reduced graphene oxide?

Context 1

Author: Benjamin Diby Ossonon

Doi:10.1039/c6ra28311j

Method:

Graphene oxide was synthesized from natural graphite through the Hummers method which has been improved. The graphite is first pre-oxidized by mixing 5g graphite powder with concentrated sulfuric acid (12.5 mL), potassium persulfate (2.5 g) and phosphorus pentoxide (2.5g). The mixture was heated at 80°C for 6 hours. After dilution with 500 mL of H_2O, the mixture was stirred at room temperature overnight. After that, the product is recovered by centrifugation and washed thoroughly with Nanopure water until the filtrate has a pH close to 7. The product obtained is then dried at room temperature for one day. Then, the pre-oxidized graphite is dispersed in H_2SO_4 (0°C, 115 mL). The temperature of the mixture is carefully controlled to not exceed 10°C. Subsequently, potassium permanganate (15 g) is gradually added with constant stirring for 1 hour. The dispersion is then incubated at 35°C for 2 h and this is followed by the addition of Nanopure water (225 mL) in small portions (15 mL) to control the temperature of the mixture, which must remain below 50°C. To completely dissolve the $KMnO_4$, hydrogen peroxide (30%, 12.5 mL) was immediately added at the end of a second dilution (H_2O, 700mL), and the mixture is stirred for 48 hours. Finally, the suspension is filtered, washed first with HCl (10%) to remove residual metal ions, and repeatedly with Nanopure water until the pH of filtrate becomes neutral. The filtrate is quickly tested by adding a few drops of 1 M NaOH to verify the presence of metal ions in graphene oxide (GO). The product obtained (graphite oxide) is then dried in air. The resultant graphite oxide was dispersed in Nanopure water kept in the ultrasonic bath for 24 hours to maximize exfoliation. The reduced graphene oxide (RGO) is obtained by thermal reduction of GO in Ar/5% H_2 at 800°C for 2 h. The resulting RGO can be dispersed in water and the dispersion stayed stable for few hours.

Context 2

Author: Tran Van Khai

Doi: 10.1039/C2JM33194B

Method:

Graphene oxide (GO) was synthesized from graphite powder via a modification of Hummers and Offeman's method. In a typical reaction, 5 g of graphite, 60 mL of H_3PO_4, and 180 mL of H_2SO_4 were stirred together with a Teflon-coated magnetic stirring bar in an ice bath. Next, 60 g of $KMnO_4$ was slowly added while the temperature was maintained at 0 °C. Once mixed,

the solution is transferred to a 35 ± 5 °C water bath and stirred for 3 h, forming a thick paste. Next, distilled water (450 mL) was slowly dropped into the resulting paste to dilute the mixture, and then the solution was stirred for 1 h while the temperature was raised to 90 ± 5 °C. Finally, 800 mL of distilled water was added, followed by the slow addition of 60 mL H2O2 (30%), turning the color of the solution from dark brown to yellow. During this final step, H2O2 (30%) reduced the residual permanganate and manganese dioxide to colorless soluble manganese sulfate. The GO deposit was collected from the GO suspension by high-speed centrifugation at 15 000 rpm for 30 min. The obtained GO was then washed with 1000 mL of HCl (5%) and repeatedly washed with distilled water until pH = 7. To obtain uniform GO, a low-speed centrifugation at 3000 rpm was first used to remove thick multilayer sheets until all the visible particles were removed (3–5 min). Then the supernatant was further centrifuged at 10 000 rpm for 30 min to remove small GO pieces and water-soluble byproducts. The final sediment was redispersed in 500 mL of DMF with mild sonication, resulting in a solution of exfoliated GO. In general, for chemically reduced GO (RGO), 500 mL of exfoliated GO was stirred for 30 min and 10 mL of hydrazine monohydrate was added. The mixtures were heated at 150 ± 5 °C in an oil bath for 24 h; a black solid precipitated from the reaction mixtures. Products were collected by centrifugation at 12 000 rpm for 45 min and washed with DI water and methanol until pH = 7. Next, the obtained RGO was dried and stored in a vacuum oven at 60 °C until use.

Context 3

Author: Nahid Rehman

Doi: 10.1016/j.sbsr.2025.100789

Method:

The process of producing graphene oxide (GO), the precursor of reduced GO (rGO), is derived from a modified Hummer's approach, which is non-explosive and non-toxic. Using ice baths with magnetic stirring allows control over the highly exothermic processes that produce GO. The modified Hummers approach synthesized the reduced graphene oxide. In a 500 ml beaker, add 27 ml of H2SO4:H3PO4 (9:1) and stir for a minute. To this solution, add 225 mg of graphite powder under continuous stirring. Slowly add 1.32 g of KMnO4; this step follows the exothermic reaction; therefore, necessary precautions should be taken. Leave the solution stirring for 6 h unless the dark green color of the solution is observed. Allow the contents of the beaker to cool down at room temperature. Add dropwise 0.675 mL of 30 % H2O2 on the ice bed and stir it for 10 min at room temperature to remove excess KMnO4. When the mixture is cooled down (exothermic nature of reaction), add 10 mL of 30 % HCl and 30 mL of distilled water (DW) followed by centrifugation at 5000 rpm for 7 min. Decant the supernatant obtained and wash thrice with the HCl and DW mix. Oven-dry the leftovers for 24 h at 50 °C to procure the graphene oxide powder. The color change from brown to black is indicative of GO reduction. This work employed the thermal reduction approach to acquire the reduced form of graphene oxide. The process of chemically reducing GO to rGO requires the use of hazardous and non-environmentally friendly chemical reagents. After the produced GO was heated to 60 °C for three days in a hot air oven, the mass yield of the material decreased as a result of the removal of oxygenated functional groups and any remaining water molecules between the graphene sheets, which allowed rGO to develop through thermal reduction

Context 4

Author: Miftahul Husnah

Doi: 10.1088/2053-1591/aa707f

Method:

Synthesis of graphite oxide: The reduced graphene oxide (rGO) synthesis process was started by preparing 9:1 mixture of H_2SO_4/H_3PO_4 (23 ml) and adding 1 g graphite flakes. Afterwards, 3 g $KMnO_4$was slowly added into the solution and maintained below 10 °C during the addition process. The mixture was stirred for 40 min at 50 °C and then diluted with deionized water. 30% hydrogen peroxide (H_2O_2) was added to stop the oxidation process. The addition of H_2O_2 into the solution changed its color into yellowish green with bubbles that indicated a high degree of oxidation process. The solution was filtered and washed until the PH close to 7. Then, the product was heated at 60 °C for 12 h. Synthesis of graphene oxide (GO) and rGO: Colloids of GO were prepared by dispersing into 133 ml ethylene glycol (EG). While stirring, the rGO was prepared by dripped hydrate hexahydrate (N_2H_4). The reduction process was completed by introducing the sample into a conventional microwave for 20 min. Afterwards, the sample was filtered and washed using deionized water and alcohol, and dried for 12 h.

Context 5

Author: Ruichao Zhao

Doi: 10.1016/j.matchemphys.2025.130850

Method:

Typically, flake graphite (10 g), $KMnO_4$ (6 g) and K_2FeO_4 (4 g) as the oxidants, and boric acid (0.01 g) as a stabilizer were first dispersed in 100 mL of concentrated sulfuric acid in a vessel and stirred for 1.5 h at less than 5 °C. After the addition of another $KMnO_4$ (5 g), the vessel was transferred into a water bath at about 35 °C and stirred for another 3 h to complete the deep oxidation. Next, as 250 mL of deionized water was slowly added, the temperature was adjusted to 95 °C and held for 15 minutes, when the diluted suspension turned brown, indicating the hydrolysis and absolute exfoliation of intercalated graphite oxide. Finally, this brown suspension was further treated with 12 mL H_2O_2 (30%) to reduce the residual oxidants and intermediates to soluble sulfate, then centrifuged at 10000 rpm for 20 min to remove the residual graphite, and washed with 1 mol/L HCl and deionized water repeatedly, producing the terminal GO. Thereafter, reduced graphene oxide (rGO1, rGO2) was prepared via a facile hydrothermal procedure as follows. GO colloidal (2 mg·mL-1, 100 mL) was ultrasonicated for 30 min to reach complete dispersion. Subsequently, $C_6H_8O_6$ was added into the above colloidal and subject to another 30 min ultrasonication to form a uniform dispersion. The as-formed dispersion was then transferred into an autoclave for hydrothermal treatment at 180 ℃ for 12 h. Finally, the solid product was cleansed and dried under 60 ℃ to obtain reduced graphene oxide. Quantitatively, usage of 0.8000 g, 1.2000 g of $C_6H_8O_6$ yields reduced graphene oxide sample rGO1, rGO2, respectively.

RAG-Qwen

To produce reduced graphene oxide (rGO), several methods can be employed, each involving different steps. Here are the general steps based on the provided contexts:

1. **Graphene Oxide (GO) Synthesis**:

- **Hummers Method**: Graphite is oxidized using a mixture of sulfuric acid, phosphoric acid, potassium permanganate, and optionally other oxidants like potassium ferrate. The mixture is heated and stirred under controlled conditions to form GO.

- **Modified Hummers Method**: Similar to the Hummers method but with modifications to improve safety and efficiency. For example, using an ice bath for better temperature control and specific ratios of chemicals.

2. **GO Reduction**:

- **Chemical Reduction**: GO is reduced using chemical reagents such as hydrazine, sodium borohydride, or glucose. The GO dispersion is heated in an oil bath or microwave to facilitate the reduction process.

- **Thermal Reduction**: GO is thermally reduced in an inert atmosphere (e.g., argon with a small amount of hydrogen) at high temperatures (typically 800°C) to remove oxygen-containing functional groups and achieve the reduced form.

3. **Specific Steps for rGO Production**:

- **Hydrothermal Reduction**: GO is dispersed in a solvent (e.g., ethylene glycol) and reduced using reductants like hydrazine. The dispersion is subjected to hydrothermal treatment under high pressure and temperature.

- **Centrifugation and Washing**: After reduction, the product is centrifuged to remove unreacted chemicals and washed with solvents to achieve a clean rGO dispersion.

These steps can vary slightly depending on the specific method and reagents used, but they generally follow these fundamental procedures to produce rGO from GO.

Ground truth

After GO is produced from the modified Hummer's method the GO can be reduced chemically using a reducing agent such as hydrazine, ascorbic acid or even sugars to reduce it to reduced graphene oxide (rGO). Alternatively, GO can be thermally reduced by heating it up at high temperature in an inert atmosphere like argon or in the presence of hydrogen. This will break down the oxygen-containing groups on the GO causing them to volatilize and restore the carbon framework. it should be nmoted that this process is often incomplete and bcan produce rGOP with a lot of defects that affect conductivity.

Question 11

"What are the steps involved to produce graphene quantum dot?

Context 1

Author: Satyaprakash Ahirwar

Doi: 10.1021/acsomega.7b01539

Method:

Graphite rods are purchased from Alfa Aesar. The graphite rods with a diameter of 3.05 mm and an approximate length of 60 mm are heated in a furnace (at 1050°C for 5 min) in the presence of air. After 5 min, the graphite rods are allowed to cool at room temperature. Thereafter, the graphite rods are washed with Milli-Q water to remove large particles from the surface of the graphite rods. The graphite rods are then used as anode and cathode, i.e., electrodes to be dipped in the electrolyte. Four types of graphene quantum dots (GQDs) are prepared i.e., GQD1−GQD4. For GQD1−GQD4, the electrolyte used is a mixture of citric acid monohydrate (0.1 M) and NaOH (0.15M (GQD1), 0.2M (GQD2), 0.3M (GQD3) and 0.4M (GQD4)) in Milli-Q water (50 mL). Thereafter, the electrochemical experiments are done with CHI660D Electrochemical Workstation. The separation between the graphite rods is ca. 25 mm. Cyclic voltammetry (CV) is performed prior to performing chronoamperometry. CV is performed with a voltage range of −1 to +1 V, to wet the graphite electrodes. Thereafter, chronoamperometry is performed with a voltage of 10 V and a sensitivity (I/V) of 0.1 for 30 min. As a result, the color of the electrolyte solution changes from colorless to yellow, which confirms the exfoliation of graphite rod. After completion of this reaction, calcium chloride (0.15 M) is added to the prepared solution. This solution is slightly heated to precipitate calcium citrate. Centrifugation is performed for 15 min at 10 000 rpm twice to separate the calcium citrate precipitate. The supernatant is thereafter filtered through membrane filtration (Himedia, Dialysis Membrane-150, LA401, pore size ca. 2.4nm) for 7 days to remove salt from the solution.

Context 2

Author: Ashwin Kumar Narasimhan

Doi: 10.1039/c7ra10702a

Method:

Synthesis of Graphene Quantum Dots (GQDs): Here we used pure highly oriented pyrolytic graphite (HOPG) plate as a source immersed in 5 mL of polyethylene glycol and 5 mL Milli-Q watersolution (1 : 2). A focused nanosecond pulsed laserwas optically directed to the graphite plate. The laser source was operated at 1064 nm with specific parameters (energy = 40 mJ, pulse duration = 6 ns,frequency = 10 Hz). After 30 minutes of ablation on graphite plate (3 mm thickness), single wall graphene layers and reduced GQDs were produced in the PEG–water

solution. For characterization studies, the solution was centrifuged at 15000 rpm for 30 minutes to remove large particles and graphene layers. We denote this preparation as control GQDs. Furthermore, the control-GQDs solution was refluxed at 200°C for 20 minutes and 1 hour. After heating for 20 min and 1 h the water evaporates completely, and the final product contains pegylated GQDs. We denote the resulting preparations as 20 min and 1 h GQDs corresponding to refluxing times of 20 minutes and 1 hour respectively.

Context 3

Author: Ling-Jun Wang

Doi: 10.1063/1.3533021

Method:

The graphene flakes were produced by mechanical cleaving of graphite crystallites by Scotch tape and then were transferred to a highly doped Si substrate with a 100 nm SiO2 top layer. Thin flakes were found by optical microscopy, and single layer graphene flakes were selected by the Raman spectroscopy measurement. We used the standard electron beam lithography and lift off technique to make the Ohmic contact (Ti/Au) on the present graphene devices. Next, a new layer of poly(methyl methacrylate) is exposed by electron beam to form a designed pattern. Then, the unprotected areas are removed by oxygen reactive ion etching. The quantum dot is an isolated central island of diameter 90 nm, connected by 30 nm wide tunneling barriers to source and drain contacts. Here, the Si wafer was used as the back gate and there is also a graphene side gate near the small dot. The SET has a similar pattern while the conducting island has a much larger diameter (180 nm).

Context 4

Author: Ruquan Ye

Doi: 10.1038/ncomms3943

Method:

Synthesis of graphene quantum dots (GQDs) from coal: In a typical procedure, 300 mg of coal was suspended in concentrated sulphuric acid (60 ml) and nitric acid (20 ml), and followed by cup sonication (Cole Parmer, model 08849−00) for 2 h. The reaction was then stirred and heated in an oil bath at 100 or 120 °C for 24 h. The solution was cooled to room temperature and poured into a beaker containing 100 ml ice, followed by adding NaOH (3 M) until the pH was 7. The neutral mixture was then filtered through a 0.45-μm polytetrafluoroethylene membrane and the filtrate was dialyzed in 1,000 Da dialysis bag for 5 days. For the larger GQDs synthesized from anthracite (a-GQDs), the time can be shortened to 1 to 2 h using cross-flow ultrafiltration (Spectrum Labs, KrosFlo Research IIi TFF system with 3 kD cutoff membrane). After purification, the solution was concentrated using rotary evaporation to obtain solid GQDs.

Context 5

Author: Sha Li

Doi: 10.1038/s41598-025-96207-z

Method:

High-quality graphene was grown in a BM Pro 2×8” Chemical Vapour Deposition (CVD) furnace from Aixtron Ltd., using methane (CH4) as precursor and copper (Cu, 200 mm × 200 mm foil) as the catalyst. Semi-dry transfer was carried out using sacrificial polymeric support placed on the graphene surface and the Cu foil was etched away using ammonium persulfate (APS) etchant. After rinsing the bottom surface of the graphene with deionised (DI) water, it was dried and stamped on the target substrate using uniaxial pressure. Finally, the polymer was removed using solvents. Local back-gated graphene field-effect transistor (GFETs) were fabricated on 150 mm and 200 mm Si wafers with 90 nm silicon dioxide (SiO2) using standard CMOS-compatible photolithography technology. Firstly, the titanium/palladium (Ti/Pd) local back gate with a thickness of 5/40 nm was evaporated onto the substrate, followed by a standard metal lift-off process. A 75 nm thick Al2O3 layer was subsequently deposited via the atomic layer deposition (ALD) method to form the dielectric layer Afterward, the bottom contact of 30 nm Pd was evaporated. After transfer, graphene was patterned using reactive ion etching (RIE) by oxygen plasma and then a final layer of 40 nm Pd was evaporated onto the substrate for top contacting. The QDs were obtained from Quantum Solutions and had diameters on the order of 6–7 nm according to the data sheet. QD deposition was performed by spin-coating a total of 12 layers of PbS QDs via layer-by-layer ligand replacement in an inert atmosphere glovebox to minimize exposure to water and oxygen, followed by encapsulation with an aluminum oxide layer deposited via an ALD tool. The oleic acid ligands were replaced with ethanedithiol (EDT) or benzene dithiol (BDT) by in-situ ligand replacement. There are two different sizes of QDs with two different ligands, resulting in a 4-4-4 absorber-type arrangement (4 coatings of QDs with a 0.64 eV bandgap, another 4 coatings of QDs with a 0.64 eV bandgap, and finally, 4 coatings of QDs with a 1.25 eV bandgap), with an overall thickness on the order of 300 nm. The absorber layer was then removed everywhere except on the graphene channels of the GFETs, using lithographic patterning and a solution-based etching process (concentrated HCl and HI acid solutions). The devices were finally encapsulated with Al2O3 using an ALD process.

RAG-Qwen

To produce graphene quantum dots (GQDs), several methods have been reported in the literature. Here are the general steps involved based on the provided contexts:

1. **Graphite Source Preparation**:

- **Satyaprakash Ahirwar**: Graphite rods with a diameter of 3.05 mm and a length of approximately 60 mm are heated in a furnace at 1050°C for 5 minutes in the presence of air. After cooling, these rods are washed with Milli-Q water to remove large particles.

2. **Electrochemical Exfoliation**:

- **Satyaprakash Ahirwar**: Electrochemical exfoliation is performed using graphite rods as anodes and cathodes in an electrolyte solution consisting of citric acid and sodium hydroxide. The electrolyte concentration varies for different types of GQDs (GQD1-4). Cyclic voltammetry (CV) is performed first to wet the graphite electrodes, followed by chronoamperometry at 10 V for 30 minutes. This process results in the exfoliation of graphite rods, leading to the

formation of GQDs. After the reaction, calcium chloride is added to precipitate calcium citrate, which is then separated through centrifugation and dialysis.

3. **Laser Ablation**:

- **Ashwin Kumar Narasimhan**: Pure highly oriented pyrolytic graphite (HOPG) is ablated using a focused nanosecond pulsed laser. The laser parameters include energy, pulse duration, and frequency. After 30 minutes of ablation, single-wall graphene layers and reduced GQDs are produced in the PEG-water solution. The solution is then centrifuged to remove large particles and graphene layers, yielding control GQDs. Further processing involves refluxing the solution at 200°C for 20 minutes or 1 hour to produce pegylated GQDs.

4. **Mechanical Exfoliation and Transfer**:

- **Ling-Jun Wang**: Graphene flakes are mechanically cleaved from graphite crystals using Scotch tape and transferred to a highly doped Si substrate with a 100 nm SiO2 top layer. Single layer graphene flakes are identified using Raman spectroscopy, and Ohmic contacts are made using electron beam lithography and lift-off techniques. Quantum dots are isolated islands of graphene connected by tunneling barriers to source and drain contacts.

5. **Chemical Synthesis from Coal**:

- **Ruquan Ye**: Coal is suspended in concentrated sulfuric acid and nitric acid, followed by sonication and heating in an oil bath at 100 or 120°C for 24 hours. The solution is then neutralized with NaOH and filtered. Dialysis is performed over several days to purify the GQDs, and the solution is concentrated using rotary evaporation.

6. **Synthesis Using CVD**:

- **Sha Li**: High-quality graphene is grown using chemical vapor deposition (CVD) in a furnace. After graphene transfer and patterning, quantum dots (QDs) are deposited by spin-coating a total of 12 layers of PbS QDs via layer-by-layer ligand replacement. The QDs are encapsulated with an aluminum oxide layer, and the device is encapsulated with Al2O3 using atomic layer deposition (ALD).

These methods demonstrate the diversity in approaches to producing GQDs, ranging from electrochemical exfoliation, laser ablation, mechanical exfoliation, chemical synthesis, and CVD growth.

Ground truth

Graphene Quantum Dots (GQDs) can be produced using hydrothermail reactions, laser ablation, microwave radiation and electrochemical oxidation. THe most straightforward is that of electrochemical oxidation. The electrochemical oxidation process involves the electrochemical cleavage of carbon precursores such as graphite into GQDs, typically under high voltage. First a potential is applied to a graphite electrode immersed in an electrolyte made of citric acid and sodium hydroxide. A second graphite rod is used as an cathode in the same solution. A DC power supply can provide the working bias voltage. The GQDs eventually exfoliate and dispersed into the electrolyte. The resulting GQDs can be purified by centrifugation and further techniques such as rotary evaporation.

Question 12

What are the steps involved to produce graphene nanoribbon?

Context 1

Author: César Moreno

Doi: 10.1126/science.aar2009

Method:

Our method relies on the hierarchical control of three thermally activated reaction steps, labeled T1 to T3. Nanoribbons and pores with nanometer size, atomic-scale uniformity, and long-range order are formed in separate steps. Graphene nanoribbons (GNRs) are first synthesized by following a previously used route, consisting of the surface-assisted Ullmann coupling of aromatic dihalide monomers into polymer chains (T1) and the cyclodehydrogenative aromatization of the intermediate polymeric chains into GNRs (T2). The final step (T3) interconnects GNRs laterally in a reproducible manner by means of a highly selective dehydrogenative cross-coupling. This step requires a careful design of the monomer precursor, which defines the edge topology of the resulting GNR that is necessary for a high yield and selectivity of the cross-coupling reaction. The monomer precursor synthesized in this work, labeled DP-DBBA (diphenyl–10,10′-dibromo-9,9′-bianthracene), is a derivative of theDBBA used in the synthesis of seven–carbon atom–wide armchair GNRs (7-AGNR), with phenyl substituents added at (2,2′) sites. The latter is the key element for the promotion of the inter-GNR connections that lead to the NPG structure. The choice of catalytic surface is also relevant for the selection of the reaction paths that define the intermediates and for the separation of thermal windows that lead to their hierarchical control. Here we use the Au(111) surface, where each reaction step has a different thermal activation onset. The NPG can then be transferred to suitable substrates in which its functionalities can be exploited.

Context 2

Author: Junan Fang

Doi: 10.1016/j.nanoms.2025.02.010

Method:

Preparation of cove-type graphene nanoribbon (cGNRs-Dipyrene:) In a dried 500 mL flask add 500 mg of Poly-Dipyrene and 300 mL of anhydrous dichloromethane. Under nitrogen protection, degas the mixture using a dry diaphragm pump three times for approximately 10 min. Dissolve 9.94 g of anhydrous FeCl3 in 15 mL of anhydrous CH3NO2 (acetonitrile), degas it, and drip it into the Poly-Dipyrene solution under a nitrogen atmosphere while stirring. Maintain stirring and pass nitrogen gas continuously through the solution at ambient temperature for 3 d. Once the reaction is done, most of the dichloromethane is removed at amlower temperature. Add 400 mL of methanol, stir for 10 min, and allow it to rest for 6 h. Decant the supernatant and collect the cloudy lower phase for spinning in a centrifuge. Obtain the black precipitate and perform extraction using the Soxhlet method with methanol. The

collected black solid is the final product compound, cGNRs-Dipyrene, with a total weight of 480 mg and a yield of 94 %.

Context 3

Author: Young Duck Kim

Doi: 10.1038/nnano.2015.118

Method:

(1) Mechanically exfoliated graphene was prepared on a SiO2/Si substrate. (2) A PMMA (Polymethyl methacrylate, 950K C4) was spin coated on graphene at 4500 rpm followed by baking process at 180°C for 5 minutes. (3-4) To make a patterned graphene array, PMMA on unwanted areas of graphene was exposed by e-beam lithography, and the remaining PMMA after development acted as an etch mask during O2 plasma etching. (5) Patterned graphene array was prepared after removing PMMA with acetone. (6) PMMA was spin coated again on the patterned graphene ribbons using the same recipe as in step (2). (7) PMMA membrane with graphene ribbons was separated from SiO2/Si substrate in 10% (wt) potassium hydroxide water solution (KOH). (8) The separated PMMA membrane with attached graphene was rinsed with DI-water to remove the KOH residue from the graphene surface and dried at room temperature in Nitrogen atmosphere. (9) The position of the PMMA membrane with patterned graphene arrays was manipulated on prepared trench substrate (depth: 300 ~ 1100 nm) using home-made micro-position aligner. (10) Using micro contact transfer method, each side of the graphene ribbons were attached to the Au electrodes of the prepared trench. (11) The PMMA layer was removed by an acetone wash followed by an IPA (Isopropanol) rinse. The suspended ME graphene devices are completed after a critical point drying process.

Context 4

Author: Xiaoyin Yang

Doi: 10.1021/ja710234t

Method:

Compound 1: Magnesium turnings (3.6 g, 150 mmol) were placed in an Ar-flushed flask and dry THF (10 mL) was added. 1,2-Dibromoethane (0.2 mL) was added and the resulting mixture was stirred at room temperature (rt) for a few minutes. After the reaction ceasing, the solution was removed via cannulation and dry THF (100 mL) was added. Then, a solution of 1-bromo-4-(3,7-dimethyloctyl)benzene (22 g, 100 mmol) was slowly added at room temperature. The reaction starts within a few minutes. After addition, the reaction mixture was stirred for 12 h at room temperature. The grey solution of 4-(3,7- dimethyloctyl)phenyl magnesium bromide was then cannulated into a suspension of 1,4-dichloro-2,3,5,6- tetrabromobenzene (4.6 g, 10 mmol) in dry THF (20 mL) under argon and the resulting mixture was stirred an rt for 12 h. I2 (25 g, 100 mmol) was added directly to the reaction mixture at 0°C and the reaction was stirred at rt under argon for 2 h. The reaction was quenched with water and the resulting mixture was extracted with CHCl3 (100 mL × 3). The combined organic layers were washed with 2M aqueous NaHSO3 solution (200 mL × 2) and brine (50 mL), dried with MgSO4 and concentrated in vacuum. The residue was purified with chromatography (Silica gel, n-

Hex./CH2Cl2 = 20/1) to give 7.7 g of compound 1 as a thick oil. The yield is 65%. Compound 2: A suspension of compound 1 (1.2 g, 1.0 mmol), 4-bromophenylboronic acid (800 mg, 4.0 mmol), K2CO3 (2.76 g, 20 mmol) and Aliquat ® 336 (8 mg, 0.02 mmol) in toluene (30 mL) was degassed three times via 'freeze-pump-thaw' cycles. Pd(PPh3)4 (58 mg, 0.05 mmol) was added quickly and the reaction mixture was degassed three times again via 'freeze-pump-thaw' cycles. Then the reaction was allowed to be stirred at 80°C under argon for 18 h. The reaction was quenched with water (20 mL) and the resulting mixture was extracted with dichloromethane (30 mL × 3). The combined organic layers were washed with brine (30 mL), dried with MgSO4 and concentrated in vacuum. The residue was purified by column (Silica gel, nHex./CH2Cl2 = 20/1) to give compound 2 as a white solid (1.17 g, Yield = 93). Compound 3: To a solution of compound 2 (600 mg, 0.48 mmol) in dry THF (8 mL) under argon at -78 °C, n-BuLi(1.6 M/n-Hexane, 0.78 mL, 1.24 mmol) was added and the resulting solution was stirred at – 78 o C for 1 h. Then 2-isopropoxy-4,4,5,5-tetramethyl-[1,3,2]dioxaborolane (267 mg, 1.44 mmol) was added at -78 °C and the reaction was allowed to be stirred at rt for 3 h. The reaction was quenched with water (5 mL) and the resulting mixture was extracted with dichloromethane (15 mL × 3). The combined organic layers were washed with brine (10 mL), dried with MgSO4 and concentrated in vacuum. The residue was purified by column (Silica gel, n-Hex./CH2Cl2 = 4/1) to give compound 3 as a thick oil (530 mg, Yield = 82%). Polymer 4: A mixture of compound 1 (89 mg, 0.074 mmol), compound 3 (100 mg, 0.074 mmol), K2CO3 (2 M/H2O, 2 mL, 4 mmol) and Aliquat® 336 (0.6 mg, 0.0015 mmol) in toluene (5 mL) was degassed three times via 'freeze-pump-thaw' cycles. Pd(PPh3)4 (4.3 mg, 0.004 mmol) was added quickly and the reaction mixture was degassed three times again via 'freeze-pump-thaw' cycles. The reaction was refluxed under argon for 3 days. Then, a degassed solution of bromobenzene (15 mg, 0.1 mmol) in toluene (0.5 mL) was added into the reaction mixture via a syringe. After refluxing for 12 h, a degassed solution of phenylboronic acid (12 mg, 0.1 mmol) in toluene (0.5 mL) was added into the reaction mixture via a syringe and the reaction was refluxed for another 12 h. The reaction mixture was poured into a mixture of methanol (200 mL) and con. aqueous HCl solution (30 mL) and stirred overnight. The resulting black solid was filtered off and subjected to Soxhlet extraction for 2 days in acetone. The residue was redissolved in hot THF and precipitated again in methanol. The solid was filtered, washed with methanol and dried in vacuum to give polymer 4 as a grey solid (112 mg, Yield = 75%). Graphene Nanoribbon 5: A suspension of polymer 4 (10 mg) in freshly distilled CH2Cl2 (200 mL) was purged with argon for 15 mins. Then, a solution of FeCl3 (114 mg, 0.7 mmol) in CH3NO2 (0.3 mL) was added dropwise and the resulting black solution was stirred at rt for 12 h with bubbling of argon. Stopped the argon bubbling and the reaction mixture was stirred at rt for 36 h. The solvent was removed under argon and THF (1 mL) was added to redissolved the reaction mixture. The resulting solution was dropped into a mixture of methanol (100 mL) and con. aqueous HCl solution (30 mL) and stirred overnight. The resulting black solid was filtered off and subjected to Soxhlet extraction for 2 days in acetone. The residue was redissolved in hot THF and precipitated again in methanol. The solid was filtered, washed with methanol and dried in vacuum to give polymer 5 as a black solid (6.4 mg, Yield = 65%).

Context 5

Author: Néstor Merino-Díez

Doi: 10.1021/acs.jpclett.8b00796

Method:

This is demonstrated using 4,4”-dibromoterphenyl (DBTP) as a molecular precursor and two different gold surfaces as substrate, namely Au(111) and Au(322). DBTP molecules adsorbed on Au surfaces are known to undergo a series of chemical reactions upon annealing. In our experiments, the Au(111) and Au(322) surfaces were cleaned similarly, by standard Ar+ sputtering and annealing cycles. The molecules were then deposited on the surfaces by means of a home-built Knudsen cell heated to ~115 °C during sublimation. During the deposition, the surfaces were held at room temperature and annealed thereafter to subsequently trigger the different reaction steps. We then anneal the samples to 180°C to trigger the polymerization of DBTP into poly paraphenylene (PPP) through Ullmann coupling, whereby single Br atoms are left on the surface as byproducts, typically forming rows sandwiched between the PPP chains. Further increasing the temperature, the Br atoms desorb from the substrate, allowing the PPP chains to approach each other and fuse as the cyclodehydrogenation is activated at temperatures around 380 °C. As a result, wider armchair-oriented graphene nanoribbons (aGNRs) form, whose width is determined by the number of participant PPP wires. Quantified by the number of dimer lines across the aGNR , the resultant widths thus correspond to multiples of three (3naGNR, n being the number of fused PPP). Since aGNRs are classified into three families depending on their number of dimer lines (3p − 1, 3p, and 3p + 1, p being an integer), all nanoribbons synthesized in these experiments thus correspond to the same 3p family.

RAG-Qwen

The production of graphene nanoribbons involves several steps, which can vary depending on the specific method used. Here are some general steps based on the provided contexts:

1. **Synthesis of Monomers or Precursors**:

- **DP-DBBA Synthesis**: The monomer precursor, DP-DBBA, is synthesized by adding diphenyl to 10,10′-dibromo-9,9′-bianthracene at (2,2′) sites.

- **Poly-Dipyrene**: Poly-Dipyrene is dissolved in dichloromethane, and a solution of FeCl3 in acetonitrile is added under nitrogen protection.

- **Magnesium Turnings Reaction**: A series of reactions involving magnesium turnings, 1,2-dibromoethane, 1-bromo-4-(3,7-dimethyloctyl)benzene, 1,4-dichloro-2,3,5,6-tetrabromobenzene, iodine, and other reagents to form a complex polymer.

2. **Surface Preparation and Deposition**:

- **Au(111) Surface Annealing**: DBTP molecules are deposited on Au(111) and Au(322) surfaces and annealed to trigger Ullmann coupling and subsequent cyclodehydrogenation.

- **PMMA Spin Coating**: PMMA is spin-coated on graphene and used as a mask for patterning graphene ribbons through e-beam lithography and O2 plasma etching.

3. **Formation of Graphene Nanoribbons**:

- **Hierarchical Control of Reactions**: For the DP-DBBA method, GNRs are formed in three steps (T1 to T3) involving polymerization, aromatization, and lateral interconnection.

- **Annealing for Polymerization and Cyclodehydrogenation**: For DBTP on Au surfaces, annealing triggers Ullmann coupling and cyclodehydrogenation to form wider armchair-oriented graphene nanoribbons (aGNRs).

4. **Post-Synthesis Processing**:

   - **Extraction and Purification**: For some methods, the products are extracted and purified using techniques like Soxhlet extraction or precipitation in solvents.

   - **Transfer to Substrates**: The graphene nanoribbons are transferred to suitable substrates for further functionalization or study.

These steps provide a comprehensive overview of the processes involved in producing graphene nanoribbons, adapted from the provided contexts.

Ground truth

Graphene nanoribbons (GNRs) are narrow strips of single-layer graphene, essentially 'slices' of graphene that are one-dimensional. They have very high surface areas. They can be synthesized either by top-down approaches such as micromechanical cleavage or bottom-up approaches such as CVD. Top down approaches usually use multi-walled Carbon nanotubes (MWCNTs) to form these graphene nanoribbons. For instance usuing the hummers method on MWCNTs to form the ribbons instead of bulk graphite. Bottom up-approaches using CVD have highly-controlled conditions for growth to ensure orientation and length.

Question 13

What is a method of exfoliating graphene from highly oriented pyrolytic graphite (HOPG) crystal?

Context 1

Author: Yuan Huang

Doi: 10.1021/acsnano.5b04258

Method:

We used SiO2/Si as the substrate and ordinary adhesive tape as the transfer medium. Contact with the tape transfers thick graphite flakes from a highly oriented pyrolytic graphite (HOPG) crystal. Prior to exfoliating thin graphene from these flakes, the SiO2/Si substrate is ultrasonically cleaned in acetone, 2-propanol, and deionized (DI) water, and then subjected to oxygen plasma to remove ambient adsorbates from its surface. Following the plasma cleaning step, the graphite-loaded tape is brought in contact with the substrate. Instead of immediately removing it to complete the exfoliation, the substrate with the attached tape is annealed for 25 min at~100°C, in air on a conventional laboratory hot plate. After the sample is cooled to room temperature, the adhesive tape is removed, which completes the exfoliation.

Context 2

Author: Dan Li

Doi: 10.1038/nnano.2007.451

Method:

Graphite oxide was synthesized from natural graphite (SP-1, Bay Carbon) by a modified Hummers method as originally presented by Kovtyukhova and colleagues. As-synthesized graphite oxide was suspended in water to give a brown dispersion, which was subjected to dialysis to completely remove residual salts and acids. Ultrapure Milli-Q® water was used in all experiments. As-purified graphite oxide suspensions were then dispersed in water to create a 0.05 wt% dispersion. Exfoliation of graphite oxide to graphene oxide (GO) was achieved by ultrasonication of the dispersion using a Brandson Digital Sonifier (S450D, 500 W, 30% amplitude) for 30 min. The obtained brown dispersion was then subjected to 30 min of centrifugation at 3,000 r.p.m. to remove any unexfoliated graphite oxide (usually present in a very small amount) using an Eppendorf 5702 centrifuge with a rotor radius of 14 cm. In a typical procedure for chemical conversion of graphite oxide to graphene, the resulting homogeneous dispersion (5.0 ml) was mixed with 5.0 ml of water, 5.0 μl of hydrazine solution (35 wt% in water, Aldrich) and 35.0 μl of ammonia solution (28 wt% in water, Crown Scientific) in a 20-ml glass vial. The weight ratio of hydrazine to GO was about 7:10. After being vigorously shaken or stirred for a few minutes, the vial was put in a water bath (~95 °C) for 1 h. Unless specifically stated, graphene dispersions prepared according to the above

procedure were used for further characterization and film fabrication in this work. The graphene paper was made by filtration of a dispersion through an Anodisc membrane filter (47 mm in diameter, 0.2 μm pore size; Whatman), similar to the method reported for making GO paper. The graphene paper was cut by a razor blade into rectangular strips of approximately 4 mm × 15 mm for mechanical testing.

Context 3

Author: Vincent Huc

Doi: 10.1088/0957-4484/20/17/179801

Method:

The proposed method is somewhat reminiscent of the process developed for the industrial production of thin silicon-on-insulator wafers . A thin "layered" crystalline film is bonded and firmly mechanically supported during the thinning. Our process mainly consists of two steps: Firstly, graphite bonding is realized using a thin layer of epoxy resist which is subsequently cured under pressure. Secondly the bonded graphite is exfoliated using the scotch tape technique. A freshly cleaved highly oriented pyrolytic graphite crystal is glued using very thin epoxy-based glue. After curing, the glue leaves creates a transparent, void and bubble-free interlayer between the substrate and the highly oriented pyrolytic graphite sample. This fabrication method can be refined by using a pre-cut (<100μm thick) flexible highly oriented pyrolytic graphite sample as the top layer. This method may appear as a refinement of the exfoliation process known as the "scotch-tape" technique. However significant differences do exist with respect to that well-known method. Firsly one should note that the useful graphene sheets is the one left on the adhesive side (i.e. the epoxy underlayer) after the curing step (hence the name "reverse exfoliation"). This leads to larger and flatter flakes with very few pleats. The second one is that a uniaxial compression is applied to the sample during the adhesive curing step, in order to increase the flatness of the adhesive interlayer. The third one is that the adhesive used in this study was found to be compatible with conventional micro/nanofabrication techniques including lithographic and etching processes. This allows transport measurements to be performed in-situ directly on the bonded monolayer.

Context 4

Author: G. Rajendra Kumar

Doi: 10.1039/C5RA24810H

Method:

In a typical procedure, 50 g of graphite flakes and 80 g of oxalic acid (OA) were placed into a stainless steel jar (2500 ml volume) containing 10 mm diameter stainless steel balls, which were used as the grinding medium. Low diameter stainless steel balls were utilized to increase the impact and shear energy between the balls and powder, and to have a low dimensional interaction with the graphite layers. Initially, the dual-drive ball milling was executed at three different critical speeds (40%, 70%, and 98%), but the critical speed of 40% offers efficient exfoliation with less defects than 70% and 98%. Accordingly, the optimized critical speed of 40% was adopted for graphite exfoliation using shear force ball milling. The milling was carried

out for up to 20 h and the rest time was one hour per cycle. Then, the milled graphite was treated with 1 M hydrochloric acid (HCl) to acidify the carboxylates and to remove the iron impurities from the milling jar. Then, the mixture was washed with distilled water several times until the pH was neutral. The mixture was filtered and subjected to heat treatment at 600 °C under an argon atmosphere for an appropriate time interval. Finally, as-prepared graphene was stored for future use.

Context 5

Author: Arjunan Ariharan

Doi: 10.4236/graphene.2016.52005

Method:

Graphene Oxide (GO) was synthesized from graphite by a modified Hummers method. Briefly, graphite powder (2.0 g) was taken in a beaker, added 50 m of concentrated sulfuric acid (H2SO4) under vigorous agitation in an ice bath. Afterwards, sodium nitrate (2.0 g) and potassium permanganate (6.0 g) were slowly added in a sequence. Then, the mixture was transferred into a water bath and kept at 35˚C for 2 hour. After that, 100 mL of distilled water was slowly added, causing a temperature rise to 98˚C. Later, 140 mL of 4% H2O2 was dropped into the reaction system. Finally, the product was washed with distilled water three times. The acquired solid was dried in vacuum at 50˚C for 48 hours.

RAG-Qwen

One method of exfoliating graphene from a highly oriented pyrolytic graphite (HOPG) crystal involves the use of a SiO2/Si substrate and adhesive tape. Specifically:

1. The SiO2/Si substrate is first ultrasonically cleaned in acetone, 2-propanol, and deionized (DI) water, followed by oxygen plasma treatment to remove ambient adsorbates from its surface.

2. A graphite-loaded tape, which has been loaded with thick graphite flakes from the HOPG crystal, is brought into contact with the cleaned substrate.

3. Instead of immediately removing the tape, the substrate with the attached tape is annealed for 25 minutes at approximately 100°C on a conventional laboratory hot plate.

4. After cooling to room temperature, the adhesive tape is carefully removed, leaving behind exfoliated graphene on the substrate.

This method, described in the work of Yuan Huang (DOI: 10.1021/acsnano.5b04258), is a common approach for obtaining high-quality graphene sheets from HOPG crystals.

Ground truth

HOPG is essentially highly oriented graphite which is ideal for producing graphene using mechanical cleavage with adhesive tape. HOPG can be purchased commercially and then exfoliated through existing processes such as micromechanical exfoliation tape to reult in high-quality graphene with minimal defects.

Question 14

How to synthesize graphene using PET bottles?

Context 1

Author: Aamir Ahmed

Doi: 10.1016/j.mseb.2024.117828

Method:

Synthesis of carbon: The bubble wraps were washed 5-times with DI water. After drying, these sheets were cut into tiny pieces and put into a petri dish. The dish was then heated at 100 °C to melt the pieces followed by solidifying. These solidified sheets were chopped into small pieces and immersed in a beaker with concentrated H2SO4. The beaker containing the sample was transferred to a muffle furnace and subjected to thermal treatment at 150 °C for a duration of 3 h. The excess sulphuric acid was drained out and the resultant was a black hard mass. The acquired solid was subjected to repeated washing with deionized water until a neutral pH was attained. This sulphonation step was followed by carbonization. The centrifuged sample was heated in a muffle furnace at 500 °C for 1 h. The sample then obtained was washed with DI water, dried at room temperature, and reduced to powder using mortal and pestle. Conversion of carbon into reduced graphene oxide (rGO): In this process, 1 g synthesized carbon and 1 g NaNO3 were added to 45 ml of H2SO4 (kept in an ice bath) and stirred for 30 min until a homogenous black solution is produced. To this solution, 5 g KMnO4 was slowly added and stirred at 50 °C for 2 h forming a green solution. 100 ml DI water and 6 ml H2O2 (35 %) were added drop-by-drop to stop the reaction. The precipitates were washed with 300 ml HCl (10 %) and 200 ml HCl (37 %) in a centrifuge. The final sample was heated at 250 °C in a muffle furnace, again washed with DI water, and reduced to rGO powder.

Context 2

Author: Jian-Hao Chen

Doi: 10.1002/adma.200701059

Method:

First, photolithography is used to prepare 30 nm thick Au source and drain electrodes on a silicon wafer with an oxidized surface (SiO2/Si). The electrodes are then transferred onto the Polyethylene Terephthalate (PET) substrate as described elsewhere. Then, single-and few-layer graphene is obtained from Kish graphite by mechanical exfoliation on 300 nm thick thermally grown silicon dioxide on silicon substrates, and its thickness and morphology characterized by atomic force microscopy. Mechanical exfoliation yields atomically clean graphene sheets and our AFM images also indicate that the graphene sheet is free of nanometer-scale contaminants. In addition, chemical contamination caused by exposure to photoresist and lift-off chemicals is avoided in this process. The desired graphene sheet is printed at 170°C at 500 psi from the SiO2/Si substrate to the source-drain electrode assembly on PET.

Context 3

Author: Subash Sharma

Doi: 10.1016/j.carbon.2014.01.051

Method:

A horizontal ambient pressure (AP) chemical vapour deposition (CVD) system with a quartz tube of length 90 cm and diameter of 50 mm was taken as the reactor placed in two split furnaces. Cu foil (Nilaco Corp.) of thickness 20 μm and purity 99.99% was used as substrate for graphene synthesis. Solid waste plastic used for material packaging is used as carbon source in these studies. Cu foil was cleaned by sonicating in acetone for 30 min and placed in the growth furnace without further treatment. Cu substrate was annealed for 30 min in 100 standard cubic centimeters per minute (sccm) of H2 at 1020 °C before the feedstock was introduced. Annealing is an important step to enlarge Cu grain size, reduce lattice mismatch and dislocations, which help to limit number of nucleation sites. 3 mg of waste plastic as carbon source was placed in a ceramic boat inside the lower temperature furnace. Graphene growth was carried out for 90 minutes using a gas mixture of Ar and H2 with the flow rate 98 and 2.5 sccm, respectively. During the growth process, the rate of increase in pyrolysis temperature for the waste plastic feedstock was optimized as 1.5 °C/min and the pyrolysis temperature was increased gradually to around 480 °C until all the precursor was evaporated. Supply of precursor was continuously increased during the growth process to provide sufficient carbon atoms for uninterrupted growth of graphene crystal. The injection rate of polymeric components contained in the waste plastic was changed by the rate of evaporation. Thereafter, it was allowed to cool at a cooling rate of (~16°C/min). Individual graphene crystal was transferred to SiO2/Si substrate by coating a poly(methyl methacrylate) (PMMA) supporting layer. Etching of underneath Cu foil was achieved by using a 50 mg/ml concentrated Fe(NO3)3 solution. Subsequently, PMMA/graphene stack layer was transferred to SiO2/Si substrate and PMMA coating was dissolved in acetone. Finally, transferred graphene was treated with a diluted nitric acid solution to remove the residual Fe(NO3)3 and dried in air.

Context 4

Author: Mohammad Choucair

Doi: 10.1038/nnano.2008.365

Method:

All solvothermal reactions were performed in a Teflon-lined Parr Instrument Company 4749 reactor having a maximum volume of 23 ml. A typical synthesis consists of heating a 1:1 molar ratio of sodium (2 g) and ethanol (5 ml) in a sealed reactor vessel at 220 °C for 72 h to yield the solid solvothermal product—the graphene precursor. This material is then rapidly pyrolysed, and the remaining product washed with deionized water (100 ml). The suspended solid is then vacuum filtered and dried in a vacuum oven at 100 °C for 24 h. The final yield of graphene is approximately 0.1 g per 1 ml of ethanol—typically yielding ~0.5 g per solvothermal reaction.

Context 5

Author: Noha A. El Essawy

Doi: 10.1016/j.ecoenv.2017.07.014

Method:

First, waste PET bottles were crushed and sieved to obtain the desired size fractions (1–3 mm) using a conventional sieve-shaker. Then 2 g of raw PET waste was introduced into an enclosed 50 mL stainless steel autoclave reactor (SS316). The closed stainless steel reactor was placed inside the center of an electric furnace and heated to 800 °C with a rate of 8 °C/min and maintained at this temperature for 1 h. After that the system was left to cool overnight. The resulted dark products were collected and crushed

RAG-Qwen

To synthesize graphene using PET bottles, follow the method outlined in the work by Noha A. El Essawy (DOI: 10.1016/j.ecoenv.2017.07.014):

1. **Preparation of PET Waste**: First, crush and sieve the waste PET bottles to obtain particles in the size range of 1–3 mm using a conventional sieve-shaker.

2. **Enclosed Autoclave Reaction**: Introduce 2 g of the crushed and sieved PET waste into a 50 mL stainless steel autoclave reactor (SS316).

3. **Heating Process**: Place the closed stainless steel reactor inside an electric furnace and heat it to 800 °C at a rate of 8 °C/min. Maintain the temperature at 800 °C for 1 hour.

4. **Cooling and Collection**: After the reaction time, allow the system to cool overnight. Collect the resulting dark products.

5. **Post-Treatment**: Crush the collected dark products if necessary to achieve a fine powder form.

This method transforms PET waste into a form that can potentially be converted into graphene through further processing steps such as chemical reduction or other graphene synthesis techniques.

Ground truth

Waste PET can be cleaned and then used as a precursor for pyrolysis into graphene. It usually takes a 2 step process. First, pyrolysis at 900 degrees centigrade into amorphous carbon. Next, the amorphous carbon is mixed with Boron powder and heat treated at 2400 degrees celsius for 1 hour under helium gas to form graphite. This high-temperature treatment with boron enables the carbon atoms to rearrange into the layered structure of graphite.. Yield rates are about 86%. This graphite can then be liquid-phase exfoliated to form graphene.

Question 15

What is the purpose of PMMA in the synthesis of graphene?

Context 1

Author: Cristina Vallés

Doi: 10.1016/j.carbon.2019.10.075

Method:

Synthesis of NH2 terminated graphene nanoplatelets (NH2-GNPs): NH2-GNPs were synthesized by a nucleophilic substitution reaction through a spontaneous diazonium coupling reaction. In a typical reaction, 150 mg of graphene nanoplatelets (GNP-M25 from XG Sciences, with lateral dimension and thickness of ~25 μm and ~6 nm, respectively, quoted by the manufacturer) was initially suspended in 125 ml of acetonitrile (CH3CN) together with 1.5 g of p-phenylene diamine and heated up to 60 °C under mechanical stirring. Once this temperature was reached, 2 mL of isoamylnitrite were added to the mixture and left to react under mechanical stirring for 24h. The resultant solid was then separated by vacuum filtration, rinsed first three times with CH3CN and then three times with EtOH and left to dry at room temperature. This material was labelled as NH2-GNPs. Synthesis of PMMA grafted graphene nanoplatelets (PMMA-NH-GNPs): NH2-GNPs and PMMA powders (50/85 mg) were dispersed together in chloroform (CHCl3) and the mixture was stirred at 70 °C for 24 h. After that time, the reaction mixture was cooled down to room temperature, filtered to collect the resultant powder, washed with 50 mL of CHCl3 and dried. This material was labelled as PMMA-NH-GNPs. (PMMA-NH-GNPs/PMMA) composite preparation: The appropriate amount of filler (PMMA-NH-GNPs or GNPs) and 1 g of PMMA were dissolved in 15 mL of CHCl3 using mechanical stirring at room temperature for 30 minutes to prepare composite materials (PMMA-NH-GNPs/PMMA or GNPs/PMMA) at various loadings from 0.5 to 5 wt.%. Composite films were prepared by depositing these dispersions on a Teflon dish using the solvent-casting method, followed by complete removal of the solvent and peeling-off of the composite films. Specimens with the desired shapes and sizes were cut out of these composite films for characterization.

Context 2

Author: Wei Wu

Doi: 10.1016/j.snb.2010.06.070

Method:

Briefly, the procedure is described as follows. Graphene was grown by thermal chemical vapour deposition (CVD) on a Cu foil, at a temperature of 1000°C and under 1 atm pressure with methane as the precursor gas. A quartz tube with 2 diameter was used as the reaction chamber for our CVD system. Cu foils were rolled up in a roll, but without the rolled-up layers touching to each other. Following the growth, poly(methyl methacrylate) (PMMA) was spun on graphene/Cu substrate to form PMMA/graphene/copper sandwich structure. Later, Cu foil was

etched away using an iron nitrate aqua solution. After the Cu foil was completely etched away, graphene with PMMA/graphene film was transferred onto a Si wafer with 300 nm thermally grown SiO2. The PMMA was then removed by repeatedly rinsing the film in acetone.

Context 3

Author: F.S. Al-Hazmi

Doi: 10.1016/j.synthmet.2014.12.028

Method:

5 g of graphite flakes (thickness ~0.1 μm) was first immersed into 30 ml of ice cooled glutaric acid over night. The pretreated graphite flakes were filtered and left to dry at ambient conditions. The pretreated graphite subjected to a thermal shock at 700 °C for 1 min to expand the graphite. 500 mg of the expanded graphite flakes were dispersed in 50 ml methanol and sonicated for 10 min to ensure the complete dispersion of pretreated graphite in the methanol solution. The graphite solution was introduced into a Teflon autoclave with capacity 80 cm3 and put into microwave oven at power of 800 W and temperature 200 °C for 30 min. After this time, the reaction was terminated by put the Teflon autoclave into ice cooled bath. The supernatant was collected by centrifuging and washed several times with deionized water and HCl (10%). The graphene sheets were dried under vacuum over night at 70 °C.

Context 4

Author: Xin Zhao

Doi: 10.1039/C9NR04720D

Method:

The raw graphite single flakes used to prepare the mechanically-cleaved monolayer graphene were purchased from Germany. The thermal release tape used to exfoliate graphite into graphene was supplied by Japan. Poly(methyl methacrylate) (PMMA) plates were purchased from UK and were subsequently cut into small beams of 70 mm in length and 20 mm in width, followed by polishing at the cut edges to minimize scratches and defects. This procedure allows the PMMA beams to be deformed to high strains (~2%) without fracture. Preparation of exfoliated monolayer graphene on PMMA: For the deformation and fracture studies of monolayer graphene, the monolayer graphene flakes were prepared by mechanical cleavage of single flake graphite. The graphite flake was initially placed in the middle of the Nitto tape and repeatedly peeled with another layer of tape. At the end of this procedure, the material that remained on the tape was a mixture of multilayer graphene flakes of different thicknesses and lateral dimensions. By repeated peeling, the multilayer graphene was finally cleaved into thin graphene sheets. The tape covered with different layers of graphene was subsequently pressed onto the PMMA beam. As a result, graphene flakes with different thickness and lateral dimensions were obtained on the PMMA beam.

Context 5

Author: Qi Chen

Doi: 10.1038/srep02315

Method:

The average diameter of poly(methly methacrylate) (PMMA) spheres used here is 300 nm. The cationic polyelectrolyte (PEI) is purchased from Alfa Aesar. Graphene Oxide (GO) nanosheets are prepared using a modified Hummer's method from graphite powders. The obtained GO nanosheets are dispersed in deionized water by ultrasonic treatment prior to being used in the experiment. The reference sample of graphene nanosheets is purchased from a commercial source (ACS Material) with purity of ~99.8% and a single-layer ratio of ~ 80%. To prepare reduced Graphene Oxide (rGO) nanospheres, we follow a previous procedure. We modify the surface of PMMA spherical templates with disperser of PEI in aqueous solution. PMMA spheres (5 mg/mL) are dispersed in deionized water containing PEI (2.5 mg/mL) at pH 9.0 under stirring to introduce positive charges at the surface of PMMA spheres. The pH value in the solution is adjusted by dilute HCl or ammonia. After 10 minutes ultrasonic treatment, the suspension is stirred for another 15 minutes to ensure saturated absorption of PEI. To remove the excess PEI, the suspension is further treated by two cycles of centrifugation (8000 rpm at 17% for 5 min) and washing. The PEI-coated PMMA spheres are then dispersed in deionized water and ultrasonically treated for another 10 min. We add a portion of the colloidal suspension of negatively charged GO nanosheets to the turbid PMMA suspension and keep stirring until the supernatant is almost transparent. The sediments are the products owing to the electrostatic interaction of the oppositely charged nanosheets and PMMA surface. The resulting materials are recovered by a separation and washing process, and then dispersed in water by sonication and dried by lyophilization. We place 0.1 g of dried sample in a carbon-powder-surrounding crucible inside a conventional microwave oven in an argon (Ar) atmosphere. The microwave oven (Galanz G80F20CN1L-DG (SO)) functions at full power (800 W, 2.45 GHz) in 200 s cycles (on for 150 s, off for 50 s) for a total reaction time of 600 s. The microwave irradiation reduces GO to graphene, removes the PEI moiety, and decomposes PMMA particles. After reaction, hollow rGO nanospheres are obtained, and trifle PMMA residue is removed with tetrahydrofuran.

RAG-Qwen

In the synthesis of graphene, PMMA (poly(methyl methacrylate)) serves multiple purposes across different methods:

1. **Wei Wu (2010)**: PMMA is used to create a protective layer on graphene during the transfer process. Specifically, it forms a PMMA/graphene/copper sandwich structure, which helps protect graphene when the copper substrate is etched away.

2. **Xin Zhao (2019)**: PMMA beams are used as substrates for mechanical exfoliation of graphene. These beams can be deformed to high strains without fracturing, allowing for the preparation of monolayer graphene on PMMA.

3. **Qi Chen (2012)**: PMMA spheres are used as templates for the modification of graphene oxide (GO) nanosheets. The positively charged PMMA spheres interact electrostatically with the negatively charged GO nanosheets, facilitating their assembly into hollow reduced graphene oxide (rGO) nanospheres.

Each of these uses leverages the properties of PMMA, such as its flexibility and ability to form stable interfaces with graphene, to achieve specific goals in graphene synthesis and functionalization.

Ground truth

PMMA can be pyrolised to decompose into carbon-rich material which can then form graphene sheets through cvd or some other pyrolytic process. PMMA can also be used as a transfer agent to protect and transfer graphene to other surfaces after growth on copper.

Question 16

What is the purpose of plasma cleaning in preparing substrates for high-quality graphene growth?

Context 1

Author: Lam Van Nang

Doi: 10.1149/2.082204jes

Method:

To investigate the rf plasma effect, graphene growth was carried out with a diluted CH_4 gas, in which graphene was not formed without applying plasma. The CH_4 gas of 1 SCCM (denoting cubic centimeter per minute at STP) and the mixed gases of 10% H_2 and 90% Ar (forming gas) of 100 SCCM were introduced in a hot-wall tubular reaction chamber. The Cu foil (Alfa Aesar) was pre-treated with an acetic acid solution at 60°C for 10 min, followed by washing with de-ionized water. The foil was heated to a graphene synthesis temperature (950°C) and annealed for 10 min with forming gas flow (100 SCCM) to remove native oxides from the Cu foil and to enlarge its grains. The synthesis pressure was fixed at 1 Torr. The synthesis time and rf plasma power is 5min and 200 W, respectively. The synthesized graphene on Cu was transferred on SiO_2/Si or glass substrate for various characterizations. First, a 500 nm layer of poly-methyl methacrylate (PMMA) was spin coated on the surface of the graphene film to serve as support. The graphene layer at the back side of the Cu foil was then etched off using oxygen plasma. After the etching of the Cu foil with $FeCl_3$ aqueous solution at 40°C, the PMMA/graphene was transferred to a target substrate. The graphene on the SiO_2/Si or glass substrate was achieved by dissolving the PMMA film using acetone

Context 2

Author: Yong Seung Kim

Doi: 10.1039/C2NR33034B

Method:

The graphene films studied in this work were grown on 25 μm-thick Cu foils by plasma-enhanced chemical vapor deposition (PECVD). The chamber is equipped with an inductively coupled plasma reactor. The radiofrequency (RF) coil, having a diameter of ~25 cm, is located 35 cm above the top surface of the graphite substrate holder. In this geometry, the heating effect of the substrate by plasma was negligible. A residual gas analyzer (RGA 100, Stanford Research System) is attached at the side wall of the chamber to investigate the density of discharged species. A differential pumping technique is employed to meet the required working pressure of the RGA. The system is pumped with a turbomolecular pump (Osaka Vacuum LTD, TG1003), keeping the base pressure as low as ~10^{-7} Torr. Polycrystalline Cu foil was cut into 7 x 7 cm^2 pieces and mounted in the chamber without any pre-cleaning treatment. Five different stages were employed to synthesize graphene films on Cu foil using methane (CH_4) as the carbon source. The Cu substrate was heated to the growth temperature

(700–830 °C) at a heating rate of 3 °C/s. When it reached the target temperature, H_2 gas was introduced into the chamber at a flow rate of 40 standard cubic centimeters per minute (sccm). Hydrogen gas was discharged by an RF power of 50 W for 2 minutes to eliminate surface oxides on the copper foil. Then, the chamber was purged with Ar at a flow rate of 100 sccm for 2 minutes to remove residual hydrogen gas. During the graphene growth stage, radiofrequency (RF) plasma was generated for a specified growth time under a continuous flow of argon (or hydrogen, 40 sccm) and methane (1 sccm), while the pressure was kept at 10 mTorr. The plasma power was varied from 10 to 200 W and the growth time from 0.2 to 4 minutes. Subsequently, the sample was cooled down rapidly to room temperature at a cooling rate of 3 °C/s by turning off the heating power, and then it was taken out for characterization.

Context 3

Author: D.A. Boyd

Doi: 10.1038/ncomms7620

Method:

The copper substrates were placed on a quartz flat inside of quartz tube. A typical substrate size was (8×13) mm^2. The tube was evacuated to 25 to 30 mTorr. A 2-5 sccm flow of room temperature hydrogen gas with 0.4% methane and a comparable amount of nitrogen gas was added and the pressure was controlled at 500 mTorr. The addition of methane to the gas flow was controlled by a precision leak valve, and a typical concentration, as measured by gas chromatography, was 0.4 %. The initially low vacuum conditions (~ 25 mTorr) provided sufficient partial pressures of nitrogen (atmospheric) for the plasma enhanced chemical vapour deposition (PECVD) process. (We note that a leak valve for including purified nitrogen was added to the setup to study the effects of increased nitrogen partial pressures.) Typical partial pressures measured by residual gas analysis (RGA) are as follows: N_2 (Mass 28): 6.9×10^{-8} Torr, CH_4 (Mass 16): 1.5×10^{-7} Torr , O_2 (Mass 32): 5.4×10^{-9} Torr , H_2O (Mass 18): 1.2×10^{-6} Torr, CO_2 (Mass 44): 2.3×10^{-8} Torr. From these values we estimate that the concentration of nitrogen in the gas flow is typically on the same order as that of methane. The PECVD process was found to be highly sensitive to the relative amounts of methane and nitrogen. Excessive methane mixtures resulted in no etching, while excessive nitrogen mixtures would result in excessive etching of the copper. Slight adjustments to the methane concentration could be made via either optical emission spectroscopy (OES) or RGA. A low-power (ranging from 10 to 40 W) cold hydrogen plasma was formed over the copper substrate using an Evenson cavity. Exposure of copper substrates to the plasma enabled continuing etching and cleansing of the copper surface during the graphene growth process. The gas temperature inside the plasma was measured using a thermocouple sheathed in boron nitride. The thermocouple was placed in the plasma above the sample and could be translated along the tube. The peak gas temperature measured in plasma treatment was 160°C at 10 W and 425°C at 40 W, and the gas temperature profile decreased rapidly (by 120°C at 10 W and 425°C at 40 W within 1 cm) from the peak value. The maximum temperature of the copper substrate (Ts) was measured using the melting point of known solids, lead and zinc, and found to be within the range of 327.5°C < Ts < 419.5°C at 40 W. Typically after 5 to 20 minutes of direct exposure to the plasma, a large-area monolayer graphene formed on the backside of copper substrates while the front side of the substrates was coated with disordered graphite. Upon the completion of graphene growth, the plasma was extinguished, and the gas flows were stopped. The process tube was then evacuated and

back filled with Ar, and the substrate with graphene coating was subsequently removed. Copper deposition was visible on the inside of the tube and on the sample holder as the result of plasma etching of the copper substrates.

Context 4

Author: Soo Min Kim

Doi: 10.1088/0957-4484/24/36/365602

Method:

Pre-cleaning step: To remove impurities, the foil was briefly dipped in Ni etchant solution for 30–90 s depending on the number of impurities observed (Note: we have observed that Cu foil having more impurities requires a longer surface cleaning time. However, if the cleaning time is more than 2 min, the copper foil becomes severely damaged and is not useful for growth of graphene.) The main component of the Ni etchant is nitric acid, which reacts with Cu according to Cu(s) + 4HNO3 (aq) -> Cu(NO3)2(aq) + 2NO2(g) + 2H2O. (1) During this reaction, the Cu on the surface is being oxidized and dissolved in the solution; at the same time NO2 gas is violently generated, helping to push the impurity particles away. In this way, the original surface of the Cu foil, together with the impurities, is scraped off and a new, clean but very rough surface is obtained. After cleaning, the copper foil is dipped in DI water for further washing. This washing process is carried out three times, each time with fresh DI water. It should be noted that this step is very important. To remove the DI water from the copper completely, the copper foil is briefly washed with acetone and isopropanol and dried by blowing with N2. Graphene growth procedure: Graphene was synthesized by low pressure chemical vapor deposition using a copper foil (25 μm, 99.8%, Alfa Aesar) as a catalytic metal substrate. After the pre-cleaning process, the copper foil was annealed at 1000°C for 30 min under a 10 sccm hydrogen atmosphere (330 mTorr) to increase the grain size and to obtain a smooth surface, followed by graphene synthesis under 5 and 10 sccm of methane and hydrogen atmospheres for 40 min (450 mTorr) while maintaining the same temperature. During the cooling of the chamber, 10 sccm of hydrogen was flowed until the furnace was cooled to room temperature.

Context 5

Author: Ki Chang Kwon

Doi: 10.1038/srep04830

Method:

Graphene growth: The as-received and electroplated Cu foils were loaded into a thermal chemical vapour deposition (CVD) chamber without precleaning. Graphene samples were grown using methane (CH4) and hydrogen (H2) gas. Under vacuum conditions of 90 mTorr (12 Pa), the furnace was heated without gas flow for 30 min. Before the growth of graphene, the Cu foil was preheated at 950°C for 30 min. In order to increase the grain size of Cu, H2 gas was supplied to the furnace under 150 mTorr (20 Pa) of pressure at a rate of 33 cm3/min (sccm). After the preheating step, a gas mixture of CH4:H2 = 200:33 sccm was supplied under vacuum conditions for 60 min to synthesize the graphene on the Cu foils electroplated by

reusing Cu etchant (R-Cu). The growth time of graphene on the R-Cu foil was reduced to 30 min from 60 min for the as-received Cu foil. After growth, the furnace was cooled to room temperature at a rate of 10–15°C/min under 33 sccm of H2 flow. Poly(methyl methacrylate) (PMMA) or fluoropolymer (CYTOP) was then spin-coated onto the graphene-coated Cu foil as a supporting material and the PMMA (or CYTOP)-coated foil was heated on a hot plate to 180°C for 1 min, after which O2 plasma was used to etch the graphene on the opposite side of the Cu foil. The sample was then immersed in an ammonium persulfate [1M (NH4)2S2O8] bath at room temperature for 3 h to etch away the Cu foil and the etching solution was collected. Then, the remaining PMMA (or CYTOP)-coated graphene was carefully dipped into a de-ionized (DI) water bath 7–9 times to remove any residual etchant. The PMMA (or CYTOP)-coated graphene was then transferred onto a glass substrate. The PMMA (or CYTOP) was removed by an acetone bath at 50°C for 30 min after the PMMA (or CYTOP)/graphene layer had completely adhered to the target substrate.

RAG-Qwen

Plasma cleaning serves multiple purposes in preparing substrates for high-quality graphene growth:

1. **Surface Oxide Removal**: Plasma cleaning helps to remove native oxides from the substrate surface. These oxides can act as nucleation sites for graphene growth, leading to defects and reducing the quality of the graphene film.

2. **Surface Activation**: Plasma exposure can activate the surface of the substrate, enhancing its catalytic properties. This is particularly important for metals like copper, which act as catalysts for graphene growth.

3. **Surface Roughness Modification**: Plasma treatment can modify the surface roughness of the substrate, which can influence the uniformity and quality of the graphene film. Proper surface roughness can promote better adhesion and growth of graphene.

4. **Impurity Removal**: Plasma cleaning can remove impurities and contaminants from the substrate surface, ensuring a clean starting point for graphene growth. This is crucial for obtaining high-quality, defect-free graphene.

5. **Enhanced Adhesion**: By creating reactive species on the substrate surface, plasma cleaning can enhance the adhesion between the graphene and the substrate, which is important for the transfer process.

In the methods described in the provided contexts, plasma cleaning is a common step in preparing copper substrates for graphene growth. For example, in the method by Yong Seung Kim, hydrogen gas is used to discharge and remove surface oxides before introducing methane and hydrogen for graphene growth. Similarly, in the method by D.A. Boyd, a low-power cold hydrogen plasma is used to etch and cleanse the copper surface during the graphene growth process.

Ground truth

Plasma cleaning removes contaminants, organic reside, enhances surface wettability and improves the adhesion of graphene to the substrate.

Question 17

Could you elaborate on the role of copper foil in the synthesis of graphene films using the chemical vapor deposition method?

Context 1

Author: A. Kumar

Doi: 10.1016/j.carbon.2011.11.033

Method:

Graphene film synthesis was performed in a SEKI AX5200S MPCVD (microwave plasma chemical vapor deposition) system with H_2 (50 sccm) and CH_4 (5 sccm) as the feed gases. A 25 μm thick copper foil (Alfa Aesar, 99.8% purity) was used as the substrate. The foil was cut in 2 x 2 cm^2 pieces and placed into the MPCVD chamber, which was evacuated to a base pressure of 2 Torr and then filled with high-purity hydrogen at a pressure of 10 Torr. The Cu foil was supported by a ceramic stand that elevated it from the Mo puck by about 15 mm. The elevation of the Cu foil above Mo puck was found to be critical for the growth of the films because it ensures a strong coupling between the plasma and the Cu foil, thus enabling rapid self-heating of the foil by the microwave plasma. No additional heater was used. The thermocouple attached to the substrate table indicated a maximum reading of approximately 65°C during the process. A dual-wavelength infrared pyrometer indicated a maximum temperature of 700 ± 25 °C. However, as the copper foil is coated with graphene, the surface emissivity changes, making reliable IR measurements difficult. The entire growth is accomplished in less than 5 min and generally involves two stages. First, in a plasma cleaning and annealing step, the foil is kept in 400 W hydrogen plasma for 3 min. A H_2 flow rate of 50 sccm was used with downstream pressure control to maintain 10 Torr chamber pressure throughout the process. This process leads to rapid self-heating and removal of copper oxide from the foil surface. The second stage involves introduction of CH_4 (while maintaining the hydrogen plasma) at a flow of 5 sccm for a short duration (between 30 s and 2 min), providing approximately 10% CH_4 concentration in hydrogen. Depending on the growth duration and whether or not annealing is used, different forms of carbon can be deposited.

Context 2

Author: Richard Piner

Doi: 10.1021/nn4031564

Method:

Graphene films were grown on a 125 μm thick oxygen-free high conductivity (OFHC) copper foil (C10200, Eagle Brass). This foil was cut into 1.5 cm × 3 cm strips that were cleaned using an ultrasonic system (VWR, B2500A-MT) with an acetone bath for 15 min, and then rinsed by acetone and isopropyl alcohol before being blow dried with nitrogen gas. The foil was then placed inside a 0.8 in. ID fused silica tube that was then put inside a 1 in. fused silica tube. (This makes it easy to load/unload the copper foils and clean any evaporated copper from the

small diameter silica tube.) A typical process is as follows: (i) load the Cu foil into the silica tube, evacuate, fill with 5% hydrogen balanced with argon to ambient pressure, heat to 1035 °C for 10 min; (ii) evacuate and fill with pure argon to ambient pressure and heat to 1035 °C for 10 min; (iii) evacuate, fill with pure argon to 100 Torr, heat to 1035 °C, fill the chamber with 5% methane balanced with argon to 150 Torr, keep the temperature at 1035 °C for 10 min and turn off the heating power. The copper foil can reach 1035 °C from room temperature within 2 min, and the cooling rate is typically about 30 °C/s.

Context 3

Author: Soo Min Kim

Doi: 10.1088/0957-4484/24/36/365602

Method:

Pre-cleaning step: To remove impurities, the foil was briefly dipped in Ni etchant solution for 30–90 s depending on the number of impurities observed (Note: we have observed that Cu foil having more impurities requires a longer surface cleaning time. However, if the cleaning time is more than 2 min, the copper foil becomes severely damaged and is not useful for growth of graphene.) The main component of the Ni etchant is nitric acid, which reacts with Cu according to Cu(s) + 4HNO3 (aq) -> Cu(NO3)2(aq) + 2NO2(g) + 2H2O. (1) During this reaction, the Cu on the surface is being oxidized and dissolved in the solution; at the same time NO2 gas is violently generated, helping to push the impurity particles away. In this way, the original surface of the Cu foil, together with the impurities, is scraped off and a new, clean but very rough surface is obtained. After cleaning, the copper foil is dipped in DI water for further washing. This washing process is carried out three times, each time with fresh DI water. It should be noted that this step is very important. To remove the DI water from the copper completely, the copper foil is briefly washed with acetone and isopropanol and dried by blowing with N2. Graphene growth procedure: Graphene was synthesized by low pressure chemical vapor deposition using a copper foil (25 µm, 99.8%, Alfa Aesar) as a catalytic metal substrate. After the pre-cleaning process, the copper foil was annealed at 1000°C for 30 min under a 10 sccm hydrogen atmosphere (330 mTorr) to increase the grain size and to obtain a smooth surface, followed by graphene synthesis under 5 and 10 sccm of methane and hydrogen atmospheres for 40 min (450 mTorr) while maintaining the same temperature. During the cooling of the chamber, 10 sccm of hydrogen was flowed until the furnace was cooled to room temperature.

Context 4

Author: Thomas H. Bointon

Doi: 10.1002/adma.201501600

Method:

25 µm thick copper foils (Alfa Aesar 99.999%) were annealed for 10 minutes at 1035°C in a H2 atmosphere to increase the Cu grain size. A typical processing for the growth of continuous graphene films involves the following steps: (1) heating up the chemical vapour deposition (CVD) system from room temperature to the growth temperature, (2) Cu foil annealing, (3)

graphene nucleation and growth, (4) cooling down the system to room temperature. During the heating up stage, the CVD system was heated to 1035°C in 3 minutes with H2 gas flown at a rate of 0.4sccm and a chamber pressure of 0.01 Torr. The annealing step was performed for 10 minutes at 1035°C in a H2 atmosphere, keeping the H2 gas flow rate at 0.4sccm and the chamber pressure of 0.01 Torr. The temperature was then lowered at 1000°C for the growth of continuous graphene films. A constant flow rate of 0.4sccm of H2 was kept throughout the nucleation and growth. For the nucleation stage, 1.4sccm of CH4 was introduced for 40 seconds. This was followed by the growth stage where the CH4 flow rate was increased to 7sccm for 300 seconds. Finally, the system was cooled down at room temperature keeping the H2 gas flow rate at 0.4sccm.

Context 5

Author: V Miseikis

Doi: 10.1088/2053-1583/2/1/014006

Method:

The 25μm thick Cu foil used in this work, was supplied by Alfa Aesar (purity 99.8%, lot no. 13382). The foil was electropolished in an electrochemical cell made using a commercially available Coplin staining jar as the vessel and an electrolyte solution. The grooves of the staining jar ensured that the foil was kept flat and parallel to the counter electrode (a thicker Cu plate), which helped achieve homogeneous polishing of the surface. Graphene films were synthesized at a pressure of 25 mbar inside a 4 inch cold-wall chemical vapour deposition (CVD) system (Aixtron BM). To achieve more reproducible atmospheric and thermal conditions and to reduce the effective gas flow, the sample was contained in a custom-made enclosure, comprising a quartz disk suspended 6 mm above the sample using graphite spacers. A typical CVD growth process contains four distinct parts: temperature ramp-up (I), annealing (II), growth (III) and cool-down (IV). The annealing as well as the growth was performed at approximately 1060 °C, which was calibrated according to the melting point of Cu. The annealing time was kept at 10 min in all cases. The gas flow during the temperature ramp up and the annealing stages was 1000 sccm. The samples which had the largest grains were annealed in argon atmosphere; other samples were annealed in hydrogen. The gas flow rates during growth were typically set to 1 sccm of methane, 20 sccm of hydrogen and 900 sccm of argon. The growth time was one hour. After the growth, the chamber was cooled in argon/hydrogen atmosphere to a temperature of 120 °C before introducing the samples to air.

RAG-Qwen

In the synthesis of graphene films using the chemical vapor deposition (CVD) method, the copper foil serves as a catalytic substrate. The role of the copper foil is crucial for the successful growth of graphene due to its unique properties and the specific conditions required for the process.

1. **Surface Preparation**: Before graphene growth, the copper foil undergoes thorough cleaning and sometimes electropolishing to remove impurities and create a clean surface. This is essential for ensuring good catalytic activity and uniform graphene growth.

2. **Annealing**: The copper foil is often annealed at high temperatures (typically around 1000-1060°C) in a hydrogen atmosphere. This step helps to increase the grain size of the

copper, promoting a smoother surface and better catalytic activity. Annealing also aids in the removal of surface oxides, which can hinder the growth process.

3. **Plasma Cleaning**: In some methods, the copper foil is exposed to hydrogen plasma to further clean the surface and remove any residual contaminants. This process also leads to self-heating of the copper foil, which is critical for initiating the graphene growth.

4. **Growth Stage**: During the growth phase, the copper foil supports the deposition of carbon atoms from the precursor gas (such as methane) onto its surface. The temperature and gas composition are carefully controlled to facilitate the formation of graphene layers. The copper acts as a catalyst, lowering the activation energy for the carbon deposition process.

5. **Cooling**: After the graphene growth is complete, the system is cooled down slowly to avoid thermal shock and potential damage to the graphene layer. The copper foil continues to play a role in maintaining the structural integrity of the graphene film during this cooling process.

The specific conditions such as temperature, gas flow rates, and the duration of each stage (annealing, growth, and cooling) vary among different methods, but the copper foil remains central to the process. Its role is to provide a suitable surface for catalyzing the graphene growth, ensuring the proper environment for the chemical reactions involved, and facilitating the overall process.

Ground truth

The copper foil usually acts as a substrate and catalyst for growing graphene layers in CVD. It provides a surface on which carbon atoms can nucleate and arrange themselves into a graphene structure.

Question 18

What role does H2O2 play in the synthesis of graphene oxide?

Context 1

Author: Dheeraj

Doi: 10.1016/j.poly.2024.117175

Method:

Graphene oxide was produced employing a modified Hummer's method. Initially, a mixture of H2SO4 and H3PO4 in a 9:1 vol ratio was prepared and placed in a 500 mL round bottom flask, where it was vigorously stirred. Subsequently, 0.55 g of graphite powder was introduced into the acid mixture while maintaining continuous stirring. After achieving complete homogenization, 2.74 g KMnO4 was gradually poured into the solution with vigorous stirring. The solution exhibited a transition to a dark green color during this process, and a small quantity of H2O2 was added dropwise, causing an exothermic reaction. To control the reaction's temperature, the entire process was conducted within an ice bath. Following this, a solution composed of 10 mL of hydrochloric acid and 50 mL of deionized water was introduced into the mixture over a span of 10 min. The resulting residue was carefully collected and subjected to multiple wash cycles with HCl and DI water. Lastly, the precipitated material was desiccated in an oven at 70 °C for a duration of 12 h.

Context 2

Author: Shruti Rialach

Doi: 10.1016/j.electacta.2025.145976

Method:

Synthesis of graphene oxide: Graphene oxide was synthesized via streamlined version of Hummers' Process. A fresh piranha solution was prepared by mixing concentrated sulfuric acid (H2SO4) and concentrated phosphoric acid (H3PO4) in a volumetric ratio of 9:1 in a beaker. The typical concentrations used were 0.81 M of H2SO4 and 0.09 M of H3PO4 to harness a 0.75 M acidic mixture which serves as the medium for oxidation. The solution was ultrasonicated for more than 30 min, followed by a stirring at 500 rpm for another 30 min. Thereafter, 0.675 mM of graphite powder was incorporated into the acid mixture while stirring continuously, ensuring the mixture is uniform and well-dispersed. Slowly, 3.96 mM of potassium permanganate (KMnO4) was introduced into the mixture. The slow addition helps in controlling the exothermic reaction and prevents overheating. Once all the KMnO4 was added, the mixture was maintained under agitation for 22 h to ensure complete oxidation. After the oxidation period, hydrogen peroxide (H2O2) was added to the mixture to reduce the residual KMnO4 and other manganese compounds until the color of the solution turned yellowish-green (2.03 mM of H2O2 was used in the reduction of this mixture). The yellow-green solution was ultrasonicated for 30 min to ensure an intimate contact between the solute and the solvent, and to break down the particles into smaller ones, again followed by

continuous stirring. Next, 100 mL diluted HCl solution (HCl:DI water = 1:3 v/v) was gradually added dropwise to the former solution using a standard rapid transfer port (RTP) three-part syringe inside a well-ventilated fume hood, initiating an exothermic reaction which caused the solution to turn dark brown, indicating the complete reduction process. The final graphene oxide solution was allowed to cool naturally for about 2–3 h in the fume hood.

Context 3

Author: Arjunan Ariharan

Doi: 10.4236/graphene.2016.52005

Method:

Graphene Oxide (GO) was synthesized from graphite by a modified Hummers method. Briefly, graphite powder (2.0 g) was taken in a beaker, added 50 m of concentrated sulfuric acid (H2SO4) under vigorous agitation in an ice bath. Afterwards, sodium nitrate (2.0 g) and potassium permanganate (6.0 g) were slowly added in a sequence. Then, the mixture was transferred into a water bath and kept at 35˚C for 2 hour. After that, 100 mL of distilled water was slowly added, causing a temperature rise to 98˚C. Later, 140 mL of 4% H2O2 was dropped into the reaction system. Finally, the product was washed with distilled water three times. The acquired solid was dried in vacuum at 50˚C for 48 hours.

Context 4

Author: Abhijit Ganguly

Doi: 10.1021/jp203741y

Method:

Highly oxidised Graphene Oxide (GO) was produced using a modified Hummers' process. The starting material Graphite powder with particle size ≤20 μm was purchased from Fluka and is denoted here as "pristine" graphite. All other chemical and reagents were purchased from Aldrich. A mixture of 2.5 g of Graphite and 1.9 g of NaNO3 was placed in a flask cooled in an ice bath. 85 mL of H2SO4 was added to the mixture and stirred until homogenized. Solution of 11.25 g of KMnO4 in distilled water was gradually added to the solution while stirring. After 2 hours, the solution was removed from the ice bath, and further stirred for 5 days. Finally, brown-coloured viscous slurry was obtained. The slurry was added to 500 mL aqueous solution of 5 wt% H2SO4 over 1 hour while being continuously stirred. The mixture was stirred for a further 2 hours. Subsequently, 10 ml of H2O2 (30 wt% aqueous solution) was then added to the mixture and stirred for further 2 hours. This mixture was then left to settle overnight. The mixture was filtered and further purified by dispersing in 500 mL aqueous solution of 3 wt% H2SO4 and 0.5 wt% H2O2. After two days of precipitation, the supernatant solution was removed. This process was repeated five times. The solid product obtained after the rigorous cleaning process was rinsed using copious amounts of distilled water and dried in oven, as reported in literature. The resulting solid was dispersed in water by ultrasonication for 2 h to produce a GO aqueous dispersion. After one-day sedimentation, the thick flakes were removed and the supernatant was collected for further measurements.

Context 5

Author: Benjamin Diby Ossonon

Doi: 10.1039/c6ra28311j

Method:

Graphene oxide was synthesized from natural graphite through the Hummers method which has been improved. The graphite is first pre-oxidized by mixing 5g graphite powder with concentrated sulfuric acid (12.5 mL), potassium persulfate (2.5 g) and phosphorus pentoxide (2.5g). The mixture was heated at 80°C for 6 hours. After dilution with 500 mL of H2O, the mixture was stirred at room temperature overnight. After that, the product is recovered by centrifugation and washed thoroughly with Nanopure water until the filtrate has a pH close to 7. The product obtained is then dried at room temperature for one day. Then, the pre-oxidized graphite is dispersed in H2SO4 (0°C, 115 mL). The temperature of the mixture is carefully controlled to not exceed 10°C. Subsequently, potassium permanganate (15 g) is gradually added with constant stirring for 1 hour. The dispersion is then incubated at 35°C for 2 h and this is followed by the addition of Nanopure water (225 mL) in small portions (15 mL) to control the temperature of the mixture, which must remain below 50°C. To completely dissolve the KMnO4 , hydrogen peroxide (30%, 12.5 mL) was immediately added at the end of a second dilution (H2O, 700mL), and the mixture is stirred for 48 hours. Finally, the suspension is filtered, washed first with HCl (10%) to remove residual metal ions, and repeatedly with Nanopure water until the pH of filtrate becomes neutral. The filtrate is quickly tested by adding a few drops of 1 M NaOH to verify the presence of metal ions in graphene oxide (GO). The product obtained (graphite oxide) is then dried in air. The resultant graphite oxide was dispersed in Nanopure water kept in the ultrasonic bath for 24 hours to maximize exfoliation. The reduced graphene oxide (RGO) is obtained by thermal reduction of GO in Ar/5% H2 at 800°C for 2 h. The resulting RGO can be dispersed in water and the dispersion stayed stable for few hours.

RAG-Qwen

In the synthesis of graphene oxide, hydrogen peroxide (H2O2) plays a crucial role in the reduction step. Specifically, H2O2 is used to reduce the residual potassium permanganate (KMnO4) and other manganese compounds that remain after the oxidation process. This reduction step helps to prevent the formation of unwanted manganese oxides and ensures that the graphene oxide remains stable and free from excessive manganese impurities. Additionally, in some methods, H2O2 is also used to further oxidize the graphene oxide or to control the exothermic reactions that occur during the synthesis process.

Ground truth

It reduces excess oxidisers such as potassium permanganate in variants of the Hummer's method, thus halting oxidation and preventing uncontrolled reactions. It also can promote the formation of grpahene oxide by facilitating the unravelling of carbon chains.

Question 19

Which method would u recommend for synthesizing monolayer graphene for producing transistors?

Context 1

Author: Thomas Moldt

Doi: 10.1021/nn202293f

Method:

Materials: Single-crystal graphite flakes (National de Graphite) 1.7 mm in size have been used to produce graphene. Few depositions have been performed with very large single-crystal graphite flakes, with a size of 5 mm. The graphite flake is cleaved once using sticky tape in order to achieve a clean and fresh surface. The flake is then placed on a microscope coverslip, with a thickness of 120 μm (Menzel-Gläser). The coverslip is cleaned before deposition by sonication in acetone and then 2-propanol. Anodic Bonding Setup: This is composed of a grounded metal block used as back electrode and can be heated to 300 °C using a temperature feedback controlled heating plate. The glass coverslip is placed on the grounded electrode. The top electrode, a cylindrical metal rod with a diameter of 2 mm, mounted vertically above the back gate, is pressed on the graphite flake, while applying a dc voltage for 20–30 min. The setup allows dc voltages of up to 10 kV. After the deposition, thick graphite material is removed from the coverslip by using sticky tape. Anodic Bonding procedure: In the anodic bonding a single crystal flake of graphite is pressed on glass, and a high voltage of 0.5–2 kV is applied between the graphite and a metal back contact, while heating the glass at about 200 °C for 10–20 min. In case of the positive electrode applied to the top contact, a negative charge concentration occurs in the glass at the side facing the positive electrode. A few layers of graphite, including single layers, stick on the glass by electrostatic interaction. The anodic bonding is a simple technique because there are only two deposition parameters: temperature and voltage. Thus, in order to determine the optimum conditions to have high-yield and high-quality single-layer graphene, we made several samples at different temperatures (between 160 and 260 °C) and voltage (between 0.4 and 3 kV). Transfer and Transport: The graphene flakes produced by anodic bonding have been transferred to other substrates by using the wedging technique. We transferred graphene flakes from the coverslip to a silicon substrate covered with 90 nm silicon oxide (IDB Technology) for transport measurements. Electron beam lithography and e-beam evaporation were used to prepare a set of contacts (5 nm Ti/50 nm Au). A Hall bar mesa structure has been prepared by reactive plasma etching.

Context 2

Author: Uk Sim

Doi: 10.1021/acsami.6b11750

Method:

Monolayer graphene was synthesized using thermal chemical vapour deposition (CVD). First, a copper (Cu) foil (10 × 10 cm2) was placed in a quartz tube and heated to 1000 °C for 60 min with flowing H2 at 10 sccm. Then, a gas mixture of 45 sccm CH4 and 10 sccm H2 flowed for 30 min under 10 Torr. After 30 min, the heater was turned off and removed from the Cu foil. Next, to handle the monolayer graphene on the front side of the Cu foil, a protective layer of poly(methyl methacrylate) (PMMA) was spin-coated on the graphene on the front side of the Cu foil, and the graphene on the back was removed using oxygen plasma (100 W RF power, 12 s). Then, the PMMA film on the graphene was removed using acetone, and the remaining Cu was etched away using a 0.1 M ammonium persulfate solution. Finally, the monolayer graphene was rinsed in deionized water and transferred onto a p-type silicon substrate without a silicon oxide layer. For the various stacked graphene layers, graphene with PMMA was floated on deionized water and transferred onto another graphene layer on Cu and etched/rinsed again. The transfer and etching/rinsing procedure was repeated for up to 4 layers of graphene. To synthesize graphene with defects, the monolayer graphene on the back side of the copper was removed using oxygen plasma, and the graphene on the front side of the Cu was treated with argon plasma (10 W RF power, 4 s). The PMMA layer was coated onto this graphene, and the Cu foil was removed. Then, the treated graphene was transferred onto silicon or used in the stacking process.

Context 3

Author: Florian R. Ong

Doi: 10.1088/0957-4484/26/40/405201

Method:

Graphene layers are then obtained by mechanical exfoliation of kish graphite onto a separate Si/SiO2 substrate, which we call the exfoliation substrate. Using Si/SiO2 as a substrate enables the discrimination of one to few layer flakes by inspection with an optical microscope. The number of layers is then confirmed by Rama spectrometry, and a monolayer is selected for the transfer step to follow. A solution of cellulose acetate butyrate (CAB) in ethyl acetate 60 mg/mL, which is a hydrophobic polymer, is dripped onto the substrate, covering the graphene monolayer of interest. The latter is still distinguishable under the optical microscope after the CAB polymer dries off. The needles of the micromanipulator are used to punch markers around the monolayer. These markers will later allow for the alignment of the graphene relative to the gate on the destination substrate, onto which graphene is optically indiscernible.

Context 4

Author: Jian-Hao Chen

Doi: 10.1002/adma.200701059

Method:

First, photolithography is used to prepare 30 nm thick Au source and drain electrodes on a silicon wafer with an oxidized surface (SiO2/Si). The electrodes are then transferred onto the Polyethylene Terephthalate (PET) substrate as described elsewhere. Then, single-and few-

layer graphene is obtained from Kish graphite by mechanical exfoliation on 300 nm thick thermally grown silicon dioxide on silicon substrates, and its thickness and morphology characterized by atomic force microscopy. Mechanical exfoliation yields atomically clean graphene sheets and our AFM images also indicate that the graphene sheet is free of nanometer-scale contaminants. In addition, chemical contamination caused by exposure to photoresist and lift-off chemicals is avoided in this process. The desired graphene sheet is printed at 170°C at 500 psi from the SiO_2/Si substrate to the source-drain electrode assembly on PET.

Context 5

Author: Peiyue Shen

Doi: 10.1088/2752-5724/acbecd

Method:

The graphene samples used in this experiment were mechanically exfoliated from bulk graphite onto the silicon substrate with an oxide layer of about 285 nm. The graphene layer numbers and domains were first selected roughly by optical microscopy and SNOM system with a CO_2 laser (~ 10.6μm), and then confirmed by a homemade Raman spectroscopy system. The selected samples were then made to be the standard field effect transistors by Electron Beam Lithography System and Electron-beam Evaporation System, and the top metal gate is made of 3nm Cr and 30nm Au. Then the samples were annealed at 180℃ with 30 sccm H_2 plasma of 35W power for one hour to remove the organic residues on the surface. Finally, we used Atomic Force Microscopy (AFM) tips at lift mode to remove the remained contaminations on the graphene surface. The force of the AFM tip was set at a tiny value to avoid changing the domain shape.

RAG-Qwen

For synthesizing monolayer graphene suitable for producing transistors, the method described by Uk Sim in the DOI 10.1021/acsami.6b11750 is recommended. This method involves the thermal chemical vapor deposition (CVD) of graphene on a copper foil, followed by careful transfer to a silicon substrate. The process ensures high quality and uniformity, which are crucial for transistor applications. Key steps include:

1. Heating a copper foil to 1000°C to form a catalytic surface for graphene growth.

2. Depositing a controlled amount of methane and hydrogen gas to grow the graphene layer.

3. Removing excess copper and transferring the graphene to a silicon substrate using a combination of plasma etching and chemical cleaning techniques.

This method provides a reliable route to obtain high-quality monolayer graphene with minimal defects, making it well-suited for advanced electronic devices such as transistors.

Ground truth

Micromechanical Cleavage.

Question 20

Which method would u recommend for synthesizing few layer graphene for dispersion into inks?

Context 1

Author: F. Torrisi

Doi: 10.1021/nn2044609

Method:

We prepare the graphene-based printable ink as follows. Graphite flakes are sonicated (Decon bath, 100W) in N-Methylpyrrolidone (NMP) for 9 hours. The unexfoliated flakes are left to settle for 10 mins after sonication. The decanted dispersions are then ultracentrifuged using a TH-641 swinging bucket rotor in a Sorvall WX100 Ultra-centrifuge at 10,000 rpm (~15,000g) for an hour and filtered to remove flakes>1μm, that might clog the nozzle. The resulting ink is characterized by Optical Absorption Spectroscopy (OAS), High Resolution Transmission Electron Microscopy (HRTEM), Electron diffraction and Raman spectroscopy

Context 2

Author: Y. Hernandez

Doi: 10.1038/nnano.2008.215

Method:

We prepared a dispersion of sieved graphite powder in N-methylpyrrolidone (NMP) by bath sonication. After sonication we obtained a grey liquid consisting of a homogenous phase and large numbers of macroscopic aggregates. As with nanotube dispersions, these aggregates could be removed by a mild centrifugation (CF), giving a homogenous dark dispersion. In order to find the concentration after CF, we filtered the graphite dispersion through polyvinylidene fluoride (PVDF) filters. Careful measurements of the filtered mass, accounting for residual solvent, gave the concentration of dispersed phase after centrifugation. This procedure was repeated for three other solvents known to successfully disperse nanotubes: N,N-Dimethylacetamide (DMA), γ Butyrolactone (GBL) and 1,3-Dimethyl-2-Imidazolidinone (DMEU). These dispersions were then characterised by UV-vis-IR absorption spectroscopy with the absorption co-efficient plotted versus wavelength. The spectra are featureless in the visible-IR region as is expected from theory. Each of these four dispersions were diluted a number of times and the absorption spectra recorded. Finally, we have deposited graphene monolayers on SiO_2 surfaces via spray coating, demonstrating that this processing method can potentially be used to prepare samples for microelectronic applications.

Context 3

Author: Emanuele Lago

Doi: 10.1039/C6RA21962D

Method:

Graphene dispersion is produced by liquid phase exfoliation (LPE) of natural graphite. 500 mg of graphite flakes are dispersed in 50 mL of NMP and exfoliated in a sonic bath (VWR Ultrasonic Cleaner USC-THD) for 6 hours. Then, the dispersion is ultra-centrifuged at 10000 rpm (~17000 g), using sediment based separation (SBS) to remove un-exfoliated and thicker graphite flakes, for 30 min at 15 °C with an ultra-centrifuge (Beckman Coulter Optima™ XE-90, equipped with a SW32Ti rotor). Finally, the supernatant is collected by pipetting. A solvent exchange process is carried out for the re-dispersion of the exfoliated flakes in 1,3-dioxolane, a nontoxic and low boiling point (78 °C) solvent, using a Heidolph Hei-Vap rotary evaporator. After the evaporation process of N-methyl-2-pyrrolidone (NMP), the graphitic material is collected and washed three times with acetone to remove the NMP residual using a compact centrifuge (Sigma-Aldrich). The washing step is repeated and the flakes are eventually dispersed in 50 ml of 1,3-dioxolane, adjusting its concentration at 10 mg mL-1.

Context 4

Author: Andrea Capasso

Doi: 10.1016/j.ssc.2015.08.011

Method:

We exploited liquid phase exfoliation of graphite to produce the graphene inks in NMP and in the mixture EtOH/H2O. For the NMP-based ink, 1 g of graphite flakes (Sigma Aldrich) was dispersed in 100 mL of NMP and ultrasonicated (Branson ® 5800) for 6 hours. The obtained dispersion was then ultracentrifuged at ~16000 g (in a Beckman Coulter Optima™ XE-90 with a SW41Ti rotor) for 30 mins at 15 °C, exploiting sedimentation-based separation (SBS) to remove thick flakes and un-exfoliated graphite. After the ultracentrifugation process, we collected the supernatant by pipetting. The optimization of ink-jet printing ideally requires highly concentrated inks. To achieve such a target, the supernatant extracted after the first ultracentrifugation process was further ultracentrifuged at ~200,000g for 60 mins at 15 °C. The high g force value promotes the sedimentation of the graphene flakes at the bottom of the ultracentrifuge tubes taking advantage of the higher density of the graphene flakes (~2.1g/cm3) in comparison with the solvent (ρNMP = 1.03 g/cm3). The pellet (sedimented graphene flakes) is collected and the supernatant is discarded. The pellet was re-suspended in 3 mL of pure NMP using an ultrasonic bath for 10 min. This time was sufficient to re-disperse the graphene flakes, thus obtaining a stable (for more than 2 months) ink. The EtOH/H2O ink was then prepared as follows. 1 g of graphite was dispersed in an EtOH/H2O mixture [1:1 in volume] by ultrasonication for 6 h (Branson® 5800). The mixture was centrifuged at 670g for 10 min (in a Beckman Coulter Optima™ XE-90 with a SW41Ti rotor), longer centrifugation time or higher speed endorses the precipitation of the flakes in dispersion together with non-exfoliated graphitic material. After the centrifugation process, the supernatant was collected by pipetting. In order to get a concentrated graphene ink, the supernatant was ultra-centrifuged at ~16,000g for 15 min at 15 °C. The supernatant was discarded and the pellets were re-suspended in 3 mL of pure EtOH/H2O [1:1] mixture, using an ultrasonic bath for 10 min, to re-disperse the graphene flakes, thus obtaining the final ink. After 1 week, there is the formation

of sediments that are however easily re-dispersed in the same solvent by manual shaking of the bottle containing the ink.

Context 5

Author: Ethan B. Secor

Doi: 10.1002/adma.201502866

Method:

Graphene was exfoliated from graphite using a high shear mixer (Silverson L5M-A) with a square hole high shear screen. Ethyl cellulose (EC) (Sigma–Aldrich, 4 cP grade as measured at 5% in 80:20 toluene: ethanol, 48% ethoxy) was dissolved in ethanol (Koptec, 200 proof) at a concentration of 0.2–2% w/v, and flake graphite (Asbury Graphite Mills, Grade 3061) was added at 10% w/v. This mixture was shear mixed for 2 h at 10,230 rpm in an ice bath, and then centrifuged at 4000 rpm (≈3000 g) for 2 h to sediment out large graphite flakes (Beckman Coulter Avanti J-26 XPI centrifuge). The supernatant containing graphene, EC, and ethanol was harvested by pipette. For the flocculation step, this supernatant was mixed in a 16:9 wt. ratio with an aqueous NaCl solution (0.04 g/mL NaCl, Sigma–Aldrich, >99.5%) and centrifuged for 6 min at 7500 rpm (≈10 000 g) to sediment the graphene/ EC composite. This sediment was washed with deionized water, collected by vacuum filtration (Millipore Nitrocellulose HAWP 0.45 µm filter paper), and then dried to yield the graphene/EC powder, with a graphene content of 25–65 wt% depending on the starting EC loading.

RAG-Qwen

Based on the provided methods, I would recommend the approach described by F. Torrisi (DOI: 10.1021/nn2044609). This method involves sonication of graphite flakes in N-Methylpyrrolidone (NMP) for 9 hours, followed by ultracentrifugation to remove unexfoliated flakes and larger particles. The resulting ink is then characterized by various techniques, ensuring a high-quality dispersion of few-layer graphene.

The advantages of this method include:

1. Extended sonication time, which enhances exfoliation.

2. Effective removal of unexfoliated flakes and larger particles through ultracentrifugation.

3. Characterization steps to ensure the quality of the graphene dispersion.

These features make it suitable for preparing high-quality few-layer graphene inks for various applications.

Ground truth

Pyrolysis

S7. Answer set for Qwen

Question 1

What are the steps involved to synthesize graphene via chemical vapour deposition?

Qwen

1. Prepare the substrate: Clean a suitable substrate (commonly silicon or copper) thoroughly to remove contaminants.

2. Set up the reactor: Assemble the chemical vapor deposition (CVD) reactor and ensure it is properly sealed.

3. Deposition chamber preparation: Evacuate the chamber to create a vacuum and then introduce a precursor gas.

4. Heat the substrate: Raise the temperature of the substrate to the desired level (typically between 900°C to 1050°C for copper substrates).

5. Introduce precursor gas: Introduce a hydrocarbon gas (e.g., methane, ethylene) into the chamber.

6. Growth process: Allow the precursor gas to react on the heated substrate surface to form graphene layers.

7. Cooling and collection: Cool the substrate to stop the reaction and collect the grown graphene.

8. Post-processing: Transfer the graphene from the substrate if needed, and perform any necessary post-treatment steps.

Ground truth

First, a substrate, such copper, is cleaned, prepared and placed into a CVD chamber that is usually a tube made out of an inert material such as quartz. After which, the CVD chamber is evacuated into a vacuum or flooded with inert gases.The CVD chamber is then heated to some kind of critical temperature (about 1000 degrees celsius), before feedstock or precursor gases with high hydrocarbon contents are pumped into the CVD chamber. The high temperature in the chamber decomposes these feedstock gases into carbon atoms which then deposit onto the substrate's surface where they reassemble to into the characteristic graphene lattice. The feedstock gases also produce hydrogen which do not contribute to the reaction. The choice of substrate matters as it plays a role in catalysing the reaction. The temperature and duration of this deposition is controlled to either form mono-layer or few-layer graphene.

Question 2

What are the steps involved to synthesize graphene via mechanical exfoliation?

Qwen

1. Obtain a high-quality graphite crystal, typically from a highly oriented pyrolytic graphite (HOPG) source.

2. Clean the graphite surface using solvents or ultrasonic cleaning to remove contaminants.

3. Use a cleanroom environment to minimize contamination.

4. Employ a micromechanical cleaving technique, often with adhesive tape, to peel off individual graphene layers from the graphite crystal.

5. Transfer the exfoliated graphene onto a suitable substrate, such as silicon dioxide or copper, for further analysis or use.

Ground truth

Mechanical exfoliation of graphene, also known as the sticky tape method, involves first taking a piece of graphite, and then using a piece of sticky tape release a small layer of graphite onto it. This is done simply by placing the sticky tape repeatedly on the piece of graphite. To produce single atomic sheets of graphene, this tape is folded in half and repeatedly pulled apart, thereby shearing layers of graphene from the already deposited material until only a single-atomic layer is present.

Question 3

What are the steps involved to synthesize graphene via plasma enhanced chemical vapour deposition (PECVD)?

Qwen

To synthesize graphene via Plasma Enhanced Chemical Vapor Deposition (PECVD), follow these steps:

1. **Preparation of Substrate**: Clean and prepare a suitable substrate (e.g., silicon dioxide/SiO2/Si, copper foil) in a cleanroom environment to ensure minimal contamination.

2. **Deposition Chamber Setup**: Set up the PECVD chamber with the substrate holder positioned inside. Ensure all components are clean and free from contaminants.

3. **Gas Flow Introduction**: Introduce the precursor gas (typically methane CH4) and an inert carrier gas (such as hydrogen H2 or argon Ar) into the chamber.

4. **Plasma Generation**: Apply a radio-frequency (RF) or microwave power to generate plasma within the chamber. The plasma excites the precursor molecules, initiating chemical reactions.

5. **Temperature Control**: Maintain the substrate at a specific temperature (typically between 600°C and 1000°C) using a heating element or hot plate to facilitate graphene growth.

6. **Deposition Process**: Allow the precursor gases to react in the plasma environment, depositing carbon atoms on the substrate to form graphene sheets.

7. **Cooling Down**: Once the desired thickness of graphene is achieved, turn off the RF power and allow the system to cool down.

8. **Post-Processing**: Remove the substrate from the chamber and perform any necessary post-processing steps such as etching, cleaning, or transfer to another substrate if needed.

9. **Characterization**: Use techniques like Raman spectroscopy, scanning electron microscopy (SEM), and atomic force microscopy (AFM) to characterize the quality and properties of the synthesized graphene.

Ground truth

The plasma-enhanced process is a modification of traditional chemical vapour deposition. As with traditional CVD, a prepared substrate (cleaned and heated to about 1000 degrees celsius) is inserted into a a CVD chamber made out of an inert material and gaseous precursors such as methane are introduced into the reaction chamber together with other inert gases such as H2 or Ar. Often plasma-enhanced CVD can use lower temperatures than traditional CVD. A plasma, typically generated by radio frequency energy at 13.56Mhz is used to excite and disassociate the precursor gases. This process creates highly reactive carbon radicals which deposit and self-assemble ont he heated substreate forming graphene layers. The growth can be tailored by adjusting parameters such as gas flow rates, plasma power and time.

Question 4

What are the steps involved to synthesize graphene via electrochemical exfoliation?

Qwen

1. Prepare graphite oxide or graphite in an aqueous solution.

2. Apply an alternating current between two electrodes immersed in the solution.

3. Exfoliate the graphite oxide or graphite into graphene sheets due to the electric field.

4. Collect the resulting graphene dispersion on a substrate or filter it.

5. Optionally, reduce graphene oxide to graphene if starting with graphite oxide.

Ground truth

First, an electrochemical set up is made involving a graphite working electrode, a counter electrode, a referene electrode and electrolyte and a power supply. All electrodes are inserted into the liquid electrolyte. Using the power supply, either a positive or negative potential is applied to the graphite electrode which either attracts anions (positive potential) or cations (negative potential) from the graphite electrode which cases expansion. The graphene can be subsequently exfoliated by mixing in the precursor.

Question 5

What are the steps involved to synthesize graphene via arc discharge?

Qwen

To synthesize graphene via arc discharge, follow these steps:

1. **Prepare the Electrodes**: Use two graphite electrodes as the source material for carbon.

2. **Vacuum Chamber Setup**: Place the electrodes in a vacuum chamber to eliminate oxygen and other gases that can interfere with the process.

3. **Purging**: Purge the chamber with an inert gas like argon to ensure a clean environment.

4. **Arc Discharge**: Create an electric arc between the graphite electrodes under high voltage, which causes the graphite to melt and evaporate, leading to the formation of carbon nanoparticles.

5. **Cooling and Deposition**: The vaporized carbon condenses on a substrate (such as copper foil) as graphene layers due to the rapid cooling effect.

6. **Substrate Removal**: After the reaction, remove the substrate from the chamber.

7. **Post-Treatment**: Optionally, perform post-treatment steps like annealing or chemical functionalization to improve properties or applications of the graphene.

8. **Characterization**: Analyze the resulting graphene using techniques such as Raman spectroscopy, scanning electron microscopy (SEM), and transmission electron microscopy (TEM) to confirm its quality and characteristics.

Ground truth

Graphene synthesis using arc discharge usises an electric arc to vaporise graphite,followed by the subsequest depostion of the graphene atoms and the formation of graphene flakes. You first need an arc reactor, with two water-cooled electrodes. The anode is usually filled with graphite which the cathode is a graphite rod. An electric arc is then established between the electrodes, using a high current (200A and under a specific pressure (e.g 500 Torr). This high temperature of the arc vaporises the graphite at the anode. The vaporised carbon atoms travel through the arc plasma and deposit on the chamber walls as graphene flakes.

Question 6

What are the steps involved to synthesize graphene via pyrolysis?

Qwen

To synthesize graphene via pyrolysis, follow these steps:

1. Prepare the precursor material, typically a polymer like polyacrylonitrile (PAN) or polystyrene.

2. Heat the precursor in an inert atmosphere (e.g., nitrogen or argon) to a temperature range of 300-500°C to remove volatiles.

3. Further heat the precursor to 700-1000°C under inert conditions to form carbon.

4. Quench the material rapidly to prevent further graphitization and obtain graphene with a high degree of sp^2 hybridization.

5. Exfoliate the resulting material using mechanical or chemical methods to produce graphene sheets.

Ground truth

Pyrolysis is done by first transforming organic materials like biomass into a carbonaceous material followed by further processing into graphene structures. First, biomass sources, such as rice husks or wheat straw are selected and pretreated by grinding, drying or in some cases, treated with chemicals like KOH to enhance carbonization. Pyrolysis is then carried out in a controlled, inert environment in a furnace or reactor. The temperature is gradually increased in the chamber in the reactor (typically between 600 to 1,200 degrees C), which causes the precursor material to decompose, thereby releasing volatile components and leaving behind a carbonaceous residue. The carbonaceous material is then post processed such as acid washing and annealing to improve the material's structural order to become graphene.

Question 7

What are the steps involved to synthesize graphene via liquid phase exfoliation?

Qwen

1. Prepare graphite powder.

2. Disperse graphite in an organic solvent using ultrasonication.

3. Filter the dispersion to remove large particles.

4. Centrifuge the filtrate to collect the graphene sheets.

5. Redisperse the graphene sheets in a suitable solvent for further processing or use.

Ground truth

Graphite is first placed into some kind of liquid suspension medium, often ethanol, alcohol or some other kind of solvent, and then sonicated either through bath sonication or tip sonication. In bath sonication, the graphite-liquid suspension is usually placed into some kind of container before being placed in the bath sonicator. In tip sonication, the tip is inserted directly into the liquid. The suspension is then sonicated for a period of time. During the sonication, cavitation within th eliquids will cause the different graphene layers to exfoliate from one another. After a certain period of time, the suspensions are removed from the sonicator and then centrifuged. The supernatent of the liquid is then taken and characterised to confirm the presence of mono-layer or few-layer graphene. Surfactants can be utilised to maintain or improve the suspension but might cause issues with final device properties.

Question 8

What are the steps involved to synthesize graphene via laser ablation?

Qwen

The steps involved to synthesize graphene via laser ablation typically include:

1. **Preparation of Target Material**: Use a graphite target or other carbon-rich materials.

2. **Setup**: Place the graphite target in a vacuum chamber and position a laser beam.

3. **Laser Ablation**: Apply high-energy laser pulses to the target, causing carbon atoms to vaporize and form graphene nanodroplets.

4. **Graphene Formation**: The vaporized carbon condenses into graphene sheets on a substrate placed near the target.

5. **Collection**: Collect the formed graphene on the substrate.

6. **Characterization**: Analyze the quality and properties of the synthesized graphene using techniques like Raman spectroscopy, scanning electron microscopy (SEM), and transmission electron microscopy (TEM).

Ground truth

Porous graphene can be made using the laser ablation of a nickel-graphite target under the flow of Argon gas. Although not technically a traditional graphene layer, porous graphene is a similar carbon allotrope with an inhomogenous structure and curvature on the graphene layers. First graphite and nickel particles are ball milled and then pressed into a graphite-nickel pellet under pressure to form a target. The target is then placed into a quartz tube where argon is allowed to flow at about 2.4l/min. The tube is then heated to 1050 degrees celsius and presurised at 300mbar. A Nd-YAG laser (1064nm in wavelength), with a 10 ns pulse duration and repetition rate of 10Hz is then focused on the carbon pellet. The illuminater area is about 12 mm2. The carbon atoms generated by this ablation are carried by the argon gas and then gathered on a collector on a water-cooled surface at the end of the tube and scraped off as porous graphene.

Question 9

What are the steps involved to produce graphene oxide?

Qwen

The steps involved to produce graphene oxide typically include:

1. Exfoliation of graphite: Start with graphite flakes and disperse them in an oxidizing agent.

2. Humic acid oxidation: Add humic acid to the dispersion to initiate oxidation.

3. Oxidation process: Gradually add concentrated sulfuric acid and potassium permanganate over time to oxidize the graphite flakes.

4. Exfoliation: Stir the mixture vigorously to exfoliate the graphite into graphene oxide sheets.

5. Washing: Carefully wash the graphene oxide with water to remove residual chemicals.

6. Drying: Dry the graphene oxide to obtain the final product.

Ground truth

Graphene oxide (GO) can be synthesized by the oxidation of graphite into graphite oxide followed by the exfoliation of graphite oxide into GO. The leading method right now is the modified Hummer's method. A common procedure is as follopws: First, mix graphite and potassium permanganate in a 1: 6 ratio by weight. Then, in another beaker, mix 96% sulphuric acid and then add 75% phosphotic acid. The acids should be in a 6:1 ratio. Ensure the acids are added to the graphite and potassium permanganate slowly and bit by bit while constantly stirring. This allows for the intercalation of the graphite and oxidation of the graphite into graphite oxide. Stir the entire solution at 50 degrees centigrade for 12 hours and then allow to cool to room temperature. Next, pour the entire acid mixture into another beaker with ice made from de-ionised water. Add about 3mL of 30% hydrogen peroxide to the mixture and stire to allow the GO to exfoliate.

Question 10

What are the steps involved to produce reduced graphene oxide?

Qwen

To produce reduced graphene oxide (rGO), the following steps are typically involved:

1. **Graphene Oxide Synthesis**: Start by synthesizing graphene oxide (GO) from graphite. This is usually done through a Hummers or modified Hummers method, which involves oxidation of graphite to form GO.

2. **Reduction**: Reduce the graphene oxide to obtain rGO. This can be achieved through various methods such as:

- **Chemical Reduction**: Using reducing agents like hydrazine, sodium borohydride, or ascorbic acid.

- **Thermal Reduction**: Heating GO in an inert atmosphere or in the presence of gases like hydrogen or argon.

- **Plasma Treatment**: Exposing GO to plasma to reduce it.

3. **Purification and Washing**: Purify the reduced material by washing it with solvents to remove any residual reducing agents or impurities.

4. **Characterization**: Characterize the rGO using techniques like Raman spectroscopy, X-ray photoelectron spectroscopy (XPS), and scanning electron microscopy (SEM) to confirm its quality and properties.

These steps provide a general outline for producing rGO, but specific conditions and methods may vary depending on the desired application and properties of the final product.

Ground truth

After GO is produced from the modified Hummer's method the GO can be reduced chemically using a reducing agent such as hydrazine, ascorbic acid or even sugars to reduce it to reduced graphene oxide (rGO). Alternatively, GO can be thermally reduced by heating it up at high temperature in an inert atmosphere like argon or in the presence of hydrogen. This will break down the oxygen-containing groups on the GO causing them to volatilize and restore the carbon framework. it should be nmoted that this process is often incomplete and bcan produce rGOP with a lot of defects that affect conductivity.

Question 11

"What are the steps involved to produce graphene quantum dot?

Qwen

To produce graphene quantum dots (GQDs), the following general steps are typically involved:

1. **Graphene Synthesis**: Start with the synthesis of graphene, which can be done through methods such as chemical vapor deposition (CVD), mechanical exfoliation (e.g., Scotch tape method), or reduced graphene oxide (rGO).

2. **Size Reduction**: Reduce the size of the graphene sheets into smaller particles. This can be achieved through physical (e.g., ball milling) or chemical (e.g., ultrasonication in the presence of reducing agents) means.

3. **Functionalization**: Modify the surface of the GQDs to improve their stability and solubility. This often involves attaching functional groups like carboxyl, amine, or thiol groups.

4. **Size Selection**: Use techniques like size exclusion chromatography or centrifugation to separate GQDs of the desired size range.

5. **Characterization**: Analyze the properties of the GQDs using techniques such as UV-Vis spectroscopy, photoluminescence, and transmission electron microscopy (TEM) to confirm their quantum dot characteristics.

These steps provide a basic framework for producing graphene quantum dots, but specific details can vary based on the exact method and application.

Ground truth

Graphene Quantum Dots (GQDs) can be produced using hydrothermail reactions, laser ablation, microwave radiation and electrochemical oxidation. THe most straightforward is that of electrochemical oxidation. The electrochemical oxidation process involves the electrochemical cleavage of carbon precursores such as graphite into GQDs, typically under high voltage. First a potential is applied to a graphite electrode immersed in an electrolyte made of citric acid and sodium hydroxide. A second graphite rod is used as an cathode in the same solution. A DC power supply can provide the working bias voltage. The GQDs eventually exfoliate and dispersed into the electrolyte. The resulting GQDs can be purified by centrifugation and further techniques such as rotary evaporation.

Question 12

What are the steps involved to produce graphene nanoribbon?

Qwen

The steps involved in producing graphene nanoribbons typically include:

1. **Synthesis**: This can be done through various methods such as top-down or bottom-up approaches. Common techniques include lithography, chemical vapor deposition (CVD), and epitaxial growth on metal substrates.

2. **Pattern Formation**: Using photolithography or electron beam lithography to define the desired width of the graphene nanoribbons.

3. **Graphene Growth**: Growing graphene on a substrate using techniques like CVD or molecular beam epitaxy (MBE).

4. **Pattern Transfer**: Transferring the patterned graphene from the substrate to a suitable material for device fabrication, often involving etching or transfer processes.

5. **Characterization**: Using techniques such as scanning tunneling microscopy (STM), Raman spectroscopy, and electrical measurements to confirm the quality and properties of the graphene nanoribbons.

Ground truth

Graphene nanoribbons (GNRs) are narrow strips of single-layer graphene, essentially 'slices' of graphene that are one-dimensional. They have very high surface areas. They can be synthesized either by top-down approaches such as micromechanical cleavage or bottom-up approaches such as CVD. Top down approaches usually use multi-walled Carbon nanotubes (MWCNTs) to form these graphene nanoribbons. For instance usuing the hummers method on MWCNTs to form the ribbons instead of bulk graphite. Bottom up-approaches using CVD have highly-controlled conditions for growth to ensure orientation and length.

Question 13

What is a method of exfoliating graphene from highly oriented pyrolytic graphite (HOPG) crystal?

Qwen

A common method for exfoliating graphene from highly oriented pyrolytic graphite (HOPG) is mechanical exfoliation, often referred to as "scotch tape" or "sticky tape" method.

Ground truth

HOPG is essentially highly oriented graphite which is ideal for producing graphene using mechanical cleavage with adhesive tape. HOPG can be purchased commercially and then exfoliated through existing processes such as micromechanical exfoliation tape to reult in high-quality graphene with minimal defects.

Question 14

How to synthesize graphene using PET bottles?

Qwen

Synthesizing graphene from PET bottles involves several steps and requires advanced knowledge in chemistry and materials science. Here's a simplified outline:

1. **Preparation of PET**: Cut the PET bottle into small pieces and clean them thoroughly.

2. **Pyrolysis**: Heat the PET pieces in an inert atmosphere (e.g., argon or nitrogen) at high temperatures (around 800-1000°C) to decompose the polymer and form carbon-rich materials.

3. **Graphitization**: Further heat the carbon-rich material at even higher temperatures (around 2500°C) in the presence of a reducing agent to promote graphitization, forming graphene-like structures.

This process is complex and typically requires specialized equipment and expertise. For educational purposes or research, it's recommended to follow detailed protocols from scientific literature or consult with experts in the field.

Ground truth

Waste PET can be cleaned and then used as a precursor for pyrolysis into graphene. It usually takes a 2 step process. First, pyrolysis at 900 degrees centigrade into amorphous carbon. Next, the amorphous carbon is mixed with Boron powder and heat treated at 2400 degrees celsius for 1 hour under helium gas to form graphite. This high-temperature treatment with boron enables the carbon atoms to rearrange into the layered structure of graphite.. Yield rates are about 86%. This graphite can then be liquid-phase exfoliated to form graphene.

Question 15

What is the purpose of PMMA in the synthesis of graphene?

Qwen

PMMA (Poly(methyl methacrylate)) is often used as a supporting layer or a template in the synthesis of graphene to facilitate the transfer process from the growth substrate to another surface, such as silicon dioxide. It helps in handling and transferring graphene without damaging it.

Ground truth

PMMA can be pyrolised to decompose into carbon-rich material which can then form graphene sheets through cvd or some other pyrolytic process. PMMA can also be used as a transfer agent to protect and transfer graphene to other surfaces after growth on copper.

Question 16

What is the purpose of plasma cleaning in preparing substrates for high-quality graphene growth?

Qwen

Plasma cleaning removes contaminants and modifies the surface chemistry of substrates, promoting better adhesion and nucleation sites for graphene, which is essential for high-quality growth.

Ground truth

Plasma cleaning removes contaminants, organic reside, enhances surface wettability and improves the adhesion of graphene to the substrate.

Question 17

Could you elaborate on the role of copper foil in the synthesis of graphene films using the chemical vapor deposition method?

Qwen

Copper foil plays a crucial role in the synthesis of graphene films using the chemical vapor deposition (CVD) method. Here's how:

1. **Substrate for Graphene Growth**: Copper foil serves as the substrate where graphene forms. The smooth surface of high-quality copper foil provides a suitable environment for graphene to grow uniformly and with high crystallinity.

2. **Metal Catalysis**: Copper acts as a catalyst during the CVD process. It facilitates the reduction of carbon-containing gases (like methane or ethylene) into graphene by providing a surface where these reactions can occur more efficiently.

3. **Controlled Growth**: The properties of the copper foil can be controlled, such as its thickness and purity, which allows for precise control over the growth conditions of the graphene film. This is essential for producing high-quality graphene with desired properties.

4. **Post-Synthesis Removal**: After the graphene has been synthesized, the copper foil can be etched away, leaving behind a free-standing graphene film. This is often done using wet chemical etching methods.

5. **Adhesion and Transfer**: The adhesion between the graphene and the copper foil is strong enough to support the graphene during transfer processes but weak enough to allow the graphene to be easily removed from the copper surface without damaging it.

In summary, copper foil is indispensable in the CVD synthesis of graphene films, acting as both a growth substrate and a catalytic surface that enables efficient and controllable graphene formation.

Ground truth

The copper foil usually acts as a substrate and catalyst for growing graphene layers in CVD. It provides a surface on which carbon atoms can nucleate and arrange themselves into a graphene structure.

Question 18

What role does H_2O_2 play in the synthesis of graphene oxide?

Qwen

H_2O_2 (hydrogen peroxide) acts as an oxidizing agent in the synthesis of graphene oxide. It helps to oxidize graphite to form graphene oxide by breaking down the carbon-carbon bonds and introducing oxygen-containing functional groups.

Ground truth

It reduces excess oxidisers such as potassium permanganate in variants of the Hummer's method, thus halting oxidation and preventing uncontrolled reactions. It also can promote the formation of grpahene oxide by facilitating the unravelling of carbon chains.

Question 19

Which method would u recommend for synthesizing monolayer graphene for producing transistors?

Qwen

For synthesizing monolayer graphene suitable for producing transistors, I recommend the chemical vapor deposition (CVD) method. This technique allows for the growth of high-quality, uniform monolayer graphene on copper or silicon carbide substrates, making it a preferred choice for transistor applications.

Ground truth

Micromechanical Cleavage.

Question 20

Which method would u recommend for synthesizing few layer graphene for dispersion into inks?

Qwen

For synthesizing few-layer graphene suitable for dispersion into inks, I recommend the Hummer's method followed by exfoliation using ultrasound. This method effectively produces graphene with good dispersion properties in solvents.

Ground truth

Pyrolysis
